# SteadyCom: Predicting microbial abundances while ensuring community stability

**DOI:** 10.1371/journal.pcbi.1005539

**Published:** 2017-05-15

**Authors:** Siu Hung Joshua Chan, Margaret N. Simons, Costas D. Maranas

**Affiliations:** Department of Chemical Engineering, The Pennsylvania State University, University Park, Pennsylvania, United States of America; Institute for Systems Biology, UNITED STATES

## Abstract

Genome-scale metabolic modeling has become widespread for analyzing microbial metabolism. Extending this established paradigm to more complex microbial communities is emerging as a promising way to unravel the interactions and biochemical repertoire of these omnipresent systems. While several modeling techniques have been developed for microbial communities, little emphasis has been placed on the need to impose a time-averaged constant growth rate across all members for a community to ensure co-existence and stability. In the absence of this constraint, the faster growing organism will ultimately displace all other microbes in the community. This is particularly important for predicting steady-state microbiota composition as it imposes significant restrictions on the allowable community membership, composition and phenotypes. In this study, we introduce the SteadyCom optimization framework for predicting metabolic flux distributions consistent with the steady-state requirement. SteadyCom can be rapidly converged by iteratively solving linear programming (LP) problem and the number of iterations is independent of the number of organisms. A significant advantage of SteadyCom is compatibility with flux variability analysis. SteadyCom is first demonstrated for a community of four *E*. *coli* double auxotrophic mutants and is then applied to a gut microbiota model consisting of nine species, with representatives from the phyla Bacteroidetes, Firmicutes, Actinobacteria and Proteobacteria. In contrast to the direct use of FBA, SteadyCom is able to predict the change in species abundance in response to changes in diets with minimal additional imposed constraints on the model. By randomizing the uptake rates of microbes, an abundance profile with a good agreement to experimental gut microbiota is inferred. SteadyCom provides an important step towards the cross-cutting task of predicting the composition of a microbial community in a given environment.

## Author summary

The microbes residing in the human gut, collectively known as the gut microbiota, have an intimate and complicated relationship with human health. In this study, we aim to enhance the understanding of the relationship between the gut microbiota composition, the metabolite production, the diet, and the metabolic repertoire of the microbes present using genome-scale metabolic models. We have proposed the modeling framework SteadyCom for predicting community compositions, for stable microbial communities growing at a time-averaged constant growth rate. We applied SteadyCom to an example system of *E*. *coli* mutants and a gut microbiota metabolic model consisting of nine representative species spanning the four major phyla in the gut. Dominance by Bacteroidetes and Firmicutes, in particular Clostridia, and the cross feeding of substrates derived from the fermentation of dietary fiber was elucidated. Using a substrate uptake randomization technique, we were able to predict compositions with a striking resemblance to experimental gut microbiota. These results suggested that genome-scale metabolic modeling is a promising tool to predict and analyze gut microbiota compositions and their dependence on nutrient availability.

## Introduction

Metagenomics has brought forth the opportunity for non-culture-based sampling of microorganisms in various environments. It has revolutionized our understanding of microbial communities and their impact on diverse ecosystems and human health. For example, marine microbes have been estimated to contribute half of the flux of global carbon and nitrogen circulation [1] while the diversity of soil microbes has been directly linked to soil health [2]. Microbes that inhabit the human intestine, collectively called the gut microbiota, and their metabolite production, especially short-chain fatty acids (SCFAs), have been found to be of significant importance to intestinal health, immune system, diabetes and weight regulation [3–5]. Mathematical modeling is an indispensable tool for understanding these microbial communities, predicting their behavior and systematically testing different hypotheses. Metabolic modeling of microbial communities has the advantage of predicting interactions at the level of metabolites and metabolic reactions, providing the quantitative means for making optimal interventions.

There are currently two major approaches to predict steady-state metabolic flux distributions in microbial communities using genome-scale metabolic models. The first approach is a direct extension of flux balance analysis (FBA; called joint FBA hereafter), which integrates metabolic reconstructions of individual microbial species into a multi-compartment model with a community compartment allowing for the exchange of metabolites between species [6–9]. The optimization objective function is usually the sum of the biomass reactions of individual species (called the community biomass), often with non-uniform weights. In general, this approach requires additional *ad-hoc* constraints for capturing the observed co-growth behavior. The second class of methods incorporates both community wide and community member objectives. OptCom was used to capture the often conflicting optimization objectives between individuals and the whole community by employing a bilevel formulation [10]. The mass balance equations in OptCom are identical to joint FBA, however the objective function of maximizing the biomass function of an organism is the inner optimization objective function. The community-wide biomass or other alternate community objectives serve as the outer community objective function. OptCom has been applied to the co-growth of two gut microbes, *Bifidobacteria adolecentis* and *Faecalibacterium prausnitzii* [11], as well as for a simple syntrophic relationship between *Desulfovibrio vulgaris* and *Methanococcus meripaludis* and phototrophic microbial mats [10]. Although the bilevel approach requires more computational resources, it can better capture the co-growth behavior of microorganisms compared to the approaches involving simple FBA extensions. The recently introduced Community And Systems-level INteractive Optimization (CASINO) differs from OptCom by incorporating measures of the community network properties to define community objective functions and iteratively optimizes the model at the organism level and community level, allowing for a larger number of organisms to be efficiently simulated [12,13]. Each of the aforementioned approaches has been shown to capture some of the important features of microbial communities, such as competition and cross-feeding.

Nevertheless, there is a fundamental omission in almost all existing FBA based frameworks arising from the fact that the biomass reaction flux is not only a sink for the biomass constituents but also measures the specific growth rate (in h^-1^) and thus the size of the system. For a mono-culture, there is only a single biomass flux that is normalized by the specific rates of consumption or production (in mmol gdw^-1^h^-1^) [14]. However, when multiple organisms are growing together, a joint FBA framework does not necessarily impose any restrictions on the growth rate of all participating members in the community. This lack of constant growth rate across all microbes in the community may lead to metabolic fluxes inconsistent with an unchanging average community composition (termed community steady-state in this article), as the fastest growing organism will take over the population. In the human gut microbiota, experimental studies suggest the existence of stable steady-states around which the gut microbiome stays near [15–18]. A number of seminal modeling studies also assumed the existence of steady-states in the gut microbiome and analyzed their stability [19–22]. The steady-state condition, however, does not imply a constant community composition at every time point. Instead, it approximates the average state of the community over time. Therefore, it is possible that one organism may grow faster at the beginning but after becoming limited by the lack of an essential nutrient another organism may take over. However, no organism consistently outgrows all others as this would result in the species dominating the entire community. Furthermore, joint FBA permits solutions in which a non-growing organism can provide substrates to a growing organism. This predicted interaction is not sustainable, as the feeding capability of the non-growing organism will need to increase in proportion with the increasing amount of biomass of the growing organism. This modeling insufficiency originates from the lack of distinction between two different quantities that describe growth: (1) the specific rate used in single-organism FBA, which captures the amount of substrate utilized per unit time per unit biomass, and (2) the actual exchange rate between the entire population of an organism and the extracellular environment, which represents the total amount of substrate per unit time and is equal to the specific rate multiplied by the biomass of the population (termed aggregate flux in this study). The aggregate flux correctly quantifies the metabolites that an organism can consume or produce in a microbial community of non-uniform relative abundances. The joint FBA framework adopts the specific rate directly to describe inter-organism metabolite exchange instead. Therefore, predictions of community compositions as the ratio of their respective biomass reaction fluxes becomes problematic as it inherently leads to the preferential uptake of carbon substrates by the organism with the highest biomass yield for the limiting substrate. *Ad hoc* constraints coupling the reaction flux with biomass production must then be incorporated to avoid spurious solutions.

The recently proposed community flux balance analysis (cFBA) was an initial attempt to address this aspect by distinguishing between relative abundance and the community growth rate [23]. The nonlinearity introduced and the solution procedure proposed, nevertheless, requires an exhaustive search through a range of relative abundances. The computation remains tractable for only a handful of community participants as the number of sub-problems increases exponentially with the number of organisms. In another study, a similar formulation was proposed for a chemostat culture at a fixed dilution rate [24].

Dynamic simulations incorporate the changing uptake rate of each organism providing an alternative approach to simulating the metabolic activities in microbial communities under steady-state. Dynamic FBA (dFBA) can directly be extended to microbial communities [25,26]. The framework is also suitable for incorporating spatial-temporal considerations as demonstrated in the algorithm COmputation of Microbial Ecosystems in Time and Space (COMETS) [27]. Another dynamic framework, LatticeMicrobes, incorporates spatial-temporal elements to the localization of molecules inside a cell [28]. The bilevel framework d-OptCom has also been developed as an extension of OptCom for dynamic simulation [29]. Reliable uptake kinetic and other parameters are required for accurate predictions by dynamic simulations. Although dynamic simulations are able to describe the dynamic behavior of microbial communities, non-trivial community steady-state (i.e. co-growth behavior in the long run) is not necessarily guaranteed. Also, existing well-established techniques for COnstraint-Based Reconstruction and Analysis (COBRA), e.g. flux variability analysis (FVA) [30,31], flux coupling analysis [32–34], and random flux sampling [35–39] are generally not applicable to dynamic systems. Alternatively, network-based methods [40,41] can be used to infer community properties. Interestingly, the method proposed by Mazumdar *et al*. 2013 [42] can predict the order of colonization based on network similarity, however no information is gleaned on metabolic fluxes.

In this study, we developed a new computational modeling framework called SteadyCom for inferring time-averaged steady-state flux distributions in microbial communities in a tractable manner while being compatible with many tools developed for FBA. SteadyCom directly imposes time-averaged equality of growth rates and apportions ATP maintenance (ATPM) requirements across different microbes in accordance with specific growth unlike joint FBA, OptCom, d-OptCom and CASINO. In addition, SteadyCom is scalable to a large number of organisms as the number of sub-problems to be solved is largely independent of the number of organisms in a community. SteadyCom is first tested in a hypothetical case of the co-growth of four *E*. *coli* mutants. FVA is performed within the framework of SteadyCom to demonstrate its compatibility with existing COBRA techniques for single-organism models. A gut microbiota model consisting of nine species was next considered to predict species relative abundance given the dietary contents as uptake constraints.

## Materials and methods

Joint FBA, the direct extension of FBA, is first stated, followed by the derivation of the SteadyCom approach and an efficient solution algorithm.

### Joint FBA

Let **K** be the set of all organisms in the community. For an organism *k* in **K**, the traditional FBA for predicting maximum growth can be stated as follows:

$$\text{max}\;v_{biomass}^{k}$$

$$\begin{array}{ccc} \text{subject to} & \sum_{j\in {\text{J}}^{k}}S_{ij}^{k}\;v_{j\;}^{k}\;=\;0, & \forall i\in {\text{I}}^{k} \end{array}$$ (1)

$$\begin{array}{cc} LB_{j}^{k}\;\leq \;v_{j}^{k}\;\leq UB_{j}^{k}, & \forall j\in {\text{J}}^{k} \end{array}$$ (2)

where $v_{j}^{k}$ is the flux of reaction *j* (in mmol gdw^-1^h^-1^ for general metabolic reactions, in g gdw^-1^h^-1^ for the biosynthesis of macromolecules, and in h^-1^ for the biomass reaction), $S_{ij}^{k}$ is the stoichiometry for metabolite *i* in reaction *j*, $LB_{j}^{k}$ and $UB_{j}^{k}$ are the lower bound and upper bounds for fluxes $v_{j}^{k}$ respectively, **I** ^*k*^ and **J** ^*k*^ are respectively the set of metabolites and reactions for organism *k*.

Microbial communities have generally been treated as multi-compartment models. There are typically two or more compartments for each organism. One compartment accounts for the extracellular space while the remaining compartments account for the intracellular space(s) (e.g. cytosol, periplasm, mitochondria, etc.). The extracellular compartments of various organisms are connected by an additional compartment (called community space hereafter). The mass balance for metabolites in the community space (termed community metabolites) is described as:

$$\begin{array}{cc} u_{i}^{c}\;-\;e_{i}^{c}\;+\;\sum_{k\in \text{K}}v_{ex(i)}^{k}\;=\;0, & \forall i\in {\text{I}}^{com} \end{array}$$ (3)

where *ex*(*i*) in **J** ^*k*^ is the index of the exchange reaction for community metabolite *i* in organism *k*, $u_{i}^{c}$ and $e_{i}^{c}$ are the community uptake and export rates respectively, and **I** ^*com*^ is the set of shared community metabolites. The objective function for the community model is defined so as to include the sum of the biomass fluxes for each organism:

$$\text{max }\sum_{k\in \text{K}}\alpha^{k}v_{biomass}^{k}$$ (4)

where *α* ^*k*^ is the objective coefficient for the biomass flux of organism *k*. Eqs (1)–(4) form the joint FBA optimization formulation for assessing the metabolic flows in microbial communities. Eq (3), however, implicitly assumes that each species has an identical biomass or relative abundance by modeling the metabolite exchange in the community space as the direct sum of the specific rates of individual organisms. Another potential problem of the above formulation is that a stable steady-state in the community is not guaranteed. As discussed in the introduction, metabolic flux distributions satisfying the community steady-state cannot be derived under this treatment. To remedy this shortcoming, we derived a necessary and sufficient condition for the requirement of a constant community composition (i.e. community steady-state). Under the assumption of identical dilution rates for all organisms in the community, the condition is simplified into an identical specific growth rate for all organisms (see S1 Text for the general condition and its derivation):

$$\begin{array}{cc} v_{biomass}^{k}\;=\;\mu , & \forall k\in \text{K} \end{array}$$ (5)

where *μ* is the community growth rate. Note that the requirement of identical growth rate between all microbial partners at steady-state applies not at every time point but averaged over the time interval of the study. Possible departures from identical growth rate in a stable microbial community are possible when one or more microbial partners enter or leave the system at different rates. For example, one gut microbe may elute slower because it is closer to the wall of the intestinal tract. In the absence of organism-dependent dilution rates, identical growth rates is a reasonable approximation for gut microbiota as experimental results showed that fecal and large intestine microbiota are quite similar [43]. The generalized analysis is presented in S1 Text where individual dilution rates are defined for each microbe.

### Deriving SteadyCom constraints

A community-modeling framework was derived to include a more accurate representation of the community space, a constraint to enforce steady-state, and a restriction to force zero flux through an organism with zero abundance. In the framework, the biomass (in gdw) for organism *k* is explicitly modeled as the variable *X* ^*k*^. A new flux quantity called the aggregate flux $V_{j}^{k}$ for reaction *j* of organism *k* is introduced:

$$\begin{array}{cc} V_{j}^{k}\;=\;X^{k}v_{j}^{k}, & \forall j\in {\text{J}}^{k},\;k\in \text{K} \end{array}$$ (6)

The aggregate flux $V_{j}^{k}$ has units of mmol h^-1^ and represents the collective flux of reaction *j* through the entire population of organism *k*. This differs from $v_{j}^{k}$ which is the reaction flux normalized by the biomass of organism *k*. The rate of consumption or production of a community metabolite *i* in **I**^com^ in the community space is captured by the sum of aggregate fluxes $V_{ex(i)}^{k}$ instead of $v_{ex(i)}^{k}$. By introducing the aggregate flux, the exchange of community metabolites between individual organisms in the community space can be properly expressed with the following mass-balance equation in the community space analogous to Eq (3):

$$\begin{array}{cc} u_{i}^{c}\;-\;e_{i}^{c}\;+\;\sum_{k\in \text{K}}V_{ex(i)}^{k}\;=\;0, & \forall i\in {\text{I}}^{com} \end{array}$$ (7)

$V_{ex(i)}^{k}$ represents the transport reaction fluxes from the extracellular space into the individual community member *k* taking into account the abundance of the organism. To maintain linearity for the problem, all individual microbe models are not expressed on a per unit of biomass, but are scaled to the abundance of each organism. Therefore term $V_{ex(i)}^{k}$ quantifies the contribution of each microbe *k* to the community-wide balance of metabolite *i* as denoted by terms $u_{i}^{c}$ and $e_{i}^{c}$. Eqs (1) and (2) can be expressed in terms of the aggregate flux by multiplying each equation by *X* ^*k*^ for each organism *k* in *K*:

$$\begin{array}{cc} \sum_{j\in {\text{J}}^{k}}S_{ij}^{k}V_{j}^{k}\;=\;0, & \forall i\in {\text{I}}^{k},k\in \text{K} \end{array}$$ (8)

$$\begin{array}{cc} LB_{j}^{k}X^{k}\;\leq \;V_{j}^{k}\;\leq \;UB_{j}^{k}X^{k}, & \forall j\in {\text{J}}^{k},k\in \text{K} \end{array}$$ (9)

This establishes the mass balance and flux capacity constraints in terms of the aggregate flux and biomass variables. The lower and upper bounds in Eq (9) have the same ranges as in single-organism models that are generally only restricted by the directionality of the associated reaction. They respectively represent the minimum and maximum specific activities of a reaction in units of mmol gdw^-1^h^-1^. Starting from Eq (5) the community steady-state condition is restated so as to relate *X* ^*k*^, *μ* and the aggregate biomass production $V_{biomass}^{k}$:

$$\begin{array}{cc} V_{biomass}^{k}\;=\;X^{k}\mu , & \forall k\in \text{K} \end{array}$$ (10)

Note that if $V_{j}^{k}\;=\;X^{k}\;=\;0$ for all *j* and *k*, Eqs (7))–(10) are satisfied regardless of the value of *μ*. To avoid this a non-zero total biomass *X*_0_ for the community is defined:

$$\sum_{k\in \text{K}}X^{k}\;=\;X_{0}$$ (11)

### SteadyCom

Using Eqs (7)–(11), the maximum community growth rate *μ*_max_ of a community satisfying the community steady-state can be found by solving the following non-linear optimization problem termed SteadyCom:

$$\begin{array}{lll} \begin{array}{ccc} \text{max} & \mu & \end{array} & & \\ \begin{array}{cc} \text{subject} & \text{to} \end{array} & \begin{array}{cc} \left[\begin{array}{ll} \sum_{j\in J^{k}}S_{ij}^{k}V_{j}^{k}=0, & \forall i\in I^{k}\\ LB_{j}^{k}X^{k}\leq V_{j}^{k}\leq UB_{j}^{k}X^{k}, & \forall j\in J^{k}\\ V_{biomass}^{k}=X^{k}\mu & \\ X^{k}\geq 0 & \end{array}\right] & \forall k\in K \end{array} & \\ & \begin{array}{cc} u_{i}^{c}-e_{i}^{c}+\sum_{k\in K}V_{ex(i)}^{k}=0, & \forall i\in I^{com} \end{array} & \\ & \sum_{k\in K}X^{k}=X_{0} & \\ & \begin{array}{cc} \begin{array}{ccc} \mu & , & e_{i}^{c}\geq 0, \end{array} & \forall i\in I^{com} \end{array} & (\text{SteadyCom)} \end{array}$$

For convenience the community export rates *$e_{i}^{c}$* and uptake rates $u_{i}^{c}$ are normalized for one unit of total community biomass, therefore *X*_*0*_ is set at 1 gdw and *X* ^*k*^ is thus equal to the relative abundance of organism *k*.

SteadyCom can be viewed as a generalization of FBA. By setting *X* ^1^ = 1 and *X* ^*k*^ = 0 for *k* > 1, SteadyCom is reduced to the standard single-organism FBA model and the aggregate biomass flux coincides with the specific growth rate. Similar to single-organism FBA, constraints on the system uptake rates $u_{i}^{c}$ are sufficient to guarantee a finite solution (i.e. finite *μ*_max_). In addition, physiologically relevant constraints on organism-specific uptake rates can be imposed whenever available. In this study, since uptake kinetics are not directly modeled, we impose constraints on the system-wide uptake rates for limiting resources. Whenever required to match known information we also impose constraints on organism-specific uptake rates as noted in the Results section. Predictions by SteadyCom are in general different from the predictions by joint FBA or OptCom because of the constraints relating biomass, the bounds for specific rates, the aggregate fluxes and the community growth rate. The flux distributions predicted by SteadyCom satisfy two important properties that are fundamentally different from the prediction by joint FBA. First, the community steady-state encoded in Eq (10) enforces an identical time-averaged growth rate for all organisms in the community such that the predicted community composition remains stable over time. Second, the coupling between the biomass and the aggregate flux by Eq (9) ensures that for a growing community, an organism can have non-zero fluxes if and only if both its total biomass and biomass production rate are non-zero. A non-growing organism in a growing community will quickly become extinct and therefore it will be unable to contribute to community metabolite exchange at a community steady-state. Though nonlinear, SteadyCom becomes a linear program (LP) once the community growth rate *μ* is fixed. SteadyCom can be solved iteratively by checking the feasibility of the LPs at various values of *μ* (generally less than 10 iterations are required for an accuracy of 10^−6^ or less). The algorithm and conditions assuring the global maximum are presented and discussed in detail in S1 Text. The optimization model implemented as functions in Matlab using CPLEX is available in S1 Dataset or at https://github.com/maranasgroup/SteadyCom.

### Extending constraint-based analysis to SteadyCom

Established constraint-based modeling techniques can be applied directly to SteadyCom after finding *μ*_max_ by fixing *μ* at any value between 0 and *μ*_max_ as all constraints in SteadyCom become linear. FVA was performed by minimizing and maximizing targeted objectives [30]. Note that this setting also allows the variability in the biomass *X* ^*k*^ to be analyzed, which is a key focus in this study. FVA under the SteadyCom framework thus requires that the objective function is changed to the reaction fluxes/biomass variables to be analyzed while the community growth rate is fixed at an explored value:

$$\begin{array}{ll} \begin{array}{cc} \text{max}/\text{min} & \end{array} & \sum_{\begin{array}{l} j\in J^{k}\\ k\in K \end{array}}w_{k,j}^{V}V_{j}^{k}+\sum_{k\in K}w_{k}^{X}X^{k}\\ \begin{array}{cc} \text{subject} & \text{to} \end{array} & \mu =\mu_{0}\\ & \text{Constraints in SteadyCom} \end{array}$$

where $w_{k,j}^{V}$ is the weight for the flux of reaction *j* of organism *k*, $w_{k}^{X}$ is the weight for the biomass of organism *k* and *μ*_0_ is between 0 and *μ*_max_. For example, to analyze the variability of the relative abundance of organism *k’*, set $w_{k\prime }^{X}\;=\;1$ and all other $w_{k}^{X}\;=\;w_{k,j\;}^{V}\;=\;0$.

### Genome-scale models

The genome-scale model *i*AF1260 for *E*. *coli* was employed to test the applicability of SteadyCom [44]. Nine models of nine organisms as proxies for four major phyla (Bacteroidetes, Firmicutes, Proteobacteria and Actinobacteria) present in the gut microbiome were selected to form a gut microbiota model (Table 1). Seven of the organisms used are among the most abundant genera in human gut: *Bacteroides* (18%), *Faecalibacterium* (7.6%), *Eubacterium* (3.9%), *Streptococcus* (3.7%), *Escherichia* (2.8%), *Lactobacillus* (2.8%), *Bifidobacterium* (2.5%) from the recent integrated catalogue of reference genes in the human gut microbiome [45]. *Enterococcus* is also a common genus seen in the gut [45,46]. The genome-scale metabolic model of *Klebsiella pneumoniae* has been used previously to study gut microbiota [8,9]. It was selected as a proxy of the genus *Klebsiella*, which is often found in the human gut [47]. Minor corrections were made to the models to fix mass balance inconsistencies and eliminate thermodynamically infeasible cycles involving ATP generation and proton gradient generation [48,49]. In particular no changes for the uptake systems were made. The compiled microbiota model is available in S1 Dataset.

**Table 1:** The nine species and their genome-scale reconstructions used in the gut community model.

Species	Phylum	Model
*Bacteroides thetaiotaomicron* (*B*. *thetaiotaomicron*)	Bacteroidetes	*i*AH991 [7] updated in [9]
*Eubacterium rectale* (*E*. *rectale*)	Firmicutes	*iEre*400 [24]
*Faecalibacterium prausnitzii* (*F*. *prausnitzii*)	Firmicutes	*i*Fpraus_v1.0 [50]
*Enterococcus faecalis* (*E*. *faecalis*)	Firmicutes	V583 [51]
*Lactobacillus casei* (*L*. *casei*)	Firmicutes	*i*Lca12A_640 [52]
*Streptococcus thermophilus* (*S*. *thermophilus*)	Firmicutes	*i*MP429 [53], updated in [9]
*Bifidobacterium adolescentis* (*B*. *adolescentis*)	Actinobacteria	*i*Bif452 [11]
*Escherichia coli* (*E*. *coli*)	Proteobacteria	*i*JO1366 [54]
*Klebsiella pneumoniae* (*K*. *pneumoniae*)	Proteobacteria	*i*YL1228 [55], updated in [9]

### Estimation of average American diet and community uptake rates

Upper bounds for community uptake rates in the unit of mmol h^-1^ were estimated by the average daily consumption of food (g day^-1^) published by USDA [56] multiplied by the chemical composition of food (mmol g^-1^) available in the USDA national nutrient database [57]. The rates were normalized by a total dry weight of 10 g for the gut microbiota, which was estimated from the recently revised number of microbial cells in an average human [58] multiplied by the dry weight per bacterial cell (BioNumber, BNID 106615) [59,60]. The estimated carbon-containing nutrients were divided into four categories of macronutrients: carbohydrates, amino acids, dietary fiber and fatty acids. The amount of each category available to the gut microbiota was reduced by a percentage representing host absorption, which is estimated from dividing the fecal excretion rate of the macronutrient [61] by the estimated uptake rate from diet: 90% for amino acids; 95%, 97% or 99% for carbohydrates, 0% for dietary fiber and 90% for fatty acids. The results presented in the main text are obtained assuming absorption of 97% of carbohydrate. See S3–S6 Figs for the corresponding results for 95% and 99% carbohydrate absorption by the host. See S1 Diet for the detailed estimation of the rates.

## Results

The potential of SteadyCom to predict species abundance and perform constraint-based analysis in community models with community steady-state implemented was first demonstrated in the hypothetical case of the co-growth of four *E*. *coli* triple mutants using the genome-scale metabolic reconstruction *E*. *coli i*AF1260 [44]. SteadyCom was then applied to a gut microbiota model consisting of nine species to predict the composition of gut microbiota given the dietary information.

### Co-growth of E. coli auxotrophic for amino acids

**Fig 1:**
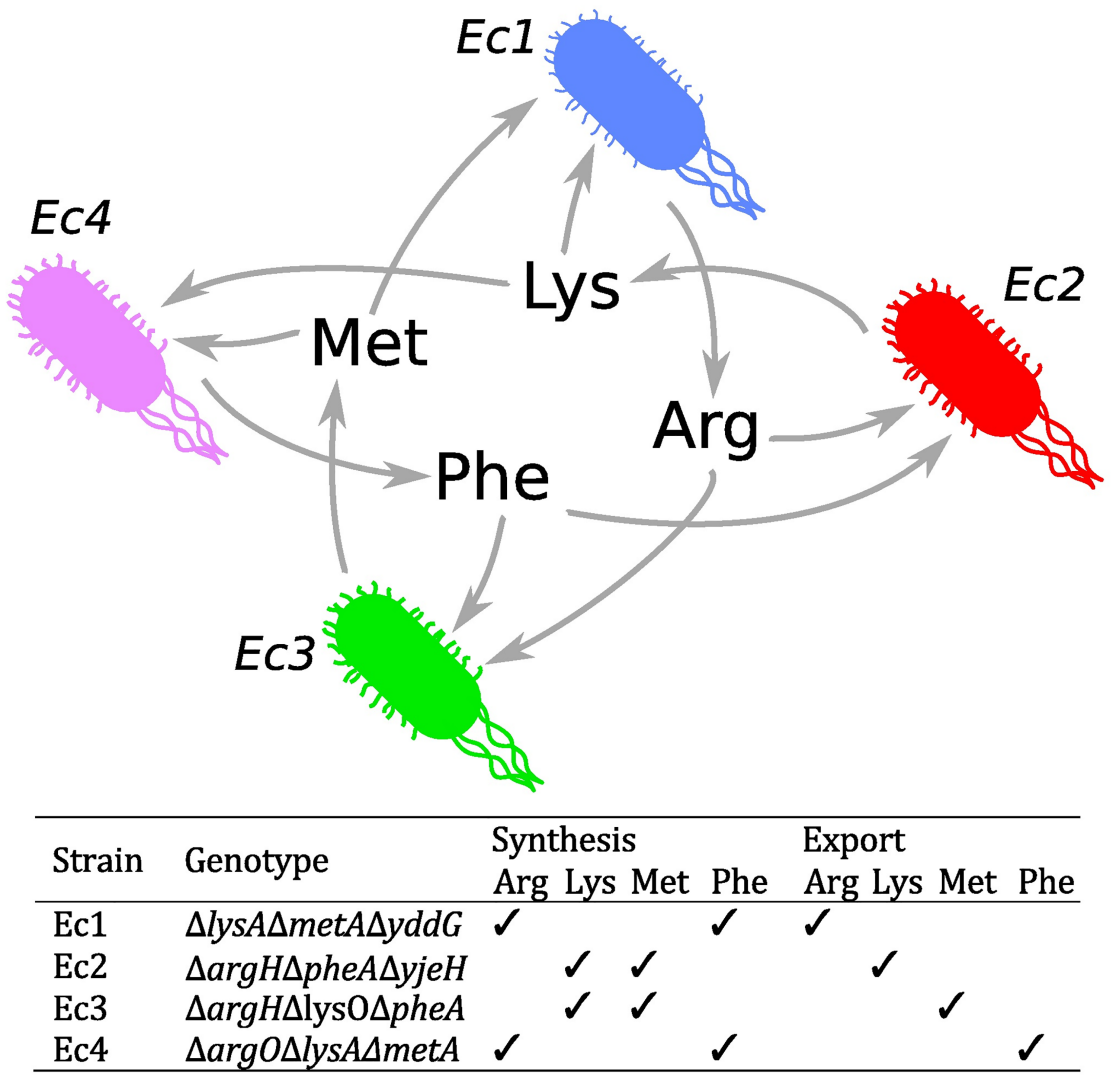
A hypothetical microbial community of four *E*. *coli* mutants. Each *E*. *coli* mutant is auxotrophic to two amino acids and produces one amino acid that is essential to the community. The genotype and ability to synthesize and export the focus amino acids are displayed.

**Fig 2:**
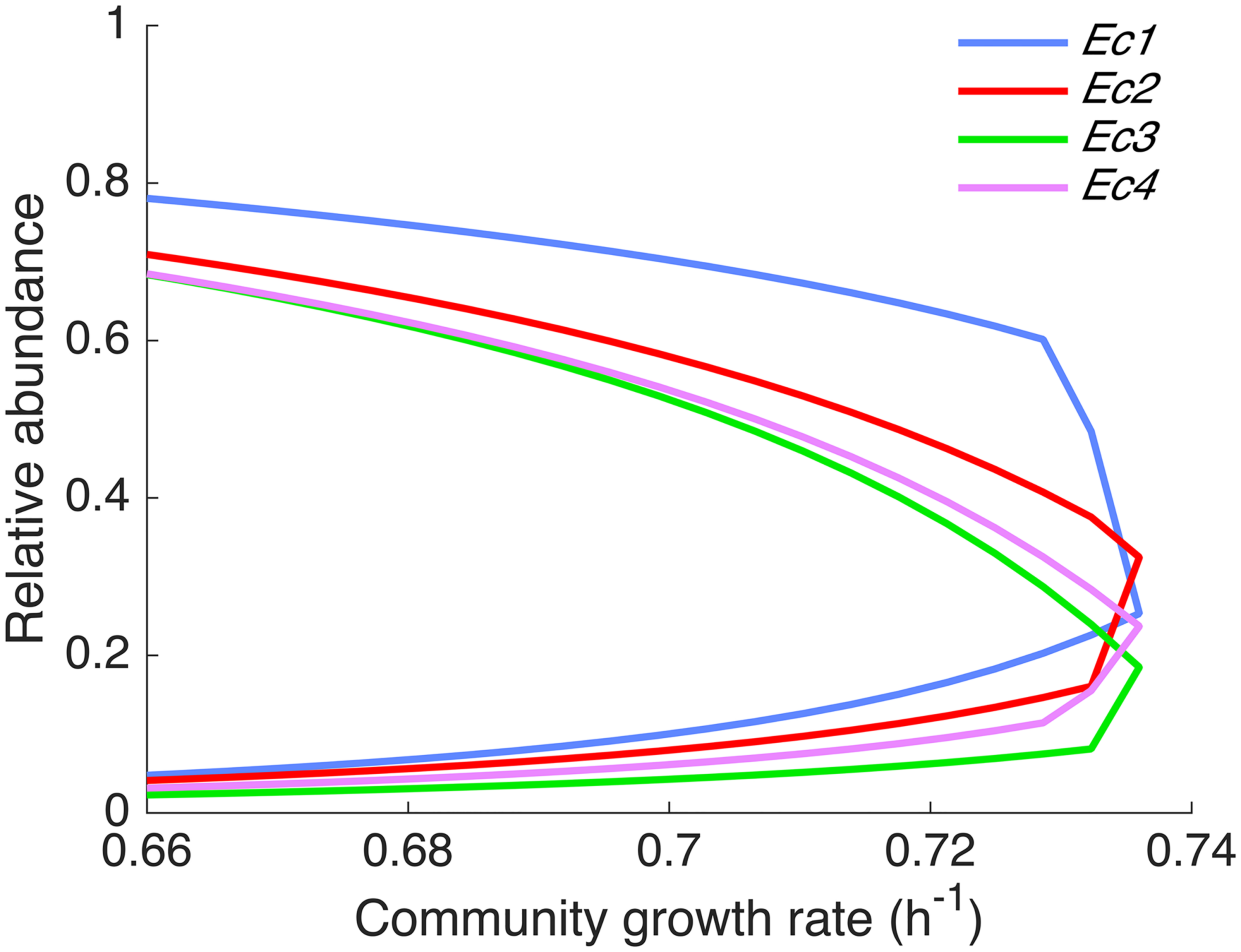
Flux variability analysis at ≥90% maximum community growth rate using SteadyCom. The maximum and minimum relative abundance of each *E*. *coli* mutant is displayed for various community growth rates. Each mutant is essential at maximum community growth rate at a defined relative abundance.

SteadyCom is a reformulation of cFBA [23] with the computational advantage that the number of LPs to be solved is independent of the number of organisms in the community as required by cFBA. Another important feature of SteadyCom is compatibility with FVA [30]. This enables the determination of the range of allowable fluxes and organism abundances while imposing the requirement of constant growth rate. These methodological advantages were demonstrated in the community of auxotrophic *E*. *coli* mutants. For the co-growth of auxotrophic *E*. *coli* mutant pairs analyzed using d-OptCom [62], the same maximum community growth rate and biomass ratio of the two strains were found using SteadyCom and cFBA (S1 Table). A more complex hypothetical case involving the cross feeding of four *E*. *coli* triple mutants originating from this study was then analyzed. Solutions using cFBA were not computed because of the high computational cost for this four-membered community. For each solution, cFBA requires solving ~10^5^ LPs given a 1% change in relative abundance in each step. The community consists of four *E*. *coli* mutants (*Ec1*, *Ec2*, *Ec3* and *Ec4*) each auxotrophic for two amino acids and devoid of the exporter of one amino acid (Fig 1). Each mutant competes with another mutant for the amino acids produced by the other two mutants. Co-growth is theoretically possible and every mutant is essential for community survival and growth. The maximum growth rate predicted by joint FBA was 0.572 h^-1^ while the prediction by SteadyCom was 0.736 h^-1^. This significant deviation was found to be a result of the non-growth-associated ATPM requirement in the model. In joint FBA, the predicted flux distribution needed to fulfill the ATPM requirement for four units of biomass (Eq (2), $v_{ATPM}^{k}\;\geq \;LB_{ATPM}^{k}$ for all mutants), leading to the underestimation of the maximum growth rate. In contrast, the flux distribution predicted by SteadyCom satisfied the ATPM requirement for one unit of biomass in total (Eq (9), $V_{ATPM}^{k}\;\geq \;LB_{ATPM}^{k}X^{k}$ for all mutants with the sum of biomass being one). The allowable ranges of the relative abundance of the mutants at ≥ 90% of the maximum community growth rate computed by flux variability analysis (FVA) indicate the essentiality of each mutant for growth (Fig 2) using SteadyCom. The ranges converge to a unique community composition as the community growth rate increases to its maximum. In contrast, joint FBA optimizing for an unweighted sum of biomass predicts that each of the mutants can have abundances ranging from 0 to 100% for ≤ 99% maximum community growth and only the growth of *Ec2* and *Ec3* are necessary at 100% maximum community growth (S1 Fig).

The underlying reason for the difference is the community steady-state condition imposed in SteadyCom. Since all mutants must produce some amino acids for other mutants, all mutants must grow if they are to co-feed the other mutants as shown in Eqs (9) and (10). In other words, all mutants must grow simultaneously with non-zero biomass in order to achieve any level of community growth. In joint FBA, however, there is no connection between the biomass and the exchange fluxes. Each mutant can produce amino acids even without growth. Joint FBA may therefore find solutions irrespective of the growth of individuals. This renders the prediction by joint FBA to be an initial response in the community but not a community that has reached its steady-state.

**Fig 3:**
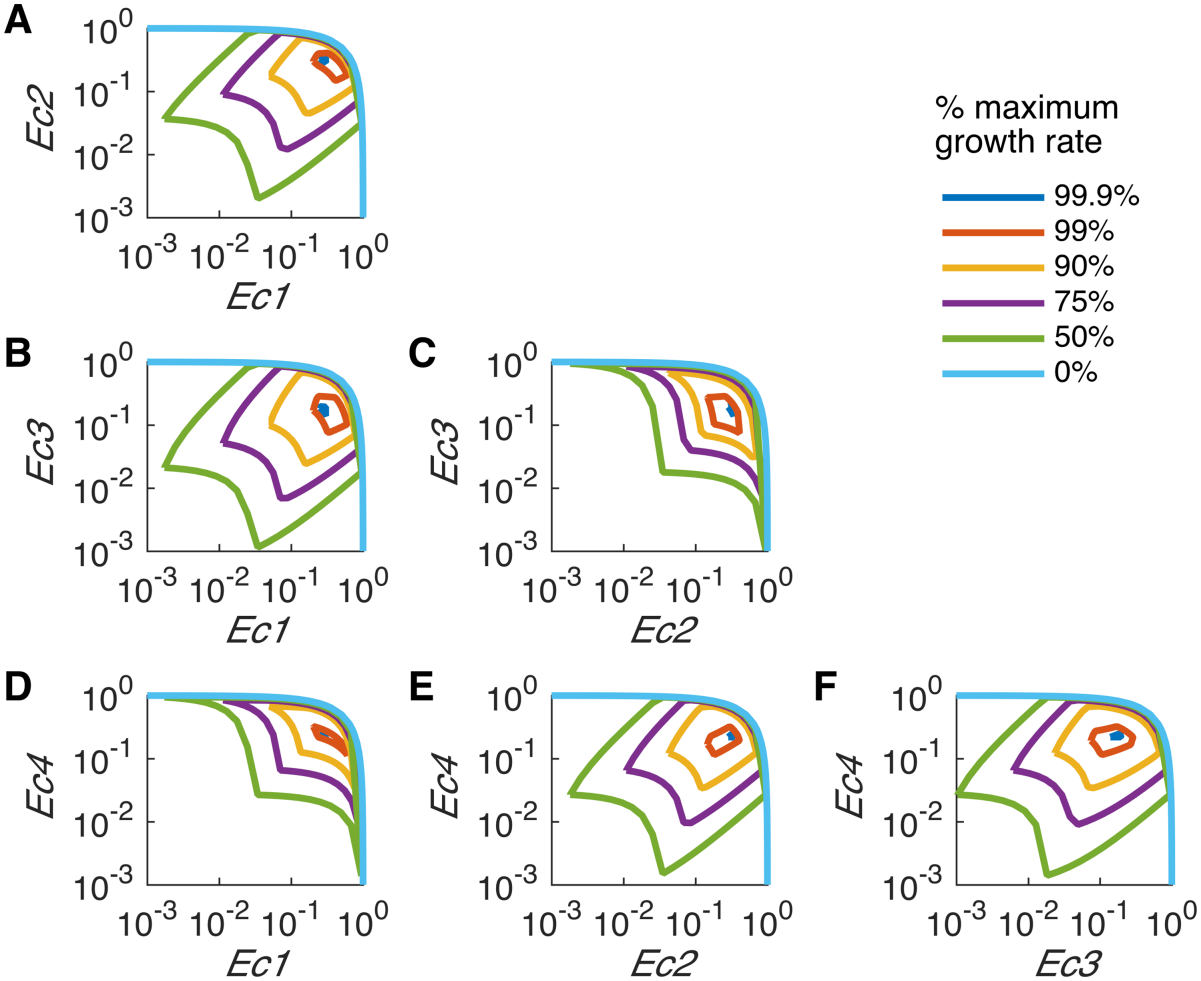
Flux variability analysis of the relative abundance of each pair of mutants. At a given percentage of the maximum community growth rate, the relative abundance range of the dependent organism was calculated by iteratively fixing the x-axis organism’s relative abundance from 10^−3^ to 1. (A) *Ec2* vs *Ec1*, (B) *Ec3* vs *Ec1*, (C) *Ec3* vs *Ec2*, (D) *Ec4* vs *Ec1*, (E) *Ec4* vs *Ec2* and (F) *Ec4* vs *Ec3*. Two patterns were observed: purely competitive (*Ec3* vs *Ec2*, *Ec4* vs *Ec1*) and conditionally mutualistic (the remaining pairs).

The conditional dependency between mutant abundances was assessed for various community growth rates by iteratively fixing the abundance of one mutant at increasing values and computing the allowable range of the abundance of the other mutants (Fig 3). At zero growth rate, the flux through the biomass reaction of each organism is constrained to zero, so the only requirement that the community must satisfy is meeting the ATPM requirement for each mutant with non-zero abundance. The sum of the maximum abundances is always one reflecting a unit of total biomass (Eq (11)) while the minimum abundances of all mutants is zero. No binding relations are suggested at this point because each mutant can satisfy their own ATPM requirement independently. As the community growth rate increases, however, the coupling between mutants becomes tighter and abundances converge to unique values at maximum growth rate. Two different types of patterns are observed. For pair *Ec1*, *Ec4* (Fig 3D) and pair *Ec2*, *Ec3* (Fig 3C), the abundance of one mutant is in direct conflict with the other mutant (as designated by the negative slope in the entire region) indicating a competitive relation between these pairs. As seen in Fig 3D, when either *Ec1* or *Ec4* is high, the *Ec2* and *Ec3* mutants are relatively low (because the sum of all abundances is equal to one), so high competition occurs between *Ec1* and *Ec4* as they rely on the lysine and methionine produced by *Ec2* and *Ec3*. However, with relatively higher abundances of *Ec2* and *Ec3*, lysine and methionine are more abundant alleviating, but not negating, the competition between *Ec1* and *Ec4*. For pair *Ec1*, *Ec2*, pair *Ec1*, *Ec3*, pair *Ec2*, *Ec4* and pair *Ec3*, *Ec4* (Fig 3A, 3B, 3E and 3F, respectively), synergism is observed within the region where abundances are positively correlated. A conditionally cooperative relation is therefore suggested by FVA while smaller regions of competition are still observed when the mutants in a pair have similar abundances. By further examining the *Ec1* and *Ec2* pairs (Fig 3A), the production of Arginine by *Ec1* is beneficial to *Ec2* and the production of lysine by *Ec2* is beneficial to *Ec1*, so synergy exists when the abundances of *Ec1* or *Ec2* are high. However, when all mutants have similar abundances, competition occurs as expected by the competitive nature of the community.

FVA and SteadyCom are able to reveal the context-dependent nature of interaction (i.e., competition or cooperation) between two organisms in a community model. Interestingly, this relatively simple hypothetical community containing elements of both cross feeding and competition suffices to demonstrate that the nature of interaction depends on individual growth levels. Heinken *et al*. has previously employed a similar pareto optimality analysis to study the tradeoff between the growth of species in gut community models [7–9]. In their studies, joint FBA models were used and additional constraints coupling certain reactions were required for non-trivial results (the presence of correlation). In SteadyCom, constraints coupling reaction fluxes and growth emerge from the community steady-state imperative and the direct tracking of biomass (gdw), growth rate (h^-1^), reaction rate (mmol h^-1^) and specific reaction rate (mmol gdw^-1^h^-1^).

### Nine-species model for gut microbiota

A community model consisting of nine microbes present in the human gut with available genome-scale metabolic reconstructions was compiled. The organisms include one species in the phylum Bacteroidetes, five species in Firmicutes (two Clostridia and three lactic acid bacteria), two species in Proteobacteria and one species in Actinobacteria (*B*. *adolescentis*) as detailed in Table 1. In the assembled community model, *B*. *thetaiotaomicron* and *F*. *prausnitzii* are the only organisms able to digest dietary fiber.

**Fig 4:**
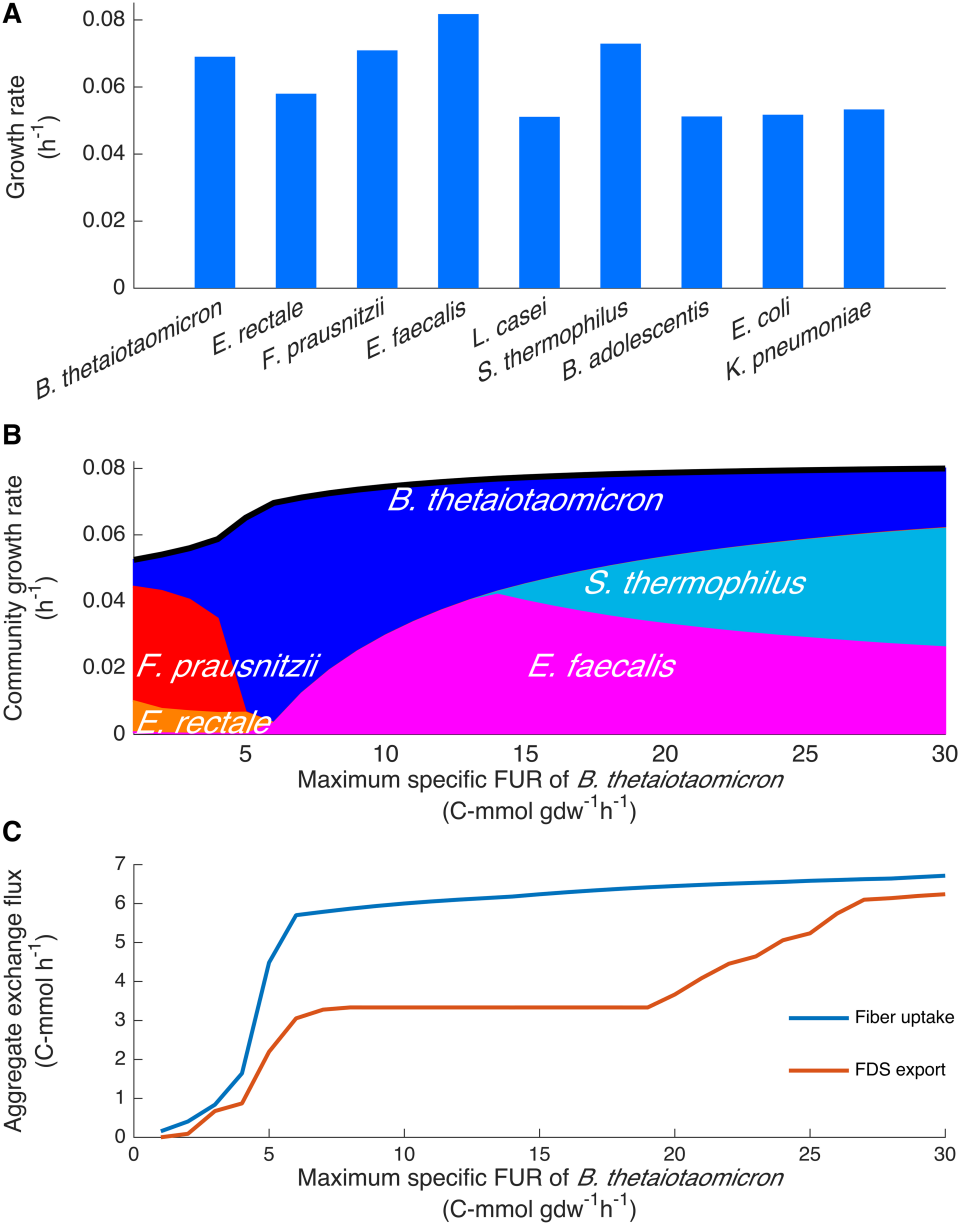
Simulation of the gut microbiota model subject to the estimated average American diet. (A) The maximum possible growth rates were predicted for each species using joint FBA by maximizing the biomass reaction of each species individually. (B) The maximum community growth rate (the black curve) and species composition (filled area) were predicted by SteadyCom at varying maximum specific fiber uptake rate (FUR) of *B*. *thetaiotaomicron*. (C) Aggregate fiber uptake and fiber-derived substrate (FDS) export by *B*. *thetaiotaomicron* that are required for maximum community growth were calculated using FVA.

Using a set of community uptake bounds derived from an average American diet estimated in this study (see Materials and methods), the maximum possible growth rate of each species was determined by maximizing the biomass reaction of each species in turn under the joint FBA framework. Each species is able to grow (Fig 4A) with the two lactic acid bacteria (LAB) *S*. *thermophilus* and *E*. *faecalis* having the highest growth rate. The growth rate computed here is suitable only for comparing the maximum possible growth yield between species under the nutrient condition (which becomes useful in explaining the results that follow), not for predicting growth rates within the community.

SteadyCom was next applied to the gut community model. As dietary fiber is the major carbon source, the microbiota composition for maximum growth under carbon limitation was simulated with the maximum specific fiber uptake rate (FUR) constrained to 5 C-mmol gdw^-1^h^-1^ for *F*. *prausnitzii* and constrained to various levels for *B*. *thetaiotaomicron*. *B*. *thetaiotaomicron* produces fiber derived substrates (FDSs), such as glucose, fructose, etc. (S3 Table) by the exoenzymes secreted to the extracellular space [8,63]. These FDSs are the primary carbon sources available for uptake by other community members as a large portion of amino acids, carbohydrates and fatty acids are absorbed by the host [3,64]. Fig 4B shows the maximum community growth rate and the species composition (represented by the proportion of the filled area) at varying maximum specific FUR of *B*. *thetaiotaomicron*. Only *B*. *thetaiotaomicron*, *F*. *prausnitzii*, *E*. *rectale*, *S*. *thermophilus* and *E*. *faecalis* have abundances above 0.1%. FVA on the range for species abundances showed that the predicted compositions are unique with no allowed variance. At low FURs of *B*. *thetaiotaomicron*, the dominance of *B*. *thetaiotaomicron*, *F*. *prausnitzii* and *E*. *rectale* resembles the dominance of Bacteroidetes and Firmicutes [65–68] with high abundance of Clostridia [67,68] in the human gut microbiota. As the FUR of *B*. *thetaiotaomicron* increases above 5 C-mmol gdw^-1^h^-1^, *B*. *thetaiotaomicron*’s abundance decreases and the two LAB begin to dominate the population. LAB require the FDSs from *B*. *thetaiotaomicron*, which explains the necessary and appreciable abundance of *B*. *thetaiotaomicron*. *B*. *thetaiotaomicron* is capable of exporting a non-decreasing amount of FDS at a lower abundance because of the higher specific FUR and meanwhile the fewer FDSs required for *B*. *thetaiotaomicron*’s growth (Fig 4C). If substrate exchange between species is independent of their abundances, the two LAB are expected to have high abundances, as they have the highest biomass yield (Fig 4A). In fact, joint FBA predicts non-zero abundances only for *E*. *rectale* and the two LAB, while *B*. *thetaiotaomicron* digests fiber and exports FDSs at high rates without growth (S5 Fig). Interestingly, the shift from *B*. *thetaiotaomicron* to the LAB at high FURs is similar to the effect of supplementing xylanase-pretreated fiber (arabinoxylan) to an *in vitro* culture of human gut microbiota by which the abundance of *Bacteroides* spp. and *Clostridium* spp. decreases and *Bifidobacterium* increases [69]. More FDSs are available in the simulation due to the increase in *B*. *thetaiotaomicron*’s FUR (Fig 4C), which allows for higher substrate availability for microbes with no or low fiber-fermenting activities. The value of 5 C-mmol gdw^-1^h^-1^ for the FUR of *F*. *prausnitzii*, slightly lower than 1 mmol gdw^-1^h^-1^ glucose uptake, was chosen because in a previous study [11] the growth of *F*. *prausnitzii* was analyzed for glucose uptake rate ranging from 0 to 1 mmol gdw^-1^h^-1^. Values ranging from 5 to 30 C-mmol gdw^-1^h^-1^ for the FUR of *F*. *prausnitzii* were also tested and similar shift in abundances was observed (see S6 Fig).

By constraining only the maximum specific FURs of *B*. *thetaiotaomicron* and *F*. *prausnitzii*, SteadyCom can capture interesting interactions in the gut microbiota. At low FURs of *B*. *thetaiotaomicron*, the dominance of *B*. *thetaiotaomicron*, *F*. *prausnitzii* and *E*. *rectale* resembles the dominance of Bacteroidetes and Firmicutes in human gut [45,65,70]. However, the prediction that *S*. *thermophilus* and *E*. *faecalis* can dominate the microbiota at high FURs is not consistent with the experimental observations. This highlights the importance of the constraints on organism-specific uptake rates. Maximizing the community growth rate given a constant total biomass favored the growth of *E*. *faecalis* and *S*. *thermophilus* because they can generate biomass more economically from the given nutrients (Fig 4A). The low biomass yield organisms simply convert substrates into metabolites that are then taken up by the high biomass yield organisms without growing appreciably. As a result, the low biomass yield organisms maintain physiologically prohibitive high specific rates of uptake or export to sustain the cross feeding relationship. In light of this, we tested an approach to impose randomized and physiologically relevant bounds for organism-specific substrate uptake rates in the absence of the actual experimental uptake rates. The results are presented in the next section. In addition, a complementary approach that constrains the relative abundances of the minority of the community known from experimental data was tested. It used partial information on the abundance of the microbes to assess the response of the proxy organisms for Bacteroidetes and Firmicutes as well as short-chain fatty acid (SCFA) production to changes in diet. See S2 Text, S7 and S8 Figs for the detailed results.

### Randomly sampled uptake bounds

**Fig 5:**
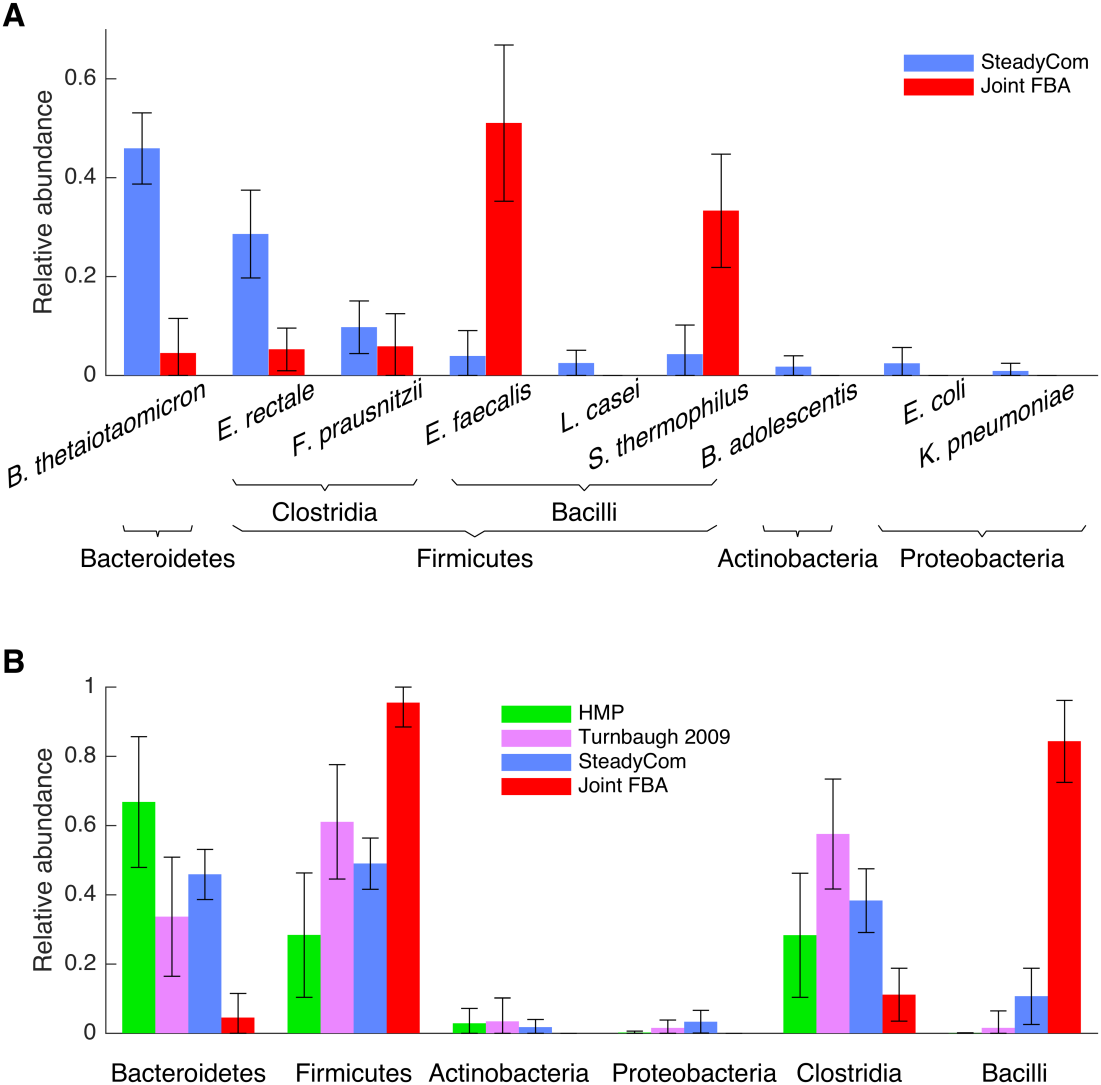
Distribution of the gut microbiota abundances simulated using 1000 sets of randomly assigned carbon uptake bounds for each species given the estimated average American diet. (A) Species abundances simulated using SteadyCom (blue) and joint FBA (red) respectively are displayed. (B) Comparison to the two sets of American gut microbiota data respectively from the Human Microbiome Project [70] (green) and Turnbaugh *et al*., 2009 [65] (purple) at the phylum level. Two known important classes in Firmicutes, the Clostridia and Bacilli are also included.

The method of bounding the maximum abundances of minor species, though able to capture some features of the interactions within the gut microbiota, still does not simulate a realistic microbiota composition which is dominated by Bacteroidetes and Firmicutes with low abundances of Actinobacteria and Proteobacteria (experimentally observed to be 5–10%) [45,46,65,68,70–72]. In addition, the constraints on species abundances are largely *ad hoc*. We expect that physiologically relevant constraints on the nutrient uptake rates for each microbe will result in predictions that are more representative of the community because unrealistically high uptake rates are ruled out as discussed in the previous subsection. All results presented so far have only constrained the specific FUR. All other specific uptake rates were set to arbitrarily large values to allow the uptake of nutrients to be based on organism requirements and nutrient availability. To test the effect of adding the uptake constraints on the prediction by SteadyCom in an unbiased way in the absence of the experimental uptake rates, 1000 sets of maximum specific uptake rates for each carbon source of each species were randomly sampled. The technique of randomly sampling model parameters and comparing the result statistics has been applied extensively before (i.e., ME-models [73], FBA with molecular crowding constraints [74,75], etc.). See S2 Text for more details. SteadyCom was solved for the gut microbiota model subject to the estimated average American diet. The average distribution among the 1000 random sets has a striking similarity to reported experimentally determined microbiota compositions [45,46,65,68,70–72]. Dominance by proxy species for Bacteroidetes (*B*. *thetaiotaomicron*) and Firmicutes (*F*. *prausnitzii*, *E*. *rectale*, *S*. *thermophilus*, *E*. *faecalis*, *L*. *casei*) with the majority among Firmicutes consisting of proxy species for Clostridia (*F*. *prausnitzii*, *E*. *rectale*), as well as the low but non-zero abundances of proxy species for Actinobacteria (*B*. *adolescentis*) and Proteobacteria (*K*. *pneumoniae*, *E*. *coli*) were predicted. Fig 5A displays the abundance of each species, while Fig 5B lumps the species into their phyla and compares four conditions: the simulation results using SteadyCom and joint FBA, and the experimental results of the American gut microbiota composition data from the Human Microbiome Project [70] and Turnbaugh *et al*., 2009 [65]. Joint FBA computed for the same conditions predicts *S*. *thermophilus* and *E*. *faecalis* as the dominating species and non-zero abundances only for *B*. *thetaiotaomicron*, *F*. *prausnitzii* and *E*. *rectale* (Fig 5A). This is a consequence of the higher growth yield of these species in the model (Fig 4A). A set of random uptake rates was selected to perform the analysis of systematically varying the contents of amino acids, fiber and carbohydrate in the diet (S10 Fig). See S2 Text for more discussion.

**Fig 6:**
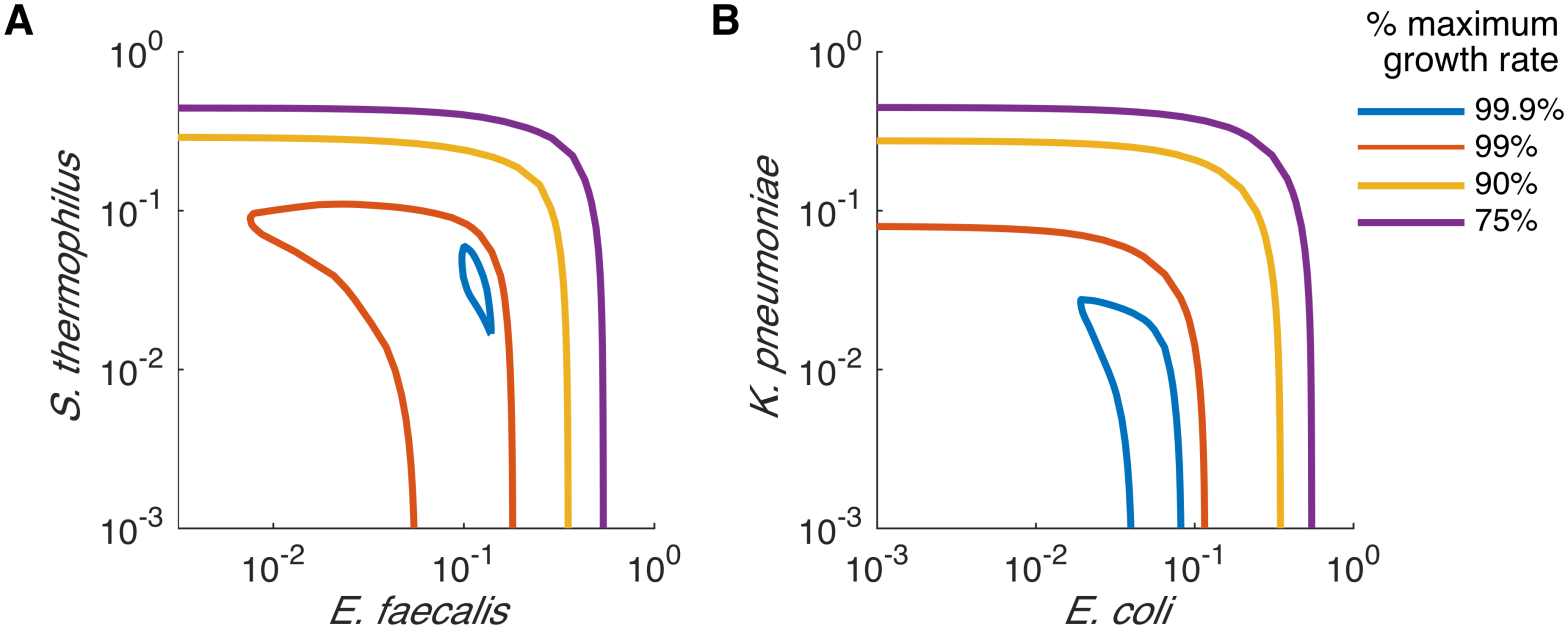
Flux variability analysis between two pairs of closely related species performed at various growth rates. Flux variability was calculated at 75%, 90%, 99% and 99.9% of the maximum growth rate utilizing the SteadyCom framework and a set of random uptake bounds given the estimated average American diet as nutrients to the community. The relationships (A) between *E*. *faecalis* and *S*. *thermophilus*, and (B) between *E*. *coli* and *K*. *pneumoniae* are displayed.

Given the more diverse community profile, it is necessary to apply FVA to examine the relationships between each pair of species. The analysis reveals two pairs of strongly competing species (Fig 6). The first pair, *S*. *thermophilus* and *E*. *faecalis*, has a negative correlation in the majority of the range (Fig 6A) while the second, *K*. *pneumoniae* and *E*. *coli* has negative correlation in the entire range (Fig 6B). Interestingly, the species within each pair are also closely related to each other relative to the other modeled species. *S*. *thermophilus* and *E*. *faecalis* are both lactic acid bacteria, while *K*. *pneumoniae* and *E*. *coli* are both Proteobacteria. The competition can be explained by the consumption of similar resources by the species in light of their close relatedness. This is similar to the intraspecific competition in ecological terms.

## Discussion

Using the nine proxy models, SteadyCom was able to predict the universal dominance of Bacteroidetes and Firmicutes with non-zero abundances for Actinobacteria and Proteobacteria given a typical diet [45]. With randomizing the uptake rates of microbes, an abundance profile of the phylum proxies similar to the experimental phylum distribution was predicted. A recent study comparing vegans and omnivores from an urban USA area found surprisingly similar gut microbiota compositions between the two groups [76]. There have also been conflicting results regarding the role of the Bacteroidetes-to-Firmicutes ratio and its change under different host conditions in lean or obese individuals [5]. The interactions in the model may not apply to every possible gut microbiota in humans. More accurate predictions would require refined models consisting of more species. In particular, the proxy models serve to represent defined aspects of the phylum (e.g. the Bacteroidetes and Clostridia act as primary fiber-fermenting microbes that indirectly feed others with simple carbohydrates).

Both the community growth rates predicted by SteadyCom (Fig 4B) and the maximum growth rates for each species predicted by joint FBA (Fig 4A) given community uptake rates based on the consumption and chemical composition of the average American diet, lie in the range of the intestinal microbial growth rates reported (i.e. 0.02–0.25 h^-1^) [77]. This consistency supports the validity of constraint-based modeling frameworks based on the mass balance of biochemical conversion and the potential for qualitative and quantitative predictions of gut microbiota metabolism. The current analysis aims to demonstrate the applicability of SteadyCom for predicting species abundance and extending the constraint-based modeling technique to microbial communities with the community steady-state. The model is simplified by the small number of proxy models compared to the over 1,000 species present in the gut microbiota. To more accurately predict gut microbiota composition in the future, a host cell model needs to be integrated into SteadyCom to account for their interactions.

Despite the aforementioned challenges, SteadyCom has distinct advantages as an important framework and algorithm for simulating microbial communities. An important practical advantage of SteadyCom is that the number of LPs required to solve SteadyCom depends only on the desired precision of the maximum growth rate and the solution’s distance from the initial guess. This is an improvement over the cFBA in which the number of LPs solved increases exponentially with the number of organisms in the community model [23]. SteadyCom gives more reasonable predictions over joint FBA which has been used in analyzing microbial communities previously [6–9,78], as a result of the community steady-state and the explicit modeling of the biomass variable to correctly describe the relationships between biomass (*X*^*k*^), biomass production rate ($V_{biomass}^{k}$), growth rate (*μ*) and exchange fluxes ($V_{ex(i)}^{k}$). SteadyCom is compatible with the constraint-based modeling techniques established for metabolic models of single organisms allowing for the use of established techniques to analyze the community. Here we demonstrated the extension of FVA to determine both synergistic and antagonistic relationships between the auxotrophic *E*. *coli* mutants. By performing FVA on pairs of abundance/flux or flux/flux variables, positively and negatively correlated variables can further reveal potential synergistic and antagonistic interactions. By randomly sampling the solution space of feasible flux distributions at different community growth rates, correlations can be discovered at a large scale. Overall, we propose that metabolic modeling of microbial communities should exploit the community steady-state and the linearization techniques applied in SteadyCom. As the number of community participants increase, it is essential to have scalable methods that correctly impose stability requirements for community models.

## Supporting information

S1 Fig Flux variability analysis at ≥90% maximum community growth rate using joint FBA. Joint FBA finds the same FVA range for *Ec1* and *Ec4*, and for *Ec2* and *Ec3*, respectively. None of the mutant is predicted to have non-zero abundance necessary for ≤99% maximum community growth.
(TIF)

S2 Fig Flux variability analysis at ≥90% maximum community growth rate using SteadyCom at various maximum amino acid uptake rates The maximum and minimum relative abundances of (A) *Ec1*, (B) *Ec2*, (C) *Ec3* and (D) *Ec4* are displayed for various community growth rates for various maximum amino acid uptake rates. The ranges for different maximum specific uptake rates of amino acids overlap when the growth rate is close to the maximum (≥0.732 h^-1^). The lower limit of *Ec1* and the upper limits of *Ec2*, *Ec3* and *Ec4* remain the same regardless of the maximum uptake rate. The results shown in the main text correspond to the simulation performed at maximum specific uptake rates of amino acids equal to 1 mmol gdw^-1^h^-1^.
(TIF)

S3 Fig Maximum possible growth rates at various levels of carbohydrate available to the gut microbiota predicted for each species using joint FBA. Predictions were obtained by maximizing the biomass reaction of each species individually, given the estimated average American diet with 1% (blue), 3% (red) or 5% (yellow) of carbohydrate available to the gut microbiota after absorption by the host.
(TIF)

S4 Fig Simulation of the gut microbiota model subject to the estimated average American diet at various levels of carbohydrate available to the gut microbiota performed using SteadyCom. (A) Relative abundance, (B) aggregate fiber uptake and (C) aggregate fiber-derived substrate (FDS) export by *B*. *thetaiotaomicron* are displayed. Relative abundances of (D) *F*. *prausnitzii*, (E) *E*. *rectale*, (F) *S*. *thermophilus* and (G) *E*. *faecalis* are displayed. Other species have negligible abundance (≤ 0.1%). (H) The corresponding maximum community growth rate is displayed. The three sets of curves represent three different nutrient conditions in which after absorption by the host, 1% (blue), 3% (red) or 5% (yellow) of the carbohydrate in the diet is available to the gut microbiota. All values shown are minimum required values calculated by FVA.
(TIF)

S5 Fig Simulation of the gut microbiota model subject to the estimated average American diet at various levels of carbohydrate available to the gut microbiota performed using joint FBA. (A) Relative abundance, (B) fiber uptake rate and (C) fiber-derived substrate (FDS) export by *B*. *thetaiotaomicron* are displayed. Relative abundances of (D) *F*. *prausnitzii*, (E) *E*. *rectale*, (F) *S*. *thermophilus* and (G) *E*. *faecalis* are displayed. Other species have negligible abundance (≤ 0.1%). (H) The corresponding maximum community growth rate is displayed. The three sets of curves represent three different nutrient conditions in which after absorption by the host, 1% (blue), 3% (red) or 5% (yellow) of the carbohydrate in the diet is available to the gut microbiota. Note that joint FBA predicts non-zero abundances only for *E*. *rectale*, *E*. *faecalis* and *S*. *thermophilus* while *B*. *thetaiotaomicron* digests fiber and exports FDS at high rates without any growth. All values shown are minimum required values calculated by FVA. Overlapping curves are plotted using dotted lines.
(TIF)

S6 Fig Simulation of the gut microbiota model subject to the estimated average American diet at varying fiber uptake rates of *B*. *thetaiotaomicron* and *F*. *prausnitzii*. The maximum community growth rate (the black curve) and species composition (filled area) were predicted by SteadyCom at varying maximum specific fiber uptake rate (FUR) of *B*. *thetaiotaomicron* with the maximum specific FUR of *F*. *prausnitzii* fixed at 5, 10, 15, 20, 25 or 30 C-mmol gdw^-1^h^-1^ respectively.
(TIF)

S7 Fig Simulation subject to bounded abundances for minor species at various levels of carbohydrate available to the gut microbiota. Simulations subject to bounded abundances for minor species are shown when 1% (left column), 3% (middle column) or 5% (right column) carbohydrate in the diet is available to the gut microbiota after host absorption. The relative abundances of (A) *B*. *thetaiotaomicron* (B) *E*. *rectale*, (C) *F*. *prausnitzii*, (D) *E*. *faecalis*, (E) *B*. *adolescentis* at maximum community growth are displayed. (F) The maximum community growth rates, (G) aggregate fiber-derived substrate (FDS) export by *B*. *thetaiotaomicron* and (H) specific rate of FDS export by *B*. *thetaiotaomicron* are displayed. The aggregate FDS export by *B*. *thetaiotaomicron* is equal to the specific rate of FDS export by *B*. *thetaiotaomicron* multiplied by its relative abundance. Curve w0 represents the estimated average American diet. wHF1 and wHF2 represent the diets derived from w0 with 50% and 100% carbohydrate content replaced by dietary fiber, respectively. All values shown are minimum required values calculated by FVA.
(TIF)

S8 Fig Effect of varying amino acids, carbohydrate and dietary fiber content on the gut microbiota. Relative abundances of (A) *B*. *thetaiotaomicron*, (B) Clostridia (*E*. *rectale* + *F*. *prausnitzii*), (C) *E*. *rectale*, and (D) *F*. *prausnitzii*, were determined for the community for various amino-acid-to-carbohydrate ratio (AA/Carb with units of gram/gram) and fiber availability rates (g/hr). The estimated diets used in Turnbaugh *et al*., 2009 [65] and the corresponding experimental relative abundances for Bacteroidetes and Clostridia were represented by the triangles. (E) The corresponding maximum community growth rate was displayed. (F) Minimum production rates of total short-chain fatty acids (SCFA) by the community were calculated by FVA over various nutrient levels. (G) The yield of SCFA on biomass was obtained by dividing the minimum SCFA production rate by the community growth rate. The total mass of amino acids, carbohydrates and available dietary fiber was kept constant over the various combinations of dietary components. The three circles shown in each plot represent three simulated nutrient conditions: the estimated average American diet (blue), and the two diets derived from the American diet with 50% (green) and 90% (red) carbohydrate content replaced by dietary fiber. The trend that Bacteroidetes and SCFA productions generally increase with dietary fiber uptake is consistent with experimental results. See S2 Text for more details.
(TIF)

S9 Fig Distribution of the gut microbiota abundances simulated using 1000 sets of carbon uptake bounds randomly sampled from a uniform distribution for each species given the estimated average American diet. Species abundances simulated using SteadyCom (blue) and joint FBA (red) respectively are displayed. Randomly sampled constraints have a mean of total carbon uptake by each species, equal to (A) 240 C-mmol gdw^-1^h^-1^ following an exponential distribution, (B) 240 C-mmol gdw^-1^h^-1^ following a uniform distribution, (C) 120 C-mmol gdw^-1^h^-1^ following an exponential distribution, (D) 120 C-mmol gdw^-1^h^-1^ following a uniform distribution, (E) 60 C-mmol gdw^-1^h^-1^ following an exponential distribution, (F) 60 C-mmol gdw^-1^h^-1^ following a uniform distribution.
(TIF)

S10 Fig Effect of varying contents of amino acids, carbohydrate and dietary fiber on the gut microbiota constrained by a set of random uptake bounds. Relative abundances of (A) *B*. *thetaiotaomicron*, (B) *F*. *prausnitzii*, (C) *E*. *rectale*, (D) Clostridia (*E*. *rectale* + *F*. *prausnitzii*), (E) *B*. *adolescentis*, F) *E*. *coli*, (G) *K*. *pneumoniae*, (H) *S*. *thermophilus* and (I) *E*. *faecalis* found at maximum community growth using SteadyCom are displayed. Note that the maximum abundance in the z-axis in each plot is not identical for visualization purpose. The estimated diets used in Turnbaugh *et al*., 2009 [65] and the corresponding experimental relative abundances for Bacteroidetes and Clostridia were represented by the triangles. (J) Maximum community growth rates are displayed. Minimum production rates by the community were calculated by FVA for (K) acetate, (L) butyrate, (M) propionate and (N) total SCFA production over various nutrient levels. (O) The yield of SCFA on biomass was obtained by dividing the minimum SCFA production rate by the community growth rate. The total mass of amino acids, carbohydrates and available dietary fiber was kept constant over the various combinations of dietary components. The three points shown in each plot represent three nutrient conditions: the estimated average American diet (blue), and the two diets derived from the American diet with 50% (green) and 100% (red) carbohydrate content replaced by dietary fiber.
(TIF)

S11 Fig Flux variability analysis between all species abundances and SCFA productions. Flux variability was calculated at 75%, 90%, 99% and 99.9% of the maximum growth rate utilizing the SteadyCom framework and a set of random uptake bounds given the estimated average American diet as nutrients to the community.
(TIF)

S1 Table Comparison of SteadyCom and cFBA for the co-growth of three pairs of *E*. *coli* mutants. (PDF)

S2 Table Simulation details for the community of four *E*. *coli* mutants. (PDF)

S3 Table Dietary fiber and fiber-derived substrates available in the nine-species gut microbiota model. (PDF)

S4 Table Detailed estimation of the average American diet. (XLSX)

S1 Text Supplementary methods. Derivation of the community steady-state, the algorithm for solving SteadyCom and theorems regarding the convergence to the maximum community growth rate by the algorithm are presented.
(DOCX)

S2 Text Supplementary results. (PDF)

S1 Dataset The nine-species microbiota model in Matlab, SBML, TSV, and excel format, including Matlab functions for SteadyCom and FVA and example scripts. (ZIP)
