# Supplementary figures and images for: SteadyCom: Predicting microbial abundances while ensuring community stability
Source: PLoS Comput Biol. 2017 May 15;13(5):e1005539. doi: 10.1371/journal.pcbi.1005539 (PMC5448816; doi:10.1371/journal.pcbi.1005539)

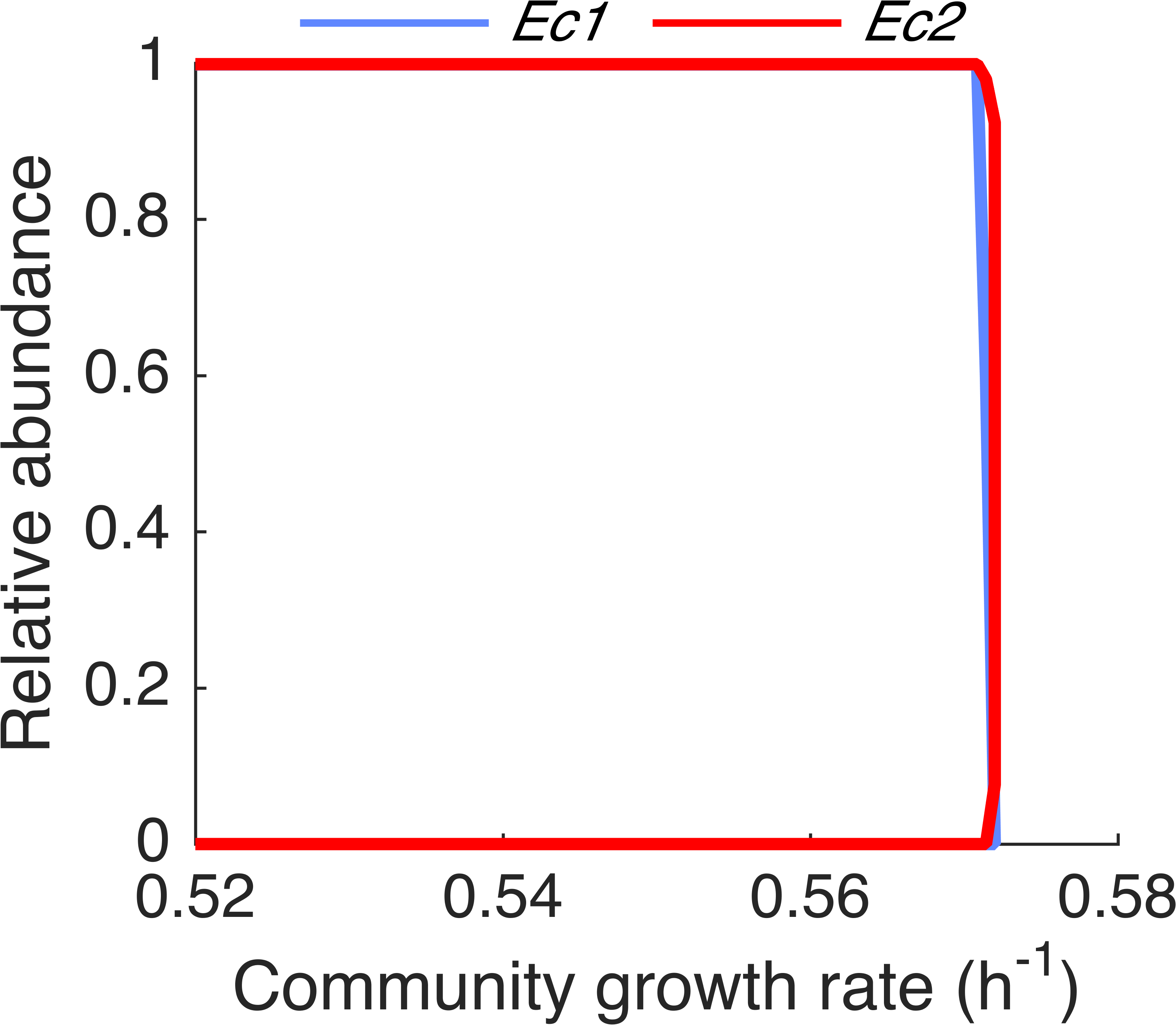

Supplement: S1 Fig — Joint FBA finds the same FVA range for Ec1 and Ec4, and for Ec2 and Ec3, respectively. None of the mutant is predicted to have non-zero abundance necessary for ≤99% maximum community growth. (TIF) [file pcbi.1005539.s001.tif]

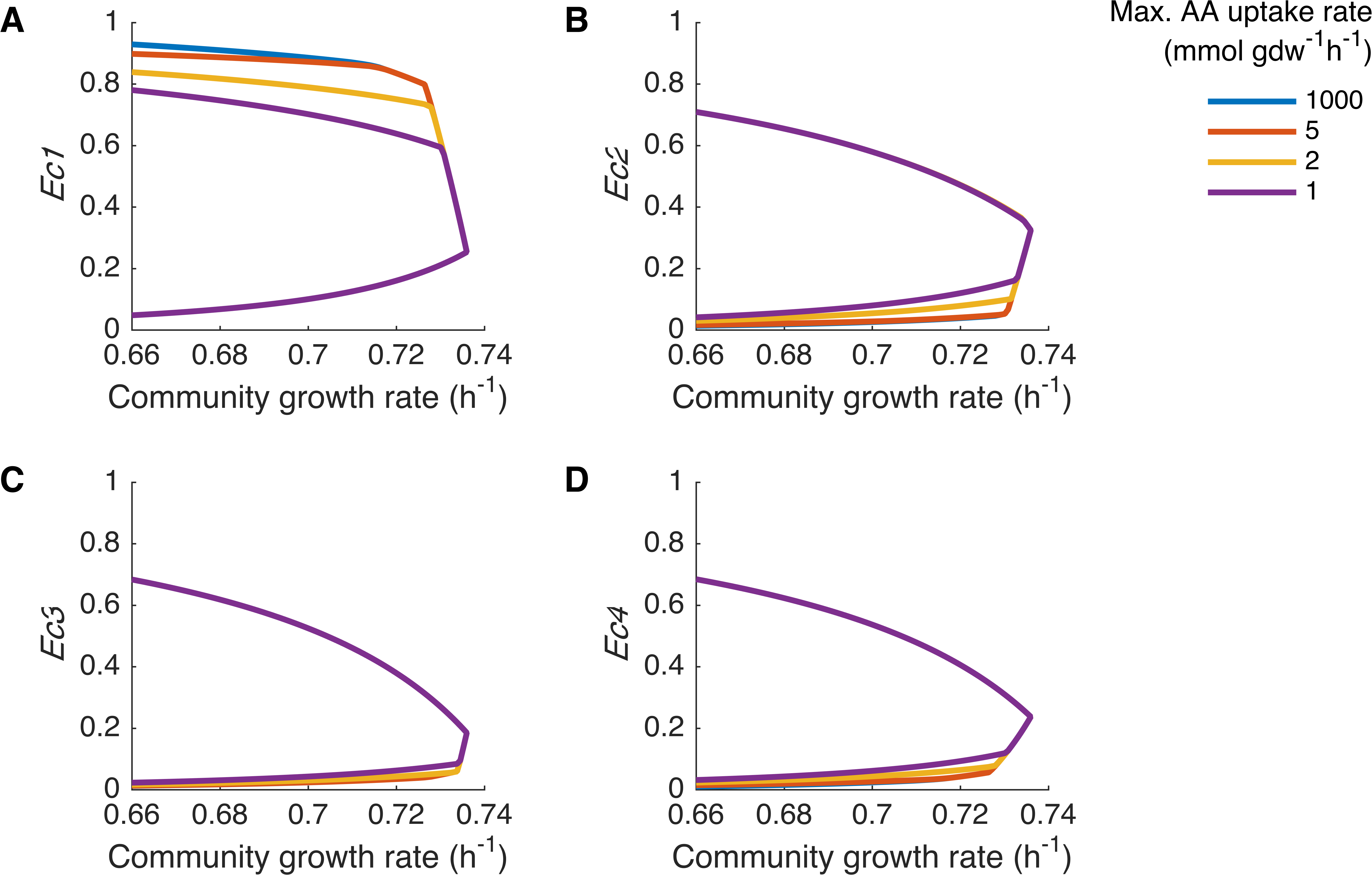

Supplement: S2 Fig — The maximum and minimum relative abundances of (A) Ec1, (B) Ec2, (C) Ec3 and (D) Ec4 are displayed for various community growth rates for various maximum amino acid uptake rates. The ranges for different maximum specific uptake rates of amino acids overlap when the growth rate is close to the maximum (≥0.732 h-1). The lower limit of Ec1 and the upper limits of Ec2, Ec3 and Ec4 remain the same regardless of the maximum uptake rate. The results shown in the main text correspond to the simulation performed at maximum specific uptake rates of amino acids equal to 1 mmol gdw-1h-1. (TIF) [file pcbi.1005539.s002.tif]

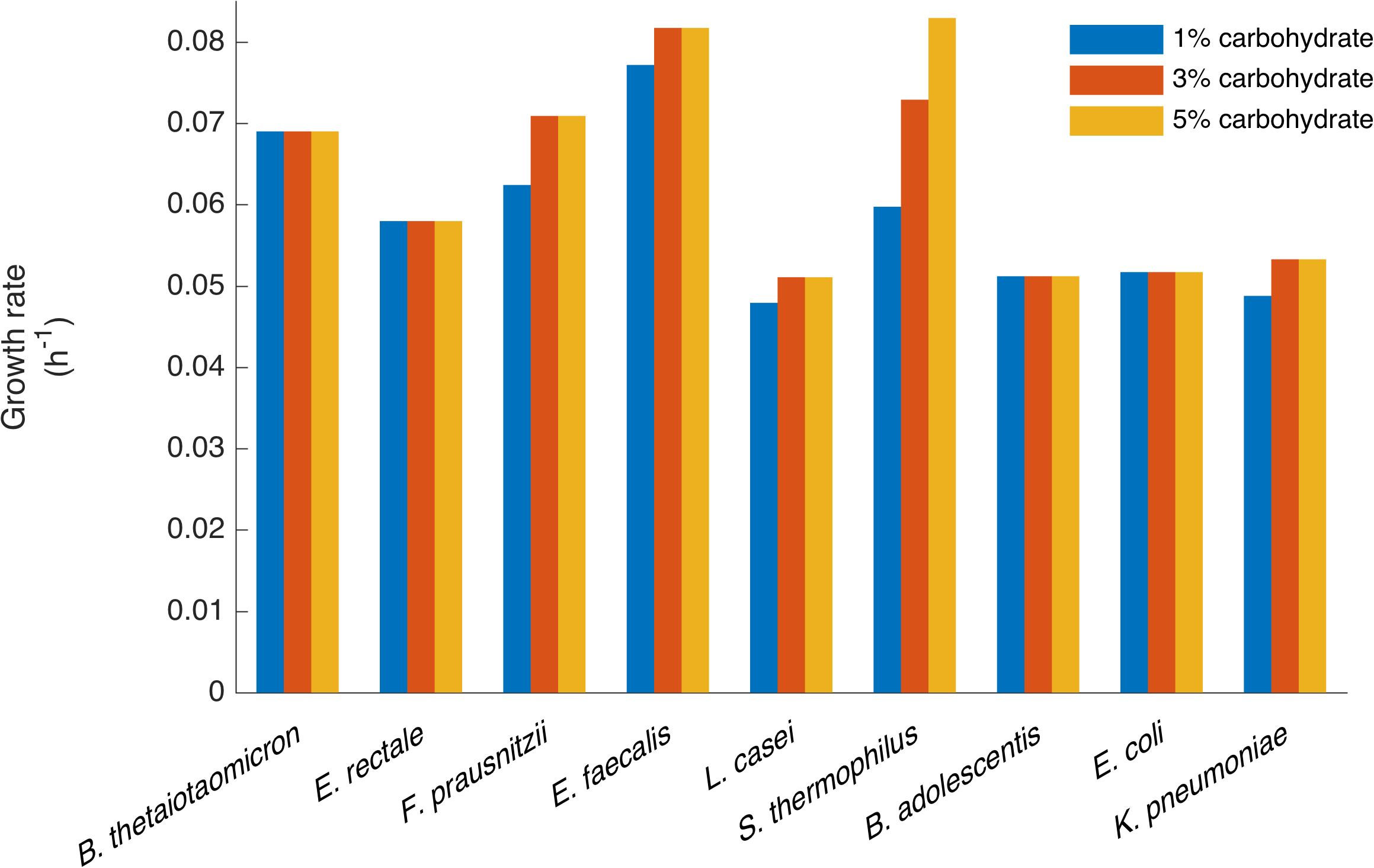

Supplement: S3 Fig — Predictions were obtained by maximizing the biomass reaction of each species individually, given the estimated average American diet with 1% (blue), 3% (red) or 5% (yellow) of carbohydrate available to the gut microbiota after absorption by the host. (TIF) [file pcbi.1005539.s003.tif]

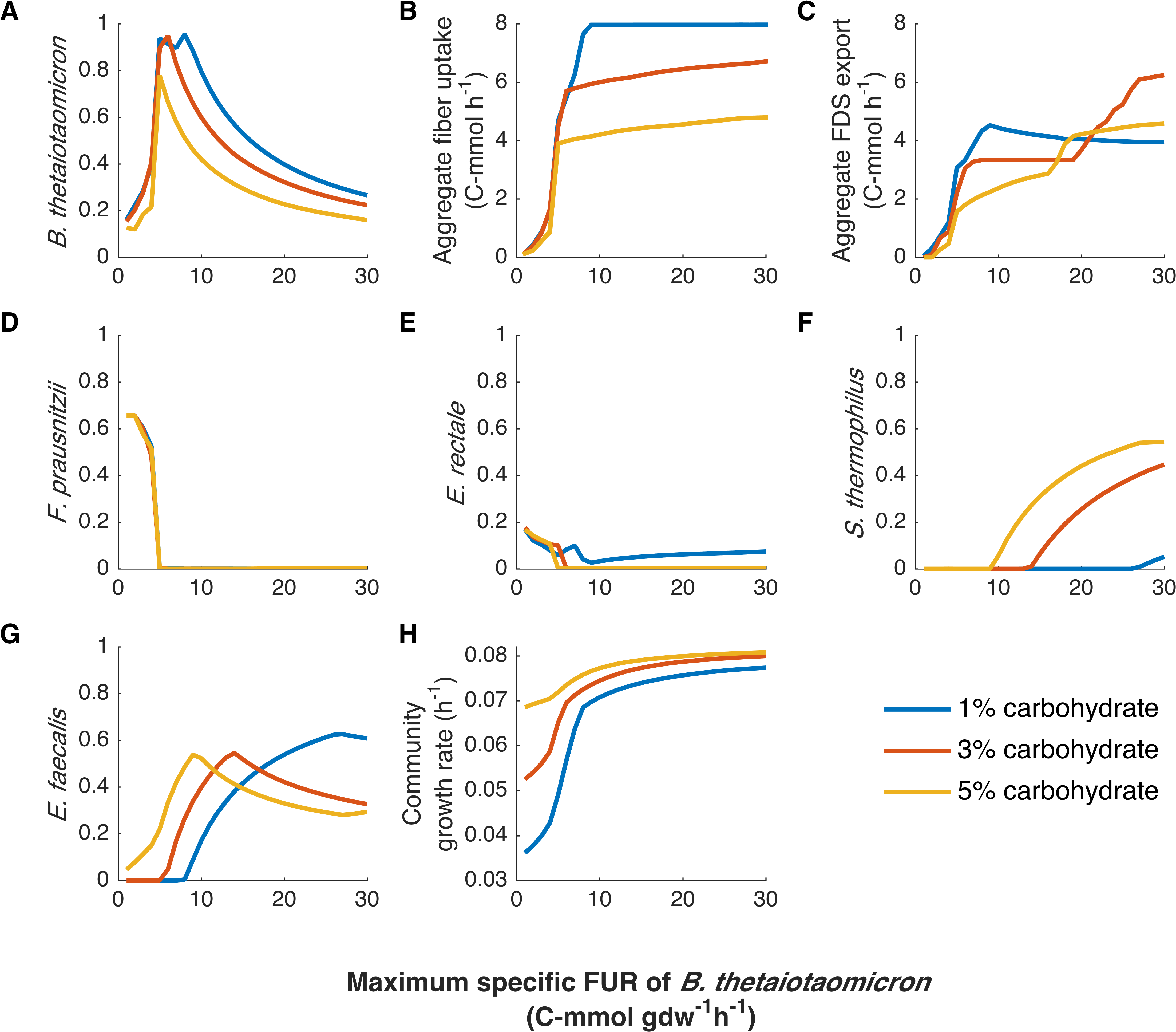

Supplement: S4 Fig — (A) Relative abundance, (B) aggregate fiber uptake and (C) aggregate fiber-derived substrate (FDS) export by B. thetaiotaomicron are displayed. Relative abundances of (D) F. prausnitzii, (E) E. rectale, (F) S. thermophilus and (G) E. faecalis are displayed. Other species have negligible abundance (≤ 0.1%). (H) The corresponding maximum community growth rate is displayed. The three sets of curves represent three different nutrient conditions in which after absorption by the host, 1% (blue), 3% (red) or 5% (yellow) of the carbohydrate in the diet is available to the gut microbiota. All values shown are minimum required values calculated by FVA. (TIF) [file pcbi.1005539.s004.tif]

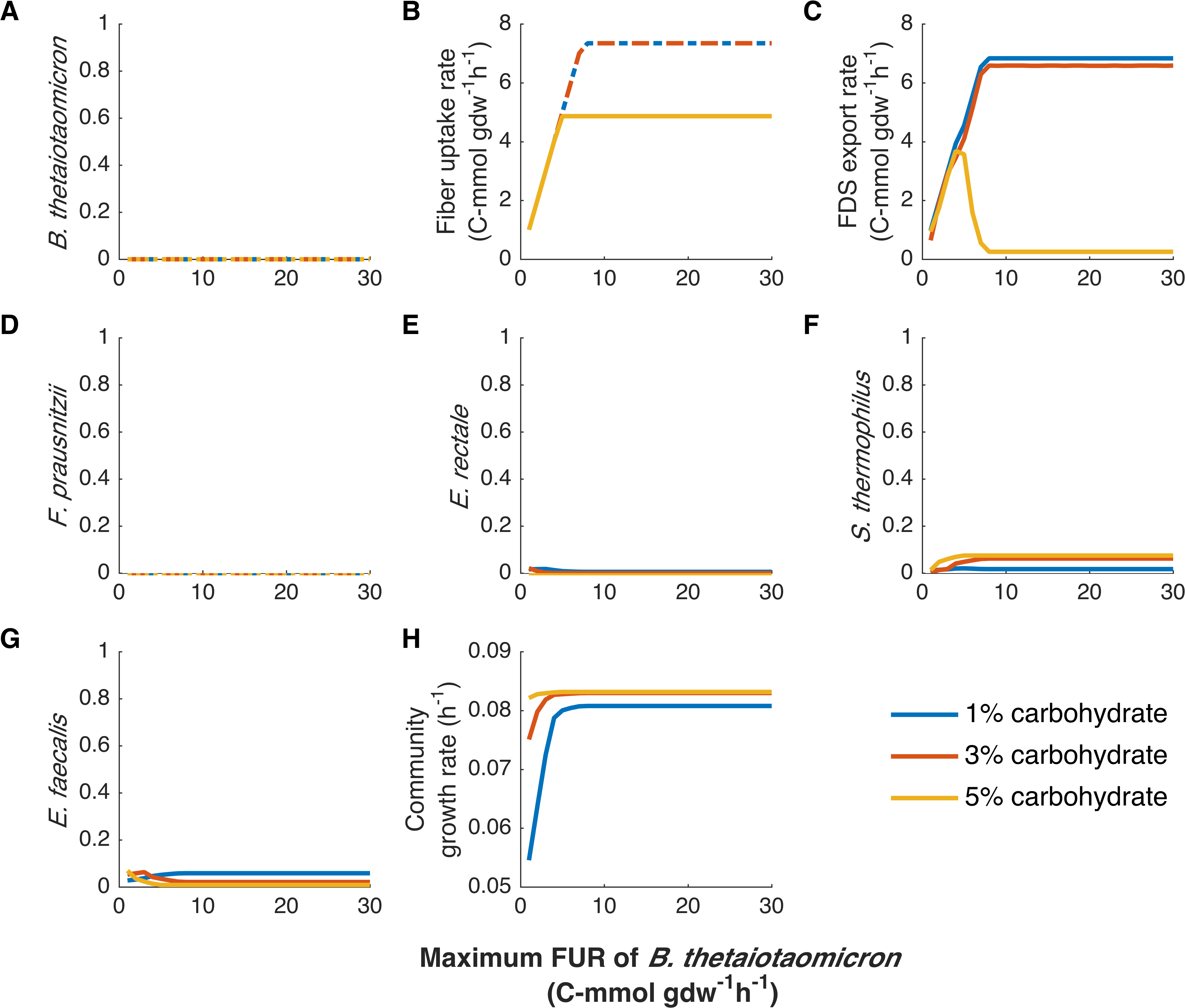

Supplement: S5 Fig — (A) Relative abundance, (B) fiber uptake rate and (C) fiber-derived substrate (FDS) export by B. thetaiotaomicron are displayed. Relative abundances of (D) F. prausnitzii, (E) E. rectale, (F) S. thermophilus and (G) E. faecalis are displayed. Other species have negligible abundance (≤ 0.1%). (H) The corresponding maximum community growth rate is displayed. The three sets of curves represent three different nutrient conditions in which after absorption by the host, 1% (blue), 3% (red) or 5% (yellow) of the carbohydrate in the diet is available to the gut microbiota. Note that joint FBA predicts non-zero abundances only for E. rectale, E. faecalis and S. thermophilus while B. thetaiotaomicron digests fiber and exports FDS at high rates without any growth. All values shown are minimum required values calculated by FVA. Overlapping curves are plotted using dotted lines. (TIF) [file pcbi.1005539.s005.tif]

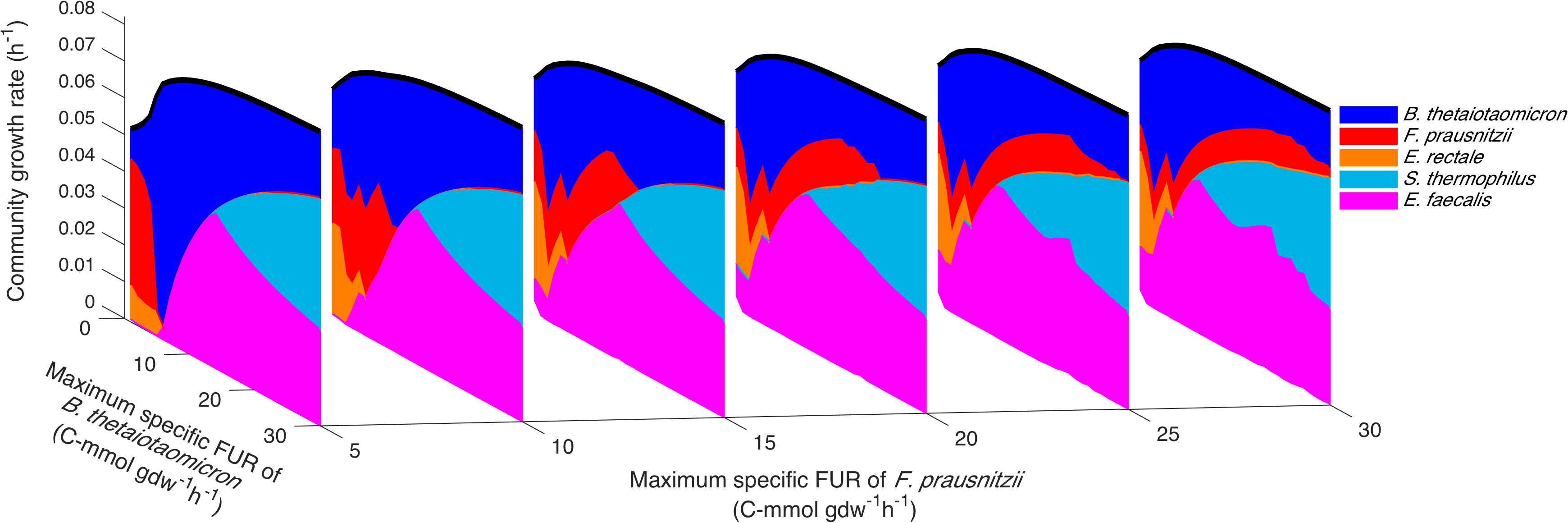

Supplement: S6 Fig — The maximum community growth rate (the black curve) and species composition (filled area) were predicted by SteadyCom at varying maximum specific fiber uptake rate (FUR) of B. thetaiotaomicron with the maximum specific FUR of F. prausnitzii fixed at 5, 10, 15, 20, 25 or 30 C-mmol gdw-1h-1 respectively. (TIF) [file pcbi.1005539.s006.tif]

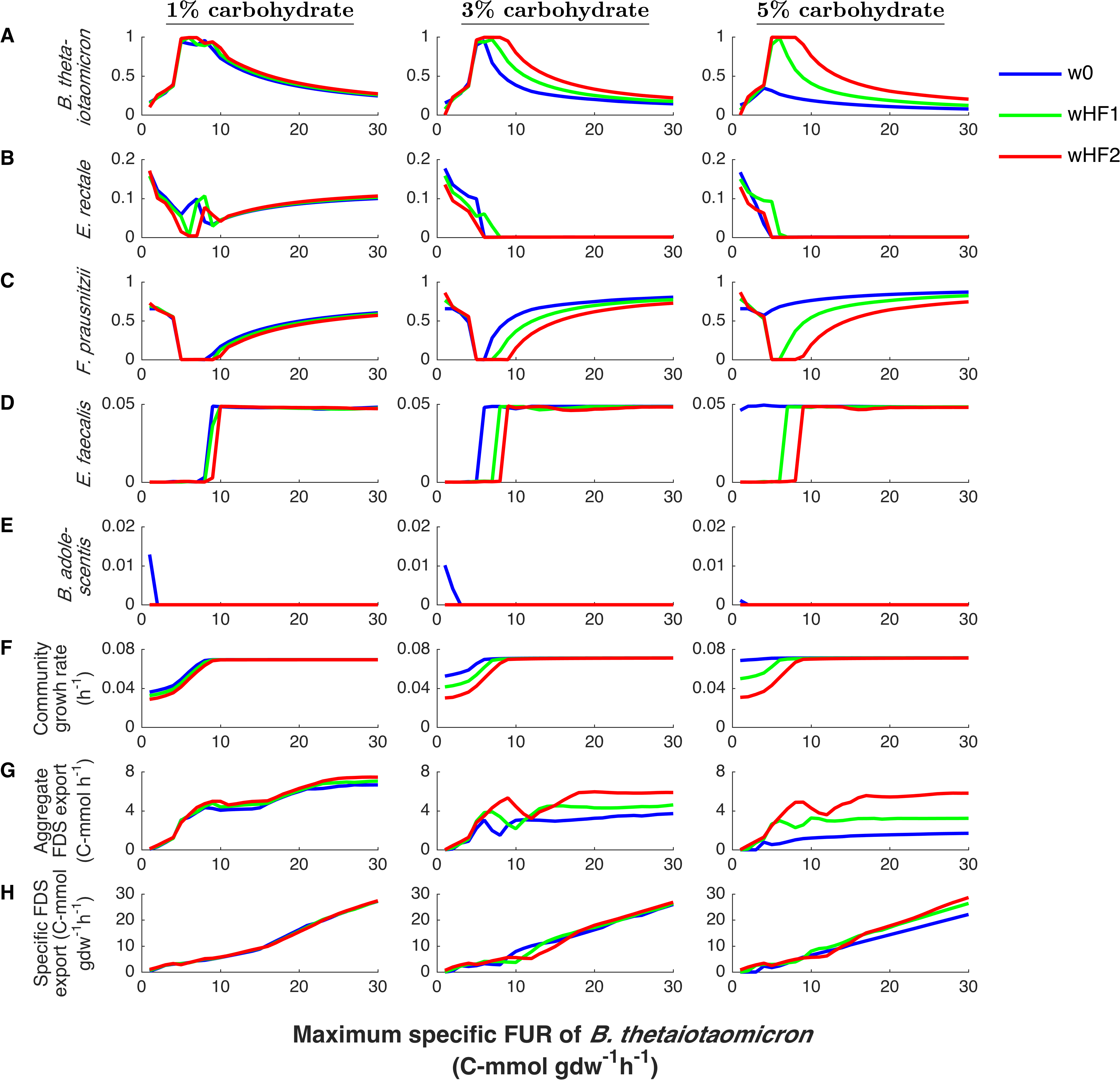

Supplement: S7 Fig — Simulations subject to bounded abundances for minor species are shown when 1% (left column), 3% (middle column) or 5% (right column) carbohydrate in the diet is available to the gut microbiota after host absorption. The relative abundances of (A) B. thetaiotaomicron (B) E. rectale, (C) F. prausnitzii, (D) E. faecalis, (E) B. adolescentis at maximum community growth are displayed. (F) The maximum community growth rates, (G) aggregate fiber-derived substrate (FDS) export by B. thetaiotaomicron and (H) specific rate of FDS export by B. thetaiotaomicron are displayed. The aggregate FDS export by B. thetaiotaomicron is equal to the specific rate of FDS export by B. thetaiotaomicron multiplied by its relative abundance. Curve w0 represents the estimated average American diet. wHF1 and wHF2 represent the diets derived from w0 with 50% and 100% carbohydrate content replaced by dietary fiber, respectively. All values shown are minimum required values calculated by FVA. (TIF) [file pcbi.1005539.s007.tif]

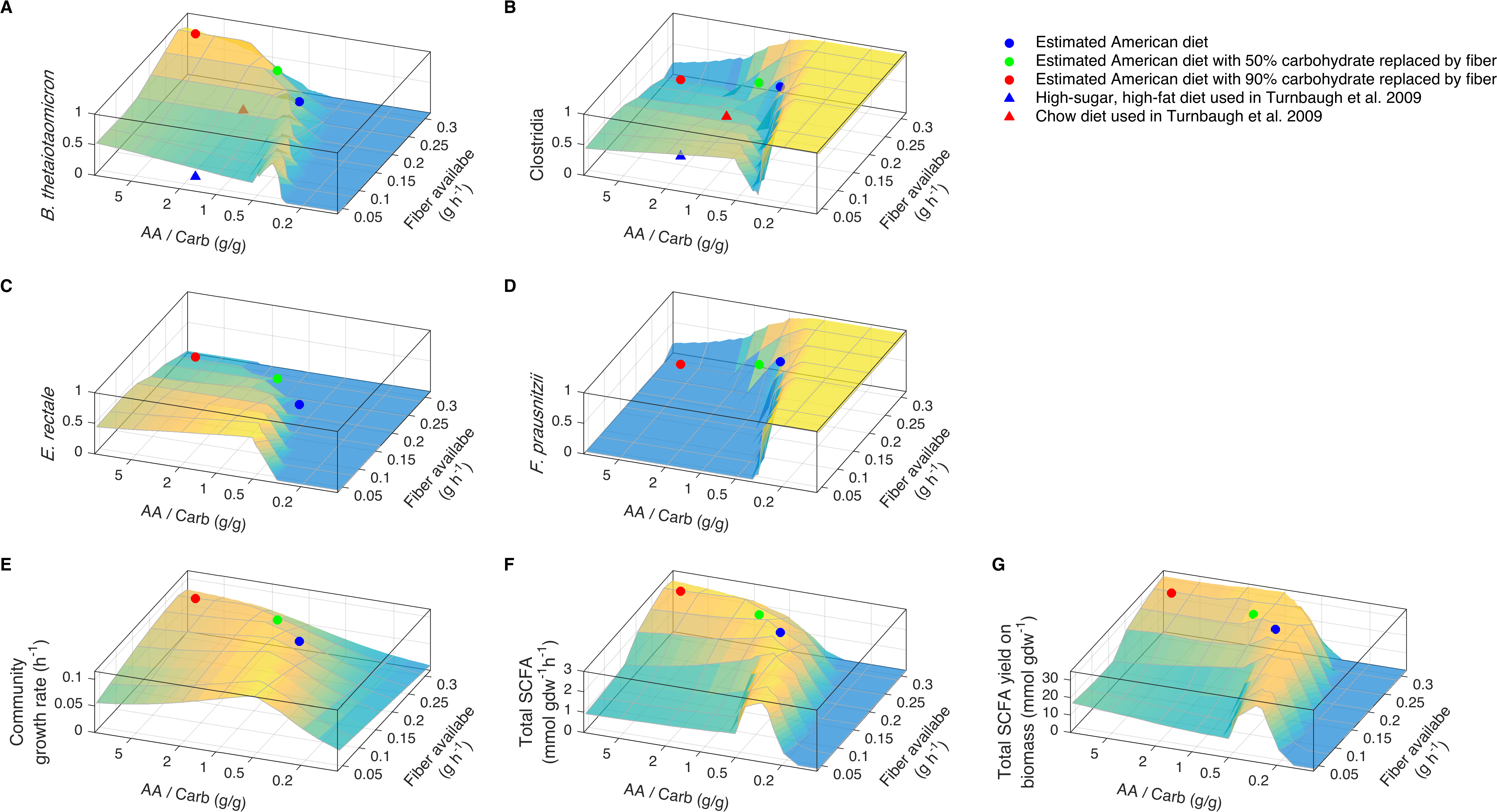

Supplement: S8 Fig — Relative abundances of (A) B. thetaiotaomicron, (B) Clostridia (E. rectale + F. prausnitzii), (C) E. rectale, and (D) F. prausnitzii, were determined for the community for various amino-acid-to-carbohydrate ratio (AA/Carb with units of gram/gram) and fiber availability rates (g/hr). The estimated diets used in Turnbaugh et al., 2009 [65] and the corresponding experimental relative abundances for Bacteroidetes and Clostridia were represented by the triangles. (E) The corresponding maximum community growth rate was displayed. (F) Minimum production rates of total short-chain fatty acids (SCFA) by the community were calculated by FVA over various nutrient levels. (G) The yield of SCFA on biomass was obtained by dividing the minimum SCFA production rate by the community growth rate. The total mass of amino acids, carbohydrates and available dietary fiber was kept constant over the various combinations of dietary components. The three circles shown in each plot represent three simulated nutrient conditions: the estimated average American diet (blue), and the two diets derived from the American diet with 50% (green) and 90% (red) carbohydrate content replaced by dietary fiber. The trend that Bacteroidetes and SCFA productions generally increase with dietary fiber uptake is consistent with experimental results. See S2 Text for more details. (TIF) [file pcbi.1005539.s008.tif]

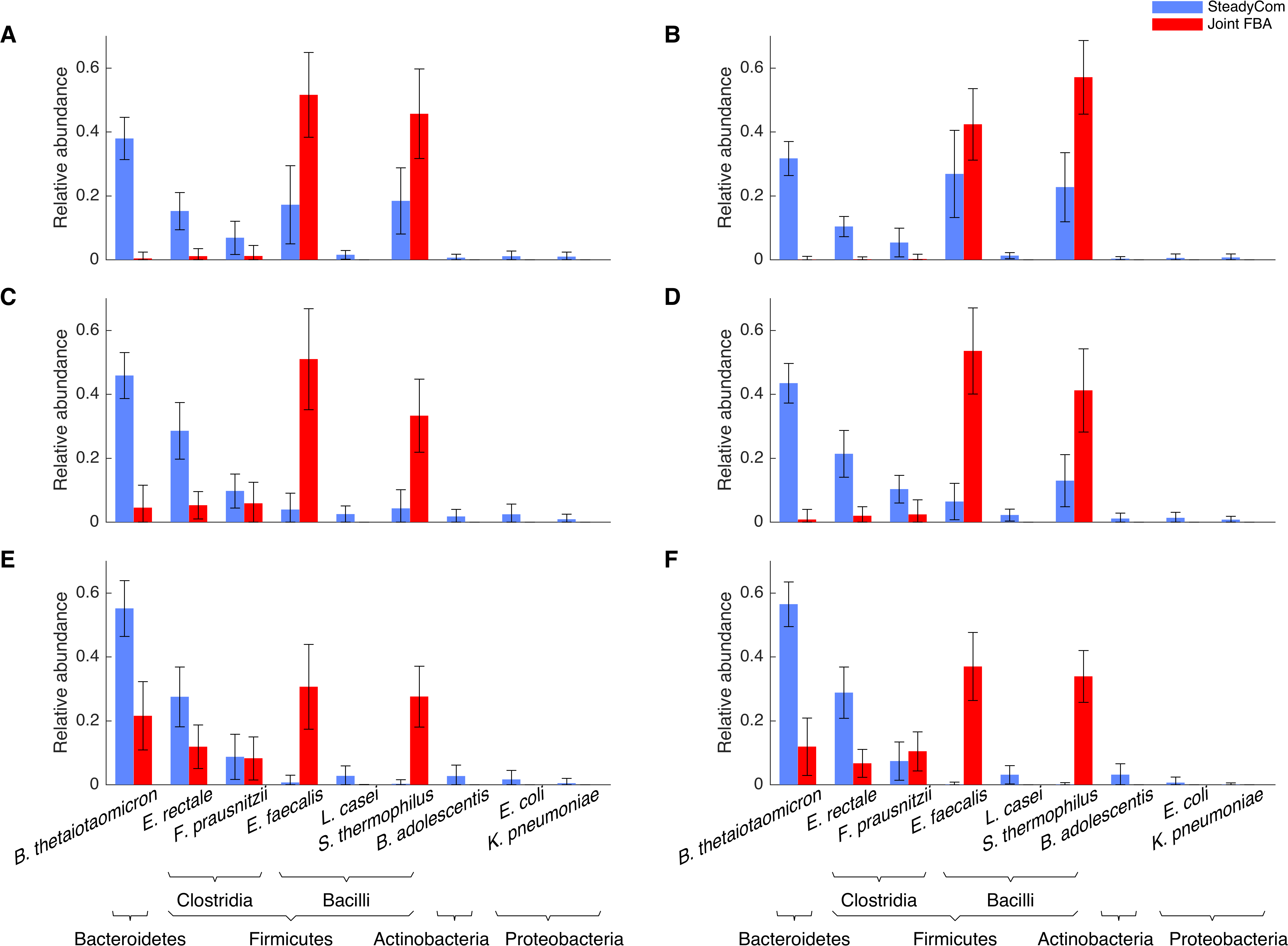

Supplement: S9 Fig — Species abundances simulated using SteadyCom (blue) and joint FBA (red) respectively are displayed. Randomly sampled constraints have a mean of total carbon uptake by each species, equal to (A) 240 C-mmol gdw-1h-1 following an exponential distribution, (B) 240 C-mmol gdw-1h-1 following a uniform distribution, (C) 120 C-mmol gdw-1h-1 following an exponential distribution, (D) 120 C-mmol gdw-1h-1 following a uniform distribution, (E) 60 C-mmol gdw-1h-1 following an exponential distribution, (F) 60 C-mmol gdw-1h-1 following a uniform distribution. (TIF) [file pcbi.1005539.s009.tif]

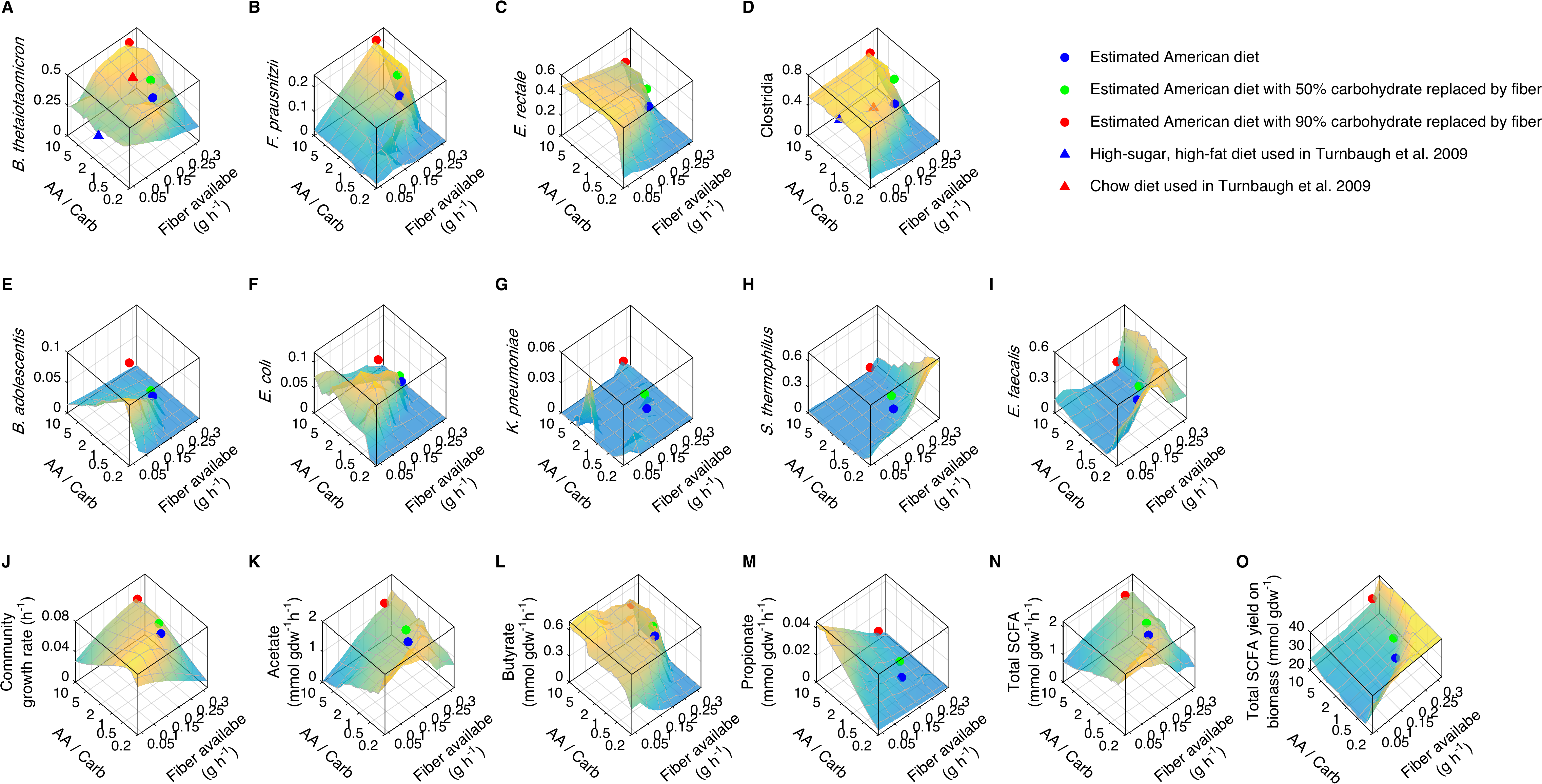

Supplement: S10 Fig — Relative abundances of (A) B. thetaiotaomicron, (B) F. prausnitzii, (C) E. rectale, (D) Clostridia (E. rectale + F. prausnitzii), (E) B. adolescentis, F) E. coli, (G) K. pneumoniae, (H) S. thermophilus and (I) E. faecalis found at maximum community growth using SteadyCom are displayed. Note that the maximum abundance in the z-axis in each plot is not identical for visualization purpose. The estimated diets used in Turnbaugh et al., 2009 [65] and the corresponding experimental relative abundances for Bacteroidetes and Clostridia were represented by the triangles. (J) Maximum community growth rates are displayed. Minimum production rates by the community were calculated by FVA for (K) acetate, (L) butyrate, (M) propionate and (N) total SCFA production over various nutrient levels. (O) The yield of SCFA on biomass was obtained by dividing the minimum SCFA production rate by the community growth rate. The total mass of amino acids, carbohydrates and available dietary fiber was kept constant over the various combinations of dietary components. The three points shown in each plot represent three nutrient conditions: the estimated average American diet (blue), and the two diets derived from the American diet with 50% (green) and 100% (red) carbohydrate content replaced by dietary fiber. (TIF) [file pcbi.1005539.s010.tif]

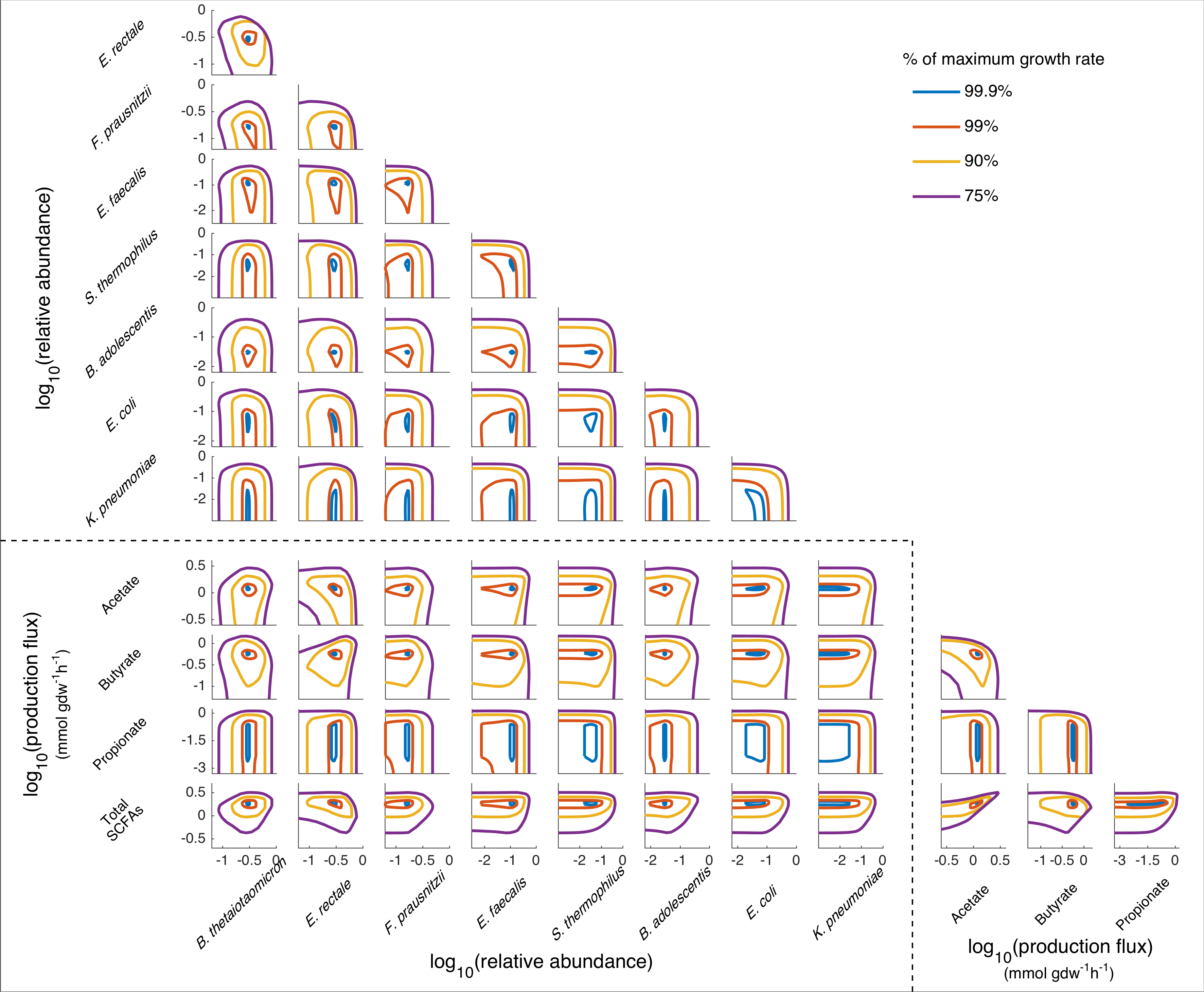

Supplement: S11 Fig — Flux variability was calculated at 75%, 90%, 99% and 99.9% of the maximum growth rate utilizing the SteadyCom framework and a set of random uptake bounds given the estimated average American diet as nutrients to the community. (TIF) [file pcbi.1005539.s011.tif]

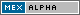

Supplement: S1 Dataset — (ZIP) [file pcbi.1005539.s018.zip › S1 Dataset/SteadyCom/doc/alpha.png]

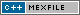

Supplement: S1 Dataset — (ZIP) [file pcbi.1005539.s018.zip › S1 Dataset/SteadyCom/doc/c++.png]

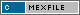

Supplement: S1 Dataset — (ZIP) [file pcbi.1005539.s018.zip › S1 Dataset/SteadyCom/doc/c.png]

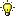

Supplement: S1 Dataset — (ZIP) [file pcbi.1005539.s018.zip › S1 Dataset/SteadyCom/doc/demoicon.gif]

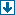

Supplement: S1 Dataset — (ZIP) [file pcbi.1005539.s018.zip › S1 Dataset/SteadyCom/doc/down.png]

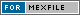

Supplement: S1 Dataset — (ZIP) [file pcbi.1005539.s018.zip › S1 Dataset/SteadyCom/doc/fortran.png]

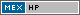

Supplement: S1 Dataset — (ZIP) [file pcbi.1005539.s018.zip › S1 Dataset/SteadyCom/doc/hp.png]

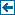

Supplement: S1 Dataset — (ZIP) [file pcbi.1005539.s018.zip › S1 Dataset/SteadyCom/doc/left.png]

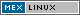

Supplement: S1 Dataset — (ZIP) [file pcbi.1005539.s018.zip › S1 Dataset/SteadyCom/doc/linux.png]

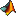

Supplement: S1 Dataset — (ZIP) [file pcbi.1005539.s018.zip › S1 Dataset/SteadyCom/doc/matlabicon.gif]

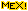

Supplement: S1 Dataset — (ZIP) [file pcbi.1005539.s018.zip › S1 Dataset/SteadyCom/doc/mex.png]

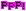

Supplement: S1 Dataset — (ZIP) [file pcbi.1005539.s018.zip › S1 Dataset/SteadyCom/doc/pcode.png]

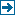

Supplement: S1 Dataset — (ZIP) [file pcbi.1005539.s018.zip › S1 Dataset/SteadyCom/doc/right.png]

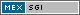

Supplement: S1 Dataset — (ZIP) [file pcbi.1005539.s018.zip › S1 Dataset/SteadyCom/doc/sgi.png]

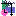

Supplement: S1 Dataset — (ZIP) [file pcbi.1005539.s018.zip › S1 Dataset/SteadyCom/doc/simulinkicon.gif]

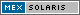

Supplement: S1 Dataset — (ZIP) [file pcbi.1005539.s018.zip › S1 Dataset/SteadyCom/doc/solaris.png]

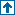

Supplement: S1 Dataset — (ZIP) [file pcbi.1005539.s018.zip › S1 Dataset/SteadyCom/doc/up.png]

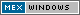

Supplement: S1 Dataset — (ZIP) [file pcbi.1005539.s018.zip › S1 Dataset/SteadyCom/doc/windows.png]
