# Supplementary material for: SteadyCom: Predicting microbial abundances while ensuring community stability
Source: PLoS Comput Biol. 2017 May 15;13(5):e1005539. doi: 10.1371/journal.pcbi.1005539 (PMC5448816; doi:10.1371/journal.pcbi.1005539)
Supplement: S1 Table — (PDF) [file pcbi.1005539.s012.pdf]

S1 Table. Comparison of SteadyCom and cFBA for the co-growth of three pairs of *E. coli* mutants

	cFBA	cFBA	SteadyCom
Step size	0.01	0.002	--
Initial guess for μ	--	--	0.01
<u>*Mutant pair: ΔargH ('ARGSL'), ΔlysA ('DAPDC')*</u>			
μ_{max}	0.736195	0.736222	0.736232
Biomass (X^1, X^2)	(0.56, 0.44)	(0.562, 0.438)	(0.562849, 0.437152)
No. of LP solved	101	501	11
Time used (sec)	12.5	57.4	3.6
<u>*Mutant pair: ΔlysA ('DAPDC'), ΔtrpC ('TRPS3')*</u>			
μ_{max}	0.736588	0.736588	0.736588
Biomass ratio (X^1, X^2)	(0.22, 0.78)	(0.22, 0.78)	(0.221090, 0.778911)
No. of LP solved	101	501	11
Time used (sec)	11.5	57.5	1.7
<u>*Mutant pair: ΔmetA ('HSST'), ΔilvE ('ILETA')*</u>			
μ_{max}	0.736267	0.736389	0.736402
Biomass ratio (X^1, X^2)	(0.61, 0.39)	(0.612, 0.388)	(0.611869, 0.388132)
No. of LP solved	101	501	11
Time used (sec)	9.9	48.2	1.4

Names in the parentheses after the deleted genes are the corresponding reactions shut down in the *E. coli* iAF1260 genome-scale model (Feist AM, Henry CS, Reed JL, Krummenacker M, et al. 2007. *Mol. Syst. Biol.* 3: 121). The algorithm provided in cFBA repeatedly changes the relative abundance profile and solves an LP problem given the abundance profile. Step size in cFBA refers to the change in organism's abundance in each step.
