# Supplementary material for: SteadyCom: Predicting microbial abundances while ensuring community stability
Source: PLoS Comput Biol. 2017 May 15;13(5):e1005539. doi: 10.1371/journal.pcbi.1005539 (PMC5448816; doi:10.1371/journal.pcbi.1005539)
Supplement: S2 Table — (PDF) [file pcbi.1005539.s013.pdf]

S2 Table. Simulation details for the community of four *E. coli* mutants.

	Ec1	Ec2	Ec3	Ec4	Community
Genotype	Δ*lysA*<br>Δ*metA*<br>Δ*yddG*	Δ*argH*<br>Δ*pheA*<br>Δ*yjeH*	Δ*argH*<br>Δ*lysO*<br>Δ*pheA*	Δ*argO*<br>Δ*lysA*<br>Δ*metA*	--
Inactivated reactions	'DAPDC', 'HSST', 'PHEt2rpp'	'ARGSL', 'PPNDH', 'METt3pp'	'ARGSL', 'LYSt3pp', 'PPNDH'	'ARGt3pp', 'DAPDC', 'HSST'	--
Maximum specific uptake rate (mmol gdw^{-1}h^{-1}) / community uptake rate (mmol h^{-1})					
Glucose	8	8	8	8	8
Oxygen	18.5	18.5	18.5	18.5	18.5
Arginine	0	1	1	0	0
Lysine	1	0	0	1	0
Methionine	1	0	0	1	0
Phenylalanine	0	1	1	0	0

The glucose uptake rate and oxygen uptake are the default values in the original *E. coli* model. 'METt3pp' is a reaction added to the model for methionine transport by proton symport. The amino acid uptake rates were set at 1 mmol gdw^{-1}h^{-1} for the amino acids that a mutant cannot synthesize and were set at 0 for the amino acids that a mutant can synthesize. Amino acid uptake rates from 1 to 1000 mmol gdw^{-1}h^{-1} were tested and the results were qualitatively similar (see S2 Figure).
