# Supplementary material for: SteadyCom: Predicting microbial abundances while ensuring community stability
Source: PLoS Comput Biol. 2017 May 15;13(5):e1005539. doi: 10.1371/journal.pcbi.1005539 (PMC5448816; doi:10.1371/journal.pcbi.1005539)
Supplement: S3 Table — (PDF) [file pcbi.1005539.s014.pdf]

S3 Table. Dietary fiber and fiber-derived substrates available in the nine-species gut microbiota model

**Dietary fiber**	**metID**	**Description** (Heinken and Thiele, 2015)	**Fiber-derived substrate**
alpha-mannan	amannan140[u]	alpha-mannan, yeast, has a mw of about 25000 (pmid: 5461381), equals ~ 140 mannose units.	D-mannose
pectin	pect[u]	pectin	D-galacturonate, methanol, 4-deoxy-L-threo-5-hexosulose uronate
pectic galactan	pecticgal[u]	pectic galactan (potato), gal: arab-l: rmn: galur = 78: 9: 4: 9 (www.megazyme.com). assumed to consist of 100 sugars per macromolecule.	D-galactose, L-arabinose, L-rhamnose, D-galacturonate, methanol, acetate
arabinan	arabinan101[u]	arabinan, polymer of 1,5-a-l-linked arabinofuranose units substituted by 1,3 or 1,2 linked branches, mw ~ 15000 da (ref: www.megazyme.com). contains arabinose: galactose: rhamnose: galacturonic acid = 97: 0.4: 0.1: 2.1. -> 97 arab-l, 1 gal, 1 rmn, 2 galur	D-galactose, L-arabinose, L-rhamnose, D-galacturonate
larch arabinogalactan	arabinogal[u]	larch arabinogalactan, gal/ara 6:1, small amount of glcur, mw ~ 116000	D-galactose, L-arabinose, D-glucuronate
homogalacturonan	homogal[u]	homogalacturonan, 100 galacturonate residues. assumed to be methylated/ acetylated in every 5th residue. citrus hg consists of 72-100 galacturonate residues and has a methylesterification degree of 22,9% (pmid: 11278866).	D-galacturonate, methanol, acetate
chicory inulin	inulin[u]	chicory inulin, 30 monosaccharide units (29 fru, 1 glc) on average	D-fructose, D-glucose
kestose	kesto[u]	kestose (2 fru, 1 glc inulin-type fructo-oligosaccharide)	D-fructose, D-glucose
kestopentaose	kestopt[u]	kestopentaose (4 fru, 1 glc inulin-type fructo-oligosaccharide)	D-fructose, D-glucose
kestotetraose	kestottr[u]	kestotetraose (3 fru, 1 glc inulin-type fructo-oligosaccharide)	D-fructose, D-glucose
potato rhamnogalacturonan I	rhamnogalurI[u]	potato rhamnogalacturonan i, mw assumed ~200000, 62 galur, 20 rmn, 12 gal, 3 arab-l, 1 xyl-d (www.megazyme.com, pmid: 16661590)	D-galactose, L-arabinose, L-rhamnose, D-galacturonate, D-xylose
wine rhamnogalacturonan II	rhamnogalurII[u]	wine rhamnogalacturonan ii, mw ~ 5-10 kda, 37% galur, 16% rmn, 3% fuc-l, 11% arab-l, 6% gal, 5% glcur, 4% 2omfuc, 3% 2omxyl, 6% apio-d, 2% acera, 4% kdo, 3% 3ddlhept, 53 sugars in total (http://www.ccrc.uga.edu/~mao/rg2/intro.htm, doi:10.1155/2011/964521)	D-galactose, L-arabinose, L-fucose, L-rhamnose, D-galacturonate, D-glucuronate, 3-Deoxy-D-manno-2-octulosonate<br><br><u>Rare sugar in rhamnogalacturonan II:</u><br>2-o-methylfucose, 2-O-methylxylose, 3-deoxy-d-lyxo-heptulosaric acid, Aceric acid (3-C-carboxy-5-deoxy-l-xylose), D-Apiose

The description for the dietary fiber is from the gut community model published by Heinken A, Thiele I. 2015. *Appl. Environ. Microbiol.* 81: 4049–61.
