# Supplementary material for: SteadyCom: Predicting microbial abundances while ensuring community stability
Source: PLoS Comput Biol. 2017 May 15;13(5):e1005539. doi: 10.1371/journal.pcbi.1005539 (PMC5448816; doi:10.1371/journal.pcbi.1005539)
Supplement: S1 Text — Derivation of the community steady-state, the algorithm for solving SteadyCom and theorems regarding the convergence to the maximum community growth rate by the algorithm are presented. (DOCX) [file pcbi.1005539.s016.docx]

**S1 Text**

**SUPPLEMENTARY METHODS**

Table of Contents

Page

Notations 2

Community steady-state 4

Algorithm for solving SteadyCom 6

Conditions for *f*(*μ*) to be decreasing in *μ* 8

Proof of theorems and corollaries 12

References 19

Notations

Sets defined for the formulation:

**K** the set of all organisms in the community

**I** the set of all metabolites

$\mathbf{I}^{k}$ the set of metabolites specific to organism *k*

$\mathbf{I}^{com}$ the set of community metabolites, not belonging to any organism

$\mathbf{J}^{k}$ the set of reactions specific to organism *k*

Sets defined in the proof of the theorems:

$\mathbf{I}_{ex}^{k}$ the set of extracellular metabolites specific to organism *k*

**J** the set of all reactions

$\mathbf{J}^{0}$ the set of all community exchange reactions

$\mathbf{J}_{irr}$ the set of all irreversible reactions

$\mathbf{J}^{req}$ the set of ‘required’ reactions

**EM** the index set for all elementary modes

$\mathbf{E}\mathbf{M}^{gr}$ the index set for elementary modes with non-zero flux in the biomass reaction.

$\mathbf{E}\mathbf{M}^{gr,req}$ the index set for elementary modes that couple any required reactions in $\mathbf{J}^{req}$ with any biomass reactions

**PE**(**v**) the index set for reactions in flux distribution **v** with positive fluxes, $\left\{ j\in\mathbf{J |}v_{j}>0 \right\}$

**NE**(**v**) the index set for reactions in flux distribution **v** with negative fluxes, $\left\{ j\in\mathbf{J |}v_{j}<0 \right\}$

Variables defined for the formulation:

$X^{k}$ biomass of organism *k*

$X_{0}$ total community biomass

*μ* community growth rate

$S_{ij}^{k}$ stoichiometry of metabolite *i* in reaction *j* of organism *k*.

$V_{j}^{k}$ aggregate flux for reaction *j* of organism *k*

$V_{biomass}^{k}$ aggregate flux for the biomass reaction of organism *k*

${LB}_{j}^{k}$ lower bound for the specific rate of reaction *j* of organism *k*

${UB}_{j}^{k}$ upper bound for the specific rate of reaction *j* of organism *k*

$u_{i}^{c}$ community uptake rate of community metabolite *i*

$e_{i}^{c}$ community export rate of community metabolite *i*

Quantities, vectors and matricies defined in the proof of the theorems:

$\alpha_{p}$ weight for elementary mode *p* in a decomposition of a flux distribution

$m^{k}$ number of metabolites of organism *k*

$m_{in}^{k}$ number of intracellular metabolites of organism *k*

$m_{ex}^{k}$ number of extracellular metabolites of organism *k*

$m^{0}$ number of community metabolites

$n^{k}$ number of reactions of organism *k*

$n_{in}^{k}$ number of internal (non-exchange) reactions of organism *k*

$N_{E}$ number chemical elements

$V_{i}^{0}$ community exchange flux for community metabolite *i*

$V_{ATPM}^{k}$ aggregate flux for the non-growth-associated ATP maintenance of organism *k*

$e_{j,p}^{k}$ flux of reaction *j* of organism *k* in elementary mode *p*

$\mathbf{X}$ biomass vector containing the biomass for each organism

**v** flux distribution

**V** aggregate flux distribution

**V***^k^* aggregate flux distribution for organism *k*

**V***^0^* flux distribution for the community exchange flux

$\mathbf{V}_{biomass}^{0}$ flux vector containing the aggregate biomass export flux of each organism

$\mathbf{V}_{mets}^{0}$ flux vector for the community exchange reactions other than biomass export

**e** elementary mode

$\mathbf{S}^{k}$ stoichiometric matrix for organism *k*

$\mathbf{Id}_{m}$ *m*-by-*m* identity matrix

$\mathbf{U}^{k}$ matrix connecting extracellular metabolites of organism *k* to community metabolites

**E** elemental matrix of all metabolites

**E***^k^* elemental matrix of metabolites of organism *k*

**E***^0^* elemental matrix of community metabolites

$\mathbf{E}_{biomass}^{0}$ elemental matrix of the biomass of each organism

$\mathbf{E}_{mets}^{0}$ elemental matrix of community metabolites other than biomasses

$\mathbf{w}_{E}$ vector of molecular weights of all elements in **E**

Community steady-state

The biomass for organism *k* is defined as *X^k^* (in gdw)*.* The total community biomass *X* is thus given by:

$$\sum_{k\in\mathbf{K}} X^{k}=X_{0}$$

The relative abundance of organism *k* is equal to *X^k^ / X*. Assume that the rate of change in *X^k^* depends only on the growth rate *μ^k^* of organism *k* and the dilution rate *D^k^*:

$$\frac{dX^{k}}{dt}=\left( \mu^{k}-D^{k} \right)X^{k}, \forall k\in\mathbf{K}$$

The time derivative of the relative abundance ${X^{k}}/X$ of organism *k* is given by:

$$\frac{d}{dt}\left( \frac{X^{k}}{X_{0}} \right)=\frac{X^{k}}{X_{0}}\left( \left( \mu^{k}-D^{k} \right)-\sum_{l\in\mathbf{K}} \left( \mu^{l}-D^{l} \right)\frac{X^{l}}{X_{0}} \right)$$

If the dilution rates are identical for all organisms, i.e. $D^{k}=D, \forall k\in\boldsymbol{K}$, then the above equation is reduced to the famous replicator equation widely used in evolutionary game theory [1]. A necessary and sufficient condition for community steady-state in which the relative abundances of all organisms are steady over time is given by:

$\mu^{k}-D^{k}=C, \forall k\in\mathbf{K}$ (1)

where *C* is a constant. This means that the difference between the growth rate and the dilution rate is constant for all organisms. To prove this, assume the condition is not satisfied. Let $p,q\in\mathbf{K}$ be the two organisms with the largest and smallest differences between the growth rate and dilution rate respectively. We have

$$\mu^{p}-D^{p}=\max_{k\in\mathbf{K}} \left\{ \mu^{k}-D^{k} | X^{k}>0 \right\}>\min_{k\in\mathbf{K}} \left\{ \mu^{k}-D^{k} | X^{k}>0 \right\}=\mu^{q}-D^{q} .$$

The abundance of organism *p* will thus continue to increase since

$$\frac{X^{p}}{X}\left( \left( \mu^{p}-D^{p} \right)-\sum_{l\in\mathbf{K}} \left( \mu^{l}-D^{l} \right)\frac{X^{l}}{X} \right)$$

$$=\frac{X^{p}}{X}\left( \sum_{l\in\mathbf{K}} \left( \left( \mu^{p}-D^{p} \right)-\left( \mu^{l}-D^{l} \right) \right)\frac{X^{l}}{X} \right)$$

$$\geq\frac{X^{p}}{X}\left( \left( \mu^{p}-D^{p} \right)-\left( \mu^{q}-D^{q} \right) \right)\frac{X^{q}}{X}>0.$$

In a similar fashion, the abundance of organism *q* will continue to decrease. Therefore the steady-state community composition ensured by imposing eq. (1). When the dilution rates are identical for all organisms as we have assumed in this study, the condition of identical growth rate, which is also the steady-state condition of the replicator equation, follows:

$\mu^{k}=\mu\forall k\in\mathbf{K}$ (2)

where *μ* is the community growth rate. This condition does not depend on the dilution rate as long as it is identical for all species. This can be assumed in a well-mixed chemostat culture performed in the laboratory. When *D* is non-zero, *μ* must be larger than *D* otherwise the community will be washed away. However, in general, for example on biomaterial surfaces, *D* is not identical for all organisms as microorganisms can have different adhesiveness depending on their hydrophobicity, surface charge and the properties of the surfaces [2]. For the gut microbiota model analyzed in this study, identical growth rates is a reasonable approximation in the absence of organism-dependent dilution rates as experimental results showed that fecal and large intestine microbiota are quite similar [3]. In practice, microbial communities in nature do not remain steady at every time point. However, the steady-state approximates the average state of a microbial community over time. The time-averaged microbial community must satisfy the steady-state condition to ensure co-existence of microbes and stability. In particular for the human gut microbiota, experimental studies have suggested the existence of stable steady-states which the gut microbiome should stay near [4–7]. A number of seminal modeling studies also assumed the existence of steady-states in the gut microbiome and analyzed their stability using the generalized Lotka-Volterra equations [8–11].

Algorithm for solving SteadyCom

Although SteadyCom is nonlinear, once the community growth rate *μ* is fixed, the problem becomes a linear program (LP) and can be solved easily. The following variant of SteadyCom called ‘maxBM(*μ*)’ can be solved to check feasibility at a given *μ*:

$$f\left( \mu\right)=\max\sum_{k\in\mathbf{K}} X^{k}$$

subject to

$$\left[ \begin{matrix} \sum_{j\in\mathbf{J}^{k}} S_{ij}^{k}V_{j}^{k}=0 \forall i\in\mathbf{I}^{k} \\ {LB}_{j}^{k}X^{k}\leq V_{j}^{k}\leq{UB}_{j}^{k}X^{k} \forall j\in\mathbf{J}^{k} \\ V_{biomass}^{k}=X^{k}\mu\end{matrix} \right] \forall k\in\mathbf{K}$$

$$u_{i}^{c}-e_{i}^{c}+\sum_{k\in\mathbf{K}} V_{ex\left( i \right)}^{k}=0 \forall i\in\mathbf{I}^{com}$$

$X^{k}, e_{i}^{c}\geq0 \forall k\in\mathbf{K}, i\in\mathbf{I}^{com}$ (maxBM(*μ*))

The objective function *f*(*μ*) of maxBM(*μ*) is the total community biomass instead of the community growth rate in SteadyCom. The constraint on the total community biomass in SteadyCom is also discarded in maxBM(*μ*). If for a given community growth rate *μ*, problem maxBM(*μ*) yields *f*(*μ*) ≥ *X*_0_ then the growth rate *μ* is deemded feasible under SteadyCom. If *f*(*μ*) is decreasing in *μ*, a unique *μ*_max_ can be found by any non-derivative-based root-finding algorithms such as bisection method or secant method that solve maxBM(*μ*) iteratively. The following algorithm is adopted to first locate an interval where *μ*_max_ lies within, followed by a root-finding procedure:

Step 0. *μ* = *μ*_0_ (any initial guess)

Step 1. Compute maxBM(*μ*).

If $f\left( \mu\right)\geq X_{0}$,
 $\mu_{LB}\leftarrow\mu$;
 *μ* 🡨 *λμ*  where *λ* = max{ *f*(*μ*) ⁄ *X*_0_ , 1 + *ε* }.
Otherwise,
 $\mu_{UB}\leftarrow\mu$;  *μ* 🡨 *λμ*  where *λ* = max{ *f*(*μ*) ⁄ *X*_0_ , 1 − *ε* }.
Repeat until both *μ_LB_* and *μ_UB_* are found.

Step 2. Invoke a non-derivative-based root-finding algorithm to solve for

$$f\left( \mu\right)=X_{0} \mathrm{for}\mu\in\left[ \mu_{LB} , \mu_{UB} \right].$$

The parameter *ε* is a small positive number to ensure a minimum percentage change of *μ* in the next iteration (0.01 used in this study). In particular, the fzero function in Matlab^®^ ([www.mathworks.com](http://www.mathworks.com)) was used in this study in Step 2. All the calculations were performed using Matlab. LPs were solved using CPLEX. The total number of LP to be solved was usually less than 10 for an accuracy of 10^-6^ for *μ* in our study. It is important to note that this algorithmic procedure relies on a unique mapping *f*(*μ*) at different *μ*. In SI Methods, two theorems regarding the conditions for *f*(*μ*) to be decreasing are stated with proof and discussed in detail. If additional constraints bounding the total biomass exist, then *f*(*μ*) is possibly fixed and the algorithmic procedure needs to be modified. The bisection method can however always guarantee convergence after only a handful of steps (e.g. ≤ 21 iterations for an accuracy of 10^-6^ assuming that *μ* lies in the interval [0, 2]).

Conditions for *f*(*μ*) to be decreasing in *μ*

The algorithm presented assumes the maximum biomass *f*(*μ*) in the system at a given growth rate *μ* is decreasing in *μ*. Throughout the computation for the community of the four *E. coli* mutants as well as the ten-species microbiota model, *f*(*μ*) was found to be decreasing in *μ* (Figure ST1). This is also intuitively correct because the higher the growth rate, the more the substrate required for the biomass reproduced by each unit of biomass in a unit of time. Given a constant amount of substrate available in the system (constant uptake bounds for community metabolites), the total biomass able to grow at rate *μ*, *f*(*μ*), should decrease if *μ* increases. This is analogous to the amount of biomass sustainable in a chemostat culture, which decreases to zero as the dilution rate (equal to the growth rate at steady-state) increases to the maximum growth rate, above which cells are washed out of the system.


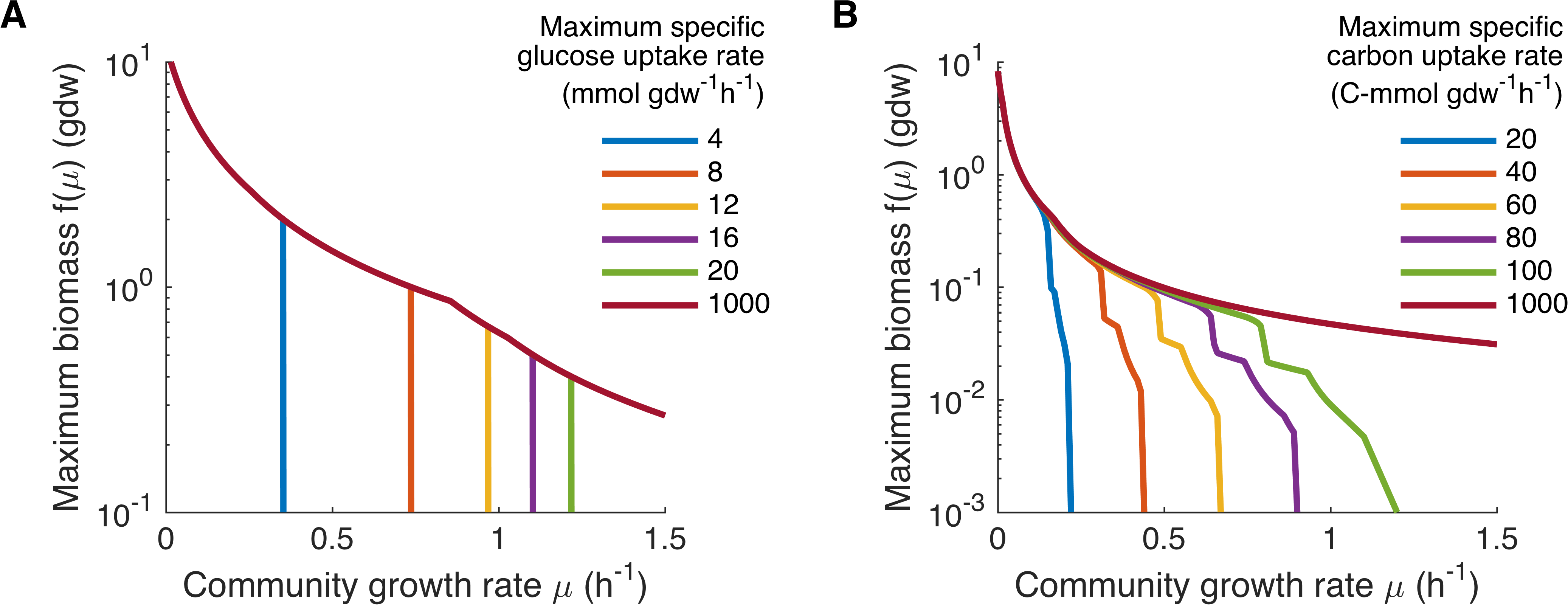


Figure ST1. Maximum community biomass *f*(*μ*) as a decreasing function of the community growth rate *μ* in (A) the community of auxotrophic *E. coli* mutants and (B) the ten-species gut microbiota model. The curves for the maximum specific glucose/carbon uptake rate for each organism constrained by various values are shown. When the maximum uptake rate is bounded by physiological values (< 1000 mmol gdw^-1^h^-1^), a theoretical cutoff community growth rate can be unambiguous defined above which not any biomass can be produced.

Though intuitive, we found that it is not necessarily true unless the metabolic network structure satisfies a certain properties or the upper bounds and lower bounds of the model satisfies some conditions which we argue that a general genome-scale metabolic network should satisfy. Here with some mathematical treatment, we arrive at two theorems. They are stated and discussed in this section. For the detailed proof, please see the next section. The first theorem is a general result limiting the value of *f*(*μ*):

**Theorem 1**. For a community network with proper elemental balance, *f*(*μ*), the maximum biomass of maxBM(*μ*), approaches 0 as *μ* approaches infinity, i.e.

$$\lim_{\mu\to\infty} f(\mu)=0$$

Theorem 1 ensures a way to find the maximum $\mu$ even though *f*(*μ*) is not completely decreasing in *μ*. By starting at a sufficiently large $\mu_{0}$ such that $f\left( \mu_{0} \right)\approx0$, decreasing $\mu$ gradually until $f\left( \mu\right)\geq X_{0}$ is satisfied results at the global maximum $\mu$. This guarantees that SteadyCom can be solved completely by scanning only the parameter $\mu$ backward. This algorithm outdoes the procedure used in community flux balance analysis (cFBA) [12] in which the number of LPs to be solved exponentially increases with the number of organisms in the model. However, a significant number of LPs still needs to be solved so that the maximum will not be missed.

Then, we have the second theorem that gives a sufficient condition for $f\left( \mu\right)$ to be decreasing in $\mu$ in terms of elementary modes (EMs). An EM is a minimal flux distribution in a metabolic network that cannot be decomposed into other ‘smaller’ flux distributions while all flux distributions can be expressed as a sum of EMs.

**Theorem 2.** If there exists a feasible solution of maxBM(*μ*) for a given *μ* such that the corresponding flux distribution can be decomposed into a set of EMs that either have zero fluxes for all biomass reactions or zero fluxes for all required reactions, i.e. fluxes constrained by positive lower bound (e.g. ATP maintenance) or negative upper bound (e.g. required uptake), then there is a feasible solution with the same objective function value for maxBM(*μ’*) for any $\mu^{'}<\mu$.

Theorem 2 provides a condition for the feasibility of a solution at *μ* being transferrable to any smaller *μ’*. The rationale of this condition is very simple. If there are no required reactions, i.e. reactions constrained to have non-zero fluxes, a flux distribution **V** at *μ* always remains feasible at any smaller *μ’* by multiplying the flux distribution by the factor *μ’* / *μ*. The growth rate will be exactly *μ’*. The abundances are unchanged. The nutrient uptakes are decreased and thus do not violate the constraints. But problems can arise when there are required reactions because the values of these fluxes will also be scaled down when multiplied by the factor *μ’* / *μ*. If the flux distribution at *μ* can be decomposed into two sub-flux distributions **V** = **V**_growth_ + **V**_req_, one responsible for the growth and the other responsible for the required conditions, then the new flux distribution (*μ’* / *μ*) × **V**_growth_ + **V**_req_ is still feasible. This is equivalent to the condition stated in the theorem that the flux distribution can be decomposed into EMs not have non-zero fluxes for both biomass reactions and the required reactions at the same time. Two corollaries leading to the condition for *f* (*μ*) to be decreasing in *μ* follow from Theorem 2:

**Corollary 1**. If for any $\mu\geq0$, there is an optimal solution of maxBM(*μ*) such that the flux distribution can be decomposed into a set of EMs that either have zero fluxes for all biomass reactions or zero fluxes for all required reactions, then *f* (*μ*) is decreasing in *μ*.

**Corollary 2.** If there are not any required reactions, then *f* (*μ*) is decreasing in *μ*.

Corollary 1 states that when the optimal solution can be decomposed into two flux sub-distributions as discussed above, *f* (*μ*) is decreasing. Corollary 2 simply states that when there is not any reaction required to have non-zero flux, *f*(*μ*) is safely guaranteed to be decreasing. Under either of the two conditions, a unique value of *μ* exists such that
*f* (*μ*) = *X_0_*, the minimum community biomass. A root found by any non-derivative-based root-finding algorithm is guaranteed to be a global maximum. However, usually in a metabolic model there is an ATP maintenance (ATPM) requirement imposed as a positive lower bound for the ATPM reaction flux. Therefore only Corollary 1 is practical. We will have more discussion on this in the following.

There are two scenarios for the ATPM necessarily coupled to biomass production in a microbial community. First, the ATPM of an organism is coupled to its own biomass production. This implies that when producing biomass optimally, the metabolic network structure forces additional ATP production more than the required amount for all the anabolic activities related to biomass production such that the additional ATP has to be hydrolyzed into ADP through the ATPM reaction. This is probably not true as it is a waste of energy that should not be favored by evolution. In the metabolic network there are always multiple pathways of generating ATP. Different end products generating/consuming ATP, NADH or other cofactors can be secreted to achieve different modes of catabolic metabolism such that it can be perfectly coupled to anabolism and biomass production to achieve efficient growth.

The second scenario is the ATPM of an organism coupled to the biomass production of another organism. EMs coupling the biomass reaction of one organism to the ATPM of another organism should reasonably exist as long as there is some chemical production coupled to the biomass production of one organism and meanwhile the produced chemical can be consumed by another organism for generating ATP. However, they should not be necessarily active in general. When the growth rate decreases by the factor *μ’* / *μ*, given the scaled flux distribution, (*μ’* / *μ*) × **V**, less nutrients are taken up by the community. If the unused substrates can be used for generating the amount of ATP that is reduced in the new distribution as an additional flux mode without any biomass production adding to the flux distribution (*μ’* / *μ*) × **V**, then the solution will be feasible. This is generally true because chemical conversion in a metabolic network is in general not coupled to biomass production at the stoichiometry level. In other words, there can still be non-zero fluxes satisfying the steady-state condition through many metabolic pathways interconverting biochemicals in the metabolic network though biomass production is set to be zero (such as glucose to lactate, acetate, or CO_2_). Indeed, biomass production usually decreases the yield of biochemical conversion because of simple mass balance.

A necessary condition for the involvement of EMs with biomass production of one organism coupled to the ATPM of another organism is that at $\mu=0$, with a positive ATPM requirement for each organism, some organisms cannot have any biomass because it cannot satisfy the ATPM requirement without the growth of the others. This situation was never observed in our computation. The above ideas and discussion may be proved formally in future attempts by formalizing these conditions on the metabolic network structure mathematically.

Finally, we give a counter example in which *f*(*μ*) can increase in a finite interval of *μ* in a toy network (Figure ST2). Note that the essential features in this toy network include (i) independent pathways for ATP production and biomass production in each organism; (ii) the only metabolite for ATP production in organism 2 (metabolite B) is tightly coupled to the biomass production of organism 1. See Table SM1 for the three optimal solutions to maxBM(*μ*) at increasing *μ* with *f*(*μ*) not monotonic decreasing.

Figure ST2. A toy network in which *f*(*μ*) can increase in a finite interval of *μ*

Table ST1. Optimal solutions of maxBM(*μ*) at *μ* = 1, 2, 4.

|  |  | *LB^k^_j_ X^k^* | *UB^k^_j_ X^k^* | Aggregate flux/Biomass value | | |
| --- | --- | --- | --- | --- | --- | --- |
|  |  |  |  | *μ* = 1 | *μ* = 2 | *μ* = 4 |
| *Organism 1* | |  |  |  |  |  |
| *X^1^* (biomass) | | 0 | 1000 *X^1^* | 1 | 1 | 0.5 |
| R1_1 | A[c1] 🡪 B[c1] + biomass[c1] | 0 | 1000 *X^1^* | 1 | 2 | 2 |
| R1_2 | C[c1] + ADP[c1] 🡪 D[c1] + ATP[c1] | 0 | 1000 *X^1^* | 1 | 1 | 0.5 |
| R1_ATPM | ATP[c1] 🡪 ADP[c1] | 1 *X^1^* | 1000 *X^1^* | 1 | 1 | 0.5 |
| Ex1_A | A[u]🡪A[c1] | 0 | 1000 *X^1^* | 1 | 2 | 2 |
| Ex1_B | B[c1]🡪 B[u] | 0 | 1000 *X^1^* | 1 | 2 | 2 |
| Ex1_C | C[u]🡪C[c1] | 0 | 1000 *X^1^* | 1 | 1 | 0.5 |
| Ex1_D | D[c1]🡪D[u] | 0 | 1000 *X^1^* | 1 | 1 | 0.5 |
| Ex1_Bm1 | biomass1[c1]🡪 biomass1[u] | 0 | 1000 *X^1^* | 1 | 2 | 2 |
| *Organism 2* | |  |  |  |  |  |
| *X^2^* (biomass) | | 0 | 1000 *X^2^* | 1 | 2 | 2 |
| R2_1 | E[c2] 🡪 biomass2[c2] | 0 | 1000 *X^2^* | 1 | 4 | 8 |
| R2_2 | B[c2] + ADP[c2] 🡪 F[c2] + ATP[c2] | 0 | 1000 *X^2^* | 1 | 2 | 2 |
| R2_ATPM | ATP[c2] 🡪 ADP[c2] | 1 *X^2^* | 1000 *X^2^* | 1 | 2 | 2 |
| Ex2_B | B[u]🡪B[c2] | 0 | 1000 *X^2^* | 1 | 2 | 2 |
| Ex2_E | E[u]🡪E[c2] | 0 | 1000 *X^2^* | 1 | 4 | 8 |
| Ex2_F | F[c2]🡪F[u] | 0 | 1000 *X^2^* | 1 | 2 | 2 |
| Ex2_Bm2 | biomass2[c2]🡪 biomass2[u] | 0 | 1000 *X^2^* | 1 | 4 | 8 |
| *Community space* | |  |  |  |  |  |
| Ex_A | 🡪A[u] | 0 | 2 | 1 | 2 | 2 |
| Ex_B | B[u]🡪 | 0 | 1000 | 0 | 0 | 0 |
| Ex_C | 🡪C[u] | 0 | 1 | 1 | 1 | 0.5 |
| Ex_D | D[u] 🡪 | 0 | 1000 | 1 | 1 | 0.5 |
| Ex_E | 🡪E[u] | 0 | 10 | 1 | 4 | 8 |
| Ex_F | F[u] 🡪 | 0 | 1000 | 1 | 2 | 2 |
| Ex_Bm1 | biomass1[u]🡪 | 0 | 1000 | 1 | 2 | 2 |
| Ex_Bm2 | biomass2[u]🡪 | 0 | 1000 | 1 | 4 | 8 |
| *f*(*μ*) = max(*X^1^* + *X^2^*) | |  |  | 2 | 3 | 2.5 |

Proof of theorems and corollaries

To formally prove the conditions, first we introduce elementary modes (EMs) and then mathematically formulate a microbial community as a multi-compartment metabolic network.

**Elementary modes**

For a metabolic network with *n* reactions, the stoichiometric matrix $\mathbf{S}$ and the set of reactions $\mathbf{J}$ divided into the set of irreversible reactions $\mathbf{J}_{irr}$ and the set of reversible reactions $\mathbf{J}_{rev}$, a flux vector $\mathbf{e}=\left[ e_{1} e_{2}\ldots e_{n} \right]^{T}$ is an elementary mode if the following conditions are satisfied [13]:

Steady-state: $\mathbf{Se}=0$.

Thermodynamic feasibility: $e_{j}\geq0 \forall j\in\mathbf{J}_{irr}$

Elementarity:
$\mathbf{e}$ cannot be expressed as the sum of any two non-zero flux vectors, i.e.

$$\mathbf{e}\boldsymbol{\neq}\mathbf{e}_{1}+\mathbf{e}_{2}$$

for any flux vectors $\mathbf{e}_{1}=\left[ e_{11} e_{21}\ldots e_{n1} \right]^{T}\neq\mathbf{0},\mathbf{e}_{2}=\left[ e_{12} e_{22}\ldots e_{n2} \right]^{T}\neq\mathbf{0}$, that have the following properties:

$\mathbf{e}_{1},\mathbf{e}_{2}$ also satisfy (i) and (ii), the steady-state condition and thermodynamic feasibility.

$e_{j1}=e_{j2}=0$ whenever $e_{j}=0$.

An elementary mode (EM) is thus a minimal operational unit in a metabolic network because no any proper subset of reactions in an EM alone can carry any flux while the whole set of reaction in an EM can. This is called ‘elementarity’ or ‘non-decomposability’ [14,15].

**Decomposition of flux distributions into EMs**

An important consequence of the elementarity of EMs is that every flux distribution can be represented as a sum of EMs with the so-called ‘no-cancellation’ property:

Every flux distribution $\mathbf{v}$ can be decomposed into a non-negative weighted sum of the whole set of EMs:

$$\mathbf{v}=\sum_{p\in\mathbf{EM}} \alpha_{p}\mathbf{e}_{\mathbf{p}}, \alpha_{p}\geq0 \forall p\in\mathbf{EM}$$

where **EM** is the index set for all EMs. The decomposition of a flux distribution by EMs possesses the important ‘no-cancellation’ property [14,15]:

$\mathbf{PE}\left( \mathbf{e}_{p} \right)\boldsymbol{\subset}\mathbf{PE}\left( \mathbf{v} \right) \mathrm{and}\mathbf{NE}\left( \mathbf{e}_{p} \right)\boldsymbol{\subset}\mathbf{N}\mathbf{E}\left( \mathbf{v} \right) \mathrm{if}\alpha_{p}>0 for p\in\mathbf{EM}$ (S1)

where $\mathbf{PE}\left( \mathbf{v} \right)=\left\{ j\in\mathbf{J |}v_{j}>0 \right\}$ and $\mathbf{NE}\left( \mathbf{v} \right)=\left\{ j\in\mathbf{J |}v_{j}<0 \right\}$ are the index sets of positive and negative fluxes in $\mathbf{v}$ respectively. In other words, all EMs decomposing the flux distribution $\mathbf{v}$ have the same direction of fluxes as $\mathbf{v}$ for any non-zero fluxes in the EMs. The proof for this property can be found in [14].

**Microbial community as a multi-compartment metabolic network**

In what follows, the metabolic model for a microbial community is formally treated as a multi-compartment metabolic network. We first explicitly define additional sets, functions and variables for clearer mathematical expression. Then the composite stoichiometric matrix is presented. Finally a set of reactions and a set of EMs relevant to the condition under which *f*(*μ*) is non-increasing with *μ* are defined.

*Additional sets, functions and variables*

Assume that there are *K* organisms in the community and $\mathbf{K=}\left\{ 1,\ldots,K \right\}$. Define explicitly the set of organism-specific metabolites $\mathbf{I}^{k}$:

$$\mathbf{I}^{k}=\left\{ 1,\ldots,m_{in}^{k}-1,m_{in}^{k},m_{in}^{k}+1,\ldots,m_{in}^{k}+m_{ex}^{k} \right\} \forall k\in\mathbf{K}$$

where $m_{in}^{k}$ is the number of intracellular metabolites for organism *k* and $m_{ex}^{k}$ is the number of extracellular metabolites for organism *k.* The subset of extracellular metabolites is given by:

$$\mathbf{I}_{ex}^{k}=\left\{ m_{in}^{k}+1,\ldots,m_{in}^{k}+m_{ex}^{k} \right\} \forall k\in\mathbf{K}$$

For community metabolites, define:

$$\mathbf{I}^{com}=\{1,\ldots,m^{0}\}$$

For all organism *k*, define an injective function ${ec}^{k}:\mathbf{I}_{ex}^{k}\to\mathbf{I}^{com}$ such that

$${ec}^{k}\left( i^{'} \right)=i$$

if $i^{'}\in\mathbf{I}_{ex}^{k}$ and $i\in\mathbf{I}^{com}$ refer to the same metabolite in the extracellular space of organism *k* and in the community space respectively.

Define also the index set for all organism-specific reactions $\mathbf{J}^{k}$:

$$\mathbf{J}^{k}=\left\{ 1,\ldots,n_{in}^{k}-1,n_{in}^{k},n_{in}^{k}+1,\ldots,n^{k} \right\} \forall k\in\mathbf{K}$$

where $n_{in}^{k}$ is the number of internal (non-exchange) reactions for organism *k* and $n^{k}$ is the total number of reactions for organism *k.* Note that the number of exchange reactions is equal to the number of extracellular metabolites in general, i.e. $n^{k}-n_{in}^{k}=m_{ex}^{k}$, such that every extracellular metabolite can be transported between the extracellular compartment of an organism and the community space with the rate limited by the lower/upper bound of the corresponding exchange reaction. The community exchange reactions similarly represent the net export or uptake of community metabolites. The number of community exchange reactions is equal to the number of community metabolites. Let $\mathbf{J}^{0}$ be the set of community exchange reactions:

$$\mathbf{J}^{0}=\{1,\ldots,m^{0}\}$$

Zero is used as superscript for the convenience of notation. Taking the usual convention for the direction of exchange reactions in which positive flux means export and negative flux mean uptake, the flux of a community exchange reaction $V_{i}^{com}$ for $i\boldsymbol{\in}\mathbf{I}^{com}$ can be related to the uptake rate and export rate of a community metabolite defined in SteadyCom:

$$V_{i}^{0}=e_{i}^{c}-u_{i}^{c} \forall i\boldsymbol{\in}\mathbf{I}^{com}$$

In SteadyCom, $u_{i}^{c}$ is a non-negative constant for the uptake rate of community metabolite and $e_{i}^{c}$ is a non-negative variable for the export rate. $V_{i}^{0}$ is thus bounded below by:

$V_{i}^{0}\geq-u_{i}^{c}$ (S2)

The constraint for the mass balance of community metabolites becomes:

$\sum_{k\in\mathbf{K}} V_{ex\left( i \right)}^{k}-V_{i}^{0}=0 \forall i\in\mathbf{I}^{com}$ (S3)

The set of all reactions $\mathbf{J}$ (including the community exchange reactions) is defined as:

$$\mathbf{J=}\left\{ \left( j,k \right) | j\in\mathbf{J}^{k} for 0\leq k\leq K \right\}$$

*Stoichiometric matrix*

The stoichiometric matrix $\mathbf{S}^{k}$ for each organism $k\in\mathbf{K}$ can be structured in the following way:

$$\mathbf{S}^{k}\mathbf{=}\left[ \begin{matrix} \mathbf{S}_{in,in}^{k} & \boldsymbol{0} \\ \mathbf{S}_{ex,in}^{k} & \mathbf{S}_{ex,ex}^{k} \end{matrix} \right]\boldsymbol{=}\left[ \begin{matrix} S_{1,1}^{k} & \boldsymbol{\cdots} & S_{1,n_{in}^{k}}^{k} & 0 & 0 & \boldsymbol{\cdots} & 0 \\ \boldsymbol{\vdots} & \boldsymbol{\ddots} & \boldsymbol{\vdots} & \boldsymbol{\vdots} & \boldsymbol{\vdots} & \boldsymbol{\ddots} & \boldsymbol{\vdots} \\ S_{m_{in}^{k},1}^{k} & \boldsymbol{\cdots} & S_{m_{in}^{k},n_{in}^{k}}^{k} & 0 & 0 & \boldsymbol{\cdots} & 0 \\ S_{m_{in}^{k}+1,1}^{k} & \boldsymbol{\cdots} & S_{m_{in}^{k}+1,n_{in}^{k}}^{k} & -1 & 0 & \boldsymbol{\cdots} & 0 \\ S_{m_{in}^{k}+2,1}^{k} & \boldsymbol{\cdots} & S_{m_{in}^{k}+2,n_{in}^{k}}^{k} & 0 & -1 & \boldsymbol{\ddots} & \boldsymbol{\vdots} \\ \boldsymbol{\vdots} & \boldsymbol{\ddots} & \boldsymbol{\vdots} & \boldsymbol{\vdots} & \boldsymbol{\ddots} & \boldsymbol{\ddots} & 0 \\ S_{m_{in}^{k}+m_{ex}^{k},1}^{k} & \boldsymbol{\cdots} & S_{m_{in}^{k}+m_{ex}^{k},n_{in}^{k}}^{k} & 0 & \boldsymbol{\cdots} & 0 & -1 \end{matrix} \right]$$

where $\mathbf{S}_{in,in}^{k}$ is the stoichiometric matrix for intracellular metabolites and internal reactions, $\mathbf{S}_{ex,in}^{k}$ is the stoichiometric matrix for extracellular metabolites and internal reactions, and $\mathbf{S}_{ex,ex}^{k}$ is the stoichiometric matrix for the extracellular metabolites and the corresponding exchange reactions, which is equal to $-\mathbf{Id}_{m_{ex}^{k}}$, the negative of the identity matrix of dimension $m_{ex}^{k}$. This structure is a conventional structure used in metabolic networks (up to permutation of columns and rows) in which the exchange reactions represent the input and output of the whole system and are not mass-balanced. A positive exchange flux means net production and a negative exchange flux means net consumption. The mass balance of the whole microbial community can be described by the following composite stoichiometric matrix $\mathbf{S}$:

$$\mathbf{S=}\left[ \begin{matrix} \mathbf{S}^{1} & \boldsymbol{0} & \boldsymbol{\cdots} & \boldsymbol{0} & \boldsymbol{0} \\ \boldsymbol{0} & \mathbf{S}^{2} & \boldsymbol{\ddots} & \boldsymbol{\vdots} & \boldsymbol{\vdots} \\ \boldsymbol{\vdots} & \boldsymbol{\ddots} & \boldsymbol{\ddots} & \boldsymbol{0} & \boldsymbol{\vdots} \\ \boldsymbol{0} & \boldsymbol{\cdots} & \boldsymbol{0} & \mathbf{S}^{K} & \boldsymbol{0} \\ \mathbf{U}^{1} & \mathbf{U}^{2} & \boldsymbol{\cdots} & \mathbf{U}^{K} & \boldsymbol{-}\mathbf{Id}_{m^{0}} \end{matrix} \right]$$

where $\mathbf{U}^{k}\boldsymbol{=}\left[ U_{ij}^{k} \right]\in\mathbb{R}^{m^{0}\times m_{ex}^{k}}$ for $k=1,\ldots,K$ is a matrix connecting extracellular metabolites and community metabolites such that positive flux of an organism-specific exchange reaction represents transporting a metabolite from the extracellular space of an organism to the community space. Mathematically,

$$U_{ij}^{k}=\left\{ \begin{matrix} 1 & \mathrm{if} & \exists i^{'}\in\mathbf{I}_{ex}^{k},j\in\mathbf{J}^{k} such that S_{i^{'}j}^{k}=-1,ec^{k}\left( i^{'} \right)=i\mathrm{and} j>n_{in}^{k} \\ 0 & & \mathrm{otherwise} \end{matrix} \right.$$

The last $m_{com}$ columns in $\mathbf{S}$ are for the community exchange reactions with fluxes $V_{i}^{com}$ for $i\in\mathbf{I}^{com}$ representing the net production and consumption of the community. The last $m_{com}$ rows in $\mathbf{S}$ represent the community metabolites. The mass balance of these rows is identical to the constraint (S2).

With this the microbial community network structure, it is obvious that if the flux vector

$$\mathbf{V}=\left[ \begin{matrix} {\mathbf{V}^{1}}^{T} & {\mathbf{V}^{2}}^{T} & \cdots& {\mathbf{V}^{K}}^{T} & {\mathbf{V}^{0}}^{T} \end{matrix} \right]^{T}=\left[ V_{1}^{1} \ldots V_{n^{1}}^{1} V_{1}^{2} \ldots V_{n^{2}}^{2} \ldots V_{1}^{K}\ldots V_{n^{K}}^{K} V_{1}^{0}\ldots V_{m^{0}}^{0} \right]^{T}$$

and the biomass vector

$$\mathbf{X}\boldsymbol{=[}X^{1} X^{2}\ldots X^{K}]$$

together form a solution of maxBM(*μ*). Then $\mathbf{V}$ satisfies the steady-state condition

$$\mathbf{SV=0}$$

as well as the thermodynamic feasibility for all species-specific reactions. It can thus be decomposed into elementary modes of the community model.

*Required reactions*

To establish a condition for *f*(*μ*) to be non-increasing in *μ*, let $\mathbf{J}^{req}$ be the set of ‘required’ reactions:

$$\mathbf{J}^{req}=\left\{ \left( j,k \right)\in\mathbf{J} \right| j\in\mathbf{J}^{k} for some k\in\mathbf{K} such that {UB}_{j}^{k}<0 \mathrm{or}{LB}_{j}^{k}>0\}.$$

These reactions are ‘required’ in the sense that the bounds impose non-zero fluxes on the reactions. Let $\mathbf{e}_{p}=\left[ e_{1,p}^{1}\ldots e_{m_{0},p}^{0} \right]^{T}$ be an EM. Let $\mathbf{E}\mathbf{M}^{gr}$ be the index set for EMs pertaining to growth, which have non-zero fluxes for any of the biomass reactions:

$$\mathbf{E}\mathbf{M}^{gr}\boldsymbol{=}\left\{ p\in\mathbf{EM}| e_{biomass}^{k^{'}}\neq0 for some k^{'}\in\mathbf{O} \right\}$$

Let $\mathbf{E}\mathbf{M}^{gr,req}$ be the index set for EMs that couple any of the required reactions with any biomass reactions:

$$\mathbf{E}\mathbf{M}^{gr,req}\boldsymbol{=}\left\{ p\in\mathbf{EM}| e_{jp}^{k}\neq0 for some \left( j,k \right)\in\mathbf{J}^{req} \mathrm{and}e_{biomass}^{k^{'}}\neq0 for some k^{'}\in\mathbf{K} \right\}\boldsymbol{.}$$

Clearly $\mathbf{E}\mathbf{M}^{gr,req}\boldsymbol{\subset}\mathbf{E}\mathbf{M}^{gr}$.

With the above definitions, first, we can prove Theorem 1.

**Theorem 1**. For a community network with proper elemental balance, *f*(*μ*), the maximum biomass of maxBM(*μ*), approaches 0 as *μ* approaches infinity, i.e.

$$\lim_{\mu\to\infty} f(\mu)=0$$

Proof:

Let $\mathbf{E=}\left[ \begin{matrix} \mathbf{E}^{1} & \boldsymbol{\cdots} & \mathbf{E}^{K} & \mathbf{E}^{0} \end{matrix} \right]$ be the elemental matrix for all metabolites in the network where $\mathbf{E}^{k}\mathbf{=}\left[ E_{qi}^{k} \right]\in\mathbb{R}^{N_{E}\times m^{k}}$. $E_{qi}^{k}\geq0$ is the stoichiometry for element *q* in the chemical formula of metabolite *i* in organism *k* (*k* = 0 for community metabolites). $N_{E}$ is the total number of chemical elements and $m^{k}=m_{in}^{k}+m_{ex}^{k} \mathrm{for}1\leq k\leq K$ is the total number of metabolites in organism *k*. For a community network with proper elemental balance,

$$\mathbf{E}\mathbf{S=}\left[ \begin{matrix} \mathbf{E}^{1} & \boldsymbol{\cdots} & \mathbf{E}^{K} & \mathbf{E}^{0} \end{matrix} \right]\left[ \begin{matrix} \mathbf{S}^{1} & \boldsymbol{0} & \boldsymbol{\cdots} & \boldsymbol{0} & \boldsymbol{0} \\ \boldsymbol{0} & \mathbf{S}^{2} & \boldsymbol{\ddots} & \boldsymbol{\vdots} & \boldsymbol{\vdots} \\ \boldsymbol{\vdots} & \boldsymbol{\ddots} & \boldsymbol{\ddots} & \boldsymbol{0} & \boldsymbol{\vdots} \\ \boldsymbol{0} & \boldsymbol{\cdots} & \boldsymbol{0} & \mathbf{S}^{K} & \boldsymbol{0} \\ \mathbf{U}^{1} & \mathbf{U}^{2} & \boldsymbol{\cdots} & \mathbf{U}^{K} & \boldsymbol{-}\mathbf{I}_{m^{0}} \end{matrix} \right]\boldsymbol{=}\left[ \begin{matrix} \mathbf{0} & \boldsymbol{-}\mathbf{E}^{0} \end{matrix} \right]$$

Therefore,

$$\mathbf{E}\left( \mathbf{SV} \right)\mathbf{=E}\left( \mathbf{0} \right)$$

$$\left[ \begin{matrix} \mathbf{0} & \boldsymbol{-}\mathbf{E}^{0} \end{matrix} \right]\left[ \begin{matrix} \mathbf{V}^{1} \\ \boldsymbol{\vdots} \\ \mathbf{V}^{K} \\ \mathbf{V}^{0} \end{matrix} \right]\mathbf{=0}$$

$$\mathbf{E}^{0}\mathbf{V}^{0}\mathbf{=0}$$

$$\Rightarrow\sum_{i\in\mathbf{I}^{com}} E_{qi}^{0}V_{i}^{0}=0 \mathrm{for}q=1,\ldots, N_{E}$$

Note that for such a perfectly mass-balanced network, all the sink and demand metabolites, as well as the biomass produced must be treated as community metabolites being properly exported and imported through the community space. Otherwise the biomass reaction, for example, is not mass balanced. Let $V_{biomass,k}^{0}$ be the corresponding export flux for the biomass of organism *k*. It has a value equal to the flux of the biomass reaction $V_{biomass}^{k}$ given the usual exchange reaction defined for biomass export (using stoichiometry 1 and -1). Let $\mathbf{E}_{biomass}^{0}\in\mathbb{R}^{N_{E}\times K}$and $\mathbf{E}_{mets}^{0}\in\mathbb{R}^{N_{E}\times(m^{0}-K)}$ be the elemental matrices for the biomass of all organisms and for other community metabolites respectively such that

$$\mathbf{E}^{0}=\left[ \begin{matrix} \mathbf{E}_{mets}^{0} & \mathbf{E}_{biomass}^{0} \end{matrix} \right].$$

Similarly, let $\mathbf{V}_{biomass}^{0}$ and $\mathbf{V}_{mets}^{0}$ be the exchange fluxes for the biomass of all organisms and other community metabolites respectively such that

$$\mathbf{V}^{0}\mathbf{=}\left[ \begin{matrix} \mathbf{V}_{mets}^{0} \\ \mathbf{V}_{biomass}^{0} \end{matrix} \right]\boldsymbol{.}$$

Then we have

$$\mathbf{E}_{biomass}^{0}\mathbf{V}_{biomass}^{0}=\mathbf{E}_{mets}^{0}\left( -\mathbf{V}_{mets}^{0} \right)\leq\mathbf{E}_{mets}^{0}\mathbf{U}_{mets}^{0}$$

Here $-\mathbf{U}_{mets}^{0}=[-u_{i}^{c}]$ provides a lower bound for $\mathbf{V}_{mets}^{0}$ by (S2). The inequality holds since $\mathbf{E}_{mets}^{0}$ is non-negative. Note that the LHS and RHS are both non-negative. Let $\mathbf{w}_{E}\in\mathbb{R}^{N_{E}}$ be the vector of molecular weight of the chemical elements corresponding to the row of $\mathbf{E}$, which is also non-negative. The molecular weights of the biomass for each organism is given by the vector:

$$\mathbf{w}^{T}=\mathbf{w}_{E}^{T}\mathbf{E}_{biomass}^{0}=\left[ \begin{matrix} w^{1} & \cdots& w^{K} \end{matrix} \right]$$

Then we have

$$\mathbf{w}^{T}\mathbf{V}_{biomass}^{0}=\mathbf{w}_{E}^{T}\mathbf{E}_{biomass}^{0}\mathbf{V}_{biomass}^{0}\leq\mathbf{w}_{E}^{T}\mathbf{E}_{mets}^{0}\mathbf{U}_{mets}^{0}$$

$$\boldsymbol{\Rightarrow}\sum_{\boldsymbol{k=}1}^{K} w^{k}V_{biomass}^{k}\boldsymbol{\leq}C=\mathbf{w}_{E}^{T}\mathbf{E}_{mets}^{0}\mathbf{U}_{mets}^{0}$$

For a proper model, all biomass has weight. We take the one with a minimum weight:

$$w_{min}=\min\left( \mathbf{w}_{biomass} \right)>0$$

Then we have

$$\sum_{\boldsymbol{k=}1}^{K} V_{biomass}^{k}\boldsymbol{=}\frac{1}{w_{min}}\sum_{\boldsymbol{k=}1}^{K} w_{min}V_{biomass}^{k}\leq\frac{1}{w_{min}}\sum_{\boldsymbol{k=}1}^{K} w^{k}V_{biomass}^{k}\leq\frac{C}{w_{min}}$$

Therefore, the sum of biomass is always bounded by:

$$\sum_{\boldsymbol{k=}1}^{K} X^{k}\boldsymbol{=}\frac{1}{\mu}\sum_{\boldsymbol{k=}1}^{K} V_{biomass}^{k}\boldsymbol{\leq}\frac{C}{\mu w_{min}}$$

Since $C$ and $w_{min}$ are both constant, the maximum community biomass *f*(*μ*) approaches to zero as $\mu$ approaches infinity. ■

**Theorem 2.** If there exists a feasible solution $(\mathbf{V,X)}$ of maxBM(*μ*) for a given *μ* such that $\mathbf{V}$ can be decomposed into EMs not belonging to the set $\mathbf{E}\mathbf{M}^{gr,req}$, then there is a feasible solution with the same objective function value for maxBM(*μ’*) for any $\mu^{'}<\mu$.

Proof:

Let $\mathbf{EM}\boldsymbol{=}\left\{ 1,\ldots,P \right\}$ be the index set for the whole set of *P* EMs of the community network. W.L.O.G., assume the following decomposition of **V**:

$$\mathbf{V}=\sum_{p\in\mathbf{E}\mathbf{M}^{gr}} \alpha_{p}\mathbf{e}_{p}+\sum_{p\in\boldsymbol{EM\backslash}\mathbf{E}\mathbf{M}^{gr}} \alpha_{p}\mathbf{e}_{p}, \alpha_{p}\geq0 \forall p\in\mathbf{EM}$$

Since $\mathbf{V}$ can be decomposed into EMs not belonging to the set $\mathbf{E}\mathbf{M}^{gr,req}$, there exists a set of weight $\alpha_{p}$ such that:

$$\alpha_{p}=0 \forall p\in\mathbf{E}\mathbf{M}^{gr,req}$$

And by definition,

$$e_{j,p}^{k}=0 \forall\left( j,k \right)\in\mathbf{J}^{req}, p\in\mathbf{E}\mathbf{M}^{gr}\backslash\mathbf{E}\mathbf{M}^{gr,req}$$

$\Rightarrow\alpha_{p}e_{j,p}^{k}=0 \forall\left( j,k \right)\in\mathbf{J}^{req}, p\in\mathbf{E}\mathbf{M}^{gr}$ (S4)

For any $\mu^{'}$ such that $0\leq\mu^{'}<\mu$, let:

$$\mathbf{V}'=\frac{\mu^{'}}{\mu}\left( \sum_{p\in\mathbf{E}\mathbf{M}^{gr}} \alpha_{p}\mathbf{e}_{p} \right)+\sum_{p\in\boldsymbol{EM\backslash}\mathbf{E}\mathbf{M}^{gr}} \alpha_{p}\mathbf{e}_{p}$$

This newly defined flux distribution satisfies the steady-state because each EM does:

$$\mathbf{S}\mathbf{V}^{'}=\mathbf{0}$$

By the no cancellation property (S1), for any reaction $\left( j,k \right)\in\mathbf{J}^{req}$,

$$V_{j}^{k}>0\Rightarrow\alpha_{p}e_{jp}^{k}\geq0 \forall p\in\mathbf{EM},$$

$$V_{j}^{k}=0\Rightarrow\alpha_{p}e_{jp}^{k}=0 \forall p\in\mathbf{EM},$$

$$V_{j}^{k}<0\Rightarrow\alpha_{p}e_{jp}^{k}\leq0 \forall p\in\mathbf{EM}.$$

Therefore,

$${V_{j}^{k}}^{'}=\frac{\mu^{'}}{\mu}\left( \sum_{p\in\mathbf{E}\mathbf{M}^{gr}} \alpha_{p}e_{jp}^{k} \right)+\sum_{p\in\boldsymbol{EM\backslash}\mathbf{E}\mathbf{M}^{gr}} \alpha_{p}e_{jp}^{k}\left\{ \begin{matrix} \leq V_{j}^{k} & \mathrm{if} & V_{j}^{k}>0 \\ =0 & \mathrm{if} & V_{j}^{k}=0 \\ \geq V_{j}^{k} & \mathrm{if} & V_{j}^{k}<0 \end{matrix} \right. \forall\left( j,k \right)\in\mathbf{R}$$

For any reaction $\left( j,k \right)\in\boldsymbol{J\backslash}\mathbf{J}^{req}$, since ${LB}_{j}^{k}\leq0$ and ${UB}_{j}^{k}\geq0$, it follows that ${V_{j}^{k}}^{'}$ satisfies also the flux capacity constraint:

$${LB}_{j}^{k}X^{k}\leq0\leq{V_{j}^{k}}^{'}\leq V_{j}^{k}\leq UB_{j}^{k}X^{k}\mathrm{for}\left( j,k \right)\in\boldsymbol{J\backslash}\mathbf{J}^{req} \mathrm{if}V_{j}^{k}\geq0,$$

$${LB}_{j}^{k}X^{k}\leq V_{j}^{k}\leq{V_{j}^{k}}^{'}\leq0\leq UB_{j}^{k}X^{k}\mathrm{for}\left( j,k \right)\in\boldsymbol{J\backslash}\mathbf{J}^{req} \mathrm{if}V_{j}^{k}<0.$$

From (S4), for each reaction $\left( j,k \right)\in\mathbf{J}^{req}$,

$${V_{j}^{k}}^{'}=\frac{\mu^{'}}{\mu}\left( \sum_{p\in\mathbf{E}\mathbf{M}^{gr}} 0 \right)+\sum_{p\in\boldsymbol{EM\backslash}\mathbf{E}\mathbf{M}^{gr}} \alpha_{p}e_{j,p}^{k}=V_{j}^{k}$$

thus it also satisfies the flux capacity constraint.

For the biomass reaction, since $e_{biomass,p}^{k}=0 \forall p\in\boldsymbol{EM\backslash}\mathbf{E}\mathbf{M}^{gr}$

$${V_{biomass}^{k}}^{'}=\frac{\mu^{'}}{\mu}\left( \sum_{p\in\mathbf{E}\mathbf{M}^{gr}} \alpha_{p}e_{jp}^{k} \right)=\frac{\mu^{'}}{\mu}V_{biomass}^{k}=X^{k}\mu^{'}.$$

The new flux distribution also satisfies the community steady-state as well.

Therefore, $(\mathbf{V}^{\mathbf{'}}\mathbf{,X)}$ is a feasible solution of maxBM(*μ’*) with the same objective value $\sum_{k\in\mathbf{K}} X^{k}$. ■

**Corollary 1**. If for any $\mu\geq0$, there is an optimal solution $(\mathbf{V,X)}$ of maxBM(*μ*) such that $\mathbf{V}$ can be decomposed into EMs not in $\mathbf{E}\mathbf{M}^{gr,req}$, then *f* (*μ*) is decreasing in *μ*.

Proof:

For any $\mu\geq0$, by Theorem 2, given an optimal solution $(\mathbf{V,X)}$ of maxBM(*μ*) such that $\mathbf{V}$ can be decomposed into EMs not in $\mathbf{E}\mathbf{M}^{gr,req}$**,** there is a solution $(\mathbf{V',X)}$ of maxBM(*μ’*) for any $0\leq\mu^{'}<\mu$.

$$f\left( \mu^{'} \right)=max\left( \sum_{k\in\mathbf{K}} X^{k} \right)\geq\sum_{k\in\mathbf{K}} X^{k}=f(\mu)$$

Therefore *f* (*μ*) is decreasing in *μ.* ■

**Corollary 2.** If the set of required reactions $\mathbf{J}^{req}$ is empty, then *f* (*μ*) is decreasing in *μ*.

Proof: Empty $\mathbf{J}^{req}$ implies empty $\mathbf{E}\mathbf{M}^{gr,req}$**.** Any flux distribution can be decomposed into EMs not in $\mathbf{E}\mathbf{M}^{gr,req}$. By Corollary 1, *f* (*μ*) is decreasing in *μ*. ■

For the second scenario of ATPM coupled to biomass production discussed in the previous section, with the proof of Theorem 2 presented, we can look at it more explicitly through the equations. From the proof of Theorem 2, though the ATPM flux

$$V_{ATPM}^{k}-{V_{ATPM}^{k}}^{'}=\left( 1-\frac{\mu^{'}}{\mu} \right)\left( \sum_{p\in\mathbf{E}\mathbf{M}^{gr}} \alpha_{p}e_{ATPM,p}^{k} \right)$$

is reduced if there are EMs coupling ATPM and biomass production, i.e.

$$\alpha_{p}e_{ATPM,p}^{k}>0 for some p\in\mathbf{E}\mathbf{M}^{gr}\boldsymbol{,}$$

we also see that at the same time the community uptake and production are also decreased:

$$V_{i}^{0}-{V_{i}^{0}}^{'}=\left( 1-\frac{\mu^{'}}{\mu} \right)\left( \sum_{p\in\mathbf{E}\mathbf{M}^{gr}} \alpha_{p}e_{i,p}^{0} \right)\mathrm{for}i\in\mathbf{I}^{com}$$

The decrease is associated with the decrease in biomass production:

$$\sum_{k=1}^{K} V_{biomass}^{k}-\sum_{k=1}^{K} {V_{biomass}^{k}}^{'}=\left( 1-\frac{\mu^{'}}{\mu} \right)\left( \sum_{k=1}^{K} \sum_{p\in\mathbf{E}\mathbf{M}^{gr}} \alpha_{p}e_{biomass,p}^{k} \right)$$

If the unused substrates can be used for generating the amount of ATP that is reduced in the new distribution as an additional flux mode without any biomass production adding to the solution $\mathbf{V}^{'}$, then the solution will be feasible.

References

1. Cressman R, Tao Y. The replicator equation and other game dynamics. Proc Natl Acad Sci. 2014 Jul 22;111(Supplement_3):10810–7.

2. An YH, Friedman RJ. Concise review of mechanisms of bacterial adhesion to biomaterial surfaces. J Biomed Mater Res. 1998 Jan;43(3):338–48.

3. Liang X, Bittinger K, Li X, Abernethy DR, Bushman FD, FitzGerald GA. Bidirectional interactions between indomethacin and the murine intestinal microbiota. Elife. 2015 Dec 23;4(DECEMBER2015):1–22.

4. Faith JJ, Guruge JL, Charbonneau M, Subramanian S, Seedorf H, Goodman AL, et al. The long-term stability of the human gut microbiota. Science. 2013;341(July):1237439.

5. David LA, Materna AC, Friedman J, Campos-Baptista MI, Blackburn MC, Perrotta A, et al. Host lifestyle affects human microbiota on daily timescales. Genome Biol. 2014;15(7):R89.

6. Caporaso JG, Lauber CL, Costello EK, Berg-Lyons D, Gonzalez A, Stombaugh J, et al. Moving pictures of the human microbiome. Genome Biol. 2011;12(5):R50.

7. Lozupone CA, Stombaugh JI, Gordon JI, Jansson JK, Knight R. Diversity, stability and resilience of the human gut microbiota. Nature. 2012 Sep 12;489(7415):220–30.

8. Stein RR, Bucci V, Toussaint NC, Buffie CG, Rätsch G, Pamer EG, et al. Ecological Modeling from Time-Series Inference: Insight into Dynamics and Stability of Intestinal Microbiota. von Mering C, editor. PLoS Comput Biol. 2013 Dec 12;9(12):e1003388.

9. Fisher CK, Mehta P. Identifying Keystone Species in the Human Gut Microbiome from Metagenomic Timeseries Using Sparse Linear Regression. White BA, editor. PLoS One. 2014 Jul 23;9(7):e102451.

10. Coyte KZ, Schluter J, Foster KR. The ecology of the microbiome: Networks, competition, and stability. Science (80- ). 2015 Nov 6;350(6261):663–6.

11. Bashan A, Gibson TE, Friedman J, Carey VJ, Weiss ST, Hohmann EL, et al. Universality of human microbial dynamics. Nature. 2016 Jun 8;534(7606):259–62.

12. Khandelwal R a, Olivier BG, Röling WFM, Teusink B, Bruggeman FJ. Community flux balance analysis for microbial consortia at balanced growth. PLoS One. 2013 Jan;8(5):e64567.

13. Schuster S, Hilgetag C. On elementary flux modes in biochemical reaction systems at steady state. J Biol Syst. 1994;2:165–82.

14. Schuster S, Hilgetag C, Woods JH, Fell D a. Reaction routes in biochemical reaction systems: algebraic properties, validated calculation procedure and example from nucleotide metabolism. J Math Biol. 2002 Aug;45(2):153–81.

15. Zanghellini J, Ruckerbauer DE, Hanscho M, Jungreuthmayer C. Elementary flux modes in a nutshell: Properties, calculation and applications. Biotechnol J. 2013 Jun 21;1–8.
