# Supplementary material for: SteadyCom: Predicting microbial abundances while ensuring community stability
Source: PLoS Comput Biol. 2017 May 15;13(5):e1005539. doi: 10.1371/journal.pcbi.1005539 (PMC5448816; doi:10.1371/journal.pcbi.1005539)
Supplement: S2 Text — (PDF) [file pcbi.1005539.s017.pdf]

S2 Text SUPPLEMENTARY RESULTS

Simulation of gut microbiota by constraining the known abundances for minor species

In this section, the relative abundances of the minority of the community known from experimental data were used to constrain the model. The rationale originates from the fact that among the published gut microbiome data, large variations are usually observed between the Firmicutes (in particular Clostridia) and the Bacteroidetes while other bacteria have low relative abundances. The abundances of each group of Proteobacteria, Actinobacteria and the lactic acid bacteria (*S. thermophilus*, *E. faecalis* and *L. casei*) were respectively constrained to be no greater than 5%, based on reported values [1–7].

Fiber-uptake-rate simulations

The fiber-uptake-rate (FUR) simulations were repeated for the estimated American diet and two high-fiber low-carbohydrate diet interventions. These additional diets were simulated by decreasing the amount of carbohydrate available to the microbiota by 50% and 100% of the estimated American diet respectively, while subsequently increasing the dietary fiber to ensure the same nutrient mass was supplied in all simulations (referred to as diet 'wHF1' and 'wHF2', respectively; S7 Figure). The community shifts from the dominance of *B. thetaiotaomicron* to *F. prausnitzii* as the FUR of *B. thetaiotaomicron* increases while high-fiber low-carbohydrate interventions result in an increase in *B. thetaiotaomicron*'s abundance and a decrease in *F. prausnitzii*'s abundance at high FURs of *B. thetaiotaomicron* (S7 Figure). The abundances of *B. thetaiotaomicron* and *F. prausnitzii* have the greatest variation among the unconstrained species (S7 Figure B – C). This shift of the predominant species at high *B. thetaiotaomicron*'s FURs can be explained by the higher FDS-exporting capacity of *B. thetaiotaomicron* to feed FDSs to *F. prausnitzii* (S7 Figure), which has a higher growth yield (S3 Figure). It is similar to the previous unbounded case in which an increased amount of FDSs exported by *B. thetaiotaomicron* are consumed by LAB at a higher *B. thetaiotaomicron*'s FUR. High-fiber low-carbohydrate diet interventions (i.e. 'wHF1' and 'wHF2') result in an increase in *B. thetaiotaomicron*'s abundance and a decrease in *F. prausnitzii*'s abundance given a maximum specific FUR of *B. thetaiotaomicron* higher than 5 C-mmol $gdw^{-1}h^{-1}$. As expected, increased fiber levels result in a larger abundance of *B. thetaiotaomicron,* as *B. thetaiotaomicron* is able to ferment the extra fiber available. At 3% or 5% carbohydrate availability, though the aggregate FDS export by *B. thetaiotamicron* is not strictly increasing as the maximum *B. thetaiotamicron*'s FUR increases (S7 Figure G), the specific rate of FDS export by *B. thetaiotamicron* does increase with the maximum *B. thetaiotamicron*'s FUR (S7 Figure H). This implies that for optimal community growth, each cell of *B. thetaiotamicron* will export more FDSs when it has a higher exporting capability. The drop in the required aggregate FDS export by *B. thetamicron* when *B. thetaiotamicron*'s FUR increases from 5 to 10 C-mmol $gdw^{-1}h^{-1}$ (S7 Figure G) may be the result of a higher availability of substrates other than FDS as the growth of *B. thetaiotamicron* decreases more rapidly in that region.

Systematic variation of dietary components

To systematically investigate the effect of major dietary components on the gut model, the steady-state composition was predicted under varying amounts of amino acids, carbohydrate, and dietary fiber using SteadyCom (S8 Figure). The total nutrient mass in each diet tested is equivalent to the American diet estimated in this study (See Materials and Methods). The maximum specific FUR of *B. thetaiotaomicron* was fixed at 10 C-mmol $gdw^{-1}h^{-1}$, at which the simulated shift between *B. thetaiotaomicron* and *F. prausnitzii* upon diet intervention (S7 Figure) were similar to the experimentally observed shift between Bacteroidetes and Firmicutes during alterations between the Western diet and low-fat plant-polysaccharide-rich diet in humanized gnotobiotic mice [1] and studies comparing individual human subjects [4,8,9].

A region dominated by *F. prausnitzii* with zero *B. thetaiotaomicron*'s abundance was observed at high fiber availability or low amino-acid-to-carbohydrate (AA/Carb) ratio (blue region in S8 Figure A; yellow region in S8 Figure C). Within this region, the total amino acids, which are the only nitrogen source to the community, are below 0.5 g h^{-1}, or 12 g/day. Only *F. prausnitzii* (S8 Figure C) and *E. faecalis* have significant abundances (>1%), however no defined cross feeding could be confirmed by FVA so the co-growth may be described by pure competition. Increasing the AA/Carb ratio in this region dominated by *F. prausnitzii* at a fixed fiber content increases the maximum community growth rate (S8 Figure D). Given that the total nutrient mass is constant, increased growth is caused by the increasing proportion of amino acids. At low AA/Carb ratios, *B. thetaiotaomicron*'s abundance is zero, which can be explained by the higher proportion of carbohydrates eliminating the required FDSs derived by *B. thetaiotaomicron*. At community steady-state, the growth of *B. thetaiotaomicron* is not favorable due to its low growth yield. The estimated average American diet corresponds to a dietary condition with an AA/Carb above the threshold for zero *B. thetaiotaomicron*'s abundance (the blue point in S8 Figure A). As the carbohydrate content decreases and simultaneously the fiber content increases, *B. thetaiotaomicron*'s abundance increases (the green and red points in S8 Figure A). In addition to the FDSs fed from *B. thetaiotaomicron* to *F. prausnitzii*, FVA revealed that formate, glutamate, glutamine and hydrogen sulfide are among the cross-fed metabolites in this region. By varying the amino acids, carbohydrate and dietary fiber components systematically, a region in which *B. thetaiotaomicron* interacts primarily with *E. rectale* was discovered (S8 Figure A, B). FVA revealed interspecific interactions including FDSs, hydrogen sulfide and acetate produced by *B. thetaiotaomicron* and assimilated by *E. rectale.*

Short-chain fatty acids (SCFAs) including acetate, butyrate and propionate are fermentation products of dietary fiber by the gut microbiota and have important consequences to health by affecting energy regulation, adipose tissue function, insulin sensitivity and the function of other organs [10]. Interestingly, in the region without interactions between *B. thetaiotaomicron, F. prausnitzii and E. rectale* (i.e. the region with AA/Carb ≤ 0.2) the total SCFA production can be zero (note the similarity between S8 Figure A and E). SCFA production is however nonzero in the region where *B. thetaiotaomicron* interacts with either *E. rectale* or *F. prausnitzii*. In the simulation, acetate is primarily produced by *B. thetaiotaomicron*, butyrate by *E. rectale* and propionate by *B. thetaiotaomicron* and *E. faecalis*. Moreover, in the region with SCFA production, the total SCFAs generally increase with the fiber content, which is consistent

to the comparison between human subjects with low or high levels of fiber intake [4] and the experimental results from *in vitro* culture of fecal microbiota [11].

Simulation of gut microbiota abundances using randomly sampled uptake bounds

To test the effect of adding the uptake capacity of the microbes on the prediction by SteadyCom, 1000 sets of maximum specific uptake rate for each carbon source of each species were randomly sampled. To avoid bias towards species with more substrate uptake reactions in their genome-scale models, the bounds were sampled following an exponential distribution such that the mean of the sum of the total carbon uptake by each species is 120 C-mmol $gdw^{-1}h^{-1}$, equivalent to a 20 mmol $gdw^{-1}h^{-1}$ glucose uptake. The exponential distribution was chosen based on the assumption that the number of carbon sources being assimilated at higher rates should be fewer. The simulation was repeated for another 1000 sets of random uptake bounds sampled from a uniform distribution in the interval [0, 240] such that the mean of total carbon uptake remained 120 C-mmol $gdw^{-1}h^{-1}$. Distributions with other mean values were also tested (S9 Figure). In all simulations, joint FBA tends to predict high abundances of *E. faecalis* and *S. thermophilus*, which have the highest growth yields (S3 Figure). Joint FBA also always predicts zero abundances for *B. adolescentis*, *E. coli* and *K. pneumoniae* because of their low growth yields.

Systematic variation of dietary components for a set of random uptake bounds

A set of random uptake bounds was chosen to repeat the analysis of systematically varying the contents of amino acids, fiber and carbohydrate in the diet as in the case of bounded abundances for minor species (S10 Figure). Unlike the analysis of bounding minor species presented in Figure 5, here the profile of species abundances is more diverse and most species in the community have significant abundances (>1%) under certain nutrient conditions except *L. casei*. In the current simulations, instead of constraining the community to be dominated by the proxy models for Bacteroidetes and Firmicutes as in the previous simulation, the domination by the proxy models for the two phyla in all diet compositions is predicted given the set of random uptake bounds (S10 Figure A – H). At high AA/Carb, the community is dominated by *B. thetaiotaomicron* and the two Clostridia species. *B. thetaiotaomicron* generally increases with the fiber content, similar to the observation in the previous analysis. At low AA/Carb, the community is dominated by the two lactic acid bacteria *S. thermophilus* and *E. faecalis* among the Firmicutes. For SCFA productions, the profile is also qualitatively similar to the previous analysis. SCFA productions are higher in the region dominated by *B. theta.* and the Clostridia (S8 Figure J – M). Acetate and the total SCFAs increase with the fiber content in this region coinciding the change of *B. thetaiotaomicron*'s abundance whereas butyrate and propionate appear to change in the same sense as *E. rectale*'s abundance.

References

1. Turnbaugh PJ, Ridaura VK, Faith JJ, Rey FE, Knight R, Gordon JI. The effect of diet on the human gut microbiome: a metagenomic analysis in humanized gnotobiotic mice. Sci Transl Med. 2009;1(6):6ra14.
2. Arumugam M, Raes J, Pelletier E, Le Paslier D, Yamada T, Mende DR, et al. Enterotypes of the human gut microbiome. Nature. 2011;473:174–80.
3. Claesson MJ, Jeffery IB, Conde S, Power SE, O'Connor EM, Cusack S, et al. Gut microbiota composition correlates with diet and health in the elderly. Nature. 2012;488(7410):178–84.
4. De Filippo C, Cavalieri D, Di Paola M, Ramazzotti M, Poullet JB, Massart S, et al. Impact of diet in shaping gut microbiota revealed by a comparative study in children from Europe and rural Africa. Proc Natl Acad Sci. 2010;107(33):14691–6.
5. Li J, Jia H, Cai X, Zhong H, Feng Q, Sunagawa S, et al. An integrated catalog of reference genes in the human gut microbiome. Nat Biotechnol. 2014 Jul 6;32(8):834–41.
6. Huttenhower C, Gevers D, Knight R, Abubucker S, Badger JH, Chinwalla AT, et al. Structure, function and diversity of the healthy human microbiome. Nature. 2012 Jun 13;486(7402):207–14.
7. Turnbaugh PJ, Hamady M, Yatsunenko T, Cantarel BL, Duncan A, Ley RE, et al. A core gut microbiome in obese and lean twins. Nature. 2009;457(7228):480–4.
8. Ley R, Turnbaugh P, Klein S, Gordon J. Microbial ecology: human gut microbes associated with obesity. Nature. 2006;444(7122):1022–3.
9. Turnbaugh PJ, Bäckhed F, Fulton L, Gordon JI. Diet-Induced Obesity Is Linked to Marked but Reversible Alterations in the Mouse Distal Gut Microbiome. Cell Host Microbe. 2008 Apr;3(4):213–23.
10. Canfora EE, Jocken JW, Blaak EE. Short-chain fatty acids in control of body weight and insulin sensitivity. Nat Rev Endocrinol. 2015;11(10):577–91.
11. Shen Q, Zhao L, Tuohy KM. High-level dietary fibre up-regulates colonic fermentation and relative abundance of saccharolytic bacteria within the human faecal microbiota in vitro. Eur J Nutr. 2012;51(6):693–705.
