# Supplementary material for: SteadyCom: Predicting microbial abundances while ensuring community stability
Source: PLoS Comput Biol. 2017 May 15;13(5):e1005539. doi: 10.1371/journal.pcbi.1005539 (PMC5448816; doi:10.1371/journal.pcbi.1005539)
Supplement: S1 Dataset — (ZIP) [file pcbi.1005539.s018.zip › S1 Dataset/SteadyCom/doc/SteadyCom/auxiliary_functions/checkSolFeas.html]

Description of checkSolFeas


# checkSolFeas

## PURPOSE

**Check the feasibility of a solution given a COBRA model structure or a CPLEX dynamic object and a solution**

## SYNOPSIS

**function varargout = checkSolFeas(LP, sol, maxInfeas, tol, internal)**

## DESCRIPTION

```
Check the feasibility of a solution given a COBRA model structure or a CPLEX dynamic object and a solution
infeas = checkSolFeas(LP)
   returns the maximum infeasibility 'infeas' of the solution in the Cplex 
   object 'LP'. infeas <= 0 means absolutely feasible.
infeas = checkSolFeas(LP,[],maxInfeas)
   returns the maximum infeasibility (maxInfeas = true, default) 
   or a struct of vectors of infeasibility 'infeas' (maxInfeas = false)
   of the solution in the Cplex object 'LP' with the fields:
   'con' for infeasibility of constraints
   'lb'  for infeasibility of lower bounds
   'ub'  for infeasibility of upper bounds
   'ind' for infeasibility of indicator constraints. = -Inf if an
   indicator is not active.
infeas = checkSolFeas(LP, sol, maxInfeas)
   returns a vector 'infeas' of maximum infeasibility for each solution in each 
   column of 'sol' to the LP problem 'LP', which can be a Cplex object, COBRA
   model, COBRA LP structure or gurobi LP structure if maxInfeas = true.
   If maxInfeas = false, return a structure of matrix of infeasibility 
   (e.g. #constraints x #solutions).
   'sol' can also be a solution structure resulting from gurobi or solveCobraLP
[infeas, sol] = checkSolFeas(LP, sol, maxInfeas, tol)
   returns also the solution structure 'sol' if the input 'sol' is a
   solution structure, with the field sol.stat = 1 if dev < tol, the
   feasibility tolerance. 'tol' is defaulted at 1e-8
```

## CROSS-REFERENCE INFORMATION

This function calls:

- checkSolFeas Check the feasibility of a solution given a COBRA model structure or a CPLEX dynamic object and a solution

This function is called by:

- SteadyComCplex Find the maximum community growth rate at community steady-state using SteadyCom
- SteadyComFVACplex Flux variability analysis for community model at community steady-state for a range of growth rates.
- SteadyComPOACplex Pairwise POA for community model at community steady-state for a range of growth rates
- SteadyComFVAgrCplex Flux variability analysis for community model at community steady-state at a given growth rate.
- SteadyComPOAgrCplex Pairwise POA for community model at community steady-state at a given growth rate
- checkSolFeas Check the feasibility of a solution given a COBRA model structure or a CPLEX dynamic object and a solution

## SOURCE CODE

```
0001 function varargout = checkSolFeas(LP, sol, maxInfeas, tol, internal)
0002 %Check the feasibility of a solution given a COBRA model structure or a CPLEX dynamic object and a solution
0003 %infeas = checkSolFeas(LP)
0004 %   returns the maximum infeasibility 'infeas' of the solution in the Cplex
0005 %   object 'LP'. infeas <= 0 means absolutely feasible.
0006 %infeas = checkSolFeas(LP,[],maxInfeas)
0007 %   returns the maximum infeasibility (maxInfeas = true, default)
0008 %   or a struct of vectors of infeasibility 'infeas' (maxInfeas = false)
0009 %   of the solution in the Cplex object 'LP' with the fields:
0010 %   'con' for infeasibility of constraints
0011 %   'lb'  for infeasibility of lower bounds
0012 %   'ub'  for infeasibility of upper bounds
0013 %   'ind' for infeasibility of indicator constraints. = -Inf if an
0014 %   indicator is not active.
0015 %infeas = checkSolFeas(LP, sol, maxInfeas)
0016 %   returns a vector 'infeas' of maximum infeasibility for each solution in each
0017 %   column of 'sol' to the LP problem 'LP', which can be a Cplex object, COBRA
0018 %   model, COBRA LP structure or gurobi LP structure if maxInfeas = true.
0019 %   If maxInfeas = false, return a structure of matrix of infeasibility
0020 %   (e.g. #constraints x #solutions).
0021 %   'sol' can also be a solution structure resulting from gurobi or solveCobraLP
0022 %[infeas, sol] = checkSolFeas(LP, sol, maxInfeas, tol)
0023 %   returns also the solution structure 'sol' if the input 'sol' is a
0024 %   solution structure, with the field sol.stat = 1 if dev < tol, the
0025 %   feasibility tolerance. 'tol' is defaulted at 1e-8
0026 
0027 if nargin < 3 || isempty(maxInfeas)
0028     maxInfeas = true;
0029 end
0030 if nargin < 5
0031     internal = false;
0032     %This arguement is for internal use only. Do not call it.
0033 end
0034 persistent varInd
0035 persistent compleInd
0036 persistent rhsInd
0037 persistent conInd
0038 persistent csInd
0039 if ~internal
0040     [varInd, compleInd, rhsInd, conInd, csInd] = deal([]);
0041 end
0042 if ~isa(LP, 'Cplex')
0043     %For COBRA or similar LP problem
0044     %check field in LP
0045     if isfield(LP, 'b')
0046         b0 = LP.b;
0047     elseif isfield(LP, 'rhs')
0048         b0 = LP.rhs;
0049     else
0050         varargout = {[]};
0051         return
0052     end
0053     if isfield(LP,'A') 
0054         A = LP.A; %COBRA LP problem or gurobi LP problem
0055     elseif isfield(LP,'S')
0056         A = LP.S; %COBRA model
0057     else
0058         varargout = {[]};
0059         return
0060     end
0061     if isfield(LP, 'sense')
0062         cs = LP.csense; %gurobi
0063         E = '=';
0064         L = '<';
0065         G = '>';
0066     elseif isfield(LP,'csense')
0067         cs = LP.sense; %COBRA
0068         E = 'E';
0069         L = 'L';
0070         G = 'G';
0071     else
0072         %COBRA model, no csense, assume all equal
0073         cs = char('E' * ones(size(A,1),1));
0074         E = 'E';
0075         L = 'L';
0076         G = 'G';
0077     end
0078    %check field in sol
0079     if isstruct(sol)
0080         sol0 = sol;
0081         if isfield(sol,'full') 
0082             sol = sol.full;
0083         elseif isfield(sol, 'x')
0084             sol = sol.x;
0085         else
0086             varargout = {[]};
0087             return
0088         end
0089     elseif ismatrix(sol) && size(sol,1) == size(A,2)
0090         sol = sol;
0091     else
0092         varargout = {[]};
0093         return
0094     end
0095     
0096     b = A * sol;
0097     b0 = b0 * ones(1, size(sol,2));
0098     if maxInfeas
0099         infeas = zeros(1,size(sol,2));
0100         infeas = max([infeas; max(abs(b(cs == E,:) - b0(cs == E,:)),[],1)],[],1);
0101         infeas = max([infeas; max(b(cs == L,:) - b0(cs == L,:),[],1)], [], 1);
0102         infeas = max([infeas; max(b0(cs == G,:) - b(cs == G,:),[],1)], [], 1);
0103         if isfield(LP,'lb')
0104             infeas = max([infeas; max(LP.lb*ones(1,size(sol,2)) - sol, [],1)], [], 1);
0105         end
0106         if isfield(LP,'ub')
0107             infeas = max([infeas; max(sol - LP.ub*ones(1,size(sol,2)), [],1)], [], 1);
0108         end
0109     else
0110         infeas.con = zeros(size(A,1),size(sol,2));
0111         infeas.con(cs == E,:) = abs(b(cs == E,:) - b0(cs == E,:));
0112         infeas.con(cs == L,:) = b(cs == L,:) - b0(cs == L,:);
0113         infeas.con(cs == G,:) = b0(cs == G,:) - b(cs == G,:);
0114         infeas.con(infeas.con < 0) = 0;
0115         if isfield(LP,'lb')
0116             infeas.lb = LP.lb*ones(1,size(sol,2)) - sol;
0117             infeas.lb(infeas.lb < 0) = 0;
0118         else
0119             infeas.lb = [];
0120         end
0121         if isfield(LP,'ub')
0122             infeas.ub = sol - LP.ub*ones(1,size(sol,2));
0123             infeas.ub(infeas.ub < 0) = 0;
0124         else
0125             infeas.ub = [];
0126         end
0127     end
0128         
0129     if isstruct(sol0)
0130         if nargin < 4
0131             tol = 1e-8;
0132         end
0133         if infeas < tol
0134             sol0.stat = 1;
0135         end
0136         varargout = {infeas, sol0};
0137     else
0138         varargout = {infeas};
0139     end
0140 else
0141     %For Cplex object
0142     if nargin < 2 || isempty(sol)
0143         %check the solution in the Cplex object if no solution input
0144         if isprop(LP,'Solution') && isfield(LP.Solution,'x') && ~isempty(LP.Solution.x)
0145             sol = LP.Solution.x;
0146         else
0147             varargout = {NaN};
0148             return
0149         end
0150     end
0151  
0152     %for checking an array of solutions
0153     %(Each column in the matrix sol is a solution)
0154     mSize = 30; %optimized parameter for my computer for models of large size (~1e4)
0155     %10 ~ 100 are good
0156     if isfield(LP.Model,'indicator') && ~internal
0157         [varInd, compleInd, rhsInd] = deal(zeros(numel(LP.Model.indicator),1));
0158         conInd = zeros(numel(LP.Model.indicator),size(LP.Model.A,2));
0159         csInd = char('E'*ones(1,numel(LP.Model.indicator)));
0160         for j = 1:numel(LP.Model.indicator)
0161             varInd(j) = LP.Model.indicator(j).variable;
0162             compleInd(j) = LP.Model.indicator(j).complemented;
0163             conInd(j,:) = LP.Model.indicator(j).a';
0164             rhsInd(j) = LP.Model.indicator(j).rhs;
0165             csInd(j) = LP.Model.indicator(j).sense;
0166         end
0167         
0168     end
0169     b = LP.Model.A * sol;
0170     
0171     if maxInfeas
0172         infeas = zeros(1,size(sol, 2));
0173         if size(sol, 2) > mSize
0174             %compute mSize solutions each time
0175             for j = 1:floor(size(sol, 2) / mSize)
0176                 infeas(((j-1)*mSize + 1):(j*mSize)) = checkSolFeas(LP, ...
0177                     sol(:,((j-1)*mSize + 1):(j*mSize)),maxInfeas,[],true);
0178             end
0179             %compute the remaining solutions
0180             if mod(size(sol, 2), mSize) > 0
0181                 infeas((floor(size(sol, 2) / mSize)*mSize+1):end) = checkSolFeas(LP, ...
0182                     sol(:,(floor(size(sol, 2) / mSize)*mSize+1):end),maxInfeas,[],true);
0183             end
0184             
0185         else
0186             infeas = max([infeas; max(repmat(LP.Model.lhs,1,size(b,2)) - b,[],1)],[],1);
0187             infeas = max([infeas; max(b - repmat(LP.Model.rhs,1,size(b,2)),[],1)],[],1);
0188             infeas = max([infeas; max(repmat(LP.Model.lb,1,size(sol,2)) - sol,[],1)],[],1);
0189             infeas = max([infeas; max(sol - repmat(LP.Model.ub,1,size(sol,2)),[],1)],[],1);
0190             if isfield(LP.Model,'indicator')
0191                 compleInd2 = compleInd * ones(1, size(sol,2));
0192                 bInd = (conInd * sol) .* ...
0193                     (sol(varInd,:) > 0.9 & compleInd2 == 0 | ... %active indicator
0194                     sol(varInd,:) < 1e-2 & compleInd2 == 1);
0195                 b0Ind = (rhsInd * ones(1, size(sol,2))) .* ...
0196                     (sol(varInd,:) > 0.9 & compleInd2 == 0 | ... %active indicator
0197                     sol(varInd,:) < 1e-2 & compleInd2 == 1);
0198                 infeas = max([infeas; max(abs(bInd(csInd == 'E',:) - b0Ind(csInd == 'E',:)),[],1)],[],1);
0199                 infeas = max([infeas; max(bInd(csInd == 'L',:) - b0Ind(csInd == 'L',:),[],1)], [], 1);
0200                 infeas = max([infeas; max(b0Ind(csInd == 'G',:) - bInd(csInd == 'G',:),[],1)], [], 1);
0201             end
0202         end
0203     else
0204         infeas =struct();
0205         [infeas.con, infeas.lb, infeas.ub, infeas.ind] = deal([]);
0206         list = {'con','lb','ub','ind'};
0207         if size(sol, 2) > mSize
0208             %compute mSize solutions each time
0209             for j = 1:floor(size(sol, 2) / mSize)
0210                 infeasJ = checkSolFeas(LP, ...
0211                     sol(:,((j-1)*mSize + 1):(j*mSize)),maxInfeas,[],true);
0212                 for k = list
0213                     infeas.(k{:}) = [infeas.(k{:}), infeasJ.(k{:})];
0214                 end
0215             end
0216             %compute the remaining solutions
0217             if mod(size(sol, 2), mSize) > 0
0218                 infeasJ = checkSolFeas(LP, ...
0219                     sol(:,(floor(size(sol, 2) / mSize)*mSize+1):end),maxInfeas,[],true);
0220                 for k = list
0221                     infeas.(k{:}) = [infeas.(k{:}), infeasJ.(k{:})];
0222                 end
0223             end
0224         else
0225             infeas.con = repmat(LP.Model.lhs,1,size(b,2)) - b;
0226             infeas.con = max(infeas.con, b - repmat(LP.Model.rhs,1,size(b,2)));
0227             infeas.con(infeas.con < 0) = 0;
0228             infeas.lb = repmat(LP.Model.lb,1,size(sol,2)) - sol;
0229             infeas.lb(infeas.lb < 0) = 0;
0230             infeas.ub = sol - repmat(LP.Model.ub,1,size(sol,2));
0231             infeas.ub(infeas.ub < 0) = 0;
0232             if isfield(LP.Model,'indicator')
0233                 compleInd2 = compleInd * ones(1, size(sol,2));
0234                 bInd = (conInd * sol);
0235                 b0Ind = rhsInd * ones(1, size(sol,2));
0236                 infeas.ind = zeros(size(conInd,1), size(sol,2));
0237                 infeas.ind(csInd == 'E',:) = abs(bInd(csInd == 'E',:) - b0Ind(csInd == 'E',:));
0238                 infeas.ind(csInd == 'L',:) = bInd(csInd == 'L',:) - b0Ind(csInd == 'L',:);
0239                 infeas.ind(csInd == 'G',:) = b0Ind(csInd == 'G',:) - bInd(csInd == 'G',:);
0240                 infeas.ind(infeas.ind < 0) = 0;
0241                 infeas.ind(~((sol(varInd,:) > 0.9 & compleInd2 == 0) | ... %active indicator
0242                     (sol(varInd,:) < 1e-2 & compleInd2 == 1))) = -inf;
0243             else
0244                 infeas.ind = [];
0245             end
0246         end
0247     end
0248     varargout = {infeas};
0249 end
0250 
0251 end
0252
```

---

Generated on Sat 06-May-2017 09:55:30 by **m2html** © 2005
