# Supplementary material for: SteadyCom: Predicting microbial abundances while ensuring community stability
Source: PLoS Comput Biol. 2017 May 15;13(5):e1005539. doi: 10.1371/journal.pcbi.1005539 (PMC5448816; doi:10.1371/journal.pcbi.1005539)
Supplement: S1 Dataset — (ZIP) [file pcbi.1005539.s018.zip › S1 Dataset/SteadyCom/doc/SteadyCom/auxiliary_functions/getCobraComParams.html]

Description of getCobraComParams


# getCobraComParams

## PURPOSE

**get the required default parameters**

## SYNOPSIS

**function varargout = getCobraComParams(param2get, options, modelCom)**

## DESCRIPTION

```
get the required default parameters
[param_1, ..., param_N] = getCobraComParams({'param_1',...,'param_N'},options,modelCom)
Input:
   'param_1',...,'param_N': parameter names
   options: option structure
            If the required parameter is a field in options, take from
            options. Otherwise, return the default value.
   modelCom: the community model for which parameters are constructed.
```

## CROSS-REFERENCE INFORMATION

This function calls:


This function is called by:

- SteadyComCplex Find the maximum community growth rate at community steady-state using SteadyCom
- SteadyComFVACplex Flux variability analysis for community model at community steady-state for a range of growth rates.
- SteadyComPOACplex Pairwise POA for community model at community steady-state for a range of growth rates
- SteadyComFVAgrCplex Flux variability analysis for community model at community steady-state at a given growth rate.
- SteadyComPOAgrCplex Pairwise POA for community model at community steady-state at a given growth rate
- updateLPcom Create and update the SteadyCom LP model in CPLEX format.
- createCommModel Create a community COBRA model. The model has an extra compartment [u]

## SUBFUNCTIONS

- function param = paramDefault(paramName,modelCom)
- function x = transformOptionInput(options, field, nSp)

## SOURCE CODE

```
0001 function varargout = getCobraComParams(param2get, options, modelCom)
0002 %get the required default parameters
0003 %[param_1, ..., param_N] = getCobraComParams({'param_1',...,'param_N'},options,modelCom)
0004 %Input:
0005 %   'param_1',...,'param_N': parameter names
0006 %   options: option structure
0007 %            If the required parameter is a field in options, take from
0008 %            options. Otherwise, return the default value.
0009 %   modelCom: the community model for which parameters are constructed.
0010    
0011 if nargin < 3
0012     modelCom = struct('rxns',[]);
0013     modelCom.infoCom.spAbbr = {};
0014     modelCom.infoCom.rxnSps = {};
0015 end
0016 if nargin < 2 || isempty(options)
0017     options = struct();
0018 end
0019 if ischar(param2get)
0020     param2get = {param2get};
0021 end
0022 paramNeedTransform = {'GRfx', 'BMlb', 'BMub', 'BMfx'};
0023     
0024 varargout = cell(numel(param2get), 1);
0025 for j = 1:numel(param2get)
0026     if any(strcmp(param2get{j}, paramNeedTransform))
0027         %if need transformation
0028         varargout{j} = transformOptionInput(options, param2get{j}, numel(modelCom.infoCom.spAbbr));
0029     elseif isfield(options, param2get{j})
0030         %if provided in the call
0031         varargout{j} = options.(param2get{j});
0032     else
0033         %use default if default exist and not provided
0034         %return empty if no default
0035         varargout{j} = paramDefault(param2get{j}, modelCom);
0036     end
0037     %if calling for a directory, make sure to return a new directory
0038     if strcmp(param2get{j}, 'directory')
0039         k = 0;
0040         while exist(varargout{j}, 'file')
0041             k = k + 1;
0042             varargout{j} = [paramDefault.directory num2str(k)];
0043         end
0044     end
0045 end
0046 
0047 
0048 end
0049 
0050 function param = paramDefault(paramName,modelCom)
0051 
0052 switch paramName
0053     %general parameters
0054     case 'threads',     param = 1; %threads for general computation, 0 or -1 to turn on maximum no. of threads
0055     case 'verbFlag',    param = 3;%verbal dispaly
0056     case 'loadModel',   param = '';
0057     case 'CplexParam',  %default Cplex parameter structure
0058         [param.simplex.display, param.tune.display, param.barrier.display,...
0059             param.sifting.display, param.conflict.display] = deal(0);
0060         [param.simplex.tolerances.optimality, param.simplex.tolerances.feasibility] = deal(1e-9,1e-8);
0061         param.read.scale = -1;
0062         
0063     %parameters for createCommModel
0064     case 'metExId',     param = '[e]';
0065         
0066     %parameters for SteadyComCplex
0067     case 'GRguess',     param = 0.2;%initial guess for growth rate
0068     case 'BMtol',       param = 0.8;%tolerance for relative biomass amount (used only for feasCrit=3)
0069     case 'BMtolAbs',    param = 1e-5;%tolerance for absolute biomass amount
0070     case 'GR0',         param = 0.001;%small growth rate to test growth
0071     case 'GRtol',       param = 1e-5;%gap for growth rate convergence
0072     case 'GRdev',       param = 1e-5;%percentage deviation from the community steady state allowed
0073     case 'maxIter',     param = 1e3;%maximum no. of iterations
0074     case 'feasCrit',    param = 1; %feasibility critierion
0075     case 'algorithm',   param = 1;%1:invoke Fzero after getting bounds; 2:simple guessing algorithm
0076     case 'BMgdw',       param = ones(numel(modelCom.infoCom.spAbbr), 1);%relative molecular weight of biomass. For scaling the relative abundance
0077     case 'saveModel',   param = '';
0078     case 'BMobj',       param = ones(numel(modelCom.infoCom.spBm),1); %objective coefficient for each species
0079     case 'BMweight',    param = 1; %sum of biomass for feasibility criterion 1
0080     case 'LPonly',      param = false;%true to return LP only but not calculate anything
0081     case 'solveGR0',    param = false;%true to solve the model at very low growth rate (GR0)
0082     case 'resultTmp',   param = struct('GRmax',[],'vBM',[],'BM',[],'Ut',[],...
0083                                 'Ex',[],'flux',[],'iter0',[],'iter',[],'stat','');%result template
0084     %parameters for SteadyComFVACplex
0085     case 'optBMpercent',param = 99.99;
0086     case 'rxnNameList', if isfield(modelCom.infoCom,'spBm'), param = modelCom.rxns(findRxnIDs(modelCom,modelCom.infoCom.spBm));else param = modelCom.rxns;end
0087     case 'rxnFluxList', if isfield(modelCom.infoCom,'spBm'), param = modelCom.rxns(findRxnIDs(modelCom,modelCom.infoCom.spBm));else param = modelCom.rxns;end
0088     case 'BMmaxLB',     param = 1; %maximum biomass when it is unknown
0089     case 'BMmaxUB',     param = 1; %maximum biomass when it is unknown
0090     case 'optGRpercent',param = 99.99;
0091     case 'saveFVA',     param = '';
0092     
0093     %parameters for SteadyComPOACplex
0094     case 'Nstep',       param = 10;
0095     case 'NstepScale',  param = 'lin';
0096     case 'symmetric',   param = true; %treat it as symmetric, optimize for only j > k
0097     case 'savePOA',     param = 'POAtmp/POA';
0098    
0099     otherwise,          param = [];
0100 end
0101 end
0102 
0103 function x = transformOptionInput(options, field, nSp)
0104 if isfield(options, field)
0105     if size(options.(field), 2) == 2
0106         x = NaN(nSp, 1);
0107         x(options.(field)(:,1)) = options.(field)(:,2);
0108     else
0109         x = options.(field);
0110     end
0111 else
0112     x = NaN(nSp, 1);
0113 end
0114 
0115 end
```

---

Generated on Sat 06-May-2017 09:55:30 by **m2html** © 2005
