# Supplementary material for: SteadyCom: Predicting microbial abundances while ensuring community stability
Source: PLoS Comput Biol. 2017 May 15;13(5):e1005539. doi: 10.1371/journal.pcbi.1005539 (PMC5448816; doi:10.1371/journal.pcbi.1005539)
Supplement: S1 Dataset — (ZIP) [file pcbi.1005539.s018.zip › S1 Dataset/SteadyCom/doc/SteadyCom/auxiliary_functions/infoCom2indCom.html]

Description of infoCom2indCom


# infoCom2indCom

## PURPOSE

**Transform between community reaction IDs and reaction names**

## SYNOPSIS

**function indCom = infoCom2indCom(modelCom,infoCom,revFlag,spAbbr,spName)**

## DESCRIPTION

```
Transform between community reaction IDs and reaction names

indCom = infoCom2indCom(modelCom)
   Get the IDs from names. modelCom must have .infoCom
indCom = infoCom2indCom(modelCom, infoCom)
   Supply infoCom if it is not a field of modelCom
infoCom = infoCom2indCom(modelCom,indCom,true,spAbbr,spName)
   Get the name structure infoCom from ID structure indCom
   spAbbr (must be supplied if revFlag = true) : the abbreviation of each organism
   spName (optional): the name of each organism
```

## CROSS-REFERENCE INFORMATION

This function calls:


This function is called by:

- SteadyComCplex Find the maximum community growth rate at community steady-state using SteadyCom
- SteadyComFVACplex Flux variability analysis for community model at community steady-state for a range of growth rates.
- SteadyComPOACplex Pairwise POA for community model at community steady-state for a range of growth rates
- SteadyComFVAgrCplex Flux variability analysis for community model at community steady-state at a given growth rate.
- SteadyComPOAgrCplex Pairwise POA for community model at community steady-state at a given growth rate
- createCommModel Create a community COBRA model. The model has an extra compartment [u]

## SOURCE CODE

```
0001 function indCom = infoCom2indCom(modelCom,infoCom,revFlag,spAbbr,spName)
0002 %Transform between community reaction IDs and reaction names
0003 %
0004 %indCom = infoCom2indCom(modelCom)
0005 %   Get the IDs from names. modelCom must have .infoCom
0006 %indCom = infoCom2indCom(modelCom, infoCom)
0007 %   Supply infoCom if it is not a field of modelCom
0008 %infoCom = infoCom2indCom(modelCom,indCom,true,spAbbr,spName)
0009 %   Get the name structure infoCom from ID structure indCom
0010 %   spAbbr (must be supplied if revFlag = true) : the abbreviation of each organism
0011 %   spName (optional): the name of each organism
0012 if nargin < 2
0013     if ~isfield(modelCom,'infoCom')
0014         error('infoCom must be provided.\n');
0015     end
0016     infoCom = modelCom.infoCom;
0017 end
0018 if nargin < 3
0019     revFlag = false;
0020 end
0021 indCom = struct();
0022 if ~revFlag
0023     %from infoCom to indCom
0024     indCom.spBm = findRxnIDs(modelCom,infoCom.spBm);
0025     if isfield(infoCom,'spATPM')
0026         indCom.spATPM = findRxnIDs(modelCom,infoCom.spATPM);
0027     end
0028     if isfield(infoCom,'rxnSD')
0029         indCom.rxnSD = findRxnIDs(modelCom,infoCom.rxnSD);
0030     end
0031     indCom.EXcom = findRxnIDs(modelCom,infoCom.EXcom);
0032     indCom.EXsp = zeros(size(infoCom.EXsp));
0033     SpCom = ~cellfun(@isempty,infoCom.EXsp);
0034     indCom.EXsp(SpCom) = findRxnIDs(modelCom,infoCom.EXsp(SpCom));
0035     indCom.Mcom = findMetIDs(modelCom,infoCom.Mcom);
0036     indCom.Msp = zeros(size(infoCom.Msp));
0037     SpCom = ~cellfun(@isempty,infoCom.Msp);
0038     indCom.Msp(SpCom) = findMetIDs(modelCom,infoCom.Msp(SpCom));
0039     [~,indCom.rxnSps] = ismember(infoCom.rxnSps,infoCom.spAbbr);
0040     [~,indCom.metSps] = ismember(infoCom.metSps,infoCom.spAbbr);
0041 else
0042     %from indCom to infoCom
0043     if nargin < 4
0044         error('spAbbr must be provided to get the organisms'' abbreviations');
0045     end
0046     if nargin < 5
0047         spName = spAbbr;
0048     end
0049     indCom.spBm = modelCom.rxns(infoCom.spBm);
0050     if isfield(infoCom,'spATPM')
0051         indCom.spATPM = modelCom.rxns(infoCom.spATPM);
0052     end
0053     if isfield(infoCom,'rxnSD')
0054         indCom.rxnSD = modelCom.rxns(infoCom.rxnSD);
0055     end
0056     indCom.EXcom = repmat({''},size(infoCom.EXcom,1),2);
0057     indCom.EXcom(infoCom.EXcom~=0) = modelCom.rxns(infoCom.EXcom(infoCom.EXcom~=0));
0058     indCom.EXsp = repmat({''},size(infoCom.EXsp,1),size(infoCom.EXsp,2));
0059     SpCom = infoCom.EXsp ~= 0;
0060     indCom.EXsp(SpCom) = modelCom.rxns(infoCom.EXsp(SpCom));
0061     indCom.Mcom = modelCom.mets(infoCom.Mcom);
0062     indCom.Msp = repmat({''},size(infoCom.Msp,1),size(infoCom.Msp,2));
0063     SpCom = infoCom.Msp ~= 0;
0064     indCom.Msp(SpCom) = modelCom.mets(infoCom.Msp(SpCom));
0065     indCom.spAbbr = spAbbr;
0066     indCom.spName = spName;
0067     indCom.rxnSps = repmat({'com'},numel(modelCom.rxns),1);
0068     indCom.rxnSps(infoCom.rxnSps > 0) = spAbbr(infoCom.rxnSps(infoCom.rxnSps > 0));
0069     indCom.metSps = repmat({'com'},numel(modelCom.mets),1);
0070     indCom.metSps(infoCom.metSps > 0) = spAbbr(infoCom.metSps(infoCom.metSps > 0));
0071 end
0072 end
```

---

Generated on Sat 06-May-2017 09:55:30 by **m2html** © 2005
