# Supplementary material for: SteadyCom: Predicting microbial abundances while ensuring community stability
Source: PLoS Comput Biol. 2017 May 15;13(5):e1005539. doi: 10.1371/journal.pcbi.1005539 (PMC5448816; doi:10.1371/journal.pcbi.1005539)
Supplement: S1 Dataset — (ZIP) [file pcbi.1005539.s018.zip › S1 Dataset/SteadyCom/doc/SteadyCom/auxiliary_functions/menu.html]

Index for Directory SteadyCom/auxiliary\_functions


 Master index 

# Index for SteadyCom/auxiliary\_functions

## Matlab files in this directory:

- SteadyComFVAgrCplex
- SteadyComPOAgrCplex
- checkSolFeas
- getCobraComParams
- infoCom2indCom
- setCplexParam
- updateLPcom

---

Generated by **m2html** © 2005
