# Supplementary material for: SteadyCom: Predicting microbial abundances while ensuring community stability
Source: PLoS Comput Biol. 2017 May 15;13(5):e1005539. doi: 10.1371/journal.pcbi.1005539 (PMC5448816; doi:10.1371/journal.pcbi.1005539)
Supplement: S1 Dataset — (ZIP) [file pcbi.1005539.s018.zip › S1 Dataset/SteadyCom/doc/SteadyCom/auxiliary_functions/setCplexParam.html]

Description of setCplexParam


# setCplexParam

## PURPOSE

**Set the parameters of the CPLEX object according to the structure solverParam**

## SYNOPSIS

**function LP = setCplexParam(LP, solverParam,verbFlag)**

## DESCRIPTION

```
Set the parameters of the CPLEX object according to the structure solverParam
For example:
[solverParam.simplex.display, solverParam.tune.display, solverParam.barrier.display,...
     solverParam.sifting.display, solverParam.conflict.display] = deal(0);
[solverParam.simplex.tolerances.optimality, solverParam.simplex.tolerances.feasibility] = deal(1e-9,1e-8);
```

## CROSS-REFERENCE INFORMATION

This function calls:


This function is called by:

- SteadyComCplex Find the maximum community growth rate at community steady-state using SteadyCom
- SteadyComFVACplex Flux variability analysis for community model at community steady-state for a range of growth rates.
- SteadyComPOACplex Pairwise POA for community model at community steady-state for a range of growth rates
- SteadyComFVAgrCplex Flux variability analysis for community model at community steady-state at a given growth rate.
- SteadyComPOAgrCplex Pairwise POA for community model at community steady-state at a given growth rate

## SUBFUNCTIONS

- function [paramList, paramPath] = getParamList(param, bottomFlag)

## SOURCE CODE

```
0001 function LP = setCplexParam(LP, solverParam,verbFlag)
0002 %Set the parameters of the CPLEX object according to the structure solverParam
0003 %For example:
0004 %[solverParam.simplex.display, solverParam.tune.display, solverParam.barrier.display,...
0005 %     solverParam.sifting.display, solverParam.conflict.display] = deal(0);
0006 %[solverParam.simplex.tolerances.optimality, solverParam.simplex.tolerances.feasibility] = deal(1e-9,1e-8);
0007 if nargin < 3
0008     verbFlag = true;
0009 end
0010 if isempty(fieldnames(solverParam))
0011     return
0012 end
0013 [paramList, paramPath] = getParamList(LP.Param, 0);
0014 [paramUserList, paramUserPath] = getParamList(solverParam, 1);
0015 paramIden = false(numel(paramUserList), 1);
0016 for p = 1:numel(paramUserList)
0017     f = strcmpi(paramList,paramUserList{p});
0018     if sum(f) == 1
0019         paramIden(p) = true;
0020         str = ['LP.Param.' paramPath{f} '.Cur = solverParam.' paramUserPath{p} ';'];
0021         eval(str);
0022     elseif sum(f) > 1
0023         if ismember(lower(paramUserPath{p}), paramPath);
0024             paramIden(p) = true;
0025             str = ['LP.Param.' lower(paramUserPath{p}) '.Cur = solverParam.' paramUserPath{p} ';'];
0026             eval(str);
0027         else
0028             if verbFlag
0029                 fprintf('solverParam.%s cannot be uniquely identified as a valid cplex parameter. Ignore.\n', paramUserPath{p});
0030             end
0031         end
0032     else
0033         if verbFlag
0034             fprintf('solverParam.%s cannot be identified as a valid cplex parameter. Ignore.\n', paramUserPath{p});
0035         end
0036     end
0037 end
0038 end
0039 
0040 function [paramList, paramPath] = getParamList(param, bottomFlag)
0041 %for matching CPLEX parameters appropriately
0042 structCur = param;
0043 lv = 1;
0044 lvFieldN = zeros(10,1);
0045 lvFieldN(1) = 1;
0046 lvField = cell(10, 1);
0047 lvField{lv} = fieldnames(structCur);
0048 paramPath = {};
0049 paramList = {};
0050 while lv > 0
0051     if isstruct(structCur.(lvField{lv}{lvFieldN(lv)})) 
0052         if ~isempty(fieldnames(structCur.(lvField{lv}{lvFieldN(lv)})))
0053             structCur = structCur.(lvField{lv}{lvFieldN(lv)});
0054             lv = lv + 1;
0055             lvFieldN(lv) = 1;
0056             lvField{lv} = fieldnames(structCur);
0057         else
0058             while lvFieldN(lv) == numel(lvField{lv})
0059                 lv = lv - 1;
0060                 if lv == 0
0061                     break
0062                 end
0063             end
0064             if lv > 0
0065                 lvFieldN(lv) = lvFieldN(lv) + 1;
0066                 structCur = param;
0067                 for j = 1:lv-1
0068                     structCur = structCur.(lvField{j}{lvFieldN(j)});
0069                 end
0070             end
0071         end
0072     else
0073         if ~bottomFlag
0074             lv = lv - 1;
0075         end
0076         if lv > 0
0077             c = {};
0078             for j = 1:lv
0079                 c = [c lvField{j}(lvFieldN(j))];
0080             end
0081             paramPath = [paramPath; strjoin(c,'.')];
0082             paramList = [paramList; c(end)];
0083             while lvFieldN(lv) == numel(lvField{lv})
0084                 lv = lv - 1;
0085                 if lv == 0
0086                     break
0087                 end
0088             end
0089             if lv > 0
0090                 lvFieldN(lv) = lvFieldN(lv) + 1;
0091                 structCur = param;
0092                 for j = 1:lv-1
0093                     structCur = structCur.(lvField{j}{lvFieldN(j)});
0094                 end
0095             end
0096         else
0097             lv = 1;
0098             if lvFieldN(lv) == numel(lvField{lv})
0099                 break
0100             else
0101                 lvFieldN(1) = lvFieldN(1) + 1;
0102             end
0103         end
0104     end
0105 end
0106 
0107 end
```

---

Generated on Sat 06-May-2017 09:55:30 by **m2html** © 2005
