# Supplementary material for: SteadyCom: Predicting microbial abundances while ensuring community stability
Source: PLoS Comput Biol. 2017 May 15;13(5):e1005539. doi: 10.1371/journal.pcbi.1005539 (PMC5448816; doi:10.1371/journal.pcbi.1005539)
Supplement: S1 Dataset — (ZIP) [file pcbi.1005539.s018.zip › S1 Dataset/SteadyCom/doc/SteadyCom/auxiliary_functions/SteadyComFVAgrCplex.html]

Description of SteadyComFVAgrCplex


# SteadyComFVAgrCplex

## PURPOSE

**Flux variability analysis for community model at community steady-state at a given growth rate.**

## SYNOPSIS

**function [minFlux,maxFlux,minFD,maxFD,LP,GR] = SteadyComFVAgrCplex(modelCom,options,solverParam,LP)**

## DESCRIPTION

```
Flux variability analysis for community model at community steady-state at a given growth rate. 
The function is capable of saving intermediate results and continuing from previous results 
if the file path is given in options.saveFVA. It also allows switch from single thread to parallel 
computation from intermediate results (but not the reverse).

[minFlux,maxFlux,minFD,maxFD,LP,GR] = SteadyComFVAgrCplex(modelCom,options,solverParam,LP)

INPUT
 modelCom        a community COBRA model structure with the following extra fields:
 (the following fields are required - others can be supplied)
   S            Stoichiometric matrix
   b            Right hand side
   c            Objective coefficients
   lb           Lower bounds
   ub           Upper bounds
 (at least one of the below two is needed)
   infoCom      structure containing community reaction info 
                (returned along with the community model created with createCommModel)
   indCom       the index structure corresponding to infoCom

 options (optional) option structure with the following fields:
   GR              The growth rate at which FVA is performed. If not
                     given, find the maximum growth rate by SteadyComCplex.m
   optBMpercent    Only consider solutions that yield at least a certain
                     percentage of the optimal biomass (Default = 99.99)
   rxnNameList     List of reactions (IDs or .rxns) for which FVA is performed.
                     Use a (N_rxns + N_organism) x K matrix for FVA of K
                     linear combinations of fluxes and/or abundances
                     (Default = biomass reaction of each species)
   rxnFluxList     List of reactions (IDs or .rxns) whose fluxes are also returned
                     (Default = biomass reaction of each species)
  (the two parameters below are usually determined by solving the problem
      during the program. Provide them only if you want to constrain the
      total biomass to a particular value)
   BMmaxLB         lower bound for the total biomass (default 1)
   BMmaxUB         upper bound for the total biomass
  (other parameters)
   saveFVA         If non-empty, become the filename to save the FVA results
                   (default empty, not saving)
   threads         > 1 for explicitly stating the no. of threads used,
                   0 or -1 for using all available threads. Default 1.
   verbFlag        Verbose output. 1 to have waitbar, >1 to have stepwise output
                   (default 3)
   loadModel       String of filename to be loaded. If non-empty, load the 
                   cplex model ('loadModel.mps'), basis ('loadModel.bas') 
                   and parameters ('loadModel.prm').
  May add also other parameters in SteadyComCplex for calculating the maximum growth rate.

 solverParam       Cplex parameter structure. E.g., struct('simplex',struct('tolerances',struct('feasibility',1e-8)))
 LP                Cplex LP object from, e.g., calling FVAComCplex

OUTPUT
 minFlux       Minimum flux for each reaction
 maxFlux       Maximum flux for each reaction

OPTIONAL OUTPUT
 minFD         #rxnFluxList x #rxnNameList matrix containing the fluxes in
               options.rxnFluxList corresponding to minimizing each reaction in
               options.rxnNameList
 maxFD         #rxnFluxList x #rxnNameList matrix containing the fluxes in
               options.rxnFluxList corresponding to maximizing each reaction in
               options.rxnNameList
 LP            Cplex LP object
 GR            the growth rate at which FVA is performed
```

## CROSS-REFERENCE INFORMATION

This function calls:

- SteadyComCplex Find the maximum community growth rate at community steady-state using SteadyCom
- checkSolFeas Check the feasibility of a solution given a COBRA model structure or a CPLEX dynamic object and a solution
- getCobraComParams get the required default parameters
- infoCom2indCom Transform between community reaction IDs and reaction names
- setCplexParam Set the parameters of the CPLEX object according to the structure solverParam
- updateLPcom Create and update the SteadyCom LP model in CPLEX format.

This function is called by:

- SteadyComFVACplex Flux variability analysis for community model at community steady-state for a range of growth rates.
- SteadyComPOAgrCplex Pairwise POA for community model at community steady-state at a given growth rate

## SUBFUNCTIONS

- function parSave(minFluxP,maxFluxP,minFDP,maxFDP,i0,jP,saveFVA)
- function [minFluxP,maxFluxP,minFDP,maxFDP,i0] = parLoad(jP,saveFVA)

## SOURCE CODE

```
0001 function [minFlux,maxFlux,minFD,maxFD,LP,GR] = SteadyComFVAgrCplex(modelCom,options,solverParam,LP)
0002 %Flux variability analysis for community model at community steady-state at a given growth rate.
0003 %The function is capable of saving intermediate results and continuing from previous results
0004 %if the file path is given in options.saveFVA. It also allows switch from single thread to parallel
0005 %computation from intermediate results (but not the reverse).
0006 %
0007 %[minFlux,maxFlux,minFD,maxFD,LP,GR] = SteadyComFVAgrCplex(modelCom,options,solverParam,LP)
0008 %
0009 %INPUT
0010 % modelCom        a community COBRA model structure with the following extra fields:
0011 % (the following fields are required - others can be supplied)
0012 %   S            Stoichiometric matrix
0013 %   b            Right hand side
0014 %   c            Objective coefficients
0015 %   lb           Lower bounds
0016 %   ub           Upper bounds
0017 % (at least one of the below two is needed)
0018 %   infoCom      structure containing community reaction info
0019 %                (returned along with the community model created with createCommModel)
0020 %   indCom       the index structure corresponding to infoCom
0021 %
0022 % options (optional) option structure with the following fields:
0023 %   GR              The growth rate at which FVA is performed. If not
0024 %                     given, find the maximum growth rate by SteadyComCplex.m
0025 %   optBMpercent    Only consider solutions that yield at least a certain
0026 %                     percentage of the optimal biomass (Default = 99.99)
0027 %   rxnNameList     List of reactions (IDs or .rxns) for which FVA is performed.
0028 %                     Use a (N_rxns + N_organism) x K matrix for FVA of K
0029 %                     linear combinations of fluxes and/or abundances
0030 %                     (Default = biomass reaction of each species)
0031 %   rxnFluxList     List of reactions (IDs or .rxns) whose fluxes are also returned
0032 %                     (Default = biomass reaction of each species)
0033 %  (the two parameters below are usually determined by solving the problem
0034 %      during the program. Provide them only if you want to constrain the
0035 %      total biomass to a particular value)
0036 %   BMmaxLB         lower bound for the total biomass (default 1)
0037 %   BMmaxUB         upper bound for the total biomass
0038 %  (other parameters)
0039 %   saveFVA         If non-empty, become the filename to save the FVA results
0040 %                   (default empty, not saving)
0041 %   threads         > 1 for explicitly stating the no. of threads used,
0042 %                   0 or -1 for using all available threads. Default 1.
0043 %   verbFlag        Verbose output. 1 to have waitbar, >1 to have stepwise output
0044 %                   (default 3)
0045 %   loadModel       String of filename to be loaded. If non-empty, load the
0046 %                   cplex model ('loadModel.mps'), basis ('loadModel.bas')
0047 %                   and parameters ('loadModel.prm').
0048 %  May add also other parameters in SteadyComCplex for calculating the maximum growth rate.
0049 %
0050 % solverParam       Cplex parameter structure. E.g., struct('simplex',struct('tolerances',struct('feasibility',1e-8)))
0051 % LP                Cplex LP object from, e.g., calling FVAComCplex
0052 %
0053 %OUTPUT
0054 % minFlux       Minimum flux for each reaction
0055 % maxFlux       Maximum flux for each reaction
0056 %
0057 %OPTIONAL OUTPUT
0058 % minFD         #rxnFluxList x #rxnNameList matrix containing the fluxes in
0059 %               options.rxnFluxList corresponding to minimizing each reaction in
0060 %               options.rxnNameList
0061 % maxFD         #rxnFluxList x #rxnNameList matrix containing the fluxes in
0062 %               options.rxnFluxList corresponding to maximizing each reaction in
0063 %               options.rxnNameList
0064 % LP            Cplex LP object
0065 % GR            the growth rate at which FVA is performed
0066 
0067 %% Initialization
0068 %check required fields for community model
0069 if ~isfield(modelCom,'indCom')
0070     if ~isfield(modelCom,'infoCom') || ~isstruct(modelCom.infoCom) || ...
0071             ~all(isfield(modelCom.infoCom,{'spBm','EXcom','EXsp','spAbbr','rxnSps','metSps'}))
0072         error('infoCom must be provided for calculating the max. community growth rate.\n');
0073     end
0074     %get useful reaction indices
0075     modelCom.indCom = infoCom2indCom(modelCom);
0076 end
0077 
0078 %get paramters
0079 if ~exist('options', 'var')
0080     options = struct();
0081 end
0082 if ~exist('solverParam', 'var') || isempty(solverParam)
0083     %default Cplex parameters
0084     solverParam = getCobraComParams('CplexParam');
0085 end
0086 param2get = {'GR', 'optBMpercent', 'rxnNameList', 'rxnFluxList', ...
0087              'GRfx', 'BMmaxLB','BMmaxUB',...
0088              'threads','verbFlag','loadModel','saveFVA'};
0089 eval(sprintf('[%s] = getCobraComParams(param2get, options, modelCom);', ...
0090             strjoin(param2get, ',')...
0091             )...
0092     );
0093 [feasTol, ~] = getCobraSolverParams('LP',{'feasTol'; 'optTol'}, solverParam);
0094 if isfield(solverParam,'simplex') && isfield(solverParam.simplex, 'tolerances')...
0095         && isfield(solverParam.simplex.tolerances,'feasibility')
0096     %override the feasTol in CobraSolverParam if given in solverParam
0097     feasTol = solverParam.simplex.tolerances.feasibility;
0098 else
0099     %otherwise use that in the solver
0100     solverParam.simplex.tolerances.feasibility = feasTol;
0101 end
0102 
0103 [m, n] = size(modelCom.S);
0104 nRxnSp = sum(modelCom.indCom.rxnSps > 0); %number of species-specific rxns
0105 nSp = numel(modelCom.indCom.spBm); %number of species
0106 
0107 if (~isfield(modelCom,'b'))
0108     modelCom.b = zeros(size(modelCom.S,1),1);
0109 end
0110 
0111 %% setup LP structure
0112 checkBMrow = false;
0113 if isempty(GR)
0114     %if max growth rate not given, find it and get the LP problem
0115     options2 = options;
0116     options2.minNorm = false;
0117     [~, result,LP] = SteadyComCplex(modelCom, options2,solverParam);
0118     GR = result.GRmax;
0119     idRow = size(LP.Model.A,1);
0120     addRow = false;
0121 elseif nargin < 4
0122     if ~isempty(loadModel)
0123         % load solution if given and growth rate is known
0124         LP = Cplex('fva');
0125         LP.readModel([loadModel '.mps']);
0126         LP.readBasis([loadModel '.bas']);
0127         LP.readParam([loadModel '.prm']);
0128         fprintf('Load model ''%s'' successfully.\n', loadModel);
0129         checkBMrow = true;
0130     else
0131         %get LP using SteadyComCplex if only growth rate is given
0132         options2 = options;
0133         options2.LPonly = true;
0134         [~, ~, LP] = SteadyComCplex(modelCom, options2, solverParam);
0135         %no constraint on total biomass using LPonly option
0136         addRow = true;
0137     end
0138 else
0139     %GR given as input and LP is supplied, expected when called by fluxVarComCplex
0140     checkBMrow = true;
0141 end
0142 %Check if a row constraining the sum of biomass exists
0143 if checkBMrow
0144     if size(LP.Model.A,1) > m + 2*nRxnSp + nSp
0145         [ynRow,idRow] = ismember(sparse(ones(nSp,1),n+1:n+nSp,ones(nSp,1),1,n+nSp),...
0146             LP.Model.A(m+2*nRxnSp+nSp+1:end,1:n+nSp),'rows');
0147         if ynRow
0148             idRow = m + 2*nRxnSp + nSp + idRow;
0149         end
0150         addRow = ~ynRow;
0151     else
0152         addRow = true;
0153     end
0154 end
0155 %add a row for constraining the sum of biomass if not exist
0156 if addRow
0157     %using default BMmaxLB and BMmaxUB if not given in options
0158     LP.addRows(BMmaxLB * optBMpercent / 100, ...
0159         sparse(ones(1, nSp), n + 1: n + nSp, ones(1, nSp), 1, size(LP.Model.A,2)),...
0160         BMmaxUB, 'UnityBiomass');
0161     idRow = size(LP.Model.A,1);
0162 else
0163     %using BMmaxLB and BMmaxUB stored in the LP if not given in options
0164     if ~isfield(options,'BMmaxLB') %take from LP if not supplied
0165         BMmaxLB = LP.Model.lhs(idRow);
0166     end
0167     if ~isfield(options,'BMmaxUB') %take from LP if not supplied
0168         BMmaxUB = LP.Model.rhs(idRow);
0169     end
0170     LP.Model.lhs(idRow) = BMmaxLB * optBMpercent / 100;
0171     %not allow the max. biomass to exceed the one at max growth rate,
0172     %can happen if optBMpercent < 100. May dismiss this constraint or
0173     %manually supply BMmaxUB in the options if sum of biomass should be variable
0174     LP.Model.rhs(idRow) = BMmaxUB;
0175 end
0176 %set Cplex parameters
0177 LP = setCplexParam(LP, solverParam);
0178 nVar = size(LP.Model.A,2);
0179 
0180 BMmax0 = LP.Model.lhs(idRow);
0181 %update the LP to ensure the current growth rate is constrained
0182 LP.Model.A = updateLPcom(modelCom, GR, GRfx, [], LP.Model.A, []);
0183 LP.Model.sense = 'minimize';
0184 LP.Model.obj(:) = 0;
0185 LP.solve();
0186 %check and adjust for feasibility
0187 dev = checkSolFeas(LP);
0188 kBMadjust = 0;
0189 while (~isfield(LP.Solution, 'x') || dev > feasTol) && kBMadjust < 10
0190     kBMadjust = kBMadjust + 1;
0191     LP.Model.lhs(end) = BMmax0 * (1 - feasTol/(11 - kBMadjust));
0192     LP.solve();
0193     dev = checkSolFeas(LP);
0194     if verbFlag
0195         fprintf('BMmax adjusment: %d\n',kBMadjust);
0196     end
0197 end
0198 
0199 if (~isfield(LP.Solution, 'x') || dev > feasTol)
0200     error('Model not feasible.')
0201 end
0202 BMmax0 = LP.Model.lhs(idRow);
0203 
0204 %% handle variables for FVA (objective matrix) and fluxes to return
0205 %fluxes to return
0206 if isnumeric(rxnFluxList)
0207     rxnFluxId = rxnFluxList;
0208 elseif iscell(rxnFluxList) || ischar(rxnFluxList)
0209     rxnFluxId = findRxnIDs(modelCom,rxnFluxList);
0210     if any(rxnFluxId) == 0
0211         error('Invalid names in rxnFluxList.');
0212     end
0213 end
0214 
0215 %objective matrix
0216 if ischar(rxnNameList)
0217     %if input is a string, make it a cell
0218     rxnNameList = {rxnNameList};
0219 end
0220 if isnumeric(rxnNameList)
0221     %if input is numeric
0222     if size(rxnNameList,1) >= n && size(rxnNameList,1) <= nVar
0223         %it is a matrix of objective vectors
0224         objList = [sparse(rxnNameList); sparse(nVar - size(rxnNameList,1), size(rxnNameList,2))];
0225     elseif size(rxnNameList,1) == 1 || size(rxnNameList,2) == 1 
0226         %reaction index
0227         objList = sparse(rxnNameList, 1:numel(rxnNameList), ones(numel(rxnNameList),1),...
0228             nVar, max(size(rxnNameList)));
0229     else
0230         error('Invalid numerical input of rxnNameList.');
0231     end
0232 elseif iscell(rxnNameList)
0233     %handle cell input
0234     objList = sparse(nVar, numel(rxnNameList));
0235     for jRxnName = 1:numel(rxnNameList)
0236         %each rxnNameList{jRxnName} can be a cell array of reactions
0237         %treat it as unweighted sum of the reactions
0238         rJ = findRxnIDs(modelCom,rxnNameList{jRxnName});
0239         if ~all(rJ)
0240             error('Invalid names in rxnNameList');
0241         end
0242         objList(rJ,jRxnName) = 1;
0243     end
0244 else
0245     error('Invalid input of rxnNameList');
0246 end
0247 
0248 %parallel computation
0249 p = gcp('nocreate');
0250 if isempty(p)
0251     if threads > 1
0252         %given explicit no. of threads
0253         parpool(ceil(threads));
0254     elseif threads ~= 1
0255         %default max no. of threads (input 0 or -1 etc)
0256         parpool;
0257     end
0258 end
0259 if ~isempty(saveFVA)
0260     directory = strsplit(saveFVA,filesep);
0261     if numel(directory) > 1
0262         %not saving in the current directory. Check existence
0263         directory = strjoin(directory(1:end-1),filesep);
0264         if ~exist(directory,'dir')
0265             mkdir(directory);
0266         end
0267     end
0268 end
0269 if verbFlag
0270     fprintf('\nFVA for %d sets of fluxes/biomass at growth rate %.6f :\n',...
0271         size(objList,2), GR);
0272 end
0273 %% main loop of FVA
0274 if threads == 1 
0275     % single-thread FVA
0276     if (verbFlag == 1)  
0277         h = waitbar(0,'Flux variability analysis in progress ...');
0278     end
0279     if (verbFlag > 1)
0280         fprintf('%4s\t%4s\t%10s\t%9s\t%9s\n','No','%','Name','Min','Max');
0281     end
0282 
0283     m = 0;
0284     maxFlux = zeros(size(objList,2), 1);
0285     minFlux = zeros(size(objList,2), 1);
0286     [minFD, maxFD] = deal(sparse(numel(rxnFluxId), size(objList,2)));
0287     i0 = 0;
0288     if ~isempty(saveFVA)
0289         %save the master model
0290         LPmodel = LP.Model;
0291         LPstart = LP.Start;
0292         optionsFVA = options;
0293         save([saveFVA '_model.mat'], 'LPmodel','LPstart','optionsFVA');
0294         clear LPmodel LPstart optionsFVA
0295         %continue from previous saved file
0296         if ~isempty(saveFVA)
0297             if exist([saveFVA '.mat'], 'file')
0298                 load([saveFVA '.mat'],'i0','minFlux','maxFlux','minFD','maxFD');
0299                 if i0 == size(objList,2)
0300                     fprintf('FVA was already finished previously and saved in %s.mat.\n', saveFVA);
0301                     return
0302                 else
0303                     fprintf('Continue FVA from i = %d.\n', i0);
0304                 end
0305             end
0306         end
0307     end
0308     for i = (i0 + 1):size(objList,2)
0309         if (verbFlag == 1)
0310             fprintf('iteration %d.  skipped %d\n', i, round(m));
0311         end
0312         %maximize
0313         LP.Model.obj = -full(objList(:,i)); 
0314         LP.solve();
0315         while ~isprop(LP, 'Solution')
0316             try
0317                 LP.solve();
0318             catch
0319                 %should not happen
0320                 fprintf('Error in solving!')
0321             end
0322         end
0323         %Infeasibility can occur occasionally. Keep trying to relax the maximum biomass
0324         eps0 = 1e-8;
0325         while ~(checkSolFeas(LP) <= feasTol) && eps0 * 10 <= 1e-3 %LP.Solution.status ~= 1
0326             eps0 = eps0 * 10;
0327             LP.Model.lhs(idRow) = BMmax0 * (1 - eps0);
0328             LP.solve();
0329         end
0330         if ~(checkSolFeas(LP) <= feasTol)
0331             %infeasible (suggests numerical issues)
0332             maxFlux(i) = NaN;
0333             maxFD(:,i) = NaN;
0334         else
0335             %LP.Solution.fval can sometimes return NaN even if a solution is found
0336             maxFlux(i) = -LP.Model.obj' * LP.Solution.x;
0337             maxFD(:,i) = LP.Solution.x(rxnFluxId);
0338         end
0339         %restore the original BMmax0
0340         LP.Model.lhs(idRow) = BMmax0;
0341         
0342         %minimize
0343         LP.Model.obj = full(objList(:,i));
0344         LP.solve();
0345         while ~isprop(LP, 'Solution')
0346             try
0347                 LP.solve();
0348             catch
0349                 %should not happen
0350                 fprintf('Error in solving!')
0351             end
0352         end
0353         %Infeasibility can occur occasionally. Keep trying to relax the maximum biomass
0354         eps0 = 1e-8;
0355         while ~(checkSolFeas(LP) <= feasTol) && eps0 * 10 <= 1e-3 %LP.Solution.status ~= 1
0356             eps0 = eps0 * 10;
0357             LP.Model.lhs(idRow) = BMmax0 * (1 - eps0);
0358             LP.solve();
0359         end
0360         if ~(checkSolFeas(LP) <= feasTol)
0361             minFlux(i) = NaN;
0362             minFD(:,i) = NaN;
0363         else
0364             minFlux(i) = LP.Model.obj' * LP.Solution.x;
0365             minFD(:,i) = LP.Solution.x(rxnFluxId);
0366         end
0367         LP.Model.lhs(idRow) = BMmax0;
0368         
0369         if (verbFlag == 1)
0370             waitbar(i/length(rxnNameList),h);
0371         end
0372         if (verbFlag > 1)
0373             rxnNameDisp = strjoin(cellstr(LP.Model.colname(objList(:,i)~=0,:)),' + ');
0374             fprintf('%4d\t%4.0f\t%10s\t%9.6f\t%9.6f\n',i,100*i/size(objList,2),rxnNameDisp,minFlux(i),maxFlux(i));
0375         end
0376         if mod(i, floor(size(objList,2)/50)) == 0 
0377             if ~isempty(saveFVA)
0378                 %save intermediate results
0379                 i0 = i;
0380                 save([saveFVA '.mat'],...
0381                     'i0','minFlux','maxFlux','minFD','maxFD')
0382             end
0383         end
0384     end
0385     if ~isempty(saveFVA)
0386         %save final results
0387         i0 = i;
0388         save([saveFVA '.mat'],...
0389             'i0','minFlux','maxFlux','minFD','maxFD')
0390     end
0391     if (verbFlag == 1)
0392         if ( regexp( version, 'R20') )
0393             close(h);
0394         end
0395     end
0396 else
0397     %% parallel FVA
0398     i0P = 0;
0399     if ~isempty(saveFVA)
0400         %check if previous results from single-thread computation exist
0401         if exist([saveFVA '.mat'], 'file')
0402             load([saveFVA '.mat'],'i0');
0403             i0P = i0;
0404             clear i0
0405             if i0P == size(objList,2)
0406                 fprintf('FVA was already finished previously and saved in %s.mat.\n', saveFVA);
0407                 load([saveFVA '.mat'],'minFlux','maxFlux','minFD','maxFD');
0408                 return
0409             else
0410                 fprintf('Continue FVA from i = %d.\n', i0P);
0411             end
0412         end
0413     end
0414     
0415     p = gcp;
0416     numPool = p.NumWorkers;
0417     %assign reactions to each thread, from reaction i0P to size(objList,2)
0418     rxnRange = cell(numPool,1);
0419     remainder = mod(size(objList,2)-i0P,numPool);
0420     kRxnDist = i0P;
0421     for jP = 1:numPool
0422         if jP <= remainder
0423             rxnRange{jP} = (kRxnDist + 1):(kRxnDist + floor((size(objList,2)-i0P) / numPool) + 1);
0424         else
0425             rxnRange{jP} = (kRxnDist + 1):(kRxnDist + floor((size(objList,2)-i0P) / numPool));
0426         end
0427         kRxnDist = kRxnDist + numel(rxnRange{jP});
0428     end
0429     LPmodel = LP.Model;
0430     LPstart = LP.Start;
0431     if ~isempty(saveFVA)
0432         %save the master model
0433         optionsFVA = options;
0434         save([saveFVA '_model.mat'], 'LPmodel','LPstart','optionsFVA','rxnRange','numPool');
0435     end
0436     
0437     [maxFluxCell, minFluxCell, minFDCell, maxFDCell] = deal(cell(numPool,1));
0438     fprintf('%s\n',saveFVA);
0439     %for technical reasons, declare the variables for Matlab
0440     save('FVAparallelTmpVar.mat','saveFVA','verbFlag');
0441     data = load('FVAparallelTmpVar.mat');
0442     saveFVA = data.saveFVA;
0443     verbFlag = data.verbFlag;
0444     parfor jP = 1:numPool
0445         LPp = Cplex('subproblem');
0446         LPp.Model = LPmodel;
0447         LPp = setCplexParam(LPp, solverParam);
0448         LPp.Start = LPstart;
0449         maxFluxP = zeros(numel(rxnRange{jP}), 1);
0450         minFluxP = zeros(numel(rxnRange{jP}), 1);
0451         [minFDP, maxFDP] = deal(sparse(numel(rxnFluxId), numel(rxnRange{jP})));
0452         iCount = 0; %counter of reactions to go
0453         iSkip = 0; %previously finished reactions
0454         if ~isempty(saveFVA)
0455             %check if previous save of parallel computation exists
0456             if exist([saveFVA '_thread' num2str(jP) '.mat'], 'file')
0457                 [minFluxP,maxFluxP,minFDP,maxFDP,iSkip] = parLoad(jP,saveFVA);
0458                 fprintf('Thread %d: continue FVA from i = %d.\n', jP, iSkip);
0459             end
0460         end
0461         for i = rxnRange{jP}
0462             iCount = iCount + 1;
0463             if i > iSkip
0464                 %maximize
0465                 LPp.Model.obj = -full(objList(:,i));
0466                 LPp.solve();
0467                 while ~isprop(LPp, 'Solution')
0468                     try
0469                         LPp.solve();
0470                     catch
0471                         %should not happen
0472                         fprintf('Error in solving!')
0473                     end
0474                 end
0475                 %Infeasibility can occur occasionally. Keep trying to relax the maximum biomass
0476                 eps0 = 1e-8;
0477                 while ~(checkSolFeas(LPp) <= feasTol) && eps0 * 10 <= 1e-3 %LP.Solution.status ~= 1
0478                     eps0 = eps0 * 10;
0479                     LPp.Model.lhs(idRow) = BMmax0 * (1 - eps0);
0480                     LPp.solve();
0481                 end
0482                 if ~(checkSolFeas(LPp) <= feasTol)
0483                     %infeasible (suggests numerical issues)
0484                     maxFluxP(iCount) = NaN;
0485                     maxFDP(:,iCount) = NaN;
0486                 else
0487                     %LP.Solution.fval can sometimes return NaN even if a solution is found
0488                     maxFluxP(iCount) = -LPp.Model.obj' * LPp.Solution.x;
0489                     maxFDP(:,iCount) = LPp.Solution.x(rxnFluxId);
0490                 end
0491                 LPp.Model.lhs(idRow) = BMmax0;
0492                 
0493                 %minimize
0494                 LPp.Model.obj = full(objList(:,i));
0495                 LPp.solve();
0496                 while ~isprop(LPp, 'Solution')
0497                     try
0498                         LPp.solve();
0499                     catch
0500                         %should not happen
0501                         fprintf('Error in solving!')
0502                     end
0503                 end
0504                 %Infeasibility can occur occasionally. Keep trying to relax the maximum biomass
0505                 eps0 = 1e-8;
0506                 while ~(checkSolFeas(LPp) <= feasTol) && eps0 * 10 <= 1e-3 %LP.Solution.status ~= 1
0507                     eps0 = eps0 * 10;
0508                     LPp.Model.lhs(idRow) = BMmax0 * (1 - eps0);
0509                     LPp.solve();
0510                 end
0511                 if ~(checkSolFeas(LPp) <= feasTol)
0512                     minFluxP(iCount) = NaN;
0513                     minFDP(:,iCount) = NaN;
0514                 else
0515                     minFluxP(iCount) = LPp.Model.obj' * LPp.Solution.x;
0516                     minFDP(:,iCount) = LPp.Solution.x(rxnFluxId);
0517                 end
0518                 LPp.Model.lhs(idRow) = BMmax0;
0519 
0520                 if mod(iCount, ceil(numel(rxnRange{jP})/10)) == 0 
0521                     if (verbFlag)
0522                         fprintf('Thread %d:\t%.2f%% finished. %04d-%02d-%02d %02d:%02d:%02.0f\n',...
0523                             jP, iCount / numel(rxnRange{jP}) * 100, clock);
0524                     end
0525                     %save intermediate data
0526                     if ~isempty(saveFVA)
0527                         parSave(minFluxP,maxFluxP,minFDP,maxFDP,i,jP,saveFVA)
0528                     end
0529                 end
0530             end
0531         end
0532         if ~isempty(saveFVA)
0533             %save finished data for each thread
0534             parSave(minFluxP,maxFluxP,minFDP,maxFDP,i,jP,saveFVA)
0535         end
0536         maxFluxCell{jP} = maxFluxP;
0537         minFluxCell{jP} = minFluxP;
0538         maxFDCell{jP} = maxFDP;
0539         minFDCell{jP} = minFDP;
0540     end
0541     %collect all results
0542     [maxFlux, minFlux, minFD, maxFD] = deal([]);
0543     if exist([saveFVA '.mat'], 'file')
0544         load([saveFVA '.mat'],'minFlux','maxFlux','minFD','maxFD'); 
0545     end
0546     for jP = 1:numPool
0547         maxFlux = [maxFlux; maxFluxCell{jP}];
0548         minFlux = [minFlux; minFluxCell{jP}];
0549         maxFD = [maxFD maxFDCell{jP}];
0550         minFD = [minFD minFDCell{jP}];
0551         maxFluxCell{jP} = [];
0552         minFluxCell{jP} = [];
0553         maxFDCell{jP} = [];
0554         minFDCell{jP} = [];
0555     end
0556     if ~isempty(saveFVA)
0557         i0 = size(objList,2);
0558         save([saveFVA '.mat'],'i0','minFlux','maxFlux','minFD','maxFD');
0559     end
0560 end
0561 
0562 maxFlux = columnVector(maxFlux);
0563 minFlux = columnVector(minFlux);
0564 end
0565 
0566 function parSave(minFluxP,maxFluxP,minFDP,maxFDP,i0,jP,saveFVA)
0567 save([saveFVA '_thread' num2str(jP) '.mat'],...
0568     'i0','minFluxP','maxFluxP','minFDP','maxFDP','jP')
0569 end
0570 
0571 function [minFluxP,maxFluxP,minFDP,maxFDP,i0] = parLoad(jP,saveFVA)
0572 load([saveFVA '_thread' num2str(jP) '.mat'],...
0573     'i0','minFluxP','maxFluxP','minFDP','maxFDP')
0574 end
```

---

Generated on Sat 06-May-2017 09:55:30 by **m2html** © 2005
