# Supplementary material for: SteadyCom: Predicting microbial abundances while ensuring community stability
Source: PLoS Comput Biol. 2017 May 15;13(5):e1005539. doi: 10.1371/journal.pcbi.1005539 (PMC5448816; doi:10.1371/journal.pcbi.1005539)
Supplement: S1 Dataset — (ZIP) [file pcbi.1005539.s018.zip › S1 Dataset/SteadyCom/doc/SteadyCom/auxiliary_functions/SteadyComPOAgrCplex.html]

Description of SteadyComPOAgrCplex


# SteadyComPOAgrCplex

## PURPOSE

**Pairwise POA for community model at community steady-state at a given growth rate**

## SYNOPSIS

**function [POAtable, fluxRange, Stat, pairList] = SteadyComPOAgrCplex(modelCom,options,solverParam,LP)**

## DESCRIPTION

```
Pairwise POA for community model at community steady-state at a given growth rate
[POAtable, fluxRange, Stat, pairList] = SteadyComPOAgrCplex(modelCom,options,solverParam,LP)

INPUT
 modelCom        a community COBRA model structure with the following extra fields:
 (the following fields are required - others can be supplied)
   S            Stoichiometric matrix
   b            Right hand side
   c            Objective coefficients
   lb           Lower bounds
   ub           Upper bounds
 (at least one of the below two is needed)
   infoCom      structure containing community reaction info 
                (returned along with the community model created with createCommModel)
   indCom       the index structure corresponding to infoCom

 options (optional) option structure with the following fields:
   GR              The growth rate at which POA is performed. If not
                     given, find the maximum growth rate.
   optBMpercent    Only consider solutions that yield at least a certain
                     percentage of the optimal biomass (Default = 95)
   rxnNameList     List of reactions (IDs or .rxns) to be analyzed.
                     Use a (N_rxns + N_organism) x K matrix for POA of K
                     linear combinations of fluxes and/or abundances
                     (Default = biomass reaction of each species)
   pairList        Pairs in rxnNameList to be analyzed. N_pair by 2 array of:
                     - indices referring to the rxns in rxnNameList, e.g.,
                       [1 2] to analyze rxnNameList{1} vs rxnNameList{2}
                     - rxn names which are members of rxnNameList, e.g.,
                       {'EX_glc-D(e)','EX_ac(e)'}
                     If not supplied, analyze all K(K-1) pairs from the K
                     targets in rxnNameList.
   symmetric       Used only when pairList is not supplied. Avoid running 
                     symmetric pairs (e.g. j vs k and k vs j)
   Nstep           Number of steps for fixing one flux at a value between 
                     the min. and the max. possible fluxes. Default 10.
                   Can also be a vector indicating the fraction of intermediate value to be analyzed
                   e.g. [0 0.5 1] means computing at minFlux, 0.5(minFlux + maxFlux) and maxFlux
   NstepScale      Used only when Nstep is a single number. 
                     -'lin' for a linear (uniform) scale of step size 
                     -'log' for a log scaling of the step sizes
   fluxRange       Flux range for each entry in rxnNameList. K x 2 matrix.
                   Defaulted to be found by SteadyComFVACplex.m
  (other parameters)
   savePOA         Must be non-empty. The filename to save the POA results
                   (default 'POAtmp/POA')
   threads         > 1 for explicitly stating the no. of threads used,
                   0 or -1 for using all available threads. Default 1.
   verbFlag        Verbose output. 0 or 1.
   loadModel       String of filename to be loaded. If non-empty, load the 
                   cplex model ('loadModel.mps'), basis ('loadModel.bas') 
                   and parameters ('loadModel.prm').
  May add also other parameters in SteadyComCplex for calculating the maximum growth rate.

OUTPUT
 POAtable          K x K cells. 
                   (i,i)-cell contains the flux range of rxnNameList{i}
                   (i,j)-cell contains a Nstep x 2 matrix, with (k,1)-entry 
                   being the min of rxnNameList{j} when rxnNameList{i} is 
                   fixed at the k-th value, (k,2)-entry being the max.
 fluxRange         K x 2 matrix of flux range for each entry in rxnNameList 
 Stat              K x K structure array with fields:
                     -'cor': the slope from linear regression between the
                             fluxes of a pair
                     -'r2':  the corresponding coefficient of determination (R-square)
 pairList          pairList after transformation from various input formats
```

## CROSS-REFERENCE INFORMATION

This function calls:

- SteadyComCplex Find the maximum community growth rate at community steady-state using SteadyCom
- SteadyComFVAgrCplex Flux variability analysis for community model at community steady-state at a given growth rate.
- checkSolFeas Check the feasibility of a solution given a COBRA model structure or a CPLEX dynamic object and a solution
- getCobraComParams get the required default parameters
- infoCom2indCom Transform between community reaction IDs and reaction names
- setCplexParam Set the parameters of the CPLEX object according to the structure solverParam
- updateLPcom Create and update the SteadyCom LP model in CPLEX format.

This function is called by:

- SteadyComPOACplex Pairwise POA for community model at community steady-state for a range of growth rates

## SUBFUNCTIONS

- function iSave(savePOA,POAtableJK,StatJK,GR,j0,k0)

## SOURCE CODE

```
0001 function [POAtable, fluxRange, Stat, pairList] = SteadyComPOAgrCplex(modelCom,options,solverParam,LP)
0002 %Pairwise POA for community model at community steady-state at a given growth rate
0003 %[POAtable, fluxRange, Stat, pairList] = SteadyComPOAgrCplex(modelCom,options,solverParam,LP)
0004 %
0005 %INPUT
0006 % modelCom        a community COBRA model structure with the following extra fields:
0007 % (the following fields are required - others can be supplied)
0008 %   S            Stoichiometric matrix
0009 %   b            Right hand side
0010 %   c            Objective coefficients
0011 %   lb           Lower bounds
0012 %   ub           Upper bounds
0013 % (at least one of the below two is needed)
0014 %   infoCom      structure containing community reaction info
0015 %                (returned along with the community model created with createCommModel)
0016 %   indCom       the index structure corresponding to infoCom
0017 %
0018 % options (optional) option structure with the following fields:
0019 %   GR              The growth rate at which POA is performed. If not
0020 %                     given, find the maximum growth rate.
0021 %   optBMpercent    Only consider solutions that yield at least a certain
0022 %                     percentage of the optimal biomass (Default = 95)
0023 %   rxnNameList     List of reactions (IDs or .rxns) to be analyzed.
0024 %                     Use a (N_rxns + N_organism) x K matrix for POA of K
0025 %                     linear combinations of fluxes and/or abundances
0026 %                     (Default = biomass reaction of each species)
0027 %   pairList        Pairs in rxnNameList to be analyzed. N_pair by 2 array of:
0028 %                     - indices referring to the rxns in rxnNameList, e.g.,
0029 %                       [1 2] to analyze rxnNameList{1} vs rxnNameList{2}
0030 %                     - rxn names which are members of rxnNameList, e.g.,
0031 %                       {'EX_glc-D(e)','EX_ac(e)'}
0032 %                     If not supplied, analyze all K(K-1) pairs from the K
0033 %                     targets in rxnNameList.
0034 %   symmetric       Used only when pairList is not supplied. Avoid running
0035 %                     symmetric pairs (e.g. j vs k and k vs j)
0036 %   Nstep           Number of steps for fixing one flux at a value between
0037 %                     the min. and the max. possible fluxes. Default 10.
0038 %                   Can also be a vector indicating the fraction of intermediate value to be analyzed
0039 %                   e.g. [0 0.5 1] means computing at minFlux, 0.5(minFlux + maxFlux) and maxFlux
0040 %   NstepScale      Used only when Nstep is a single number.
0041 %                     -'lin' for a linear (uniform) scale of step size
0042 %                     -'log' for a log scaling of the step sizes
0043 %   fluxRange       Flux range for each entry in rxnNameList. K x 2 matrix.
0044 %                   Defaulted to be found by SteadyComFVACplex.m
0045 %  (other parameters)
0046 %   savePOA         Must be non-empty. The filename to save the POA results
0047 %                   (default 'POAtmp/POA')
0048 %   threads         > 1 for explicitly stating the no. of threads used,
0049 %                   0 or -1 for using all available threads. Default 1.
0050 %   verbFlag        Verbose output. 0 or 1.
0051 %   loadModel       String of filename to be loaded. If non-empty, load the
0052 %                   cplex model ('loadModel.mps'), basis ('loadModel.bas')
0053 %                   and parameters ('loadModel.prm').
0054 %  May add also other parameters in SteadyComCplex for calculating the maximum growth rate.
0055 %
0056 %OUTPUT
0057 % POAtable          K x K cells.
0058 %                   (i,i)-cell contains the flux range of rxnNameList{i}
0059 %                   (i,j)-cell contains a Nstep x 2 matrix, with (k,1)-entry
0060 %                   being the min of rxnNameList{j} when rxnNameList{i} is
0061 %                   fixed at the k-th value, (k,2)-entry being the max.
0062 % fluxRange         K x 2 matrix of flux range for each entry in rxnNameList
0063 % Stat              K x K structure array with fields:
0064 %                     -'cor': the slope from linear regression between the
0065 %                             fluxes of a pair
0066 %                     -'r2':  the corresponding coefficient of determination (R-square)
0067 % pairList          pairList after transformation from various input formats
0068 
0069 %% Initialization
0070 %check required fields for community model
0071 if ~isfield(modelCom,'indCom')
0072     if ~isfield(modelCom,'infoCom') || ~isstruct(modelCom.infoCom) || ...
0073             ~all(isfield(modelCom.infoCom,{'spBm','EXcom','EXsp','spAbbr','rxnSps','metSps'}))
0074         error('infoCom must be provided for calculating the max. community growth rate.\n');
0075     end
0076     %get useful reaction indices
0077     modelCom.indCom = infoCom2indCom(modelCom);
0078 end
0079 
0080 %get paramters
0081 if ~exist('options', 'var')
0082     options = struct();
0083 end
0084 if ~exist('solverParam', 'var') || isempty(solverParam)
0085     %default Cplex parameters
0086     solverParam = getCobraComParams('CplexParam');
0087 end
0088 param2get = {'GRfx','GR', 'BMmaxLB','BMmaxUB','optBMpercent',... %parameters for finding maximum growth rate
0089              'symmetric','rxnNameList','pairList', 'fluxRange', 'Nstep', 'NstepScale',... %parameters for POA
0090              'verbFlag', 'threads', 'savePOA','loadModel',...
0091              };
0092 eval(sprintf('[%s] = getCobraComParams(param2get, options, modelCom);', ...
0093     strjoin(param2get, ',')...
0094     )...
0095     );
0096 if isempty(savePOA)
0097     %always use save option to reduce memory need
0098     savePOA = 'POAtmp/POA';
0099 end
0100 directory = strsplit(savePOA,filesep);
0101 if numel(directory) > 1
0102     %not saving in the current directory. Check existence
0103     directory = strjoin(directory(1:end-1),filesep);
0104     if ~exist(directory,'dir')
0105         mkdir(directory);
0106     end
0107 end
0108 [feasTol, ~] = getCobraSolverParams('LP',{'feasTol'; 'optTol'}, solverParam);
0109 if isfield(solverParam,'simplex') && isfield(solverParam.simplex, 'tolerances')...
0110         && isfield(solverParam.simplex.tolerances,'feasibility')
0111     %override the feasTol in CobraSolverParam if given in solverParam
0112     feasTol = solverParam.simplex.tolerances.feasibility;
0113 else
0114     %otherwise use that in the solver
0115     solverParam.simplex.tolerances.feasibility = feasTol;
0116 end
0117 
0118 %Check if the whole computation been finished before
0119 if ~isempty(savePOA)
0120     if exist(sprintf('%s.mat',savePOA), 'file')
0121         data0 = load(sprintf('%s.mat',savePOA));
0122         if isfield(data0, 'finished')
0123             if verbFlag
0124                 fprintf('Already finished. Results loaded from %s.mat\n',savePOA);
0125             end
0126             load(sprintf('%s.mat',savePOA), 'POAtable', 'fluxRange', 'Stat')
0127             return
0128         else
0129             clear data0
0130         end
0131     end
0132 end
0133 
0134 %parallel computation
0135 if isempty(gcp('nocreate'))
0136     if threads > 1
0137         %given explicit no. of threads
0138         parpool(ceil(threads));
0139     elseif threads ~= 1
0140         %default max no. of threads (input 0 or -1 etc)
0141         parpool;
0142     end
0143 end
0144 %sizes
0145 [m, n] = size(modelCom.S);
0146 nRxnSp = sum(modelCom.indCom.rxnSps > 0); %number of species-specific rxns
0147 nSp = numel(modelCom.indCom.spBm); %number of species
0148 
0149 %% handle LP structure
0150 checkBMrow = false;
0151 if isempty(GR)
0152     %if max growth rate not given, find it and get the LP problem
0153     options2 = options;
0154     options2.minNorm = false;
0155     [~, result,LP] = SteadyComCplex(modelCom, options2, solverParam);
0156     GR = result.GRmax;
0157     idRow = size(LP.Model.A,1);
0158     addRow = false;
0159 elseif nargin < 4
0160     if ~isempty(loadModel)
0161         % load solution if given and growth rate is known
0162         LP = Cplex('poa');
0163         LP.readModel([loadSol '.mps']);
0164         LP.readBasis([loadSol '.bas']);
0165         LP.readParam([loadSol '.prm']);
0166         fprintf('Load model ''%s'' successfully.\n', loadModel);
0167         checkBMrow = true;
0168     else
0169         %get LP using optimizeCbModelComCplex if only growth rate is given
0170         options2 = options;
0171         options2.LPonly = true;
0172         [~, ~, LP] = SteadyComCplex(modelCom, options2, solverParam);
0173         addRow = true;
0174     end
0175 else
0176     checkBMrow = true;
0177 end
0178 
0179 if checkBMrow && size(LP.Model.A,1) > m + 2*nRxnSp + nSp
0180     %if a row constraining the sum of biomass exists
0181     [ynRow,idRow] = ismember(sparse(ones(nSp,1),n+1:n+nSp,ones(nSp,1),1,n+nSp),...
0182             LP.Model.A(m+2*nRxnSp+nSp+1:end,1:n+nSp),'rows');
0183     if ynRow
0184         idRow = m + 2*nRxnSp + nSp + idRow;
0185     end
0186     addRow = ~ynRow;
0187 end
0188 if addRow
0189     %add a row for constraining the sum of biomass if not exist
0190     %using default BMmaxLB and BMmaxUB if not given in options
0191     LP.addRows(BMmaxLB * optBMpercent / 100, ...
0192         sparse(ones(1, nSp), n + 1: n + nSp, ones(1, nSp), 1, size(LP.Model.A,2)),...
0193         BMmaxUB, 'UnityBiomass');
0194     idRow = size(LP.Model.A,1);
0195 else
0196     %using BMmaxLB and BMmaxUB stored in the LP if not given in options
0197     if ~isfield(options,'BMmaxLB') %take from LP if not supplied
0198         BMmaxLB = LP.Model.lhs(idRow);
0199     end
0200     if ~isfield(options,'BMmaxUB') %take from LP if not supplied
0201         BMmaxUB = LP.Model.rhs(idRow);
0202     end
0203     LP.Model.lhs(idRow) = BMmaxLB * optBMpercent / 100;
0204     %not allow the max. biomass to exceed the one at max growth rate,
0205     %can happen if optBMpercent < 100. May dismiss this constraint or
0206     %manually supply BMmaxUB in the options if sum of biomass should be variable
0207     LP.Model.rhs(idRow) = BMmaxUB;
0208 end
0209 %set Cplex parameters
0210 LP = setCplexParam(LP, solverParam);
0211 BMmax0 = LP.Model.lhs(idRow);
0212 %update the LP to ensure the current growth rate is constrained
0213 LP.Model.A = updateLPcom(modelCom, GR, GRfx, [], LP.Model.A, []);
0214 LP.Model.sense = 'minimize';
0215 LP.Model.obj(:) = 0;
0216 %number of variables
0217 nVar = size(LP.Model.A,2);
0218 
0219 %% handle objective matrix
0220 if ischar(rxnNameList)
0221     %treat single character vector as cell with one element
0222     rxnNameList = {rxnNameList};
0223 end
0224 if isnumeric(rxnNameList)
0225     if size(rxnNameList,1) >= n && size(rxnNameList,1) <= nVar
0226         %it is a matrix of objective vectors
0227         objList = [sparse(rxnNameList); sparse(nVar - size(rxnNameList,1), size(rxnNameList,2))];
0228     elseif size(rxnNameList,1) == 1 || size(rxnNameList,2) == 1 
0229         %reaction index
0230         objList = sparse(rxnNameList, 1:numel(rxnNameList), ones(numel(rxnNameList),1),...
0231             nVar, max(size(rxnNameList)));
0232     else
0233         error('Invalid numerical input of rxnNameList.');
0234     end
0235 elseif iscell(rxnNameList)
0236     objList = sparse(nVar, numel(rxnNameList));
0237     for jRxnName = 1:numel(rxnNameList)
0238         rJ = findRxnIDs(modelCom,rxnNameList{jRxnName});
0239         if ~all(rJ)
0240             error('Invalid names in rxnNameList');
0241         end
0242         objList(rJ,jRxnName) = 1;
0243     end
0244 else
0245     error('Invalid input of rxnNameList');
0246 end
0247 %number of variables for POA
0248 Ncheck = size(objList,2);
0249 
0250 %% Handle pairList
0251 if isempty(pairList)
0252     %if pairList not given, run for all pairs
0253     pairList = [reshape(repmat(1:Ncheck,Ncheck,1),Ncheck^2,1), repmat((1:Ncheck)',Ncheck,1)];
0254     if symmetric 
0255         %the option 'symmetric' is only used here when pairList is not
0256         %supplied to avoid running symmetric pairs (e.g. j vs k and k vs j)
0257         pairList(pairList(:,1) > pairList(:,2),:) = [];
0258     end
0259 elseif numel(size(pairList)) ~= 2 || size(pairList,2) < 2 
0260     error('pairList must be an N-by-2 array denoting the pairs (rxn names or indices in rxnNameList) to analyze!')
0261 else
0262     if iscell(pairList) 
0263         if ~iscell(rxnNameList)
0264             error('rxnNameList must be cell array of rxn names to match the rxn names in pairList!');
0265         else
0266             if iscellstr(pairList) && iscellstr(rxnNameList)
0267                 %both are character arrays
0268                 [~,pairId] = ismember(pairList, rxnNameList);
0269             else
0270                 %Need to use isequal
0271                 pairId = zeros(size(pairList));
0272                 for jP = 1:numel(pairList)
0273                     for jR = 1:numel(rxnNameList)
0274                         if isequal(pairList{jP},rxnNameList{jR})
0275                             pairId(jP) = jR;
0276                             break
0277                         end
0278                     end
0279                 end
0280             end
0281             if ~all(all(pairId))
0282                 error('Some entries in pairList cannot be mapped to rxnNameList!')
0283             end
0284             pairList = pairId;
0285             clear pairId
0286         end
0287     end  
0288 end
0289 Npair = size(pairList,1);
0290 
0291 %% find pairs in the pairList not analyzed yet
0292 undone = true(Npair,1);
0293 if ~isempty(savePOA) 
0294     if exist(sprintf('%s.mat',savePOA),'file')
0295         fprintf('Already finished. Results were already saved to %s.mat\n',savePOA);
0296         load(sprintf('%s.mat',savePOA), 'POAtable', 'fluxRange', 'Stat');
0297         return
0298     elseif exist(sprintf('%s_POApre.mat',savePOA),'file')
0299         fluxRange = load(sprintf('%s_POApre.mat',savePOA),'fluxRange');
0300         fluxRange = fluxRange.fluxRange;
0301         for jP = 1:Npair
0302             undone(jP) = ~exist(sprintf('%s_j%d_k%d.mat',savePOA,pairList(jP,1),pairList(jP,2)), 'file');
0303         end
0304         fprintf('Unfinished pairs: %d\n', sum(undone));
0305     end
0306 end
0307 
0308 if any(undone)
0309     %% initial solve to ensure feasibility
0310     LP.solve();
0311     %check and adjust for feasibility
0312     dev = checkSolFeas(LP);
0313     kBMadjust = 0;
0314     while (~isfield(LP.Solution, 'x') || dev > feasTol) && kBMadjust < 10
0315         kBMadjust = kBMadjust + 1;
0316         LP.Model.lhs(end) = BMmax0 * (1 - feasTol/(11 - kBMadjust));
0317         LP.solve();
0318         dev = checkSolFeas(LP);
0319         if verbFlag
0320             fprintf('BMmax adjusment: %d\n',kBMadjust);
0321         end
0322     end
0323     %terminate if not feasible
0324     if (~isfield(LP.Solution, 'x') || dev > feasTol)
0325         error('Model not feasible.')
0326     end
0327     %% Find flux range by FVA
0328     if isempty(fluxRange)
0329         optionsFVA = options;
0330         optionsFVA.rxnNameList = objList;
0331         optionsFVA.GR = GR;
0332         fluxRange = zeros(size(objList,2),2);
0333         [fluxRange(:,1), fluxRange(:,2)] = SteadyComFVAgrCplex(modelCom,optionsFVA,solverParam,LP);
0334         printFluxRange = false;
0335         if ~isempty(savePOA)
0336             save(sprintf('%s_POApre.mat',savePOA),'fluxRange','GR','options',...
0337                 'objList','pairList','options','solverParam');
0338         end
0339     else
0340         printFluxRange = true;
0341     end
0342     %print flux range
0343     if verbFlag && printFluxRange
0344         fprintf('Flux range:\n');
0345         fprintf('rxn\tmin\tmax\n');
0346         for jRxn = 1:size(objList,2)
0347             strPrint = strjoin(strtrim(cellstr(LP.Model.colname(objList(:,jRxn)~=0,:))),',');
0348             fprintf('%s\t%.6f\t%.6f\n', strPrint, fluxRange(jRxn,1), fluxRange(jRxn,2));
0349         end
0350     end
0351     if (~isfield(modelCom,'b'))
0352         modelCom.b = zeros(size(modelCom.S,1),1);
0353     end
0354     
0355     Npair =sum(undone);
0356     undoneId = find(undone);
0357     
0358     %print starting point
0359     if verbFlag
0360         fprintf('\nPOA for %d pairs of reactions at growth rate %.6f\n', ...
0361             Npair - sum(pairList(undoneId,1)==pairList(undoneId,2)), GR);
0362         strPrintj = strjoin(strtrim(cellstr(LP.Model.colname(objList(:,pairList(undoneId(1),1))~=0,:))),',');
0363         strPrintk = strjoin(strtrim(cellstr(LP.Model.colname(objList(:,pairList(undoneId(1),2))~=0,:))),',');
0364         fprintf('Start from #%d %s vs #%d %s.\n', pairList(undoneId(1),1), ...
0365             strPrintj, pairList(undoneId(1),2), strPrintk);
0366         fprintf('%15s%15s%10s%10s%10s%10s   %s\n','Rxn1','Rxn2','corMin','r2','corMax','r2','Time')
0367     end
0368     lb0 = LP.Model.lb;
0369     ub0 = LP.Model.ub;
0370     addConstraint = false;
0371 
0372     if threads == 1
0373         %% single thread computation
0374         %Get data points to be computated
0375         
0376         for jP = 1:Npair
0377             [j,k] = deal(pairList(undoneId(jP),1),pairList(undoneId(jP),2));
0378             if ~exist(sprintf('%s_j%d_k%d.mat',savePOA,j,k),'file')
0379                 if abs(fluxRange(j,2) - fluxRange(j,1)) < 1e-8
0380                     fluxRangeJ = fluxRange(j, 1);
0381                     NstepJK = 1;
0382                 else
0383                     if numel(Nstep) > 1
0384                         %manually supply Nstep vector (% from min to max)
0385                         NstepJK = numel(Nstep);
0386                         fluxRangeJ = fluxRange(j,1) + (fluxRange(j,2) - fluxRange(j,1)) * Nstep;
0387                     else
0388                         %uniform step or log-scaled step
0389                         NstepJK = Nstep;
0390                         if strcmp(NstepScale, 'log')
0391                             if sign(fluxRange(j,2)) == sign(fluxRange(j,1))
0392                                 if fluxRange(j,1) > 0
0393                                     [a, b] = deal(fluxRange(j,1), fluxRange(j,2));
0394                                     fluxRangeJ = exp(log(a) + ((log(b) - log(a))/(Nstep - 1)) * (0:(Nstep - 1)));
0395                                 else
0396                                     [b, a] = deal(-fluxRange(j,1), -fluxRange(j,2));
0397                                     fluxRangeJ = exp(log(a) + ((log(b) - log(a))/(Nstep - 1)) * (0:(Nstep - 1)));
0398                                     fluxRangeJ = -fluxRangeJ(end:-1:1);
0399                                 end
0400                             else
0401                                 %Not an ideal situation. Flux ranges containing zero
0402                                 %should not use step size at log scale
0403                                 a = [-inf, (1:(Nstep-1))/(Nstep-1)];
0404                                 fluxRangeJ = fluxRange(j,1) + (fluxRange(j,2) - fluxRange(j,1)) * 0.01 * (100 .^ a);
0405                             end
0406                         else
0407                             %uniform step size
0408                             fluxRangeJ = (fluxRange(j,1) : (fluxRange(j,2) - fluxRange(j,1)) / (Nstep - 1) : fluxRange(j,2))';
0409                         end
0410                     end
0411                     fluxRangeJ = fluxRangeJ(:);
0412                 end
0413                 %delete constraint added in the previous round if any
0414                 if addConstraint
0415                     LP.delRows(size(LP.Model.A,1));
0416                     addConstraint = false;
0417                 end
0418                 %if not a single flux, but a linear combination, add explicit constraint.
0419                 if nnz(objList(:,j)) > 1
0420                     LP.addRows(-inf, objList(:,j)',inf, 'POArow');
0421                     addConstraint = true;
0422                 else
0423                     rxnNameId = objList(:,j) ~= 0;
0424                 end
0425                 
0426                 if j == k
0427                     %Nothing to analyze. Just record the flux range
0428                     POAtableJK = fluxRangeJ;
0429                     StatJK.cor = [1 1];
0430                     StatJK.r2 = [1 1];
0431                 else
0432                     fluxPOAvalue = zeros(NstepJK, 2);
0433                     %reset LP bounds
0434                     LP.Model.lb = lb0;
0435                     LP.Model.ub = ub0;
0436                     for p = 1:NstepJK
0437                         %fix flux of the j-th reaction
0438                         if addConstraint
0439                             LP.Model.lhs(end) = fluxRangeJ(p) - 1e-12;
0440                             LP.Model.rhs(end) = fluxRangeJ(p) + 1e-12;
0441                         else
0442                             LP.Model.lb(rxnNameId) = fluxRangeJ(p) - 1e-12;
0443                             LP.Model.ub(rxnNameId) = fluxRangeJ(p) + 1e-12;
0444                         end
0445                         LP.Model.obj(:) = 0;
0446                         %minimize flux of the k-th reaction
0447                         LP.Model.obj = objList(:, k);
0448                         LP.Model.sense = 'minimize';
0449                         LP.solve();
0450                         %handle possible tolerance issues
0451                         dev = checkSolFeas(LP);
0452                         eps0 = 1e-9;
0453                         while dev > feasTol && eps0 < 1e-3 %largest acceptable tolerance set to be 0.001
0454                             eps0 = eps0 * 10;
0455                             if addConstraint
0456                                 LP.Model.lhs(end) = fluxRangeJ(p) - eps0;
0457                                 LP.Model.rhs(end) = fluxRangeJ(p) + eps0;
0458                             else
0459                                 LP.Model.lb(rxnNameId) = fluxRangeJ(p) - eps0;
0460                                 LP.Model.ub(rxnNameId) = fluxRangeJ(p) + eps0;
0461                             end
0462                             LP.solve();
0463                             dev = checkSolFeas(LP);
0464                         end
0465                         if dev <= feasTol
0466                             fluxPOAvalue(p, 1) = LP.Model.obj'*LP.Solution.x;
0467                         else
0468                             fluxPOAvalue(p, 1) = NaN; %shoud not happen
0469                         end
0470                         %maximize flux of the k-th reaction
0471                         LP.Model.sense = 'maximize';
0472                         LP.solve();
0473                         dev = checkSolFeas(LP);
0474                         eps0 = 1e-9;
0475                         while dev > feasTol && eps0 < 1e-3
0476                             eps0 = eps0 * 10;
0477                             if addConstraint
0478                                 LP.Model.lhs(end) = fluxRangeJ(p) - eps0;
0479                                 LP.Model.rhs(end) = fluxRangeJ(p) + eps0;
0480                             else
0481                                 LP.Model.lb(rxnNameId) = fluxRangeJ(p) - eps0;
0482                                 LP.Model.ub(rxnNameId) = fluxRangeJ(p) + eps0;
0483                             end
0484                             LP.solve();
0485                             dev = checkSolFeas(LP);
0486                         end
0487                         if dev <= feasTol
0488                             fluxPOAvalue(p, 2) = LP.Model.obj'*LP.Solution.x;
0489                         else
0490                             fluxPOAvalue(p, 2) = NaN; %shoud not happen
0491                         end
0492                         
0493                     end
0494                     POAtableJK = fluxPOAvalue;
0495                     %simple linear regression to check correlations
0496                     notNan = ~isnan(fluxPOAvalue(:,1));
0497                     [bMin,~,~,~,statMin] = regress(fluxPOAvalue(notNan,1),...
0498                         [fluxRangeJ(notNan) ones(sum(notNan),1)]);
0499                     notNan = ~isnan(fluxPOAvalue(:,2));
0500                     [bMax,~,~,~,statMax] = regress(fluxPOAvalue(notNan,2),...
0501                         [fluxRangeJ(notNan) ones(sum(notNan),1)]);
0502                     StatJK.cor = [bMin(1) bMax(1)];
0503                     StatJK.r2 = [statMin(1) statMax(1)];
0504                 end
0505                 if verbFlag
0506                     if j ~= k
0507                         strPrintj = strjoin(strtrim(cellstr(LP.Model.colname(objList(:,j)~=0,:))),',');
0508                         strPrintk = strjoin(strtrim(cellstr(LP.Model.colname(objList(:,k)~=0,:))),',');
0509                         fprintf('%15s%15s%10.4f%10.4f%10.4f%10.4f   %04d-%02d-%02d %02d:%02d:%02.0f\n',...
0510                             strPrintj, strPrintk, StatJK.cor(1), StatJK.r2(1), ...
0511                             StatJK.cor(2), StatJK.r2(2), clock);
0512                     end
0513                 end
0514                 %save
0515                 if ~isempty(savePOA)
0516                     iSave(savePOA,POAtableJK,StatJK,GR,j,k);
0517                 end
0518             end
0519         end
0520     else
0521         %% parallel
0522         fprintf('POA in parallel...\n');
0523         
0524         %save and load the variables to ensure the parallel code processor can
0525         %recognize the variables
0526         tmpSave = 'POAtmp.mat';
0527         kTemp = 0;
0528         while exist(tmpSave,'file')
0529             kTemp = kTemp + 1;
0530             tmpSave = ['POAtmp' num2str(kTemp) '.mat'];
0531         end
0532         save(tmpSave, 'symmetric','verbFlag','savePOA','fluxRange','Nstep');
0533         tmpLoad = load(tmpSave);
0534         verbFlag = tmpLoad.verbFlag;
0535         savePOA = tmpLoad.savePOA;
0536         fluxRange = tmpLoad.fluxRange;
0537         Nstep = tmpLoad.Nstep;
0538         delete(tmpSave);
0539         clear tmpLoad
0540         LPmodel = LP.Model;
0541         LPstart = LP.Start;
0542         %parallelization using spmd to allow redistribution of jobs upon
0543         %completion of any of the workers to avoid being idle
0544         numPool = gcp;
0545         numPool = numPool.NumWorkers;
0546         while Npair > 0
0547             %mannually distribute jobs
0548             remainder = mod(Npair,numPool);
0549             nJ = floor(Npair/numPool);
0550             kJ = 0;
0551             nRange = cell(numPool,1);
0552             undoneCur = Composite();
0553             %Composite object is assigned as one cell/worker outside
0554             %spmd blocks but called as the cell content inside spmd blocks
0555             for kP = 1:numPool
0556                 if kP <= remainder
0557                     nRange{kP} = (kJ + 1) : (kJ + nJ + 1);
0558                     kJ = kJ + nJ + 1;
0559                 else
0560                     nRange{kP} = (kJ + 1) : (kJ + nJ);
0561                     kJ = kJ + nJ;
0562                 end
0563                 nRange{kP} = undoneId(nRange{kP});
0564                 undoneCur{kP} = undone(nRange{kP});
0565             end
0566             spmd
0567                 %setup local LP
0568                 LPp = Cplex('subproblem');
0569                 LPp.Model = LPmodel;
0570                 LPp = setCplexParam(LPp, solverParam);
0571                 LPp.Start = LPstart;
0572                 addConstraint = false;
0573                 %denote wether the current flag is the first to finish
0574                 first = true;
0575                 for jP = 1:numel(nRange{labindex}) %labindex = #thread
0576                     [j,k] = deal(pairList(nRange{labindex}(jP),1),pairList(nRange{labindex}(jP),2));
0577                     if ~exist(sprintf('%s_j%d_k%d.mat',savePOA,j,k),'file')
0578                         if abs(fluxRange(j,2) - fluxRange(j,1)) < 1e-8
0579                             fluxRangeJ = fluxRange(j, 1);
0580                             NstepJK = 1;
0581                         else
0582                             if numel(Nstep) > 1
0583                                 %mannually supplied Nstep vector (% from min to max)
0584                                 NstepJK = numel(Nstep);
0585                                 fluxRangeJ = fluxRange(j,1) + (fluxRange(j,2) - fluxRange(j,1)) * Nstep;
0586                             else
0587                                 %uniform steps or log-scaled steps
0588                                 NstepJK = Nstep;
0589                                 if strcmp(NstepScale, 'log')
0590                                     if sign(fluxRange(j,2)) == sign(fluxRange(j,1))
0591                                         if fluxRange(j,1) > 0
0592                                             [a, b] = deal(fluxRange(j,1), fluxRange(j,2));
0593                                             fluxRangeJ = exp(log(a) + ((log(b) - log(a))/(Nstep - 1)) * (0:(Nstep - 1)));
0594                                         else
0595                                             [b, a] = deal(-fluxRange(j,1), -fluxRange(j,2));
0596                                             fluxRangeJ = exp(log(a) + ((log(b) - log(a))/(Nstep - 1)) * (0:(Nstep - 1)));
0597                                             fluxRangeJ = -fluxRangeJ(end:-1:1);
0598                                         end
0599                                     else
0600                                         %Not an ideal situation. Flux ranges containing zero
0601                                         %should not use step size at log scale
0602                                         a = [-inf, (1:(Nstep-1))/(Nstep-1)];
0603                                         fluxRangeJ = fluxRange(j,1) + (fluxRange(j,2) - fluxRange(j,1)) * 0.01 * (100 .^ a);
0604                                     end
0605                                 else
0606                                     %linear step size
0607                                     fluxRangeJ = (fluxRange(j,1) : (fluxRange(j,2) - fluxRange(j,1)) / (Nstep - 1) : fluxRange(j,2))';
0608                                 end
0609                             end
0610                             fluxRangeJ = fluxRangeJ(:);
0611                         end
0612                         StatJK = struct();
0613                         if j == k
0614                             %Nothing to analyze. Just record the flux range
0615                             POAtableJK = fluxRangeJ;
0616                             StatJK.cor = [1 1];
0617                             StatJK.r2 = [1 1];
0618                         else
0619                             %reset LP bounds
0620                             LPp.Model.lb = lb0;
0621                             LPp.Model.ub = ub0;
0622                             %delete constraint added in the previous round if any
0623                             if addConstraint
0624                                 LPp.delRows(size(LPp.Model.A,1));
0625                                 addConstraint = false;
0626                             end
0627                             %if not a single flux, but a linear combination, add explicit constraint.
0628                             rxnNameId = [];
0629                             if nnz(objList(:,j)) > 1
0630                                 LPp.addRows(-inf, objList(:,j)',inf, 'POArow');
0631                                 addConstraint = true;
0632                             else
0633                                 rxnNameId = objList(:,j) ~= 0;
0634                             end
0635                             
0636                             fluxPOAvalue = zeros(NstepJK, 2);
0637                             for p = 1:NstepJK
0638                                 %fix flux of the j-th reaction
0639                                 if addConstraint
0640                                     LPp.Model.lhs(end) = fluxRangeJ(p) - 1e-12;
0641                                     LPp.Model.rhs(end) = fluxRangeJ(p) + 1e-12;
0642                                 else
0643                                     LPp.Model.lb(rxnNameId) = fluxRangeJ(p) - 1e-12;
0644                                     LPp.Model.ub(rxnNameId) = fluxRangeJ(p) + 1e-12;
0645                                 end
0646                                 %minimize flux of the k-th reaction
0647                                 LPp.Model.obj = objList(:, k);
0648                                 LPp.Model.sense = 'minimize';
0649                                 LPp.solve();
0650                                 %handle possible tolerance issues
0651                                 dev = checkSolFeas(LPp);
0652                                 eps0 = 1e-9;
0653                                 while dev > feasTol && eps0 < 1e-3
0654                                     eps0 = eps0 * 10;
0655                                     if addConstraint
0656                                         LPp.Model.lhs(end) = fluxRangeJ(p) - eps0;
0657                                         LPp.Model.rhs(end) = fluxRangeJ(p) + eps0;
0658                                     else
0659                                         LPp.Model.lb(rxnNameId) = fluxRangeJ(p) - eps0;
0660                                         LPp.Model.ub(rxnNameId) = fluxRangeJ(p) + eps0;
0661                                     end
0662                                     LPp.solve();
0663                                     dev = checkSolFeas(LPp);
0664                                 end
0665                                 if dev <= feasTol
0666                                     minf = LPp.Model.obj'*LPp.Solution.x;
0667                                 else
0668                                     minf = NaN; %shoud not happen
0669                                 end
0670                                 %maximize flux of the k-th reaction
0671                                 LPp.Model.sense = 'maximize';
0672                                 LPp.solve();
0673                                 %handle possible tolerance issues
0674                                 dev = checkSolFeas(LPp);
0675                                 eps0 = 1e-9;
0676                                 while dev > feasTol && eps0 < 1e-3
0677                                     eps0 = eps0 * 10;
0678                                     if addConstraint
0679                                         LPp.Model.lhs(end) = fluxRangeJ(p) - eps0;
0680                                         LPp.Model.rhs(end) = fluxRangeJ(p) + eps0;
0681                                     else
0682                                         LPp.Model.lb(rxnNameId) = fluxRangeJ(p) - eps0;
0683                                         LPp.Model.ub(rxnNameId) = fluxRangeJ(p) + eps0;
0684                                     end
0685                                     LPp.solve();
0686                                     dev = checkSolFeas(LPp);
0687                                 end
0688                                 if dev <= feasTol
0689                                     maxf = LPp.Model.obj'*LPp.Solution.x;
0690                                 else
0691                                     maxf = NaN; %shoud not happen
0692                                 end
0693                                 fluxPOAvalue(p,:) = [minf maxf];
0694                             end
0695                             
0696                             POAtableJK = fluxPOAvalue;
0697                             %simple linear regression to check correlations
0698                             notNan = ~isnan(fluxPOAvalue(:,1));
0699                             [bMin,~,~,~,statMin] = regress(fluxPOAvalue(notNan,1),...
0700                                 [fluxRangeJ(notNan) ones(sum(notNan),1)]);
0701                             notNan = ~isnan(fluxPOAvalue(:,2));
0702                             [bMax,~,~,~,statMax] = regress(fluxPOAvalue(notNan,2),...
0703                                 [fluxRangeJ(notNan) ones(sum(notNan),1)]);
0704                             StatJK.cor = [bMin(1) bMax(1)];
0705                             StatJK.r2 = [statMin(1) statMax(1)];
0706                         end
0707                         if verbFlag
0708                             if j ~= k
0709                                 strPrintj = strjoin(strtrim(cellstr(LPp.Model.colname(objList(:,j)~=0,:))),',');
0710                                 strPrintk = strjoin(strtrim(cellstr(LPp.Model.colname(objList(:,k)~=0,:))),',');
0711                                 fprintf('%15s%15s%10.4f%10.4f%10.4f%10.4f   %04d-%02d-%02d %02d:%02d:%02.0f\n',...
0712                                     strPrintj, strPrintk,...
0713                                     StatJK.cor(1), StatJK.r2(1), ...
0714                                     StatJK.cor(2), StatJK.r2(2), clock);
0715                             end
0716                         end
0717                         if ~isempty(savePOA)
0718                             iSave(savePOA,POAtableJK,StatJK,GR,j,k);
0719                         end
0720                     end
0721                     undoneCur(jP) = false;
0722                     %check if any of workers has finished its loop, break the
0723                     %loop and redistribute if finished
0724                     if labProbe('any',0);
0725                         first = false;
0726                         break
0727                     end
0728                 end
0729                 if first
0730                     %finish of one worker, call off other workers
0731                     if verbFlag
0732                         fprintf('Current loop finished. Stop other workers...\n');
0733                     end
0734                     labSend(true,setdiff(1:numlabs,labindex),0);
0735                     if verbFlag
0736                         fprintf('All workers have ceased. Redistributing...\n');
0737                     end
0738                 end
0739                 %avoid warning of missed message
0740                 pause(1e-8);
0741                 while labProbe('any',0);
0742                     pause(1e-8);
0743                     labReceive('any',0);
0744                 end
0745             end
0746             %update undone
0747             for kP = 1:numPool
0748                 undone(nRange{kP}) = undoneCur{kP};
0749             end
0750             undoneId = find(undone);
0751             Npair = numel(undoneId);
0752         end
0753     end
0754 end
0755 if verbFlag
0756     fprintf('Finished. Save final results to %s.mat\n',savePOA);
0757 end
0758 POAtable = cell(Ncheck, Ncheck);
0759 Stat = repmat(struct('cor',0,'r2',0), Ncheck, Ncheck);
0760 for jP = 1:size(pairList,1)
0761     data = load(sprintf('%s_j%d_k%d.mat',savePOA,pairList(jP,1),...
0762         pairList(jP,2)),'POAtableJK', 'StatJK');
0763     POAtable{pairList(jP,1),pairList(jP,2)} = data.POAtableJK;
0764     Stat(pairList(jP,1),pairList(jP,2)) = data.StatJK;
0765 end
0766 save(sprintf('%s.mat',savePOA),'POAtable', 'Stat', 'GR', ...
0767     'fluxRange');
0768 end
0769 
0770 function iSave(savePOA,POAtableJK,StatJK,GR,j0,k0)
0771 save(sprintf('%s_j%d_k%d.mat',savePOA,j0,k0),'POAtableJK', 'StatJK', 'GR','j0','k0');
0772 end
```

---

Generated on Sat 06-May-2017 09:55:30 by **m2html** © 2005
