# Supplementary material for: SteadyCom: Predicting microbial abundances while ensuring community stability
Source: PLoS Comput Biol. 2017 May 15;13(5):e1005539. doi: 10.1371/journal.pcbi.1005539 (PMC5448816; doi:10.1371/journal.pcbi.1005539)
Supplement: S1 Dataset — (ZIP) [file pcbi.1005539.s018.zip › S1 Dataset/SteadyCom/doc/SteadyCom/auxiliary_functions/updateLPcom.html]

Description of updateLPcom


# updateLPcom

## PURPOSE

**Create and update the SteadyCom LP model in CPLEX format.**

## SYNOPSIS

**function LPproblem = updateLPcom(modelCom, grCur, GRfx, BMcon, LPproblem, BMgdw)**

## DESCRIPTION

```
 Create and update the SteadyCom LP model in CPLEX format.
 Create the LP problem [LP(grCur)] given growth rate grCur and other
 constraints if LPproblem in the input does not contain the field 'A',
 or is empty or is not given.
 Otherwise, update LPproblem with the growth rate grCur. Only the
 arguements 'modelCom', 'grCur', 'GRfx' and 'LPproblem' are used in this
 case.

 LPproblem = updateLPcom(modelCom, grCur, GRfx, BMcon, LPproblem, BMgdw)

 Input:
   modelCom:   community model
   grCur:      the current growth rate for the LP to be updated to
   GRfx:       fixed growth rate of a certain species
   BMcon:      constraint matrix for species biomass
   LPproblem:  LP problem structure with field 'A' or the problem matrix
               directly
   BMgdw:      the gram dry weight per mmol of the biomass reaction of
               each species (nSp x 1 vector, default all 1)

 Return a structure with the field 'A' updated if the input 'LPproblem' is
 a structure or return a matrix if 'LPproblem' is the problem matrix
```

## CROSS-REFERENCE INFORMATION

This function calls:

- getCobraComParams get the required default parameters

This function is called by:

- SteadyComCplex Find the maximum community growth rate at community steady-state using SteadyCom
- SteadyComFVACplex Flux variability analysis for community model at community steady-state for a range of growth rates.
- SteadyComPOACplex Pairwise POA for community model at community steady-state for a range of growth rates
- SteadyComFVAgrCplex Flux variability analysis for community model at community steady-state at a given growth rate.
- SteadyComPOAgrCplex Pairwise POA for community model at community steady-state at a given growth rate

## SOURCE CODE

```
0001 function LPproblem = updateLPcom(modelCom, grCur, GRfx, BMcon, LPproblem, BMgdw)
0002 % Create and update the SteadyCom LP model in CPLEX format.
0003 % Create the LP problem [LP(grCur)] given growth rate grCur and other
0004 % constraints if LPproblem in the input does not contain the field 'A',
0005 % or is empty or is not given.
0006 % Otherwise, update LPproblem with the growth rate grCur. Only the
0007 % arguements 'modelCom', 'grCur', 'GRfx' and 'LPproblem' are used in this
0008 % case.
0009 %
0010 % LPproblem = updateLPcom(modelCom, grCur, GRfx, BMcon, LPproblem, BMgdw)
0011 %
0012 % Input:
0013 %   modelCom:   community model
0014 %   grCur:      the current growth rate for the LP to be updated to
0015 %   GRfx:       fixed growth rate of a certain species
0016 %   BMcon:      constraint matrix for species biomass
0017 %   LPproblem:  LP problem structure with field 'A' or the problem matrix
0018 %               directly
0019 %   BMgdw:      the gram dry weight per mmol of the biomass reaction of
0020 %               each species (nSp x 1 vector, default all 1)
0021 %
0022 % Return a structure with the field 'A' updated if the input 'LPproblem' is
0023 % a structure or return a matrix if 'LPproblem' is the problem matrix
0024 m = size(modelCom.S, 1);
0025 n = size(modelCom.S, 2);
0026 nRxnSp = sum(modelCom.indCom.rxnSps > 0);
0027 nSp = numel(modelCom.infoCom.spAbbr);
0028 if ~exist('grCur', 'var')
0029     grCur = 0;
0030 elseif isempty(grCur)
0031     grCur = 0;
0032 end
0033 if ~exist('GRfx', 'var') || isempty(GRfx)
0034     GRfx  = getCobraComParams({'GRfx'}, struct(), modelCom);
0035 end
0036 if ~exist('LPproblem', 'var')
0037     LPproblem = struct();
0038 end
0039 
0040 construct = false;
0041 if ~isstruct(LPproblem)
0042     if isempty(LPproblem)
0043         construct = true;
0044     end
0045 elseif ~isfield(LPproblem, 'A')
0046     construct = true;
0047 end
0048 if construct
0049     if ~exist('BMgdw', 'var')
0050         BMgdw = ones(nSp,1);
0051     end
0052     %upper bound matrix
0053     S_ub = sparse([1:nRxnSp 1:nRxnSp]', [(1:nRxnSp)'; n + modelCom.indCom.rxnSps(1:nRxnSp)],...
0054           [ones(nRxnSp,1); -modelCom.ub(1:nRxnSp)], nRxnSp, n + nSp);
0055     %lower bound matrix
0056     S_lb = sparse([1:nRxnSp 1:nRxnSp]', [(1:nRxnSp)'; n + modelCom.indCom.rxnSps(1:nRxnSp)],...
0057           [-ones(nRxnSp,1); modelCom.lb(1:nRxnSp)], nRxnSp, n + nSp);
0058     %growth rate and biomass link matrix
0059     grSp = zeros(nSp, 1);
0060     grSp(isnan(GRfx)) = grCur;
0061     %given fixed growth rate
0062     grSp(~isnan(GRfx)) = GRfx(~isnan(GRfx));
0063     S_gr = sparse([1:nSp 1:nSp]', [modelCom.indCom.spBm(:) (n + 1:n + nSp)'],...
0064                   [BMgdw(:); -grSp], nSp, n + nSp);
0065     if isempty(BMcon)
0066         A = [modelCom.S sparse([],[],[], m, nSp); S_ub; S_lb; S_gr];
0067     else
0068         A = [modelCom.S sparse([],[],[], m, nSp); S_ub; S_lb; S_gr;...
0069                    sparse([],[],[],size(BMcon, 1), n) BMcon];
0070     end
0071     if isstruct(LPproblem)
0072         LPproblem.A = A;
0073     else
0074         LPproblem = A;
0075     end
0076 else
0077     for j = 1:nSp
0078         if isstruct(LPproblem)
0079             if isnan(GRfx(j))
0080                 LPproblem.A(m + 2*nRxnSp + j, n + j) = -grCur;
0081             else
0082                 LPproblem.A(m + 2*nRxnSp + j, n + j) = -GRfx(j);
0083             end
0084         else
0085             if isnan(GRfx(j))
0086                 LPproblem(m + 2*nRxnSp + j, n + j) = -grCur;
0087             else
0088                 LPproblem(m + 2*nRxnSp + j, n + j) = -GRfx(j);
0089             end
0090         end
0091     end
0092 end
0093 end
0094
```

---

Generated on Sat 06-May-2017 09:55:30 by **m2html** © 2005
