# Supplementary material for: SteadyCom: Predicting microbial abundances while ensuring community stability
Source: PLoS Comput Biol. 2017 May 15;13(5):e1005539. doi: 10.1371/journal.pcbi.1005539 (PMC5448816; doi:10.1371/journal.pcbi.1005539)
Supplement: S1 Dataset — (ZIP) [file pcbi.1005539.s018.zip › S1 Dataset/SteadyCom/doc/SteadyCom/createCommModel.html]

Description of createCommModel


# createCommModel

## PURPOSE

**Create a community COBRA model. The model has an extra compartment [u]**

## SYNOPSIS

**function [modelCom,infoCom,indCom] = createCommModel(modelCell, options)**

## DESCRIPTION

```
Create a community COBRA model. The model has an extra compartment [u] 
for inter-organism and community exchange, i.e.,
 (environment) <=> [u] <=> [e_organism1]
                       <=> [e_organism2] ...

[modelCom,infoCom,indCom] = createCommModel(modelCell,options)

INPUT
 modelCell:    Cell array of COBRA model (e.g., {model1, model2, model3})
               or a structure with modelCell.org being the model for
               organism org
 (if a model in 'modelCell' has the field 'metComs', the name in
 model.metComs would be used to map community metabolites instead of the
 original name in model.mets [recommended to provide])

 options:      Structure containing the following fields
     spBm:     Cell array of reaction names of the biomass reaction in
               each model in 'modelCell'
   (below are optional, but recommended to provide)
     spAbbr:   Cell array of abbrivation for each organism in modelCell
     spName:   Full names of the species
     spATPM:   Cell array of reaction names of the ATP maintenance reaction in
               each model in 'modelCell'
    ('sp' originally for 'species')
     metExId:  Identifier for extracellular metabolites metabolites (default '[e]')
               If input is the empty string (''), find all metabolites that have exchange reactions
     rxnField: Cell array of field names in the models that have the same
               size as rxns. Default to include all fields starting
               with 'rxn' and include 'grRules', 'rules', 'confidenceScores', 'subSystems'
     metField: Cell array of field names in the models that have the same
               size as mets. Default to include all fields starting with 'met'

OUTPUT
 modelCom:     COBRA community model with the following extra fields
               'infoCom' and 'indCom', which are also outputed as separate
               output argument. See below.
 infoCom:      A structure of useful reaction names and organism names:
   spBm:          spBm{k} the biomass reaction of info.speciesAbbr{k}                                        
   spATPM:        spATPM{k} the ATP maintenance reaction of info.speciesAbbr{k}                                            
   rxnSD:         all sink and demand reactions other than the community exchange reactions (EXcom)
   EXcom:         EXcom{i,1} the community uptake reaction for community metabolite info.metCom{i}
                  EXcom{i,2} the community production reaction for community metabolite info.metCom{i}
   EXsp:          EXsp{i,k} the species-community exchange reaction for community metabolite info.metCom{i}
   Mcom:          community metabolites
   Msp:           Msp{i,k} the extracellular met of organism k
                  corresponding to community metabolite Mcom{i}
   speciesAbbr:   species abbreviation, used in mets and rxns                       
                  to identify species-specific metabolites                          
   speciesName:   full name of each species        
   rxnSps:        rxnSps{j} is the abbreviation of species k (speciesAbbr{k}) 
                  if reaction j is of species k. 'com' for community exchange reactions    
   metSps:        metSps{i} is the abbreviation of species k (speciesAbbr{k}) 
                  if metabolite i is of species k. 'com' for community exchange reactions 
 indCom:       The corresponding reaction IDs and organism IDs:
   spBm:          reaction IDs for info.spBm
   spATPM:        reaction IDs for info.spATPM
   rxnSD:         reaction IDs for info.rxnSD 
   EXcom:         reaction IDs for info.EXcom                               
   EXsp:          reaction IDs for info.EXsp. EXsp(i,k) = 0 if info.EXsp{i,k} is empty          
   Mcom:          metabolite IDs for info.Mcom
   Msp:           metabolite IDs for info.Msp
   rxnSps:        index.rxnSps(j) = k implies that reaction j is of                      
                  species info.speciesName{k}. = 0 for community exchange reactions    
   metSps:        index.metSps(i) = k implies that metabolite i is of                    
                  species info.speciesName{k}. = 0 for community metabolites
```

## CROSS-REFERENCE INFORMATION

This function calls:

- getCobraComParams get the required default parameters
- infoCom2indCom Transform between community reaction IDs and reaction names

This function is called by:


## SOURCE CODE

```
0001 function [modelCom,infoCom,indCom] = createCommModel(modelCell, options)
0002 %Create a community COBRA model. The model has an extra compartment [u]
0003 %for inter-organism and community exchange, i.e.,
0004 % (environment) <=> [u] <=> [e_organism1]
0005 %                       <=> [e_organism2] ...
0006 %
0007 %[modelCom,infoCom,indCom] = createCommModel(modelCell,options)
0008 %
0009 %INPUT
0010 % modelCell:    Cell array of COBRA model (e.g., {model1, model2, model3})
0011 %               or a structure with modelCell.org being the model for
0012 %               organism org
0013 % (if a model in 'modelCell' has the field 'metComs', the name in
0014 % model.metComs would be used to map community metabolites instead of the
0015 % original name in model.mets [recommended to provide])
0016 %
0017 % options:      Structure containing the following fields
0018 %     spBm:     Cell array of reaction names of the biomass reaction in
0019 %               each model in 'modelCell'
0020 %   (below are optional, but recommended to provide)
0021 %     spAbbr:   Cell array of abbrivation for each organism in modelCell
0022 %     spName:   Full names of the species
0023 %     spATPM:   Cell array of reaction names of the ATP maintenance reaction in
0024 %               each model in 'modelCell'
0025 %    ('sp' originally for 'species')
0026 %     metExId:  Identifier for extracellular metabolites metabolites (default '[e]')
0027 %               If input is the empty string (''), find all metabolites that have exchange reactions
0028 %     rxnField: Cell array of field names in the models that have the same
0029 %               size as rxns. Default to include all fields starting
0030 %               with 'rxn' and include 'grRules', 'rules', 'confidenceScores', 'subSystems'
0031 %     metField: Cell array of field names in the models that have the same
0032 %               size as mets. Default to include all fields starting with 'met'
0033 %
0034 %OUTPUT
0035 % modelCom:     COBRA community model with the following extra fields
0036 %               'infoCom' and 'indCom', which are also outputed as separate
0037 %               output argument. See below.
0038 % infoCom:      A structure of useful reaction names and organism names:
0039 %   spBm:          spBm{k} the biomass reaction of info.speciesAbbr{k}
0040 %   spATPM:        spATPM{k} the ATP maintenance reaction of info.speciesAbbr{k}
0041 %   rxnSD:         all sink and demand reactions other than the community exchange reactions (EXcom)
0042 %   EXcom:         EXcom{i,1} the community uptake reaction for community metabolite info.metCom{i}
0043 %                  EXcom{i,2} the community production reaction for community metabolite info.metCom{i}
0044 %   EXsp:          EXsp{i,k} the species-community exchange reaction for community metabolite info.metCom{i}
0045 %   Mcom:          community metabolites
0046 %   Msp:           Msp{i,k} the extracellular met of organism k
0047 %                  corresponding to community metabolite Mcom{i}
0048 %   speciesAbbr:   species abbreviation, used in mets and rxns
0049 %                  to identify species-specific metabolites
0050 %   speciesName:   full name of each species
0051 %   rxnSps:        rxnSps{j} is the abbreviation of species k (speciesAbbr{k})
0052 %                  if reaction j is of species k. 'com' for community exchange reactions
0053 %   metSps:        metSps{i} is the abbreviation of species k (speciesAbbr{k})
0054 %                  if metabolite i is of species k. 'com' for community exchange reactions
0055 % indCom:       The corresponding reaction IDs and organism IDs:
0056 %   spBm:          reaction IDs for info.spBm
0057 %   spATPM:        reaction IDs for info.spATPM
0058 %   rxnSD:         reaction IDs for info.rxnSD
0059 %   EXcom:         reaction IDs for info.EXcom
0060 %   EXsp:          reaction IDs for info.EXsp. EXsp(i,k) = 0 if info.EXsp{i,k} is empty
0061 %   Mcom:          metabolite IDs for info.Mcom
0062 %   Msp:           metabolite IDs for info.Msp
0063 %   rxnSps:        index.rxnSps(j) = k implies that reaction j is of
0064 %                  species info.speciesName{k}. = 0 for community exchange reactions
0065 %   metSps:        index.metSps(i) = k implies that metabolite i is of
0066 %                  species info.speciesName{k}. = 0 for community metabolites
0067 
0068 
0069 %% arguement checking
0070 if ~exist('options', 'var')
0071     options = struct();
0072 end
0073 %get parameters
0074 [spAbbr,spName,spBm,spATPM,metExId,rxnField,metField] = getCobraComParams(...
0075     {'spAbbr','spName','spBm','spATPM','metExId','rxnField','metField'}, options);
0076 %organisms' abbreviations and names
0077 nameSpecies = false;
0078 if isstruct(modelCell)
0079     if isempty(spAbbr)
0080         spAbbr = fieldnames(modelCell);
0081     end
0082     modelCell = struct2cell(modelCell);
0083 else
0084     if isempty(spAbbr)
0085         nameSpecies = true;
0086     end
0087 end
0088 nSp = numel(modelCell);
0089 if nameSpecies
0090     spAbbr = strcat('org',strtrim(cellstr(num2str((1:nSp)'))));
0091 end
0092 if isempty(spName)
0093     spName = spAbbr;
0094 end
0095 %biomass reactions
0096 findspBm = false;
0097 if isempty(spBm)
0098     error('Please provide the names of the biomass reactions in options.spBm'); 
0099 elseif numel(spBm) ~= nSp
0100     error('Number of entries in options.spBm not equal to the number of models.');
0101 elseif iscell(spBm)
0102     rxnBiomassID = zeros(nSp, 1);
0103     for j = 1: nSp
0104         rxnBiomassID(j) = findRxnIDs(modelCell{j}, spBm{j});
0105     end
0106     spBm = rxnBiomassID;
0107 end
0108 %ATPM
0109 if isempty(spATPM)
0110     spATPM = zeros(nSp,1);
0111 elseif iscell(spATPM)
0112     spATPM0 = spATPM;
0113     spATPM = zeros(nSp,1);
0114     for j = 1: nSp
0115         spATPM(j) = findRxnIDs(modelCell{j}, spATPM0{j});
0116     end
0117 end
0118 %% Copy fields from COBRA model
0119 field = {};
0120 [fieldNumeric, fieldCell, fieldStruct]  = deal(false(0));
0121 for jSp = 1:nSp
0122     fCur = fieldnames(modelCell{jSp});
0123     fNcur = cellfun(@(x) isnumeric(modelCell{jSp}.(x)),fCur);
0124     fCcur = cellfun(@(x) iscell(modelCell{jSp}.(x)),fCur);
0125     fScur = cellfun(@(x) isstruct(modelCell{jSp}.(x)),fCur);
0126     field = [field; fCur];
0127     fieldNumeric = [fieldNumeric; fNcur];
0128     fieldCell = [fieldCell; fCcur];
0129     fieldStruct = [fieldStruct; fScur];
0130 end
0131 [field,id] = unique(field);
0132 [fieldNumeric,fieldCell,fieldStruct] = deal(fieldNumeric(id),fieldCell(id),fieldStruct(id));
0133 %fields need special care
0134 id = ismember(field,{'S', 'rxns', 'mets', 'rev', 'lb', 'ub', 'c', 'b', 'metComs','rxnGeneMat','genes'});
0135 [field,fieldNumeric,fieldCell,fieldStruct] = deal(field(~id),fieldNumeric(~id),fieldCell(~id),fieldStruct(~id));
0136 rxnField = unique([rxnField(:);{'grRules';'rules';'confidenceScores';'subSystems'}]);
0137 
0138 modelCom = struct();
0139 for j = 1:numel(field)
0140     modelCom.(field{j}) = [];
0141 end
0142 
0143 %% fields to be changed
0144 S = [];
0145 rxns = {};
0146 mets = {};
0147 metsCom = {};
0148 rev = [];
0149 lb = [];
0150 ub = [];
0151 c = [];
0152 b = [];
0153 %map rxns to species
0154 rxnSps = [];
0155 %map mets to species
0156 metSps = [];
0157 %rxn IDs of biomass
0158 spBmId = zeros(nSp, 1);
0159 
0160 %extracellular metabolites
0161 ex = cell(nSp, 1);
0162 %exchange reactions
0163 rxnEx = cell(nSp, 1);
0164 %map ex mets and ex rxns
0165 rxnEx2met = cell(nSp, 1);
0166 
0167 %% loop for each species
0168 if ~isempty(metField)
0169     metFieldKnown = ismember(field,metField);
0170 else
0171     metFieldKnown = false(numel(field),1);
0172 end
0173 if ~isempty(rxnField)
0174     rxnFieldKnown = ismember(field,rxnField);
0175 else
0176     rxnFieldKnown = false(numel(field),1);
0177 end
0178 %met-related fields
0179 metFieldL = strncmp('met',field,3) | metFieldKnown;
0180 %rxn-related fields
0181 rxnFieldL = strncmp('rxn',field,3) | rxnFieldKnown;
0182 %all other fields just put in a cell for each model
0183 spFieldL = ~metFieldL & ~rxnFieldL;
0184 
0185 % metField = union(setdiff(fieldAll(strncmp('met',fieldAll,3)),...
0186 %     {'mets','metNames','metFormulas','metSps'}),...
0187 %     metField);
0188 % %rxn-related fields
0189 % rxnField = union(setdiff(fieldAll(strncmp('rxn',fieldAll,3)),...
0190 %     {'rxns','rxnNames','rxnSps','rxnGeneMat','spBm','spATPM'}),...
0191 %     rxnField);
0192 
0193 row = 0;
0194 col = 0;
0195 for j = 1:nSp
0196     [rowJ, colJ] = size(modelCell{j}.S);
0197     %get bounds and objective
0198     lbJ = modelCell{j}.lb;
0199     ubJ = modelCell{j}.ub;
0200     cJ = modelCell{j}.c;
0201     if isfield(modelCell{j}, 'metComs') %given the mapping to community metabolites
0202         if numel(modelCell{j}.metComs) < numel(modelCell{j}.mets)
0203             warning('input model %d: size of metComs < size of mets.');
0204             modelCell{j}.metComs(end+1:numel(modelCell{j}.mets)) = {''};
0205         elseif numel(modelCell{j}.metComs) > numel(modelCell{j}.mets)
0206             warning('input model %d: size of metComs > size of mets.');
0207             modelCell{j}.metComs(numel(modelCell{j}.mets)+1:end) = [];
0208         end
0209         %logical vector for extracellular metabolites
0210         ex{j} = ~cellfun(@isempty, modelCell{j}.metComs);
0211         modelCell{j}.metComs(ex{j}) = regexprep(modelCell{j}.metComs(ex{j}),'\[[^\[\]]*\]$','');
0212         %logical vector for exchange reactions
0213         rxnEx{j} = (sum(modelCell{j}.S(ex{j},:) ~= 0) == 1)' ...
0214                 & (sum(modelCell{j}.S(~ex{j},:) ~= 0) == 0)';
0215         rxnEx2met{j} = zeros(rowJ,2);
0216     elseif ~isempty(metExId) %using identifier for extracellular mets
0217         %logical vector for extracellular metabolites
0218         ex{j} = cellfun(@(x) ~isempty(strfind(x, metExId)), ...
0219             modelCell{j}.mets);
0220         modelCell{j}.metComs = repmat({''},rowJ,1);
0221         modelCell{j}.metComs(ex{j}) = regexprep(modelCell{j}.mets(ex{j}),'\[[^\[\]]*\]$','');
0222         %logical vector for exchange reactions
0223         rxnEx{j} = (sum(modelCell{j}.S(ex{j},:) ~= 0) == 1)' ...
0224                 & (sum(modelCell{j}.S(~ex{j},:) ~= 0) == 0)';
0225         %exchange reactions mapped to metabolites
0226         rxnEx2met{j} = zeros(rowJ,2);
0227     else %if no identifier, just check exchange reactions
0228         rxnEx{j} = (sum(modelCell{j}.S ~= 0) == 1)';
0229         rxnEx2met{j} = zeros(rowJ,2);
0230         ex{j} = any(modelCell{j}.S(:,rxnEx{j}),2);
0231         modelCell{j}.metComs = repmat({''},rowJ,1);
0232         modelCell{j}.metComs(ex{j}) = regexprep(modelCell{j}.mets(ex{j}),'\[[^\[\]]*\]$','');
0233     end
0234     for k = 1:colJ
0235         if rxnEx{j}(k)
0236             metJK = find(modelCell{j}.S(:, k), 1);
0237             rxnEx2met{j}(metJK,:) = [col + k, k];
0238             %set positive flux of exchange reaction as uptake
0239             %(convention)
0240             if modelCell{j}.S(metJK, k) > 0
0241                 s = modelCell{j}.S(metJK,k);
0242                 modelCell{j}.S(metJK,k) = -1;
0243                 [ubJ(k), lbJ(k), cJ(k)] = deal(-s * lbJ(k), -s * ubJ(k), cJ(k));
0244             end
0245         end
0246     end
0247     %update community metabolites (now the model has .metComs, which has no compartment identifier)
0248     metsCom = unique([metsCom; modelCell{j}.metComs(ex{j})]);    
0249     %stoichiometric matrix
0250     S = [S                 sparse(row, colJ);...
0251          sparse(rowJ, col) sparse(modelCell{j}.S)];
0252     %reversibility, bounds, objective and RHS
0253     rev = [rev; modelCell{j}.rev];
0254     lb = [lb; lbJ];
0255     ub = [ub; ubJ];
0256     c = [c; cJ];
0257     b = [b; modelCell{j}.b];
0258     %add species's name to rxns and mets (add to the compartment if exist)
0259     if 1
0260         metJ = regexprep(modelCell{j}.mets,'\]$',['_' spAbbr{j} '\]']);
0261         metJ(cellfun(@(x) ~strcmp(x(end),']'), metJ)) = strcat(metJ(cellfun(@(x) ~strcmp(x(end),']'), metJ)),'[',spAbbr{j},']');
0262         mets = [mets; metJ];
0263     else
0264         mets = [mets; strcat(modelCell{j}.mets,'[',spAbbr{j},']')];
0265     end
0266     rxns = [rxns; strcat(modelCell{j}.rxns,'_',spAbbr{j})];
0267     %incorporate other field
0268     for k = 1:numel(field)
0269         if metFieldL(k)
0270             str = 'mets';
0271             sizeCk = rowJ;
0272         elseif rxnFieldL(k)
0273             str = 'rxns';
0274             sizeCk = colJ;
0275         else
0276             %spField
0277             str = '1';
0278             sizeCk = 1;
0279         end
0280         if isfield(modelCell{j}, field{k})
0281             if spFieldL(k) && ischar(modelCell{j}.(field{k}))
0282                 %If that is a string, put it in a cell.
0283                 modelCell{j}.(field{k}) = {modelCell{j}.(field{k})};
0284             end
0285             %check sizes
0286             if size(modelCell{j}.(field{k}),1) ~= sizeCk
0287                 if size(modelCell{j}.(field{k}),2) == sizeCk
0288                     fieldJK = modelCell{j}.(field{k})';
0289                 else
0290                     error('Dimension of modelCell{%d}.%s (%d,%d) does not match %s (%d).',...
0291                         j,field{k},size(modelCell{j}.(field{k})),str,sizeCk)
0292                 end
0293             else
0294                 fieldJK = modelCell{j}.(field{k});
0295             end
0296             %assignment
0297             if isempty(modelCom.(field{k}))
0298                 modelCom.(field{k}) = fieldJK;
0299             else
0300                 modelCom.(field{k}) = [modelCom.(field{k});fieldJK];
0301             end
0302         else
0303             if isempty(modelCom.(field{k}))
0304                 if fieldNumeric(k)
0305                     modelCom.(field{k}) = zeros(sizeCk,1);
0306                 elseif fieldCell(k)
0307                     modelCom.(field{k}) = repmat({''},sizeCk,1);
0308                 elseif fieldStruct(k)
0309                     modelCom.(field{k}) = repmat(struct(),sizeCk,1);
0310                 end
0311             else
0312                 if fieldNumeric(k)
0313                     modelCom.(field{k})(end+1:end+sizeCk,:) = 0;
0314                 elseif fieldCell(k)
0315                     modelCom.(field{k})(end+1:end+sizeCk,:) = {''};
0316                 elseif fieldStruct(k)
0317                     f = fieldnames(modelCom.(field{k})(end));
0318                     modelCom.(field{k})(end+1:end+sizeCk,:) = ...
0319                         repmat(cell2struct(repmat({[]},numel(f),1),f),sizeCk,1);
0320                 end
0321             end
0322         end
0323     end
0324     
0325     %species specific rxns and mets
0326     rxnSps = [rxnSps; j * ones(colJ,1)];
0327     metSps = [metSps; j * ones(rowJ,1)];
0328     %biomass rxn ID
0329     spBmId(j) = col + spBm(j);
0330     spATPM(j) = col + spATPM(j);
0331     %size of the network
0332     row = row + rowJ;
0333     col = col + colJ;
0334 end
0335 
0336 %% Community metabolites
0337 metsCom = sort(unique(metsCom));
0338 %Ids of exchange reactions corresponding to community metabolites
0339 % [a_ij] = exchange reaction Id for community metabolite i and species j
0340 EXsp = zeros(numel(metsCom), nSp);
0341 Msp = zeros(numel(metsCom), nSp);
0342 [rowS,colS,entryS] = deal([]);
0343 for kSp = 1:nSp
0344     %organism-community exchange reactions
0345     [r0,c0,e0] = find(modelCell{kSp}.S);
0346     modelCell{kSp}.metComs(cellfun(@isempty,modelCell{kSp}.metComs)) = {''};
0347     [yn,id] = ismember(modelCell{kSp}.metComs,metsCom);
0348     rowS = [rowS; id(yn)];
0349     colS = [colS; rxnEx2met{kSp}(yn,1)];
0350     [yn2,id2] = ismember([find(yn),rxnEx2met{kSp}(yn,2)],[r0,c0],'rows');
0351     entryS = [entryS; -e0(id2)];
0352     EXsp(id(yn),kSp) = rxnEx2met{kSp}(yn,1);
0353     Msp(id(yn),kSp) = find(metSps == kSp, 1) - 1 + find(yn);
0354 end
0355 %community uptake/export reactions
0356 rowS = [rowS; repmat((1:numel(metsCom))',2,1)];
0357 colS = [colS; ((col + 1):(col + 2*numel(metsCom)))'];
0358 entryS = [entryS; ones(numel(metsCom),1); -ones(numel(metsCom),1)];
0359 SmetCom = sparse(rowS,colS,entryS,numel(metsCom),col+2*numel(metsCom));
0360 % %new submatrix for balancing community metabolites
0361 % SmetCom = sparse(numel(metsCom), col + 2 * numel(metsCom));
0362 % for j = 1:numel(metsCom)
0363 %     for kSp = 1:nSp
0364 %         %for each community metabolite, for each species, find the row of
0365 %         %the corresponding extracellular metabolite
0366 %         metJK = find(strcmp(modelCell{kSp}.metComs, metsCom{j}),1);
0367 %         if ~isempty(metJK) %if it is found
0368 %             %update the stoichiometric matrix
0369 %             SmetCom(j, rxnEx2met{kSp}(metJK, 1)) = - modelCell{kSp}.S(metJK, rxnEx2met{kSp}(metJK, 2));
0370 %             EXsp(j, kSp) = rxnEx2met{kSp}(metJK, 1);
0371 %         end
0372 %     end
0373 %     %add two reactions for uptake of and export from the community.
0374 %     SmetCom(j, [col + j, col + numel(metsCom) + j]) = [1 -1];
0375 % end
0376 
0377 S = [S sparse([],[],[], row, 2*numel(metsCom)); SmetCom];
0378 b = [b; zeros(numel(metsCom),1)];
0379 %For non-limiting substrate for uptake, if the lower bound for uptake is high (say
0380 % 1000), then the upper bound of the corresponding export reaction of the
0381 % community metabolites should be significantly larger (say 10000) in order
0382 % not to overconstrain the community in the way that it is not allowed to
0383 % produce those metabolites
0384 ub = [ub; zeros(numel(metsCom), 1); 10000 * ones(numel(metsCom),1)];
0385 lb = [lb; zeros(2*numel(metsCom),1)];
0386 c = [c;  zeros(2*numel(metsCom),1)];
0387 rev = [rev;  zeros(2*numel(metsCom),1)];
0388 rxnSps = [rxnSps; zeros(2*numel(metsCom),1)];
0389 metSps = [metSps; zeros(numel(metsCom),1)];
0390 
0391 %names of community metabolites
0392 mets = [mets; strcat(metsCom,'[u]')];
0393 %names of community exchange reactions
0394 rxns = [rxns; strcat('UT_',metsCom,'(u)'); strcat('EX_',metsCom,'(u)')];
0395 
0396 [modelCom.rxns, modelCom.mets, modelCom.S, modelCom.c, modelCom.lb, ...
0397     modelCom.ub, modelCom.b, modelCom.rev] =...
0398     deal(rxns, mets, S, c, lb, ub, b, rev);
0399 %get community reaction indices
0400 indCom = struct();
0401 
0402 rxnSD = sum(modelCom.S ~= 0, 1) <= 1;
0403 rxnSD((col + 1) : (col + 2*numel(metsCom))) = false;
0404 rxnSD = find(rxnSD);
0405 %reaction Ids for [uptake | export] of community metabolites
0406 EXcom = [(col + 1: col + numel(metsCom))' ...
0407                     (col + numel(metsCom) + 1: col + 2 * numel(metsCom))'];
0408 [indCom.spBm, indCom.spATPM, indCom.rxnSD, indCom.EXcom, indCom.EXsp,...
0409     indCom.Mcom, indCom.Msp, indCom.rxnSps, indCom.metSps] = deal(...
0410     spBmId, spATPM, rxnSD, EXcom, EXsp, ...
0411     ((row + 1) : (row + numel(metsCom)))', Msp, rxnSps, metSps);
0412 
0413 
0414 %add rxnNames if exist
0415 if ~isfield(modelCom, 'rxnNames')
0416     modelCom.rxnNames = modelCom.rxns;
0417 else
0418     if numel(modelCom.rxnNames) ~= col
0419         modelCom.rxnNames(col + 1: col + 2*numel(metsCom)) = modelCom.rxns(col + 1: col + numel(metsCom)*2);
0420     else
0421         for j = 1:numel(metsCom)
0422             rxnNameLength = zeros(nSp, 1);
0423             for k = 1:nSp
0424                 if EXsp(j,k) > 0
0425                     rxnNameLength(k) = length(modelCom.rxnNames{EXsp(j,k)});
0426                 end
0427             end
0428             [maxLength, maxLengthId] = max(rxnNameLength);
0429             if maxLength > 0
0430                 rxnNameJ = modelCom.rxnNames{EXsp(j,maxLengthId)};
0431                 if ~isempty(strfind(rxnNameJ, 'exchange'))
0432                     rxnNameJut = strrep(rxnNameJ, 'exchange', '(community uptake)');
0433                     rxnNameJex = strrep(rxnNameJ, 'exchange', '(community export)');
0434                 else
0435                     rxnNameJut = strcat(rxnNameJ, ' (community uptake)');
0436                     rxnNameJex = strcat(rxnNameJ, ' (community export)');
0437                 end
0438                 modelCom.rxnNames{col + j} = rxnNameJut;
0439                 modelCom.rxnNames{col + numel(metsCom) + j} = rxnNameJex;
0440             else
0441                 modelCom.rxnNames{col + j} = modelCom.rxns{col + j};
0442                 modelCom.rxnNames{col + numel(metsCom) + j} = modelCom.rxns{col + numel(metsCom) + j};
0443             end
0444         end
0445     end
0446 end
0447 %add metNames, metFormulas and other metField if exist
0448 if ~isfield(modelCom, 'metFormulas')
0449     modelCom.metFormulas = repmat({''}, row + numel(metsCom), 1);
0450 end
0451 if ~isfield(modelCom, 'metNames')
0452     modelCom.metNames = modelCom.mets;
0453 else
0454     if numel(modelCom.metNames) ~= row
0455         modelCom.metNames(row + 1: row + numel(metsCom)) = modelCom.mets(row + 1: row + numel(metsCom));
0456     else
0457         for j = 1:numel(metsCom)
0458 %             metNameLength = zeros(nSp, 1);
0459             metNameId = zeros(nSp, 1);
0460             metNameJ = {};
0461             metForm = '';
0462             %get all names, put in cell array
0463             for k = 1:nSp
0464                 if EXsp(j,k) > 0
0465                     metEUid = modelCom.S(:,EXsp(j,k)) ~= 0;
0466                     metEUid(row + j) = false;
0467                     metNameId(k) = find(metEUid, 1);
0468                     metNameJK = modelCom.metNames{metNameId(k)};
0469                     metNameJK = strrep(strrep(metNameJK, '(extracellular)', ''), '(Extracellular)', '');
0470                     metNameJK = strrep(strrep(metNameJK, 'extracellular', ''), 'Extracellular', '');
0471                     metNameJK = unique(strtrim(splitString(strtrim(metNameJK),';')))';
0472                     metNameJ = [metNameJ metNameJK];
0473                     if isfield(modelCom, 'metFormulas') && isempty(metForm)
0474                         metForm = strtrim(modelCom.metFormulas{metNameId(k)});
0475                     end
0476                 end
0477             end
0478             modelCom.metFormulas{row + j} = metForm;
0479             metNameJ = unique(metNameJ);
0480             %remove those are contained totally in another name
0481             %             k = 1;
0482             %             while k <= numel(metNameJ)
0483             %                 if any(cellfun(@(x) ~isempty(strfind(x, lower(metNameJ{k}))), lower(metNameJ([1:k-1 k+1:end]))))
0484             %                     %if totally contained, delete it
0485             %                     metNameJ(k) = [];
0486             %                 else
0487             %                     k = k + 1;
0488             %                 end
0489             %             end
0490             metNameLength = cellfun(@length,metNameJ);
0491             if ~isempty(metNameLength)
0492                 metNameJ = metNameJ(metNameLength > 0);
0493             end
0494             if ~isempty(metNameJ)
0495                 if iscell(metNameJ) && numel(metNameJ) == 1
0496                     modelCom.metNames{row + j} = metNameJ{1};
0497                 else
0498                     modelCom.metNames{row + j} = strjoin(metNameJ,'|');
0499                 end
0500             else
0501                 modelCom.metNames{row + j} = modelCom.mets{row + j};
0502             end
0503             for kF = 1:numel(metField)
0504                 modelCom.(metField{kF})(row + j) = modelCom.(metField{kF})(metEUid);
0505             end
0506                 
0507 %             for k = 1:nSp
0508 %                 if modelCom.EXsp(j,k) > 0
0509 %                     metEUid = modelCom.S(:,modelCom.EXsp(j,k)) ~= 0;
0510 %                     metEUid(row + j) = false;
0511 %                     metNameId(k) = find(metEUid, 1);
0512 %                     metNameLength(k) = length(modelCom.metNames{metNameId(k)});
0513 %                 end
0514 %             end
0515 %             [maxLength, maxLengthId] = max(metNameLength);
0516 %             if maxLength > 0
0517 %                 modelCom.metNames{row + j} = modelCom.metNames{metNameId(maxLengthId)};
0518 %             else
0519 %                 modelCom.metNames{row + j} = modelCom.mets{row + j};
0520 %             end
0521         end
0522     end
0523 end
0524 if isfield(modelCom,'subSystems')
0525     modelCom.subSystems(col + 1: col + numel(metsCom)*2) = repmat({'community exchange'},2*numel(metsCom),1);
0526 end
0527 %extend all other fields
0528 uMet = zeros(numel(metsCom),1);
0529 for jM = 1:numel(metsCom)
0530     uMet(jM) = Msp(jM,find(Msp(jM,:),1));
0531 end
0532 for j = 1:numel(field)
0533     if metFieldL(j) || rxnFieldL(j)
0534         if metFieldL(j) && ~any(strcmp({'metNames','metFormulas'},field{j}))
0535             v = row + 1: row + numel(metsCom);
0536             modelCom.(field{j})(v,:) = modelCom.(field{j})(uMet,:);
0537         elseif rxnFieldL(j) && ~any(strcmp({'subSystems','rxnNames'},field{j}))
0538             v = col + 1: col + numel(metsCom)*2;
0539             if fieldNumeric(j)
0540                 u = 0;
0541             elseif fieldCell(j)
0542                 u = {''};
0543             elseif fieldStruct(j)
0544                 f = fieldnames(modelCom.(field{j}));
0545                 u = cell2struct(repmat({[]},numel(f),1),f);
0546             end
0547             modelCom.(field{j})(v,:) = u;
0548         end
0549     end
0550 end
0551 %get community info
0552 infoCom = infoCom2indCom(modelCom,indCom,true,spAbbr,spName);
0553 
0554 %special care for genes and rxnGeneMat
0555 [rGMrow,rGMcol,rGMent] = deal([]);
0556 rGMm = 0;
0557 rGMn = 0;
0558 genes = {};
0559 for jSp = 1:nSp
0560     if ~isfield(modelCell{jSp},'genes')
0561         modelCell{jSp}.genes = {};
0562     end
0563     if ~isfield(modelCell{jSp},'rxnGeneMat')
0564         modelCell{jSp}.rxnGeneMat = sparse(size(modelCell{jSp}.S,2),numel(modelCell{jSp}.genes));
0565     end
0566     if size(modelCell{jSp}.rxnGeneMat,1) ~= size(modelCell{jSp}.S,2)
0567         warning('#%d (%s): No. of rows in rxnGeneMat not equal to the number of reactions.',jSp,modelCom.sps{jSp});
0568     end
0569     if numel(modelCell{jSp}.genes) < size(modelCell{jSp}.rxnGeneMat,2)
0570         warning('#%d (%s): No. of columns in rxnGeneMat not equal to the number of genes.',jSp,modelCom.sps{jSp});
0571         modelCell{jSp}.genes(end+1:size(modelCell{jSp}.rxnGeneMat,2)) = {''};
0572     elseif numel(modelCell{jSp}.genes) > size(modelCell{jSp}.rxnGeneMat,2)
0573         warning('#%d (%s): No. of columns in rxnGeneMat not equal to the number of genes.',jSp,modelCom.sps{jSp});
0574         modelCell{jSp}.rxnGeneMat(:,end+1:numel(modelCell{jSp}.genes)) = 0;
0575     end
0576     genes = [genes; modelCell{jSp}.genes(:)];
0577     [rGMrowJ,rGMcolJ,rGMentJ] = find(modelCell{jSp}.rxnGeneMat);
0578     rGMrow = [rGMrow; (rGMrowJ+rGMm)];
0579     rGMcol = [rGMcol; (rGMcolJ+rGMn)];
0580     rGMent = [rGMent; rGMentJ];
0581     rGMm = rGMm + size(modelCell{jSp}.rxnGeneMat,1);
0582     rGMn = rGMn + size(modelCell{jSp}.rxnGeneMat,2);
0583 end
0584 modelCom.genes = genes;
0585 modelCom.rxnGeneMat = sparse(rGMrow,rGMcol,rGMent,size(modelCom.S,2),rGMn);
0586 % add infoCom and indCom into modelCom
0587 modelCom.infoCom = infoCom;
0588 modelCom.indCom = indCom;
0589 end
0590     
0591
```

---

Generated on Sat 06-May-2017 09:55:30 by **m2html** © 2005
