# Supplementary material for: SteadyCom: Predicting microbial abundances while ensuring community stability
Source: PLoS Comput Biol. 2017 May 15;13(5):e1005539. doi: 10.1371/journal.pcbi.1005539 (PMC5448816; doi:10.1371/journal.pcbi.1005539)
Supplement: S1 Dataset — (ZIP) [file pcbi.1005539.s018.zip › S1 Dataset/SteadyCom/doc/SteadyCom/menu.html]

Index for Directory SteadyCom


 Master index 

# Index for SteadyCom

## Matlab files in this directory:

- SteadyComCplex
- SteadyComFVACplex
- SteadyComPOACplex
- createCommModel

## Subsequent directories:

- .git
- auxiliary\_functions
- doc

---

Generated by **m2html** © 2005
