# Supplementary material for: SteadyCom: Predicting microbial abundances while ensuring community stability
Source: PLoS Comput Biol. 2017 May 15;13(5):e1005539. doi: 10.1371/journal.pcbi.1005539 (PMC5448816; doi:10.1371/journal.pcbi.1005539)
Supplement: S1 Dataset — (ZIP) [file pcbi.1005539.s018.zip › S1 Dataset/SteadyCom/doc/SteadyCom/SteadyComCplex.html]

Description of SteadyComCplex


# SteadyComCplex

## PURPOSE

**Find the maximum community growth rate at community steady-state using SteadyCom**

## SYNOPSIS

**function [sol, result, LP, LP2,indLP] = SteadyComCplex(modelCom,options, solverParam,LP)**

## DESCRIPTION

```
Find the maximum community growth rate at community steady-state using SteadyCom
Call the CPLEX dynamic object directly.

[sol, result, LP,LP2,indLP] = SteadyComCplex(modelCom,options, solverParam)

INPUT
 modelCom   community COBRA model created with crateCommModel.m 
 (the following fields are required - others can be supplied)
   S            Stoichiometric matrix
   b            Right hand side
   c            Objective coefficients
   lb           Lower bounds
   ub           Upper bounds
 (at least one of the below two is needed)
   infoCom      structure containing community reaction info 
   indCom       the index structure corresponding to infoCom
                (returned along with the community model created with createCommModel) 

 options (optional)   struct with the following possible fields:
 (for constraining individual growth rates and biomass amounts, default [])
   GRfx            Fixed growth rate for species apart from the community
                   (N_species x 1 vector, NaN for unfixed growth rate,
                    or [#species | value]) e.g. to fix species 2, 3 
                   at growth rate 0.1, GRfx = [2, 0.1; 3, 0.1];
   BMcon           Biomass constraint matrix (sum(a_ij * X_j) </=/> b_i)
                   (given as K x N_species matrix for K constraints)
                   e.g. [0 1 1 0] for X_2 + X_3 in a 4-species model
   BMrhs           RHS for BMcon, K x 1 vector for K constraints
   BMcsense        Sense of the constraint, 'L', 'E', 'G' for <=, =, >=
 (for general constraints on e.g. total carbon uptake, molecular crowding, default [])
   MC              K x (N_rxns+N_species) coefficient matrix, for K additional constraints
   MCmode          K x (N_rxns+N_species) matrix , with number 0 ~ 3
                   0: original variable
                   1: positive part of the variable
                   2: negative part of the variable
                   3: absolute value of the variable
   MCrhs           RHS of the constraints (optional, default all zeros)
   MClhs           LHS of the constraints (optional, default -inf)
 (parameters in the iterative algorithm, [default value])
   GRguess [0.2]   Initial guess of the growth rate.
   feasCrit [1]    Criteria for feasibility, 1, 2 or 3.
          The algorithm tests iteratively at a given growth rate
          whether a feasible solution can be found.
           1: Use a threshold total biomass BMweight (see below).
              i.e. sum(X) >= BMweight
              (use it if the total biomass is known)
           2: Use a threshold on minimum biomass production
              (=specific growth rate x sum(biomass), which is roughly 
              constant over a range of growth rate if the sum of biomass 
              is not bounded above) 
              i.e. sum(X) * gr >= BMtol * BMref * GR0
              where BMref is the maximum biomass at a small growth rate GR0
              and BMtol is a fraction ranging from 0 to 1
   algorithm [1]   algorithm to find the maximum growth rate
           1. Fzero after finding grLB and grUB with simple guessing [gr' = gr * sum(X)/sum(X')]
           2. Simple guessing with minimum one percent step size
           3. Bisection method
   BMweight [1]    Minimum total biomass for feasibility. Used only if feasCrit = 1
                   Set BMweight to a close-to-zero value to compute the
                   wash-out dilution rate.
   GR0 [0.001]     A small growth rate to obtain a reference value for
                   maximum total biomass production. 
                   Used only if feasCrit = 2 or solveGR0 = true
   BMtol [0.8]     Fractional tolerance for biomass production to check
                   feasibility. Used only if feasCrit = 2
   solveGR0[false] true to first solve the model at a low growth rate GR0
   GRtol [1e-6]    Precision for the growth rate found (grUB - grLB < GRtol)
   BMtolAbs [1e-5] Absolute tolerance for positivity of biomass
   maxIter (1e3)   maximum nummber of iteration
 (parameters in the optimization model, [default value])
   minNorm [0]     0: No minNorm. 1: min sum of absolution flux of the final solution.
   BMgdw [all 1s]  The gram dry weight per mmol of the biomass reaction of
                   each organism. Maybe used to scale the biomass reactions between species.
   BMobj [all 1s]  Objective coefficient for the biomass of each species
                   when doing the maximization at each step. Maybe used to
                   scale the biomass reactions between species.
 (other parameters)
   verbFlag  [3]   Print level
                   0, 1, 2, 3 for silence, one log per 10, 5 (default) or 1
                   iteration respectively
   LPonly [false]  Return the initial LP at zero growth rate only. Calculate nothing.
   saveModel ['']  String, if non-empty, save the cplex model, basis and parameters.

 solverParam       Cplex parameter structure. E.g., struct('simplex',struct('tolerances',struct('feasibility',1e-8)))

OUTPUT
 sol: cplex solution structure
 result: structure with the following fields:
   GRmax:          maximum specific growth rate found (/h)
   vBM:            biomass formation rate (gdw/h)
   BM:             Biomass vector at GRmax (gdw)
   Ut:             uptake fluxes (mmol/h)
   Ex:             export fluxes (mmol/h)
   flux:           flux distribution for the original model
   (the following 'iter' fields are status in each iteration:)
   [GR | biomass X | biomass flux (GR * X) | max. infeas. of solution])
   iter0:          stationary, no growth, gr = 0
   iter1:          small growth rate, gr = GR0
   iterPre:        iterations for finding upper and lower bounds
   iter:           iterations for finding max gr using bisectional method
   stat:           status at the termination of the algorithm
                   infeasible: infeasible model, even with maintenance
                               requirement only
                   maintenance:feasible at maintenance, but cannot grow
                   optimal:    optimal growth rate found
```

## CROSS-REFERENCE INFORMATION

This function calls:

- checkSolFeas Check the feasibility of a solution given a COBRA model structure or a CPLEX dynamic object and a solution
- getCobraComParams get the required default parameters
- infoCom2indCom Transform between community reaction IDs and reaction names
- setCplexParam Set the parameters of the CPLEX object according to the structure solverParam
- updateLPcom Create and update the SteadyCom LP model in CPLEX format.

This function is called by:

- SteadyComFVACplex Flux variability analysis for community model at community steady-state for a range of growth rates.
- SteadyComPOACplex Pairwise POA for community model at community steady-state for a range of growth rates
- SteadyComFVAgrCplex Flux variability analysis for community model at community steady-state at a given growth rate.
- SteadyComPOAgrCplex Pairwise POA for community model at community steady-state at a given growth rate

## SUBFUNCTIONS

- function [LP,index] = constructLPcom(modelCom, options, solverParam)
- function LPproblem = updateLPcom(modelCom, grCur, GRfx, BMcon, LPproblem, BMgdw)
- function [paramList, paramPath] = getParamList(param, bottomFlag)
- function dBM = LP4fzero1(grCur, LP, modelCom, GRfx, feasTol, BMequiv,BMgdw)
- function dBM = LP4fzero2(grCur, LP, modelCom, GRfx, feasTol, BMequiv, GR0, BMgdw)
- function yn = ErrBecauseInfeas(ME)

## SOURCE CODE

```
0001 function [sol, result, LP, LP2,indLP] = SteadyComCplex(modelCom,options, solverParam,LP)
0002 %Find the maximum community growth rate at community steady-state using SteadyCom
0003 %Call the CPLEX dynamic object directly.
0004 %
0005 %[sol, result, LP,LP2,indLP] = SteadyComCplex(modelCom,options, solverParam)
0006 %
0007 %INPUT
0008 % modelCom   community COBRA model created with crateCommModel.m
0009 % (the following fields are required - others can be supplied)
0010 %   S            Stoichiometric matrix
0011 %   b            Right hand side
0012 %   c            Objective coefficients
0013 %   lb           Lower bounds
0014 %   ub           Upper bounds
0015 % (at least one of the below two is needed)
0016 %   infoCom      structure containing community reaction info
0017 %   indCom       the index structure corresponding to infoCom
0018 %                (returned along with the community model created with createCommModel)
0019 %
0020 % options (optional)   struct with the following possible fields:
0021 % (for constraining individual growth rates and biomass amounts, default [])
0022 %   GRfx            Fixed growth rate for species apart from the community
0023 %                   (N_species x 1 vector, NaN for unfixed growth rate,
0024 %                    or [#species | value]) e.g. to fix species 2, 3
0025 %                   at growth rate 0.1, GRfx = [2, 0.1; 3, 0.1];
0026 %   BMcon           Biomass constraint matrix (sum(a_ij * X_j) </=/> b_i)
0027 %                   (given as K x N_species matrix for K constraints)
0028 %                   e.g. [0 1 1 0] for X_2 + X_3 in a 4-species model
0029 %   BMrhs           RHS for BMcon, K x 1 vector for K constraints
0030 %   BMcsense        Sense of the constraint, 'L', 'E', 'G' for <=, =, >=
0031 % (for general constraints on e.g. total carbon uptake, molecular crowding, default [])
0032 %   MC              K x (N_rxns+N_species) coefficient matrix, for K additional constraints
0033 %   MCmode          K x (N_rxns+N_species) matrix , with number 0 ~ 3
0034 %                   0: original variable
0035 %                   1: positive part of the variable
0036 %                   2: negative part of the variable
0037 %                   3: absolute value of the variable
0038 %   MCrhs           RHS of the constraints (optional, default all zeros)
0039 %   MClhs           LHS of the constraints (optional, default -inf)
0040 % (parameters in the iterative algorithm, [default value])
0041 %   GRguess [0.2]   Initial guess of the growth rate.
0042 %   feasCrit [1]    Criteria for feasibility, 1, 2 or 3.
0043 %          The algorithm tests iteratively at a given growth rate
0044 %          whether a feasible solution can be found.
0045 %           1: Use a threshold total biomass BMweight (see below).
0046 %              i.e. sum(X) >= BMweight
0047 %              (use it if the total biomass is known)
0048 %           2: Use a threshold on minimum biomass production
0049 %              (=specific growth rate x sum(biomass), which is roughly
0050 %              constant over a range of growth rate if the sum of biomass
0051 %              is not bounded above)
0052 %              i.e. sum(X) * gr >= BMtol * BMref * GR0
0053 %              where BMref is the maximum biomass at a small growth rate GR0
0054 %              and BMtol is a fraction ranging from 0 to 1
0055 %   algorithm [1]   algorithm to find the maximum growth rate
0056 %           1. Fzero after finding grLB and grUB with simple guessing [gr' = gr * sum(X)/sum(X')]
0057 %           2. Simple guessing with minimum one percent step size
0058 %           3. Bisection method
0059 %   BMweight [1]    Minimum total biomass for feasibility. Used only if feasCrit = 1
0060 %                   Set BMweight to a close-to-zero value to compute the
0061 %                   wash-out dilution rate.
0062 %   GR0 [0.001]     A small growth rate to obtain a reference value for
0063 %                   maximum total biomass production.
0064 %                   Used only if feasCrit = 2 or solveGR0 = true
0065 %   BMtol [0.8]     Fractional tolerance for biomass production to check
0066 %                   feasibility. Used only if feasCrit = 2
0067 %   solveGR0[false] true to first solve the model at a low growth rate GR0
0068 %   GRtol [1e-6]    Precision for the growth rate found (grUB - grLB < GRtol)
0069 %   BMtolAbs [1e-5] Absolute tolerance for positivity of biomass
0070 %   maxIter (1e3)   maximum nummber of iteration
0071 % (parameters in the optimization model, [default value])
0072 %   minNorm [0]     0: No minNorm. 1: min sum of absolution flux of the final solution.
0073 %   BMgdw [all 1s]  The gram dry weight per mmol of the biomass reaction of
0074 %                   each organism. Maybe used to scale the biomass reactions between species.
0075 %   BMobj [all 1s]  Objective coefficient for the biomass of each species
0076 %                   when doing the maximization at each step. Maybe used to
0077 %                   scale the biomass reactions between species.
0078 % (other parameters)
0079 %   verbFlag  [3]   Print level
0080 %                   0, 1, 2, 3 for silence, one log per 10, 5 (default) or 1
0081 %                   iteration respectively
0082 %   LPonly [false]  Return the initial LP at zero growth rate only. Calculate nothing.
0083 %   saveModel ['']  String, if non-empty, save the cplex model, basis and parameters.
0084 %
0085 % solverParam       Cplex parameter structure. E.g., struct('simplex',struct('tolerances',struct('feasibility',1e-8)))
0086 %
0087 %OUTPUT
0088 % sol: cplex solution structure
0089 % result: structure with the following fields:
0090 %   GRmax:          maximum specific growth rate found (/h)
0091 %   vBM:            biomass formation rate (gdw/h)
0092 %   BM:             Biomass vector at GRmax (gdw)
0093 %   Ut:             uptake fluxes (mmol/h)
0094 %   Ex:             export fluxes (mmol/h)
0095 %   flux:           flux distribution for the original model
0096 %   (the following 'iter' fields are status in each iteration:)
0097 %   [GR | biomass X | biomass flux (GR * X) | max. infeas. of solution])
0098 %   iter0:          stationary, no growth, gr = 0
0099 %   iter1:          small growth rate, gr = GR0
0100 %   iterPre:        iterations for finding upper and lower bounds
0101 %   iter:           iterations for finding max gr using bisectional method
0102 %   stat:           status at the termination of the algorithm
0103 %                   infeasible: infeasible model, even with maintenance
0104 %                               requirement only
0105 %                   maintenance:feasible at maintenance, but cannot grow
0106 %                   optimal:    optimal growth rate found
0107 %
0108 %
0109 t = tic;
0110 t0 = 0;
0111 %% Initialization
0112 %check required fields for community model
0113 if ~isfield(modelCom,'indCom')
0114     if ~isfield(modelCom,'infoCom') || ~isstruct(modelCom.infoCom) || ...
0115             ~all(isfield(modelCom.infoCom,{'spBm','EXcom','EXsp','spAbbr','rxnSps','metSps'}))
0116         error('infoCom must be provided for calculating the max. community growth rate.\n');
0117     end
0118     %get useful reaction indices
0119     modelCom.indCom = infoCom2indCom(modelCom);
0120 end
0121 
0122 %get paramters
0123 if ~exist('options', 'var')
0124     options = struct();
0125 end
0126 if ~exist('solverParam', 'var') || isempty(solverParam)
0127     %default Cplex parameters
0128     solverParam = getCobraComParams('CplexParam');
0129 end
0130 param2get = {'GRguess', 'GR0', 'GRfx', 'GRtol', 'solveGR0',...
0131              'BMweight', 'BMtol', 'BMtolAbs', 'BMgdw',...
0132              'feasCrit', 'maxIter', 'verbFlag', 'algorithm',...
0133              'minNorm', 'LPonly', 'saveModel'};
0134 eval(sprintf('[%s] = getCobraComParams(param2get, options, modelCom);', ...
0135             strjoin(param2get, ',')...
0136             )...
0137     );
0138 %print level
0139 pL = [0 10 5 1];
0140 pL = pL(verbFlag + 1);
0141 
0142 [m, n] = size(modelCom.S); %model size
0143 nRxnSp = sum(modelCom.indCom.rxnSps > 0); %number of organism-specific rxns
0144 nSp = numel(modelCom.indCom.spBm); %number of organism
0145 
0146 if verbFlag && ~LPonly
0147     fprintf('Find maximum community growth rate..\n');
0148 end
0149 %% Construct LP
0150 
0151 if nargin < 4
0152     %create the CPLEX LP problem if not given
0153     [LP,indLP] = constructLPcom(modelCom, options, solverParam);
0154 else
0155     % LP given: delete the row constraining the sum of biomass if exist
0156     f = find(strcmp(cellstr(LP.Model.rowname),'UnityBiomass'));
0157     if ~isempty(f)
0158         LP.delRows(f);
0159     end
0160     LP.Model.obj(n+1:n+nSp) = 1;
0161     LP.Model.sense = 'maximize';
0162     indLP = [];
0163 end
0164 % Make sure the feasibility tolerance used in CPLEX and in the main loop
0165 % are the same ('constructLPcom' has already reconciled the two tolerances)
0166 feasTol = LP.Param.simplex.tolerances.feasibility.Cur;
0167 LP2 = [];
0168 % terminate if only the LP structure is called as output
0169 if LPonly
0170     result = struct();
0171     [result.GRmax, result.vBM, result.BM, result.Ut, result.Ex, ...
0172         result.flux, result.iter, result.iter0, sol] = deal([]);
0173     result.stat = 'LPonly';
0174     return
0175 end
0176 
0177 %counter for iteration
0178 k = 0;
0179 iter = [];
0180 
0181 % if LP is supplied by user, directly jump to the main loop
0182 if nargin < 4
0183     %% Test the ability of the model to stay at maintenance only.
0184     
0185     %solve for maintenance (zero growth)
0186     %This step usually costs very little time. Worth doing to confirm
0187     %feasibility
0188     feas = true;
0189     try
0190         LP.solve();
0191     catch ME
0192         %possible internal error of cplex
0193         if ErrBecauseInfeas(ME)
0194             %treat as infeasible
0195             feas = false;
0196         else
0197             disp(ME);
0198             error('Unknown error from CPLEX.');
0199         end
0200     end
0201         
0202     % check the feasibility of the solution manually
0203     dev = checkSolFeas(LP);
0204 
0205     result = struct();
0206     [result.GRmax, result.vBM, result.BM, result.Ut, result.Ex, result.flux, ...
0207         result.iter0, result.iter, result.stat] = deal([]);
0208     %terminate if time limit has been exceeded.
0209     if feas && LP.Solution.status == 11
0210         result.stat = 'time limit exceeded';
0211         sol = [];
0212         return
0213     end
0214     %biomass at zero growth rate
0215     BM0 = 0;
0216     if feas && isfield(LP.Solution, 'x') && dev <= feasTol
0217         if ~any(isnan(LP.Solution.x))
0218             %if feasible
0219             BM0 = LP.Model.obj' * LP.Solution.x;
0220         end
0221     end
0222     if BM0 < BMtolAbs
0223         %if no biomass is formed, infeasible. Terminate.
0224         if verbFlag
0225             t0 = toc(t);
0226             fprintf('Model infeasible at maintenance. Time elapsed: %.0f / %.0f sec\n', t0, t0);
0227         end
0228         sol = [];
0229         result.stat = 'infeasible';
0230         LP2 = [];
0231         return
0232     else
0233         %record the current result if feasible
0234         if verbFlag
0235             t0 = toc(t);
0236             fprintf('Model feasible at maintenance. Time elapsed: %.0f / %.0f sec\n', t0, t0);
0237         end
0238         sol = LP.Solution;
0239         if ~isempty(saveModel)
0240             LP.writeBasis([saveModel '.bas']);
0241         end
0242         result.GRmax = 0;
0243         result.vBM = LP.Solution.x(modelCom.indCom.spBm);
0244         result.BM = LP.Solution.x(n + 1 : n + nSp);
0245         result.BM(abs(result.BM) < 1e-8) = 0;
0246         result.Ut = LP.Solution.x(modelCom.indCom.EXcom(:,1));
0247         result.Ex = LP.Solution.x(modelCom.indCom.EXcom(:,2));
0248         result.flux = LP.Solution.x(1:n);
0249         result.iter0 = [0 BM0 0 dev];
0250         result.iter = [];
0251         result.stat = 'maintenance';
0252     end
0253 
0254     %% Test at very small growth rate to see if the model is able to grow
0255     % only if using the reference biomass at GR0 to define maximum growth rate
0256     if feasCrit == 2 || solveGR0
0257         %update the growth rate
0258         LP.Model.A =updateLPcom(modelCom, GR0, GRfx, [], LP.Model.A, BMgdw);
0259         feas = true;
0260         try
0261             LP.solve();
0262         catch ME
0263             if ErrBecauseInfeas(ME)
0264                 %treat as infeasible
0265                 feas = false;
0266             else
0267                 disp(ME);
0268                 error('Unknown error from CPLEX.');
0269             end
0270         end
0271         if feas && LP.Solution.status == 11
0272             result.stat = 'time limit exceeded';
0273             sol = [];
0274             LP2 = [];
0275             return
0276         end
0277         % check the feasibility of the solution manually
0278         dev = checkSolFeas(LP);
0279         %biomass for reference (at a very low growth rate)
0280         BMref = 0;
0281         if feas && isfield(LP.Solution, 'x') && dev <= feasTol
0282             if ~any(isnan(LP.Solution.x))
0283                 BMref = LP.Model.obj' * LP.Solution.x;
0284             end
0285         end
0286 
0287         iter = [iter; 0 GR0 BMref GR0 * BMref dev 0];
0288         if BMref < BMtolAbs
0289             %if no biomass can be formed, the model can only stay at maintenance.
0290             if verbFlag
0291                 t1 = toc(t);
0292                 fprintf('Model infeasible at a minimal growth rate (%.6f). Time elapsed: %.0f / %.0f sec\n.', GR0, t1 - t0, t1);
0293             end
0294             result.iter = iter;
0295             return
0296         else
0297             %able to grow. Compute bounds
0298             if verbFlag
0299                 t1 = toc(t);
0300                 fprintf('Model feasible at a minimal growth (%.6f). Time elapsed: %.0f / %.0f sec.\nLook for upper and lower bounds...\n', GR0, t1 - t0, t1);
0301                 t0 = t1;
0302             end
0303             sol = LP.Solution;
0304             if ~isempty(saveModel)
0305                 LP.writeBasis([saveModel '.bas']);
0306             end
0307             result.GRmax = GR0;
0308             result.vBM = LP.Solution.x(modelCom.indCom.spBm);
0309             result.BM = LP.Solution.x(n + 1 : n + nSp);
0310             result.BM(abs(result.BM) < 1e-8) = 0;
0311             result.Ut = LP.Solution.x(modelCom.indCom.EXcom(:,1));
0312             result.Ex = LP.Solution.x(modelCom.indCom.EXcom(:,2));
0313             result.flux = LP.Solution.x(1:n);
0314             result.stat = 'minimal growth';
0315         end
0316     end
0317     %initial growth rate
0318     grCur = GRguess(1);
0319 else
0320     % if LP is given, assume it has a starting basis for the growth rate
0321     % encoded in the problem. Start from there should give quick
0322     % convergence
0323     jSpGrCur = find(isnan(GRfx),1);
0324     %initial growth rate
0325     grCur = full(abs(LP.Model.A(m + 2*nRxnSp + jSpGrCur, n + jSpGrCur)));
0326 end
0327 
0328 %% main loop to solve for maximum growth rate
0329 
0330 %feasibility criteria
0331 switch feasCrit
0332     %condition1 for determining the feasibility of the current growth rate
0333     %condition2 for ensuring the final feasibility after the max growth
0334     %rate is found
0335     case 1
0336         %maximum growth rate given a fixed total community biomass, defaulted to be 1
0337         BMequiv = BMweight;
0338         condition1 = @(BMcur, grCur) BMcur >= BMweight;
0339         condition2 = @(BMcur, grCur) BMcur >= BMweight * (1 - BMtolAbs);
0340         %guess for grCur
0341         updateGRguess = @(BMcur, grCur) grCur * BMcur / BMweight;
0342         LP4fzero = @(grCur, LP)...
0343             LP4fzero1(grCur, LP, modelCom, GRfx, feasTol, BMequiv, BMgdw);
0344     case 2
0345         %maximum growth rate with production rate of community biomass not
0346         %less than the reference value at growth rate GR0
0347         BMequiv = BMtol * BMref;
0348         condition1 = @(BMcur, grCur) BMcur * grCur >= BMtol * BMref * GR0;
0349         condition2 = @(BMcur, grCur) BMcur * grCur >= BMtol * BMref * GR0 * (1 - BMtolAbs);
0350         %guess for grCur
0351         updateGRguess = @(BMcur, grCur) grCur * BMcur / (BMtol * BMref * GR0);
0352         LP4fzero = @(grCur, LP)...
0353             LP4fzero2(grCur, LP, modelCom, GRfx, feasTol, BMequiv, GR0, BMgdw);
0354 end
0355 
0356 grLB = 0;%lower bound for growth rate
0357 grUB = Inf;%upper bound for growth rate
0358 grLBrecord = grLB;%vector recording all intermediate grLB
0359 grUBrecord = grUB;%vector recording all intermediate grUB
0360 guessMethod = 0; %guess used for updating the growth rate
0361 numInstab = false; %flag for numerical instability
0362 grUnstable = []; %growth rate at which numerical instability occurs
0363 optionsf0 = optimset; %matlab optimization parameters
0364 switch pL
0365     case 0
0366         optionsf0.Display = 'off';
0367     case 10
0368         optionsf0.Display = 'final';
0369     case 5
0370         optionsf0.Display = 'notify';
0371     case 1
0372         optionsf0.Display = 'iter';
0373 end
0374 optionsf0.MaxIter = maxIter; %max. number of iteration
0375 optionsf0.TolX = GRtol; %tolerance for the root found
0376 % optionsf0.TolFun = BMtolAbs;
0377 
0378 %Finding an interval for the max. growth rate using the simple guess
0379 %growth rate x max(biomass) = constant
0380 %apparently better than guess by matlab fzero
0381 %Then initiate fzero or continue using simple guess or bisection depending
0382 %on the parameter 'algorithm'
0383 col1disp = num2str(max([log10(maxIter)+1,4]));
0384 if pL
0385     fprintf(['%' col1disp 's  %8s  %8s  %8s  Time elapsed (iteration/total)\n'],...
0386         'Iter','LB','To test', 'UB');
0387 end
0388 if 0
0389     %totally solved by fzero (unused)
0390     GRmax = fzero(@(x) LP4fzero(x, LP), grCur, optionsf0);
0391 else
0392     k1LB = false; %lower bound found at k = 1
0393     %If an LB is found at k = 1, kLU counts the number of LBs found.
0394     %If an UB is found at k = 1, kLU counts the number of UBs found.
0395     kLU = 0; 
0396     while true
0397         %solve for initial guess
0398         k = k + 1;
0399         if mod(k, pL) == 0
0400             t1 = toc(t);
0401             if ~numInstab
0402                 fprintf(['%' col1disp 'd  %8.6f  %8.6f  %8.6f  %.0f / %.0f sec\n'],...
0403                     k, grLB, grCur, grUB, t1 - t0, t1);
0404             else
0405                 fprintf(['%' col1disp 'd  %8.6f  %8.6f  %8.6f  %.0f / %.0f sec (numerical instability)\n'],...
0406                     k, grLB, grCur, grUB, t1 - t0, t1);
0407                 numInstab = false;
0408             end
0409             %fprintf('%.0f\t%.6f\t%.6f\tTime elapsed: %.0f / %.0f sec\n', k, grLB, grUB, t1 - t0, t1);
0410             t0 = t1;
0411         end
0412         %update growth rate
0413         LP.Model.A = updateLPcom(modelCom, grCur, GRfx, [], LP.Model.A, BMgdw);
0414         feas = true;
0415         try
0416             LP.solve();
0417         catch ME
0418             if ErrBecauseInfeas(ME)
0419                 %treat as infeasible
0420                 feas = false;
0421             else
0422                 disp(ME);
0423                 error('Unknown error from CPLEX.');
0424             end
0425         end
0426         if feas && LP.Solution.status == 11
0427             result.stat = 'time limit exceeded';
0428             sol = [];
0429             LP2 = [];
0430             return
0431         end
0432         % check the feasibility of the solution manually
0433         dev = checkSolFeas(LP);
0434         %get biomass of the current iteration
0435         BMcur = 0;
0436         if feas && isfield(LP.Solution, 'x') && dev <= feasTol
0437             if ~any(isnan(LP.Solution.x))
0438                 %update the current biomass if successfully solved
0439                 BMcur = LP.Model.obj' * LP.Solution.x;
0440             end
0441             if condition1(BMcur, grCur)
0442                 %feasible at the current growth rate (sum(X) >= X_0)
0443                 grLB = grCur; %an LB is found
0444                 grLBrecord = [grLBrecord; grLB];
0445                 if k == 1
0446                     k1LB = true;
0447                 end
0448                 kLU = kLU + k1LB;
0449             else
0450                 %infeasible at the current growth rate (sum(X) < X_0)
0451                 grUB = grCur; %an UB is found
0452                 grUBrecord = [grUBrecord; grUB];
0453                 if k == 1
0454                     k1LB = false;
0455                 end
0456                 kLU = kLU + ~k1LB; 
0457             end
0458         else
0459             %No solution
0460             %(can become infeasible because of numerical instability)
0461             grUB = grCur;
0462             grUBrecord = [grUBrecord; grUB];
0463             if k == 1
0464                 k1LB = false;
0465             end
0466             kLU = kLU + ~k1LB;
0467         end
0468         %record results for the current iteration
0469         iter = [iter; k, grCur, BMcur, grCur * BMcur, dev, guessMethod];    
0470         
0471         % condition for switching to fzero or concluding GRmax = 0:
0472         %   kLU >= 2 to ensure neither of the bounds is the initial guess.
0473         % Algorithm:
0474         %   1. Fzero after finding LB and UB by simple guessing [gr' = gr * sum(X)/sum(X')]
0475         %   2. Simple guessing with minimum one percent step size
0476         %   3. Bisection method
0477         if (grLB > 0 && grUB < Inf && kLU >= 2 && algorithm == 1)
0478             %switch to fzero
0479             dBMneg = LP4fzero(grLB, LP);%expected to be -ve
0480             dBMpos = LP4fzero(grUB, LP);%expected to be +ve
0481             if isempty(dBMneg) || isempty(dBMpos)
0482                 result.stat = 'time limit exceeded';
0483                 sol = [];
0484                 LP2 = [];
0485                 return
0486             end
0487             %Check for numerical instability.
0488             % Can happens when the model is bounded such that the maximum growth rate
0489             % for the given biomass is close to the critical wash-out dilution rate
0490             % of the system. In this case, the maximum biomass sum(X) can drop very
0491             % abruptly with sum(X) ~ 0 at GRmax but sum(X) >> BMequiv at GRmax - eps.
0492             % Feasibility in this range returned by the solver is not trustworthy.
0493             % Should consider adjust the BMweight to a higher level. Or scan the whole
0494             % range of growth rate to see how it changes. (To be implemented)
0495             if dBMneg > 0 %the lower bound is indeed infeasible
0496                 dBMneg = LP4fzero(grLBrecord(end - 1), LP);
0497                 dBMpos = LP4fzero(grLB, LP);
0498                 grUnstable = [grUnstable; grLB];
0499                 numInstab = true; %unstable
0500                 %reset the bounds
0501                 if dBMpos > 0 %keep infeasible even optimizing again provided the previous lower bound basis
0502                     grUB = grLB;
0503                     grLB = 0;
0504                     grCur = (grLBrecord(end - 1) + grUB) / 2;
0505                     grUBrecord(end) = grUB;
0506                     grLBrecord(end) = grLB;
0507                     %loop until it becomes feasible again
0508                 else %can indeed feasible
0509                     %unstable solution
0510                     GRmax = grLB;
0511                     grUBrecord(end) = grUB;
0512                     grLBrecord(end) = grLB;
0513                     BMcur = BMequiv - dBMpos;
0514                     break
0515                 end
0516             elseif dBMpos < 0 %the upper bound is indeed feasible
0517                 GRmax = grUB;
0518                 grLB = grUB;
0519                 grUB = inf;
0520                 grUBrecord(end) = grUB;
0521                 grLBrecord(end) = grLB;
0522                 BMcur = BMequiv - dBMpos;
0523                 numInstab = true; %unstable
0524                 break
0525             else
0526                 % normal situation
0527                 % got interval, use fzero, LP will also be dynamically updated
0528                 % (Users may create a modified version of fzero on their own
0529                 % to supply function values [dBMneg, dBMpos] for the initial
0530                 % points to save the time for evaluting the initial points)
0531                 GRmax = fzero(@(x) LP4fzero(x, LP), [grLB, grUB], optionsf0);
0532                 %the final LP may not be at GRmax
0533                 dBM = LP4fzero(GRmax, LP);
0534                 BMcur = BMequiv - dBM;
0535                 break
0536             end
0537             
0538         elseif grUB <= GRtol %zero growth rate
0539             GRmax = 0;
0540             break
0541         else
0542             if algorithm ~= 1 && (grUB - grLB < GRtol)
0543                 %maximum growth rate found using an algorithm other than fzero
0544                 GRmax = grLB;
0545                 LP.Model.A =updateLPcom(modelCom, GRmax, GRfx, [], LP.Model.A, BMgdw);
0546                 feas = true;
0547                 try
0548                     LP.solve();
0549                 catch ME
0550                     if ErrBecauseInfeas(ME)
0551                         %treat as infeasible
0552                         feas = false;
0553                     else
0554                         disp(ME);
0555                         error('Unknown error from CPLEX.');
0556                     end
0557                 end
0558                 if feas && LP.Solution.status == 11
0559                     result.stat = 'time limit exceeded';
0560                     sol = [];
0561                     LP2 = [];
0562                     return
0563                 end
0564                 BMcur = 0;
0565                 dev = checkSolFeas(LP);
0566                 if dev <= feasTol
0567                     %update current biomass if successfully solved
0568                     BMcur = LP.Model.obj' * LP.Solution.x;
0569                 end
0570                 break
0571             end
0572             %Get the new guess for the growth rate using simple guess or bisection
0573             %Simple guess
0574             grNext = updateGRguess(BMcur, grCur);
0575             if grNext >= grUB * 0.99 || algorithm == 3 
0576                 %bisection if designated or the guess is too close to the
0577                 %upper bound
0578                 grCur = (grUB + grLB) / 2;
0579                  guessMethod = 1;
0580             elseif grNext <= max([grLB * 1.01, GRtol])
0581                 %if the guess is too close to the lower bound
0582                 if ~isinf(grUB)
0583                     %bisection if finite UB has been found
0584                     grCur = (grUB + grLB) / 2;
0585                 else
0586                     % 1% larger than LB if UB not found yet
0587                     grCur = grLB * 1.01;
0588                 end
0589                 guessMethod = 2;
0590             elseif abs(grNext - grCur) < 1e-2 * grCur
0591                 %When the step size is less than 1%, should be quite close to
0592                 %the solution but still not bounded from the
0593                 %other side. Use a 1% distance to get a bound
0594                 if grNext > grCur
0595                     grCur = grCur * 1.01;
0596                 else
0597                     grCur = grCur * 0.99;
0598                 end
0599                 guessMethod = 3;
0600             else
0601                 %new guess from simple guessing
0602                 grCur = grNext;
0603                 guessMethod = 0;
0604             end
0605         end
0606     end
0607 end
0608 
0609 %% final correction for a feasible solution in case of numerical instability
0610 % In this case fzero may return a GRmax with sum(X) = 0.
0611 % If it happens, take a slightly smaller growth rate
0612 kGRadjust = 0;
0613 while ~condition2(BMcur, GRmax) && GRmax > GRtol && kGRadjust <= 10
0614     kGRadjust = kGRadjust + 1;
0615     GRmax = GRmax - GRtol / 10;
0616     LP.Model.A =updateLPcom(modelCom, GRmax, GRfx, [], LP.Model.A, BMgdw);
0617     feas = true;
0618     try
0619         LP.solve();
0620     catch ME
0621         if ErrBecauseInfeas(ME)
0622             %treat as infeasible
0623             feas = false;
0624         else
0625             disp(ME);
0626             error('Unknown error from CPLEX.');
0627         end
0628     end
0629     if feas && LP.Solution.status == 11
0630         result.stat = 'time limit exceeded';
0631         sol = [];
0632         LP2 = [];
0633         return
0634     end
0635     % check the feasibility of the solution manually
0636     dev = checkSolFeas(LP);
0637     %biomass of the current iteration
0638     BMcur = 0;
0639     if feas && isfield(LP.Solution, 'objval') && dev <= feasTol
0640         BMcur = LP.Solution.objval;
0641     end
0642     if verbFlag
0643         fprintf('GRmax adjusment: %d\n',kGRadjust);
0644     end
0645 end
0646 %corrected solution not feasible
0647 numInstab2 = ~condition2(BMcur, GRmax) && GRmax > GRtol;
0648 %confirm the maximum growth rate
0649 result.GRmax = GRmax;
0650 if ~feas
0651     result.stat = 'infeasible';
0652     sol = [];
0653     LP2 = [];
0654     return
0655 end
0656 %take this as the solution as it contains useful information on dual values and
0657 %reduced cost (e.g. to find out limiting substrate)
0658 sol = LP.Solution;    
0659 
0660 %add maximum biomass as a constraint to ensure
0661 %that the model is feasible for further analysis (e.g. FVA)
0662 LP.addRows(BMcur * (1 - feasTol * 100),...
0663     sparse(ones(nSp,1), (n+1):(n+nSp), ones(nSp,1), 1, size(LP.Model.A,2)),...
0664     BMcur,'UnityBiomass');        
0665 LP.Model.obj(:) = 0;
0666 LP.Model.sense = 'minimize';
0667 feas = true;
0668 try
0669     LP.solve();
0670 catch ME
0671     if ErrBecauseInfeas(ME)
0672         %treat as infeasible
0673         feas = false;
0674     else
0675         disp(ME);
0676         error('Unknown error from CPLEX.');
0677     end
0678 end
0679 if feas && LP.Solution.status == 11
0680     result.stat = 'time limit exceeded';
0681     sol = [];
0682     LP2 = [];
0683     return
0684 end
0685 dev = checkSolFeas(LP);
0686 
0687 %the infeasibility may increase after adding the biomass constraint (Cplex issue),
0688 %adjust the minimum biomass slightly until feasible
0689 kBMadjust = 0;
0690 BMmaxLB = LP.Model.lhs(end);
0691 while (~isfield(LP.Solution, 'x') || dev > feasTol) && kBMadjust < 10
0692     kBMadjust = kBMadjust + 1;
0693     LP.Model.lhs(end) = BMmaxLB * (1 - feasTol/(11 - kBMadjust));
0694     LP.solve();
0695     if LP.Solution.status == 11
0696         result.stat = 'time limit exceeded';
0697         sol = [];
0698         LP2 = [];
0699         return
0700     end
0701     dev = checkSolFeas(LP);
0702     if verbFlag
0703         fprintf('BMmax adjusment: %d\n',kBMadjust);
0704     end
0705 end
0706 %solution after adding the biomass constraint becomes infeasible
0707 numInstab3 = ~isfield(LP.Solution, 'x') || dev > feasTol;
0708 
0709 LP2 = [];
0710 flux = LP.Solution.x;
0711 if numel(minNorm) == 1
0712     if minNorm == 1
0713         if verbFlag
0714             fprintf('Minimizing L1-norm...\n');
0715         end
0716         LP2 = Cplex('minSumFlux');
0717         LP2.Model = LP.Model;
0718         LP2 = setCplexParam(LP2,solverParam);
0719         LP2.Start = LP.Start;
0720         LP2.Model.obj(:) = 0;
0721         LP2.addCols(ones(n,1), sparse(size(LP2.Model.A,1),n), zeros(n,1), inf(n,1));
0722         n2 = size(LP2.Model.A,2);
0723         indLP.var.vAbs = (n2-n+1):n2;
0724         LP2.addRows(-inf(n,1), sparse([1:n, 1:n], [1:n, (n2-n+1):n2], ...
0725             [ones(n,1); -ones(n,1)], n, n2), zeros(n,1), char(strcat(modelCom.rxns,'_MinSumAbs1')));
0726         indLP.con.vAbs1 = (size(LP2.Model.A,1)-n+1):size(LP2.Model.A,1);
0727         LP2.addRows(-inf(n,1), sparse([1:n, 1:n], [1:n, (n2-n+1):n2], ...
0728             [-ones(n,1); -ones(n,1)], n, n2), zeros(n,1), char(strcat(modelCom.rxns,'_MinSumAbs2')));
0729         indLP.con.vAbs2 = (size(LP2.Model.A,1)-n+1):size(LP2.Model.A,1);
0730         LP2.solve();
0731         flux = LP2.Solution.x;
0732         sol = LP2.Solution;
0733     end
0734 end
0735 
0736 result.vBM = flux(modelCom.indCom.spBm);
0737 result.BM = flux(n + 1 : n + nSp);
0738 % result.BM(abs(result.BM) < 1e-8) = 0;
0739 result.Ut = flux(modelCom.indCom.EXcom(:,1));
0740 result.Ex = flux(modelCom.indCom.EXcom(:,2));
0741 result.flux = flux(1:n);
0742 result.iter = iter;
0743 if result.GRmax > GRtol
0744     if numInstab
0745         result.stat = 'Numerical instability (feasibility)';
0746     elseif numInstab2
0747         result.stat = 'Numerical instability (growth rate correction)';
0748     elseif numInstab3
0749         result.stat = 'Numerical instability (biomass constraint)';
0750     else
0751         %otherwise 'maintenance' set at the very beginning
0752         result.stat = 'optimal';
0753     end
0754 end
0755 if pL
0756     if numInstab
0757         fprintf('Numerical instability for feasibility during the iterations.\n');
0758     elseif numInstab2
0759         fprintf('Numerical instability after final correction of growth rate.\n');
0760     elseif numInstab3
0761         fprintf('Numerical instability after adding the biomass constraint.\n');
0762     end
0763     fprintf('Maximum community growth rate: %.6f (abs. error < %.1g).\tTime elapsed: %.0f sec\n', GRmax, GRtol, toc(t));
0764 end
0765 
0766 if ~isempty(saveModel)
0767     LP.writeModel([saveModel '.mps']);
0768     LP.writeBasis([saveModel '.bas']);
0769 end
0770 
0771 end
0772 
0773 function [LP,index] = constructLPcom(modelCom, options, solverParam)
0774 %the problem matrix is structured as follows:
0775 %variables (column):
0776 %[flux (species-specific rxn) | flux (community exchange) | biomass | absolute flux for MC]
0777 %constraint (row):
0778 % [mass balance;
0779 %  flux bouned above by ub * biomass;
0780 %  flux bouned below by lb * biomass;
0781 %  biomass reaction = growth rate * biomass;
0782 %  sum(mc_j * flux_j) <= biomass (molecular crowding constraint);
0783 %  constraint for uptake advantage]
0784 
0785 %% Initialization
0786 % get paramters
0787 if ~exist('options', 'var')
0788     options = struct();
0789 end
0790 if ~exist('solverParam', 'var')
0791     solverParam = struct();
0792 end
0793 param2get = {'BMcon', 'BMrhs','BMcsense', 'BMobj', 'BMgdw',...
0794              'GRfx', 'MCrhs',...
0795              'verbFlag', 'saveModel'};
0796 eval(sprintf('[%s] = getCobraComParams(param2get, options, modelCom);', ...
0797             strjoin(param2get, ',')...
0798             )...
0799     );
0800 
0801 [feasTol, optTol] = getCobraSolverParams('LP',{'feasTol'; 'optTol'}, solverParam);
0802 
0803 [m, n] = size(modelCom.S);
0804 nRxnSp = sum(modelCom.indCom.rxnSps > 0); %number of species-specific rxns
0805 nSp = numel(modelCom.indCom.spBm); %number of species
0806 
0807 if ~isempty(BMcon)
0808     if size(BMcon,2) ~= nSp || numel(unique([size(BMcon, 1) numel(BMrhs) length(BMcsense)])) ~= 1
0809         error('size of BMcon, BMrhs or BMcsense not correct.')
0810     end
0811 end
0812 
0813 if ~isempty(BMcon)
0814     if ismember(BMobj(:)', BMcon, 'rows')
0815         warning('BMobj should not be constrained. The algorithm may not converge.');
0816     end
0817 end
0818 
0819 %% construct LP
0820 %create CPLEX interactive object
0821 LP = Cplex('maxGrCom');
0822 nVar = 0;
0823 nCon = 0;
0824 %optimization sense
0825 LP.Model.sense = 'maximize';
0826 %objective vector
0827 obj = zeros(n + nSp, 1);
0828 %sum of biomass at default
0829 obj(n + 1: n + nSp) = BMobj;
0830 %constraint matrix
0831 A = updateLPcom(modelCom, 0, GRfx, BMcon, [], BMgdw);
0832 % species-specific fluxes bounded by biomass variable but not by constant
0833 lb = -inf(nRxnSp, 1);
0834 lb(modelCom.lb(1:nRxnSp)>=0) = 0;
0835 lb = [lb; modelCom.lb(nRxnSp + 1: n); zeros(nSp, 1)];
0836 % biomass upper bound should also be arbitrarily large, but set as 1000 here
0837 ub = inf(nRxnSp, 1);
0838 ub(modelCom.ub(1:nRxnSp)<=0) = 0;
0839 ub = [ub; modelCom.ub(nRxnSp + 1: n); 1000 * ones(nSp, 1)];
0840 %variable type, all continuous
0841 ctype = char('C' * ones(1, n + nSp));
0842 %variable names, X for biomass
0843 colname = [modelCom.rxns; strcat('X_', modelCom.infoCom.spAbbr(:))];
0844 index.var.v = nVar + 1: nVar + n;
0845 index.var.x = nVar + n + 1 : nVar + n + nSp;
0846 nVar = nVar + n + nSp;
0847 
0848 %handle constraint sense
0849 if ~isfield(modelCom, 'csense')
0850     cs = char(['E' * ones(1, m) 'L' * ones(1, 2 * nRxnSp) 'E' * ones(1, nSp) BMcsense(:)']);
0851 else 
0852     cs = [modelCom.csense(:)' char(['L' * ones(1, 2 * nRxnSp) 'E' * ones(1, nSp) BMcsense(:)'])];
0853 end
0854 %LHS, RHS for constraints
0855 [rhsAdd, lhsAdd] = deal(zeros(size(A, 1), 1));
0856 rhsAdd(cs == 'G') = inf;
0857 lhsAdd(cs == 'L') = -inf;
0858 rhs = [modelCom.b; zeros(2 * nRxnSp + nSp, 1); BMrhs] + rhsAdd;
0859 lhs = [modelCom.b; zeros(2 * nRxnSp + nSp, 1); BMrhs] + lhsAdd;
0860 %constraints' names
0861 rowname = [modelCom.mets; strcat(modelCom.rxns(modelCom.indCom.rxnSps > 0), '_ub');...
0862     strcat(modelCom.rxns(modelCom.indCom.rxnSps > 0), '_lb'); ...
0863     strcat('gr,mu,X_', modelCom.infoCom.spAbbr(:))];
0864 index.con.ub = nCon + 1 : nCon + m;
0865 nCon = nCon + m;
0866 index.con.ub = nCon + 1 : nCon + nRxnSp;
0867 nCon = nCon + nRxnSp;
0868 index.con.lb = nCon + 1 : nCon + nRxnSp;
0869 nCon = nCon + nRxnSp;
0870 index.con.gr = nCon + 1 : nCon + nSp;
0871 nCon = nCon + nSp;
0872 %names for biomass constraints if any
0873 if ~isempty(BMcon)
0874     rowname = [rowname;strcat('BMcon_',cellstr(num2str((1:size(BMcon,1))')))];
0875     index.con.bm = nCon + 1 : nCon + size(BMcon,1);
0876     nCon = nCon + size(BMcon,1);
0877 end
0878 
0879 %% More user-supplied constraints (optional)
0880 %options.MC: [n+nSp x K] matrix, for K additional constraints
0881 %options.MCmode: [n+nSp x K] matrix, with number 0 ~ 3
0882 %       0: original variable
0883 %       1: positive part of the variable
0884 %       2: negative part of the variable
0885 %       3: absolute value of the variable
0886 %options.MCrhs: right hand side of the constraints (optional, default all zeros)
0887 %options.MClhs: left hand side of the constraints (optional, default -inf)
0888 if isfield(options, 'MC') && ~isempty(options.MC)
0889     MCcont = true;
0890     %Check sizes
0891     if isfield(options,'MCmode')
0892         %MC and MCmode must have the same size of n+nSp x no. of constraints
0893         if ~isequal(size(options.MC),size(options.MCmode))
0894             if ~isequal(size(options.MC),size(options.MCmode'))
0895                 warning('Size of MCmode does not match that of MC. Ignore.')
0896                 MCcont = false;
0897             else
0898                 MCmode = options.MCmode';
0899             end
0900         else
0901             MCmode = options.MCmode;
0902         end
0903     else
0904         MCmode = sparse(size(options.MC,1),size(options.MC,2));
0905     end
0906     if size(options.MC, 1) ~= n + nSp
0907         if size(options.MC,2) == n + nSp
0908             options.MC = options.MC';
0909         else
0910             warning('Size of the crowding constraint matrix not correct. Ignore.')
0911             MCcont = false;
0912         end
0913     end
0914     %RHS for MC constraint.
0915     if isfield(options,'MCrhs')
0916         MCrhs = options.MCrhs(:);
0917     else
0918         MCrhs = zeros(size(options.MC,2),1);
0919     end
0920     if isfield(options,'MClhs')
0921         MClhs = options.MClhs(:);
0922     else
0923         MClhs = -inf(size(options.MC,2),1);
0924     end
0925     if numel(MCrhs) == 1
0926         MCrhs = MCrhs * ones(size(options.MC,2),1);
0927     elseif numel(MCrhs) ~= size(options.MC,2)
0928         warning('size of MCrhs not equal to size(options.MC,2). Ignore.')
0929         MCcont = false;
0930     end
0931     if numel(MClhs) == 1
0932         MClhs = MClhs * ones(size(options.MC,2),1);
0933     elseif numel(MClhs) ~= size(options.MC,2)
0934         warning('size of MClhs not equal to size(options.MC,2). Ignore.')
0935         MCcont = false;
0936     end
0937     
0938     if MCcont
0939         if verbFlag
0940             fprintf('User-supplied constraints imposed.\n');
0941         end
0942         %list of fluxes requiring decomposition variables (non-zero MCmode and
0943         %non-zero MC)
0944         %first filter by lb and ub to reduce variables to be added
0945         for j = 1:size(MCmode,2)
0946             %Ignore variables with non-negative lb but designated to use
0947             %negative part. Must be zero
0948             options.MC(lb >= 0 & MCmode(:,j) == 2,j) = 0;
0949             %variables with non-negative lb and designated to use positive part or
0950             %absolute value, simply using the original variable
0951             MCmode(lb >= 0 & (MCmode(:,j) == 3 | MCmode(:,j) == 1),j) = 0;
0952             %Ignore variables with non-positive ub but designated to use
0953             %positive part. Must be zero
0954             options.MC(ub <= 0 & MCmode(:,j) == 1,j) = 0;
0955             %variables with non-positive ub and designated to use negative part or
0956             %absolute value, simply using the negative of the original variable
0957             options.MC(ub <= 0 & (MCmode(:,j) == 3 | MCmode(:,j) == 2),j) ...
0958                 = - options.MC(ub <= 0 & (MCmode(:,j) == 3 | MCmode(:,j) == 2),j);
0959             MCmode(ub <= 0 & (MCmode(:,j) == 3 | MCmode(:,j) == 2),j) = 0;
0960         end
0961         Vdecomp = find(any(options.MC ~=0 & MCmode ~= 0,2));
0962         nMCrow = numel(Vdecomp);
0963         %record the index for each new variable and flux
0964         VdecompInd = [(1:n+nSp)', sparse(repmat(Vdecomp(:),2,1),reshape(repmat(1:2,nMCrow,1),2*nMCrow,1),...
0965             n+nSp+1:n+nSp+nMCrow*2,n+nSp,2)];
0966         %new columns for decomposition variables
0967         obj = [obj; zeros(nMCrow*2,1)];
0968         lb = [lb;zeros(nMCrow*2,1)];
0969         ub = [ub;inf(nMCrow*2,1)];
0970         colname = [colname; strcat(modelCom.rxns(Vdecomp),'_pos');strcat(modelCom.rxns(Vdecomp),'_neg')];
0971         index.var.vp = nVar + 1 : nVar + nMCrow;
0972         nVar = nVar + nMCrow;
0973         index.var.vn = nVar + 1 : nVar + nMCrow;
0974         nVar = nVar + nMCrow;
0975         ctype = [ctype char('C' * ones(1, nMCrow*2))];
0976         %new rows to add ( 0<= V - V_pos + V_neg <= 0)
0977         lhs = [lhs; zeros(nMCrow,1)];
0978         rhs = [rhs; zeros(nMCrow,1)];
0979         %matrix to update
0980         row = [1:nMCrow, 1:nMCrow, 1:nMCrow];
0981         col = [Vdecomp(:)', n+nSp+1:n+nSp+nMCrow*2];
0982         entry = [ones(1,nMCrow), -ones(1,nMCrow), ones(1,nMCrow)];
0983         A = [A sparse(size(A, 1), nMCrow*2);...
0984             sparse(row, col, entry, nMCrow, n + nSp + nMCrow*2)];
0985         rowname = [rowname; strcat(modelCom.rxns(Vdecomp),'_decomp')];
0986         index.con.decomp = nCon + 1 : nCon + nMCrow;
0987         nCon = nCon + nMCrow;
0988         %add MC constraints
0989         MCmodeLogic = repmat(struct('mode',[]),3,1);
0990         %MCmode: 0, original flux; 1, +ve flux; 2, -ve flux; 3, absolute flux
0991         %original variable
0992         MCmodeLogic(1).mode = MCmode == 0 & options.MC ~= 0;
0993         %positive part
0994         MCmodeLogic(2).mode = (MCmode == 1 | MCmode == 3) & options.MC ~= 0;
0995         %negative part
0996         MCmodeLogic(3).mode = (MCmode == 2 | MCmode == 3) & options.MC ~= 0;
0997         nMCcon = 0;
0998         for j = 1:3
0999             nMCcon = nMCcon + nnz(MCmodeLogic(j).mode);
1000         end
1001         %each original, positive or negative flux 1 entry, each absolute
1002         %flux 2 entires
1003         [row, col, entry] = deal(zeros(nMCcon , 1));
1004         ct = 0;
1005         ct1 = 0;
1006         for j = 1:size(options.MC,2)
1007             for k = 1:3
1008                 %add the corresponding variables into the constraint:
1009                 %original, positive part and negative part
1010                 nJ = MCmodeLogic(k).mode(:,j);
1011                 col(ct1+1:ct1+sum(nJ)) = VdecompInd(nJ,k);
1012                 entry(ct1+1:ct1+sum(nJ)) = options.MC(nJ,j);
1013                 ct1 = ct1 + sum(nJ);
1014             end
1015             row(ct+1:ct1) = j;
1016             ct = ct1;
1017         end
1018         lhs = [lhs; MClhs];
1019         rhs = [rhs; MCrhs];
1020         A = [A; sparse(row, col, entry, size(options.MC,2), n + nSp + nMCrow*2)];
1021         rowname = [rowname; strcat('more_con_', ...
1022             strtrim(cellstr(num2str((1:size(options.MC,2))'))))]; 
1023         index.con.mc = nCon + 1 : nCon + size(options.MC,2);
1024         nCon = nCon + size(options.MC,2);
1025     end
1026 end
1027 
1028 LP.addRows(lhs, [], rhs, char(rowname));
1029 LP.addCols(obj,A,lb,ub,[],char(colname));
1030 
1031 %% Set Cplex parameters
1032 % set feasibility and optimality to follow the default setting in COBRA
1033 LP.Param.simplex.tolerances.feasibility.Cur = feasTol;
1034 LP.Param.simplex.tolerances.optimality.Cur = optTol;
1035 % set parameters given in solverParam. Will override the above two setting
1036 % if given in solverParam.
1037 [paramList, paramPath] = getParamList(LP.Param, 0);
1038 [paramUserList, paramUserPath] = getParamList(solverParam, 1);
1039 paramIden = false(numel(paramUserList), 1);
1040 for p = 1:numel(paramUserList)
1041     f = strcmpi(paramList,paramUserList{p});
1042     if sum(f) == 1
1043         paramIden(p) = true;
1044         str = ['LP.Param.' paramPath{f} '.Cur = solverParam.' paramUserPath{p} ';'];
1045         eval(str);
1046     elseif sum(f) > 1
1047         if ismember(lower(paramUserPath{p}), paramPath);
1048             paramIden(p) = true;
1049             str = ['LP.Param.' lower(paramUserPath{p}) '.Cur = solverParam.' paramUserPath{p} ';'];
1050             eval(str);
1051         else
1052             if verbFlag
1053                 fprintf('solverParam.%s cannot be uniquely identified as a valid cplex parameter. Ignore.\n', paramUserPath{p});
1054             end
1055         end
1056     else
1057         if verbFlag
1058             fprintf('solverParam.%s cannot be identified as a valid cplex parameter. Ignore.\n', paramUserPath{p});
1059         end
1060     end
1061 end
1062 
1063 if ~isempty(saveModel)
1064     LP.writeParam([saveModel '.prm']);
1065 end
1066 
1067 end
1068 
1069 function LPproblem = updateLPcom(modelCom, grCur, GRfx, BMcon, LPproblem, BMgdw)
1070 %LPproblem = updateLPcom(modelCom, grCur, GRfx, BMcon, LPproblem, BMgdw)
1071 % create the LP problem [LP(grCur)] given growth rate grCur and other
1072 % constraints if LPproblem as input does not contain the field 'A',
1073 % or is empty or is not inputted.
1074 % Otherwise, update LPproblem with the growth rate grCur. Only the
1075 % arguements 'modelCom', 'grCur', 'GRfx' and 'LPproblem' are used in this
1076 % case.
1077 %
1078 % Input:
1079 %   modelCom:   community model
1080 %   grCur:      the current growth rate for the LP to be updated to
1081 %   GRfx:       fixed growth rate of a certain species
1082 %   BMcon:      constraint matrix for species biomass
1083 %   LPproblem:  LP problem structure with field 'A' or the problem matrix
1084 %               directly
1085 %   BMgdw:      the gram dry weight per mmol of the biomass reaction of
1086 %               each species (nSp x 1 vector, default all 1)
1087 %
1088 % return a structure with the field 'A' updated if the input 'LPproblem' is
1089 % a structure or return a matrix if 'LPproblem' is the problem matrix
1090 m = size(modelCom.S, 1);
1091 n = size(modelCom.S, 2);
1092 nRxnSp = sum(modelCom.indCom.rxnSps > 0);
1093 nSp = numel(modelCom.infoCom.spAbbr);
1094 if ~exist('grCur', 'var')
1095     grCur = 0;
1096 elseif isempty(grCur)
1097     grCur = 0;
1098 end
1099 if ~exist('GRfx', 'var')|| isempty(GRfx)
1100     GRfx  = getCobraComParams({'GRfx'}, struct(), modelCom);
1101 end
1102 if ~exist('LPproblem', 'var')
1103     LPproblem = struct();
1104 end
1105 
1106 construct = false;
1107 if ~isstruct(LPproblem)
1108     if isempty(LPproblem)
1109         construct = true;
1110     end
1111 elseif ~isfield(LPproblem, 'A')
1112     construct = true;
1113 end
1114 if construct
1115     if ~exist('BMgdw', 'var')
1116         BMgdw = ones(nSp,1);
1117     end
1118     %upper bound matrix
1119     S_ub = sparse([1:nRxnSp 1:nRxnSp]', [(1:nRxnSp)'; n + modelCom.indCom.rxnSps(1:nRxnSp)],...
1120           [ones(nRxnSp,1); -modelCom.ub(1:nRxnSp)], nRxnSp, n + nSp);
1121     %lower bound matrix
1122     S_lb = sparse([1:nRxnSp 1:nRxnSp]', [(1:nRxnSp)'; n + modelCom.indCom.rxnSps(1:nRxnSp)],...
1123           [-ones(nRxnSp,1); modelCom.lb(1:nRxnSp)], nRxnSp, n + nSp);
1124     %growth rate and biomass link matrix
1125     grSp = zeros(nSp, 1);
1126     grSp(isnan(GRfx)) = grCur;
1127     %given fixed growth rate
1128     grSp(~isnan(GRfx)) = GRfx(~isnan(GRfx));
1129     S_gr = sparse([1:nSp 1:nSp]', [modelCom.indCom.spBm(:) (n + 1:n + nSp)'],...
1130                   [BMgdw; -grSp], nSp, n + nSp);
1131     if isempty(BMcon)
1132         A = [modelCom.S sparse([],[],[], m, nSp); S_ub; S_lb; S_gr];
1133     else
1134         A = [modelCom.S sparse([],[],[], m, nSp); S_ub; S_lb; S_gr;...
1135                    sparse([],[],[],size(BMcon, 1), n) BMcon];
1136     end
1137     if isstruct(LPproblem)
1138         LPproblem.A = A;
1139     else
1140         LPproblem = A;
1141     end
1142 else
1143     for j = 1:nSp
1144         if isstruct(LPproblem)
1145             if isnan(GRfx(j))
1146                 LPproblem.A(m + 2*nRxnSp + j, n + j) = -grCur;
1147             else
1148                 LPproblem.A(m + 2*nRxnSp + j, n + j) = -GRfx(j);
1149             end
1150         else
1151             if isnan(GRfx(j))
1152                 LPproblem(m + 2*nRxnSp + j, n + j) = -grCur;
1153             else
1154                 LPproblem(m + 2*nRxnSp + j, n + j) = -GRfx(j);
1155             end
1156         end
1157     end
1158 end
1159 end
1160 
1161 function [paramList, paramPath] = getParamList(param, bottomFlag)
1162 %for matching CPLEX parameters appropriately
1163 structCur = param;
1164 lv = 1;
1165 lvFieldN = zeros(10,1);
1166 lvFieldN(1) = 1;
1167 lvField = cell(10, 1);
1168 lvField{lv} = fieldnames(structCur);
1169 paramPath = {};
1170 paramList = {};
1171 while lv > 0
1172     if isstruct(structCur.(lvField{lv}{lvFieldN(lv)}))
1173         structCur = structCur.(lvField{lv}{lvFieldN(lv)});
1174         lv = lv + 1;
1175         lvFieldN(lv) = 1;
1176         lvField{lv} = fieldnames(structCur);
1177     else
1178         if ~bottomFlag
1179             lv = lv - 1;
1180         end
1181         if lv > 0
1182             c = {};
1183             for j = 1:lv
1184                 c = [c lvField{j}(lvFieldN(j))];
1185             end
1186             paramPath = [paramPath; strjoin(c,'.')];
1187             paramList = [paramList; c(end)];
1188             while lvFieldN(lv) == numel(lvField{lv})
1189                 lv = lv - 1;
1190                 if lv == 0
1191                     break
1192                 end
1193             end
1194             if lv > 0
1195                 lvFieldN(lv) = lvFieldN(lv) + 1;
1196                 structCur = param;
1197                 for j = 1:lv-1
1198                     structCur = structCur.(lvField{j}{lvFieldN(j)});
1199                 end
1200             end
1201         else
1202             lv = 1;
1203             if lvFieldN(lv) == numel(lvField{lv})
1204                 break
1205             else
1206                 lvFieldN(1) = lvFieldN(1) + 1;
1207             end
1208         end
1209     end
1210 end
1211 
1212 end
1213 
1214 function dBM = LP4fzero1(grCur, LP, modelCom, GRfx, feasTol, BMequiv,BMgdw)
1215     LP.Model.A =updateLPcom(modelCom, grCur, GRfx, [], LP.Model.A, BMgdw);
1216     LP.solve();
1217     if LP.Solution.status == 11
1218         dBM = [];
1219         return
1220     end
1221     % check the feasibility of the solution manually
1222     dev = checkSolFeas(LP);
1223     %biomass of the current iteration
1224     BMcur = 0;
1225     if isfield(LP.Solution, 'x') && dev <= feasTol
1226         if ~any(isnan(LP.Solution.x))
1227             BMcur = LP.Model.obj' * LP.Solution.x;
1228         end
1229     end
1230     dBM = BMequiv - BMcur;
1231 end
1232 
1233 function dBM = LP4fzero2(grCur, LP, modelCom, GRfx, feasTol, BMequiv, GR0, BMgdw)
1234     LP.Model.A =updateLPcom(modelCom, grCur, GRfx, [], LP.Model.A, BMgdw);
1235     LP.solve();
1236     if LP.Solution.status == 11
1237         dBM = [];
1238         return
1239     end
1240     % check the feasibility of the solution manually
1241     dev = checkSolFeas(LP);
1242     %biomass of the current iteration
1243     BMcur = 0;
1244     if isfield(LP.Solution, 'x') && dev <= feasTol
1245         if ~any(isnan(LP.Solution.x))
1246             BMcur = LP.Model.obj' * LP.Solution.x;
1247         end
1248     end
1249     dBM = (BMequiv * GR0 / grCur) - BMcur;
1250 end
1251 
1252 function yn = ErrBecauseInfeas(ME)
1253 yn = ~isempty(strfind(lower(ME.message),'cplex')) && ...
1254     ~isempty(strfind(lower(ME.message),'error')) && ...
1255     ~isempty(strfind(lower(ME.message),'1256'));
1256 end
```

---

Generated on Sat 06-May-2017 09:55:30 by **m2html** © 2005
