# Supplementary material for: SteadyCom: Predicting microbial abundances while ensuring community stability
Source: PLoS Comput Biol. 2017 May 15;13(5):e1005539. doi: 10.1371/journal.pcbi.1005539 (PMC5448816; doi:10.1371/journal.pcbi.1005539)
Supplement: S1 Dataset — (ZIP) [file pcbi.1005539.s018.zip › S1 Dataset/SteadyCom/doc/SteadyCom/SteadyComFVACplex.html]

Description of SteadyComFVACplex


# SteadyComFVACplex

## PURPOSE

**Flux variability analysis for community model at community steady-state for a range of growth rates.**

## SYNOPSIS

**function [minFlux,maxFlux,minFD,maxFD, GRvector, result,LP] = SteadyComFVACplex(modelCom,options,solverParam)**

## DESCRIPTION

```
Flux variability analysis for community model at community steady-state for a range of growth rates. 
The function is capable of saving intermediate results and continuing from previous results 
if the file path is given in options.saveFVA. It also allows switch from single thread to parallel 
computation from intermediate results (but not the reverse).

[minFlux,maxFlux,Vmin,Vmax] = SteadyComFVACplex(modelCom,options,solverParam)

INPUT
 modelCom       A community COBRA model structure with the following extra fields:
 (the following fields are required - others can be supplied)
   S            Stoichiometric matrix
   b            Right hand side
   c            Objective coefficients
   lb           Lower bounds
   ub           Upper bounds
 (at least one of the below two is needed)
   infoCom      structure containing community reaction info 
                (returned along with the community model created with createCommModel)
   indCom       the index structure corresponding to infoCom

 options (optional) structure with the following fields:
   optGRpercent    A vector of percentages. Perform FVA at these percents
                     of max. growth rate respectively (Default = 99.99)
   optBMpercent    Only consider solutions that yield at least a certain
                     percentage of the optimal biomass (Default = 99.99)
   rxnNameList     List of reactions (IDs or .rxns) for which FVA is performed.
                     Use a (N_rxns + N_organism) x K matrix for FVA of K
                     linear combinations of fluxes and/or abundances
                     (Default = biomass reaction of each species)
   rxnFluxList     List of reactions (IDs or .rxns) whose fluxes are also returned
                     (Default = biomass reaction of each species)
   GRmax           maximum growth rate of the model (default to be found
                     SteadyComCplex.m)
  (the two parameters below are usually determined by solving the problem
      during the program. Provide them only if you want to constrain the
      total biomass to a particular value)
   BMmaxLB         lower bound for the total biomass (default 1)
   BMmaxUB         upper bound for the total biomass
   (other parameters)
   saveFVA         If non-empty, become the filename to save the FVA results
                   (default empty, not saving)
   threads         > 1 for explicitly stating the no. of threads used,
                   0 or -1 for using all available threads. Default 1.
   verbFlag        Verbose output. 1 to have waitbar, >1 to have stepwise output
                   (default 3)
   loadModel       String of filename to be loaded. If non-empty, load the 
                   cplex model ('loadModel.mps'), basis ('loadModel.bas') 
                   and parameters ('loadModel.prm').
  May add also other parameters in SteadyComCplex for calculating the maximum growth rate.

 solverParam       Cplex parameter structure. E.g., struct('simplex',struct('tolerances',struct('feasibility',1e-8)))

OUTPUT
 minFlux       Minimum flux for each reaction
 maxFlux       Maximum flux for each reaction

 OPTIONAL OUTPUT
 minFD         #rxnFluxList x #rxnNameList matrix containing the fluxes in
               options.rxnFluxList corresponding to minimizing each reaction in
               options.rxnNameList
 maxFD         #rxnFluxList x #rxnNameList matrix containing the fluxes in
               options.rxnFluxList corresponding to maximizing each reaction in
               options.rxnNameList
 GRvector      A vector of growth rates at which FVA has been performed
 result        result structure from SteadyComCplex
 LP            Cplex LP object
```

## CROSS-REFERENCE INFORMATION

This function calls:

- SteadyComCplex Find the maximum community growth rate at community steady-state using SteadyCom
- SteadyComFVAgrCplex Flux variability analysis for community model at community steady-state at a given growth rate.
- checkSolFeas Check the feasibility of a solution given a COBRA model structure or a CPLEX dynamic object and a solution
- getCobraComParams get the required default parameters
- infoCom2indCom Transform between community reaction IDs and reaction names
- setCplexParam Set the parameters of the CPLEX object according to the structure solverParam
- updateLPcom Create and update the SteadyCom LP model in CPLEX format.

This function is called by:


## SOURCE CODE

```
0001 function [minFlux,maxFlux,minFD,maxFD, GRvector, result,LP] = SteadyComFVACplex(modelCom,options,solverParam)
0002 %Flux variability analysis for community model at community steady-state for a range of growth rates.
0003 %The function is capable of saving intermediate results and continuing from previous results
0004 %if the file path is given in options.saveFVA. It also allows switch from single thread to parallel
0005 %computation from intermediate results (but not the reverse).
0006 %
0007 %[minFlux,maxFlux,Vmin,Vmax] = SteadyComFVACplex(modelCom,options,solverParam)
0008 %
0009 %INPUT
0010 % modelCom       A community COBRA model structure with the following extra fields:
0011 % (the following fields are required - others can be supplied)
0012 %   S            Stoichiometric matrix
0013 %   b            Right hand side
0014 %   c            Objective coefficients
0015 %   lb           Lower bounds
0016 %   ub           Upper bounds
0017 % (at least one of the below two is needed)
0018 %   infoCom      structure containing community reaction info
0019 %                (returned along with the community model created with createCommModel)
0020 %   indCom       the index structure corresponding to infoCom
0021 %
0022 % options (optional) structure with the following fields:
0023 %   optGRpercent    A vector of percentages. Perform FVA at these percents
0024 %                     of max. growth rate respectively (Default = 99.99)
0025 %   optBMpercent    Only consider solutions that yield at least a certain
0026 %                     percentage of the optimal biomass (Default = 99.99)
0027 %   rxnNameList     List of reactions (IDs or .rxns) for which FVA is performed.
0028 %                     Use a (N_rxns + N_organism) x K matrix for FVA of K
0029 %                     linear combinations of fluxes and/or abundances
0030 %                     (Default = biomass reaction of each species)
0031 %   rxnFluxList     List of reactions (IDs or .rxns) whose fluxes are also returned
0032 %                     (Default = biomass reaction of each species)
0033 %   GRmax           maximum growth rate of the model (default to be found
0034 %                     SteadyComCplex.m)
0035 %  (the two parameters below are usually determined by solving the problem
0036 %      during the program. Provide them only if you want to constrain the
0037 %      total biomass to a particular value)
0038 %   BMmaxLB         lower bound for the total biomass (default 1)
0039 %   BMmaxUB         upper bound for the total biomass
0040 %   (other parameters)
0041 %   saveFVA         If non-empty, become the filename to save the FVA results
0042 %                   (default empty, not saving)
0043 %   threads         > 1 for explicitly stating the no. of threads used,
0044 %                   0 or -1 for using all available threads. Default 1.
0045 %   verbFlag        Verbose output. 1 to have waitbar, >1 to have stepwise output
0046 %                   (default 3)
0047 %   loadModel       String of filename to be loaded. If non-empty, load the
0048 %                   cplex model ('loadModel.mps'), basis ('loadModel.bas')
0049 %                   and parameters ('loadModel.prm').
0050 %  May add also other parameters in SteadyComCplex for calculating the maximum growth rate.
0051 %
0052 % solverParam       Cplex parameter structure. E.g., struct('simplex',struct('tolerances',struct('feasibility',1e-8)))
0053 %
0054 %OUTPUT
0055 % minFlux       Minimum flux for each reaction
0056 % maxFlux       Maximum flux for each reaction
0057 %
0058 % OPTIONAL OUTPUT
0059 % minFD         #rxnFluxList x #rxnNameList matrix containing the fluxes in
0060 %               options.rxnFluxList corresponding to minimizing each reaction in
0061 %               options.rxnNameList
0062 % maxFD         #rxnFluxList x #rxnNameList matrix containing the fluxes in
0063 %               options.rxnFluxList corresponding to maximizing each reaction in
0064 %               options.rxnNameList
0065 % GRvector      A vector of growth rates at which FVA has been performed
0066 % result        result structure from SteadyComCplex
0067 % LP            Cplex LP object
0068 %
0069 
0070 %% Initialization
0071 %check required fields for community model
0072 if ~isfield(modelCom,'indCom')
0073     if ~isfield(modelCom,'infoCom') || ~isstruct(modelCom.infoCom) || ...
0074             ~all(isfield(modelCom.infoCom,{'spBm','EXcom','EXsp','spAbbr','rxnSps','metSps'}))
0075         error('infoCom must be provided for calculating the max. community growth rate.\n');
0076     end
0077     %get useful reaction indices
0078     modelCom.indCom = infoCom2indCom(modelCom);
0079 end
0080 
0081 %get paramters
0082 if ~exist('options', 'var')
0083     options = struct();
0084 end
0085 if ~exist('solverParam', 'var') || isempty(solverParam)
0086     %default Cplex parameters
0087     solverParam = getCobraComParams('CplexParam');
0088 end
0089 param2get = {'GRmax', 'optGRpercent', 'rxnNameList', 'rxnFluxList',...
0090              'GRfx','BMmaxLB','BMmaxUB', ...
0091              'verbFlag', 'loadModel','saveFVA','threads'};
0092 eval(sprintf('[%s] = getCobraComParams(param2get, options, modelCom);', ...
0093             strjoin(param2get, ',')...
0094             )...
0095     );
0096 
0097 [feasTol, ~] = getCobraSolverParams('LP',{'feasTol'; 'optTol'}, solverParam);
0098 if isfield(solverParam,'simplex') && isfield(solverParam.simplex, 'tolerances')...
0099         && isfield(solverParam.simplex.tolerances,'feasibility')
0100     %override the feasTol in CobraSolverParam if given in solverParam
0101     feasTol = solverParam.simplex.tolerances.feasibility;
0102 else
0103     %otherwise use the feasTol in COBRA toolbox
0104     solverParam.simplex.tolerances.feasibility = feasTol;
0105 end
0106 
0107 [m, n] = size(modelCom.S);
0108 nSp = numel(modelCom.indCom.spBm); %number of species
0109 nRxnSp = sum(modelCom.indCom.rxnSps > 0); %number of species-specific rxns
0110 
0111 if ischar(rxnNameList)
0112     rxnNameList = {rxnNameList};
0113 end
0114 if iscell(rxnNameList)
0115     nRxnFVA = numel(rxnNameList);
0116 else
0117     nRxnFVA = size(rxnNameList,2);
0118 end
0119 if ischar(rxnFluxList)
0120     rxnFluxList = {rxnFluxList};
0121 end
0122 %get maximum growth rate
0123 addRow = false;
0124 GRgiven = false;
0125 if isempty(GRmax)
0126     if exist('Cplex.p','file') == 6
0127         [~, result,LP] = SteadyComCplex(modelCom, options, solverParam);
0128         
0129     else
0130         warning('Support Cplex only right now.');
0131         return
0132         %need further achitecture for using COBRA solver
0133     end
0134     if strcmp(result.stat,'infeasible')
0135         %infeasible model
0136         warning('Model is infeasible.');
0137         [minFlux,maxFlux] = deal(NaN(nRxnFVA,1));
0138         [minFD,maxFD] = deal(NaN(numel(rxnFluxList), nRxnFVA));
0139         GRvector = NaN(numel(optGRpercent), 1);
0140         return
0141     end
0142     GRmax = result.GRmax;
0143     idRow = size(LP.Model.A,1); %row that constrains total biomass
0144 else
0145     %If GRmax is given, BMmaxLB and BMmaxUB should be included in options in this case to ensure feasibility
0146     if ~isempty(loadModel)
0147         % load solution if given and growth rate is known
0148         LP = Cplex('fluxSampling');
0149         LP.readModel([loadSol '.mps']);
0150         LP.readBasis([loadSol '.bas']);
0151         LP.readParam([loadSol '.prm']);
0152         fprintf('Load model ''%s'' successfully.\n', loadModel);
0153         addRow = true;
0154         if size(LP.Model.A,1) > m + 2*nRxnSp + nSp
0155             %try to find the row that constrains total biomass
0156             [ynRow,idRow] = ismember(sparse(ones(nSp,1),n+1:n+nSp,ones(nSp,1),1,n+nSp),...
0157                 LP.Model.A(m+2*nRxnSp+nSp+1:end,1:n+nSp),'rows');
0158             if ynRow
0159                 idRow = m + 2*nRxnSp + nSp + idRow;
0160             end
0161             addRow = ~ynRow;
0162         end
0163     else
0164         %get LP using SteadyComCplex if only growth rate is given
0165         options2 = options;
0166         options2.LPonly = true;
0167         [~, ~, LP] = SteadyComCplex(modelCom, options2, solverParam);
0168         %no constraint on total biomass using LPonly option
0169         addRow = true;
0170     end
0171     result = struct('GRmax',GRmax,'vBM',[],'BM',[],'Ut',[],'Ex',[],'flux',[],'iter0',[],'iter',[],'stat','optimal');
0172     GRgiven = true;
0173 end
0174 if addRow
0175     %add a row for constraining the sum of biomass if not exist
0176     %using default BMmaxLB and BMmaxUB if not given in options
0177     LP.addRows(BMmaxLB, ...
0178         sparse(ones(1, nSp), n + 1: n + nSp, ones(1, nSp), 1, size(LP.Model.A,2)),...
0179         BMmaxUB, 'UnityBiomass');
0180     idRow = size(LP.Model.A,1);
0181 else
0182     %using BMmaxLB and BMmaxUB stored in the LP if not given in options
0183     if ~isfield(options,'BMmaxLB') %take from LP if not supplied
0184         BMmaxLB = LP.Model.lhs(idRow);
0185     end
0186     if ~isfield(options,'BMmaxUB') %take from LP if not supplied
0187         BMmaxUB = LP.Model.rhs(idRow);
0188     end
0189     LP.Model.lhs(idRow) = BMmaxLB;
0190     %not allow the max. biomass to exceed the one at max growth rate,
0191     %can happen if optBMpercent < 100. May dismiss this constraint or
0192     %manually supply BMmaxUB in the options if sum of biomass should be variable
0193     LP.Model.rhs(idRow) = BMmaxUB;
0194 end
0195 %set Cplex parameters
0196 LP = setCplexParam(LP, solverParam);
0197 %update the LP to ensure the current growth rate is constrained
0198 LP.Model.A = updateLPcom(modelCom, GRmax, GRfx, [], LP.Model.A, []);
0199 LP.Model.sense = 'minimize';
0200 LP.Model.obj(:) = 0;
0201 LP.solve();
0202 %check and adjust for feasibility
0203 %(LP from SteadyComCplex should pass this automatically as the row has
0204 % been added in SteadyComCplex)
0205 dev = checkSolFeas(LP);
0206 kBMadjust = 0;
0207 while (~isfield(LP.Solution, 'x') || dev > feasTol) && kBMadjust < 10
0208     kBMadjust = kBMadjust + 1;
0209     %the row of biomass constraint should at the end
0210     LP.Model.lhs(idRow) = BMmaxLB * (1 - feasTol/(11 - kBMadjust));
0211     LP.solve();
0212     dev = checkSolFeas(LP);
0213     if verbFlag
0214         fprintf('BMmax adjusment: %d\n',kBMadjust);
0215     end
0216 end
0217 if (~isfield(LP.Solution, 'x') || dev > feasTol)
0218     warning('Model not feasible.')
0219     [minFlux,maxFlux] = deal(NaN(nRxnFVA,1));
0220     [minFD,maxFD] = deal(NaN(numel(rxnFluxList), nRxnFVA));
0221     GRvector = NaN(numel(optGRpercent), 1);
0222     result.stat = 'infeasible';
0223     return
0224 end
0225 if GRgiven
0226     %assign result structure if in the rare case of given maximum growth
0227     %rate
0228     result.vBM = LP.Solution.x(modelCom.indCom.spBm);
0229     result.BM = LP.Solution.x(n+1:n+nSp);
0230     result.Ut = LP.Solution.x(modelCom.indCom.EXcom(:,1));
0231     result.Ex = LP.Solution.x(modelCom.indCom.EXcom(:,2));
0232     result.flux = LP.Solution.x(1:n);
0233 end
0234 if ~isfield(options, 'BMmaxLB')
0235     options.BMmaxLB = LP.Model.lhs(idRow);
0236 end
0237 if ~isfield(options, 'BMmaxUB')
0238     options.BMmaxUB = LP.Model.rhs(idRow);
0239 end
0240 
0241 GRvector = GRmax * optGRpercent/100;
0242 if ~isempty(saveFVA)
0243     %decide number of digits in the save name
0244     if numel(optGRpercent) == 1
0245         kDisp = 2;
0246     else
0247         d = min(GRvector(2:end) - GRvector(1:end-1));
0248         if d < 1
0249             kDisp = abs(floor(log10(d)));
0250         else
0251             kDisp = 0;
0252         end
0253     end
0254 end
0255 
0256 [minFlux, maxFlux] = deal(zeros(nRxnFVA, numel(GRvector)));
0257 [minFD, maxFD] = deal(zeros(numel(rxnFluxList), nRxnFVA, numel(GRvector)));
0258 
0259 %parallel computation
0260 p = gcp('nocreate');
0261 if isempty(p)
0262     if threads > 1
0263         %given explicit no. of threads
0264         parpool(ceil(threads));
0265     elseif threads ~= 1
0266         %default max no. of threads (input 0 or -1 etc)
0267         parpool;
0268     end
0269 end
0270 %perform FVA at each growth rate
0271 for j = 1:numel(GRvector)
0272     optionsJ = options;
0273     optionsJ.GR = GRvector(j);
0274     if ~isempty(saveFVA)
0275         optionsJ.saveFVA = sprintf(['%s_GR%.' num2str(kDisp) 'f'], saveFVA, GRvector(j));  
0276     end
0277     [minFluxJ,maxFluxJ,minFDj,maxFDj,LP] = SteadyComFVAgrCplex(modelCom,optionsJ, solverParam,LP);
0278     minFlux(:, j) = minFluxJ;
0279     maxFlux(:, j) = maxFluxJ;
0280     minFD(:,:, j) = minFDj;
0281     maxFD(:,:, j) = maxFDj;
0282 end
0283 
0284 end
```

---

Generated on Sat 06-May-2017 09:55:30 by **m2html** © 2005
