# Supplementary material for: SteadyCom: Predicting microbial abundances while ensuring community stability
Source: PLoS Comput Biol. 2017 May 15;13(5):e1005539. doi: 10.1371/journal.pcbi.1005539 (PMC5448816; doi:10.1371/journal.pcbi.1005539)
Supplement: S1 Dataset — (ZIP) [file pcbi.1005539.s018.zip › S1 Dataset/SteadyCom/doc/SteadyCom/SteadyComPOACplex.html]

Description of SteadyComPOACplex


# SteadyComPOACplex

## PURPOSE

**Pairwise POA for community model at community steady-state for a range of growth rates**

## SYNOPSIS

**function [POAtable, fluxRange, Stat, GRvector] = SteadyComPOACplex(modelCom,options,solverParam)**

## DESCRIPTION

```
Pairwise POA for community model at community steady-state for a range of growth rates
[POAtable, fluxRange, Stat, GRvector] = SteadyComPOACplex(modelCom,options,solverParam)

INPUT
 modelCom       A community COBRA model structure with the following extra fields:
 (the following fields are required - others can be supplied)
   S            Stoichiometric matrix
   b            Right hand side
   c            Objective coefficients
   lb           Lower bounds
   ub           Upper bounds
 (at least one of the below two is needed)
   infoCom      structure containing community reaction info 
                (returned along with the community model created with createCommModel)
   indCom       the index structure corresponding to infoCom

 options (optional) option structure with the following fields:
   GRmax           Maximum growth rate of the model (default to be found
                     SteadyComCplex.m)
   optGRpercent    A vector of percentages. Perform POA at these percents
                     of max. growth rate respectively (Default = 99.99)
   optBMpercent    Only consider solutions that yield at least a certain
                     percentage of the optimal biomass (Default = 95)
   rxnNameList     List of reactions (IDs or .rxns) to be analyzed.
                     Use a (N_rxns + N_organism) x K matrix for POA of K
                     linear combinations of fluxes and/or abundances
                     (Default = biomass reaction of each species)
   pairList        Pairs in rxnNameList to be analyzed. N_pair by 2 array of:
                     - indices referring to the rxns in rxnNameList, e.g.,
                       [1 2] to analyze rxnNameList{1} vs rxnNameList{2}
                     - rxn names which are members of rxnNameList, e.g.,
                       {'EX_glc-D(e)','EX_ac(e)'}
                     If not supplied, analyze all K(K-1) pairs from the K
                     targets in rxnNameList.
   symmetric       Used only when pairList is not supplied. Avoid running 
                     symmetric pairs (e.g. j vs k and k vs j)
   Nstep           Number of steps for fixing one flux at a value between 
                     the min. and the max. possible fluxes. Default 10.
                   Can also be a vector indicating the fraction of intermediate value to be analyzed
                   e.g. [0 0.5 1] means computing at minFlux, 0.5(minFlux + maxFlux) and maxFlux
   NstepScale      Used only when Nstep is a single number. 
                     -'lin' for a linear (uniform) scale of step size 
                     -'log' for a log scaling of the step sizes
   fluxRange       Flux range for each entry in rxnNameList. K x 2 matrix.
                     Defaulted to be found by SteadyComFVACplex.m
  (other parameters)
   savePOA         Must be non-empty. The filename to save the POA results
                     (default 'POAtmp/POA')
   threads         > 1 for explicitly stating the no. of threads used,
                     0 or -1 for using all available threads. Default 1.
   verbFlag        Verbose output. 0 or 1.
   loadModel       String of filename to be loaded. If non-empty, load the 
                     cplex model ('loadModel.mps'), basis ('loadModel.bas') 
                     and parameters ('loadModel.prm').
  May add also other parameters in SteadyComCplex for calculating the maximum growth rate.

OUTPUT
 (N_gr = numel(optGRpercent))
 POAtable          K x K cells.  
                     Each (i,i)-cell contains a Nstep x 1 x N_gr matrix of
                     the fluxes at which rxnNameList{i} is fixed
                     (i,j)-cell contains a Nstep x 2 x N_gr matrix,  
                     (p,:,q)-entry being the range of rxnNameList{j} 
                     when rxnNameList{i} is fixed at POAtable{i,i}(p,1,q)
                     at growth rate = GRvector(q)
 fluxRange         K x 2 x N_gr matrix of flux range for each entry in rxnNameList 
 Stat              K x K x N_gr structure array with fields:
                     -'cor': the slope from linear regression between the
                             fluxes of a pair
                     -'r2':  the corresponding coefficient of determination (R-square)
 GRvector          Vector of growth rates being analyzed
```

## CROSS-REFERENCE INFORMATION

This function calls:

- SteadyComCplex Find the maximum community growth rate at community steady-state using SteadyCom
- SteadyComPOAgrCplex Pairwise POA for community model at community steady-state at a given growth rate
- checkSolFeas Check the feasibility of a solution given a COBRA model structure or a CPLEX dynamic object and a solution
- getCobraComParams get the required default parameters
- infoCom2indCom Transform between community reaction IDs and reaction names
- setCplexParam Set the parameters of the CPLEX object according to the structure solverParam
- updateLPcom Create and update the SteadyCom LP model in CPLEX format.

This function is called by:


## SOURCE CODE

```
0001 function [POAtable, fluxRange, Stat, GRvector] = SteadyComPOACplex(modelCom,options,solverParam)
0002 %Pairwise POA for community model at community steady-state for a range of growth rates
0003 %[POAtable, fluxRange, Stat, GRvector] = SteadyComPOACplex(modelCom,options,solverParam)
0004 %
0005 %INPUT
0006 % modelCom       A community COBRA model structure with the following extra fields:
0007 % (the following fields are required - others can be supplied)
0008 %   S            Stoichiometric matrix
0009 %   b            Right hand side
0010 %   c            Objective coefficients
0011 %   lb           Lower bounds
0012 %   ub           Upper bounds
0013 % (at least one of the below two is needed)
0014 %   infoCom      structure containing community reaction info
0015 %                (returned along with the community model created with createCommModel)
0016 %   indCom       the index structure corresponding to infoCom
0017 %
0018 % options (optional) option structure with the following fields:
0019 %   GRmax           Maximum growth rate of the model (default to be found
0020 %                     SteadyComCplex.m)
0021 %   optGRpercent    A vector of percentages. Perform POA at these percents
0022 %                     of max. growth rate respectively (Default = 99.99)
0023 %   optBMpercent    Only consider solutions that yield at least a certain
0024 %                     percentage of the optimal biomass (Default = 95)
0025 %   rxnNameList     List of reactions (IDs or .rxns) to be analyzed.
0026 %                     Use a (N_rxns + N_organism) x K matrix for POA of K
0027 %                     linear combinations of fluxes and/or abundances
0028 %                     (Default = biomass reaction of each species)
0029 %   pairList        Pairs in rxnNameList to be analyzed. N_pair by 2 array of:
0030 %                     - indices referring to the rxns in rxnNameList, e.g.,
0031 %                       [1 2] to analyze rxnNameList{1} vs rxnNameList{2}
0032 %                     - rxn names which are members of rxnNameList, e.g.,
0033 %                       {'EX_glc-D(e)','EX_ac(e)'}
0034 %                     If not supplied, analyze all K(K-1) pairs from the K
0035 %                     targets in rxnNameList.
0036 %   symmetric       Used only when pairList is not supplied. Avoid running
0037 %                     symmetric pairs (e.g. j vs k and k vs j)
0038 %   Nstep           Number of steps for fixing one flux at a value between
0039 %                     the min. and the max. possible fluxes. Default 10.
0040 %                   Can also be a vector indicating the fraction of intermediate value to be analyzed
0041 %                   e.g. [0 0.5 1] means computing at minFlux, 0.5(minFlux + maxFlux) and maxFlux
0042 %   NstepScale      Used only when Nstep is a single number.
0043 %                     -'lin' for a linear (uniform) scale of step size
0044 %                     -'log' for a log scaling of the step sizes
0045 %   fluxRange       Flux range for each entry in rxnNameList. K x 2 matrix.
0046 %                     Defaulted to be found by SteadyComFVACplex.m
0047 %  (other parameters)
0048 %   savePOA         Must be non-empty. The filename to save the POA results
0049 %                     (default 'POAtmp/POA')
0050 %   threads         > 1 for explicitly stating the no. of threads used,
0051 %                     0 or -1 for using all available threads. Default 1.
0052 %   verbFlag        Verbose output. 0 or 1.
0053 %   loadModel       String of filename to be loaded. If non-empty, load the
0054 %                     cplex model ('loadModel.mps'), basis ('loadModel.bas')
0055 %                     and parameters ('loadModel.prm').
0056 %  May add also other parameters in SteadyComCplex for calculating the maximum growth rate.
0057 %
0058 %OUTPUT
0059 % (N_gr = numel(optGRpercent))
0060 % POAtable          K x K cells.
0061 %                     Each (i,i)-cell contains a Nstep x 1 x N_gr matrix of
0062 %                     the fluxes at which rxnNameList{i} is fixed
0063 %                     (i,j)-cell contains a Nstep x 2 x N_gr matrix,
0064 %                     (p,:,q)-entry being the range of rxnNameList{j}
0065 %                     when rxnNameList{i} is fixed at POAtable{i,i}(p,1,q)
0066 %                     at growth rate = GRvector(q)
0067 % fluxRange         K x 2 x N_gr matrix of flux range for each entry in rxnNameList
0068 % Stat              K x K x N_gr structure array with fields:
0069 %                     -'cor': the slope from linear regression between the
0070 %                             fluxes of a pair
0071 %                     -'r2':  the corresponding coefficient of determination (R-square)
0072 % GRvector          Vector of growth rates being analyzed
0073 
0074 %% Initialization
0075 %check required fields for community model
0076 if ~isfield(modelCom,'indCom')
0077     if ~isfield(modelCom,'infoCom') || ~isstruct(modelCom.infoCom) || ...
0078             ~all(isfield(modelCom.infoCom,{'spBm','EXcom','EXsp','spAbbr','rxnSps','metSps'}))
0079         error('infoCom must be provided for calculating the max. community growth rate.\n');
0080     end
0081     %get useful reaction indices
0082     modelCom.indCom = infoCom2indCom(modelCom);
0083 end
0084 
0085 %get paramters
0086 if ~exist('options', 'var')
0087     options = struct();
0088 end
0089 if ~exist('solverParam', 'var') || isempty(solverParam)
0090     %default Cplex parameters
0091     solverParam = getCobraComParams('CplexParam');
0092 end
0093 param2get = {'GRmax', 'optGRpercent', 'Nstep', 'GRfx','BMmaxLB','BMmaxUB',...
0094              'rxnNameList', 'verbFlag', 'savePOA','loadModel'};
0095 eval(sprintf('[%s] = getCobraComParams(param2get, options, modelCom);', ...
0096             strjoin(param2get, ',')...
0097             )...
0098     );
0099 if isempty(savePOA)
0100     error('A non-empty file name must be provided to save the POA results.');
0101 end
0102 [feasTol, ~] = getCobraSolverParams('LP',{'feasTol'; 'optTol'}, solverParam);
0103 if isfield(solverParam,'simplex') && isfield(solverParam.simplex, 'tolerances')...
0104         && isfield(solverParam.simplex.tolerances,'feasibility')
0105     %override the feasTol in CobraSolverParam if given in solverParam
0106     feasTol = solverParam.simplex.tolerances.feasibility;
0107 else
0108     %otherwise use the feasTol in COBRA toolbox
0109     solverParam.simplex.tolerances.feasibility = feasTol;
0110 end
0111 
0112 init = true;
0113 if exist([savePOA '_MasterModel.mat'], 'file')
0114     load([savePOA '_MasterModel.mat'],'LPstart','LPmodel','GRvector','kDisp','idRow');
0115     LP = Cplex('POA');
0116     LP.Model = LPmodel;
0117     LP.Start = LPstart;
0118     LP = setCplexParam(LP, solverParam);
0119     if ~isfield(options, 'BMmaxLB')
0120         options.BMmaxLB = LP.Model.lhs(idRow);
0121     end
0122     if ~isfield(options, 'BMmaxUB')
0123         options.BMmaxUB = LP.Model.rhs(idRow);
0124     end
0125     init = false;
0126 end
0127 if numel(Nstep) > 1
0128     Nstep = numel(Nstep);
0129 end
0130 if ischar(rxnNameList)
0131     rxnNameList = {rxnNameList};
0132 end
0133 if iscell(rxnNameList)
0134     Ncheck = numel(rxnNameList);
0135 else
0136     Ncheck = size(rxnNameList,2);
0137 end
0138 if init
0139     addRow = false;
0140     %get maximum growth rate
0141     if isempty(GRmax)
0142         if exist('Cplex.p','file') == 6
0143             options.minNorm = false;
0144             [~, result,LP] = SteadyComCplex(modelCom, options,solverParam);
0145         else
0146             %need further achitecture for using COBRA solver
0147             warning('Currently support Cplex only.');
0148             return
0149         end
0150         if strcmp(result.stat,'infeasible')
0151             %infeasible model
0152             warning('Model is infeasible.');
0153             POAtable = cell(Ncheck);
0154             fluxRange = NaN(Ncheck,2); 
0155             Stat = repmat(struct('cor',[],'r2',[]),Ncheck,Ncheck);
0156             GRvector = NaN(numel(optGRpercent), 1);
0157             return
0158         end
0159         GRmax = result.GRmax;
0160         idRow = size(LP.Model.A,1);
0161     else
0162         %If GRmax is given, BMmaxLB and BMmaxUB should be included in options in this case to ensure feasibility
0163         if ~isempty(loadModel)
0164             [m, n] = size(modelCom.S);
0165             nRxnSp = sum(modelCom.indCom.rxnSps > 0); %number of species-specific rxns
0166             nSp = numel(modelCom.indCom.spBm); %number of species
0167             % load solution if given and growth rate is known
0168             LP = Cplex('fluxSampling');
0169             LP.readModel([loadSol '.mps']);
0170             LP.readBasis([loadSol '.bas']);
0171             LP.readParam([loadSol '.prm']);
0172             fprintf('Load model ''%s'' successfully.\n', loadModel);
0173             addRow = true;
0174             if size(LP.Model.A,1) > m + 2*nRxnSp + nSp
0175                 [ynRow,idRow] = ismember(sparse(ones(nSp,1),n+1:n+nSp,ones(nSp,1),1,n+nSp),...
0176                     LP.Model.A(m+2*nRxnSp+nSp+1:end,1:n+nSp),'rows');
0177                 if ynRow
0178                     idRow = m + 2*nRxnSp + nSp + idRow;
0179                 end
0180                 addRow = ~ynRow;
0181             end
0182         else
0183             %get LP using SteadyComCplex if only growth rate is given
0184             options2 = options;
0185             options2.LPonly = true;
0186             [~, ~, LP] = SteadyComCplex(modelCom, options2, solverParam);
0187             %no constraint on total biomass using LPonly option
0188             addRow = true;
0189         end
0190     end
0191     if addRow
0192         %add a row for constraining the sum of biomass if not exist
0193         LP.addRows(BMmaxLB, ...
0194             sparse(ones(1, nSp), n + 1: n + nSp, ones(1, nSp), 1, size(LP.Model.A,2)),...
0195             BMmaxUB, 'UnityBiomass');
0196         idRow = size(LP.Model.A,1);
0197     else
0198         %using BMmaxLB and BMmaxUB stored in the LP if not given in options
0199         if ~isfield(options,'BMmaxLB') %take from LP if not supplied
0200             BMmaxLB = LP.Model.lhs(idRow);
0201         end
0202         if ~isfield(options,'BMmaxUB') %take from LP if not supplied
0203             BMmaxUB = LP.Model.rhs(idRow);
0204         end
0205         LP.Model.lhs(idRow) = BMmaxLB;
0206         %not allow the max. biomass to exceed the one at max growth rate,
0207         %can happen if optBMpercent < 100. May dismiss this constraint or
0208         %manually supply BMmaxUB in the options if sum of biomass should be variable
0209         LP.Model.rhs(idRow) = BMmaxUB;
0210     end
0211     %set Cplex parameters
0212     LP = setCplexParam(LP, solverParam);
0213     LP.Model.A = updateLPcom(modelCom, GRmax, GRfx, [], LP.Model.A, []);
0214     LP.Model.sense = 'minimize';
0215     LP.Model.obj(:) = 0;
0216     LP.solve();
0217     %check and adjust for feasibility
0218     dev = checkSolFeas(LP);
0219     kBMadjust = 0;
0220     BMmaxLB = LP.Model.lhs(idRow);
0221     while (~isfield(LP.Solution, 'x') || dev > feasTol) && kBMadjust < 10
0222         kBMadjust = kBMadjust + 1;
0223         %row of biomass constraint should at the end
0224         LP.Model.lhs(idRow) = BMmaxLB * (1 - feasTol/(11 - kBMadjust));
0225         LP.solve();
0226         dev = checkSolFeas(LP);
0227         if verbFlag
0228             fprintf('BMmax adjusment: %d\n',kBMadjust);
0229         end
0230     end
0231     if (~isfield(LP.Solution, 'x') || dev > feasTol)
0232         error('Model not feasible.')
0233     end
0234     if ~isfield(options, 'BMmaxLB')
0235         options.BMmaxLB = LP.Model.lhs(idRow);
0236     end
0237     if ~isfield(options, 'BMmaxUB')
0238         options.BMmaxUB = LP.Model.rhs(idRow);
0239     end
0240 
0241     GRvector = GRmax * optGRpercent/100;
0242     if numel(optGRpercent) == 1
0243         kDisp = 2;
0244     else
0245         d = max(GRvector(2:end) - GRvector(1:end-1));
0246         if d < 1
0247             kDisp = abs(floor(log10(abs(d))));
0248         else
0249             kDisp = 0;
0250         end
0251     end
0252 end
0253 
0254 nVar = size(LP.Model.A,2);
0255 %handle objective matrix
0256 if isnumeric(rxnNameList)
0257     if size(rxnNameList,1) >= size(modelCom.S,2) && size(rxnNameList,1) <= nVar
0258         %it is a matrix of objective vectors
0259         objList = [sparse(rxnNameList); sparse(nVar - size(rxnNameList,1), size(rxnNameList,2))];
0260     elseif size(rxnNameList,1) == 1 || size(rxnNameList,2) == 1 
0261         %reaction index
0262         objList = sparse(rxnNameList, 1:numel(rxnNameList), ones(numel(rxnNameList),1),...
0263             nVar, max(size(rxnNameList)));
0264     else
0265         error('Invalid numerical input of rxnNameList.');
0266     end
0267 elseif iscell(rxnNameList)
0268     objList = sparse(nVar, numel(rxnNameList));
0269     for jRxnName = 1:numel(rxnNameList)
0270         rJ = findRxnIDs(modelCom,rxnNameList{jRxnName});
0271         if ~all(rJ)
0272             error('Invalid names in rxnNameList');
0273         end
0274         objList(rJ,jRxnName) = 1;
0275     end
0276 else
0277     error('Invalid input of rxnNameList');
0278 end
0279 options.rxnNameList = objList;
0280 clear objList
0281 
0282 if isempty(savePOA)
0283     %always use save option to reduce memory need
0284     savePOA = ['POAtmp' filesep 'POA'];
0285 end
0286 directory = strsplit(savePOA,filesep);
0287 if numel(directory) > 1
0288     %not saving in the current directory. Check existence
0289     directory = strjoin(directory(1:end-1),filesep);
0290     if ~exist(directory,'dir')
0291         mkdir(directory);
0292     end
0293 end
0294 if init
0295     LPstart = LP.Start;
0296     LPmodel = LP.Model;
0297     save([savePOA '_MasterModel.mat'],'LPstart','LPmodel','GRvector', 'kDisp','idRow','GRmax');
0298 end
0299 for j = 1:numel(GRvector)
0300     optionsJ = options;
0301     optionsJ.GR = GRvector(j);
0302     optionsJ.savePOA = sprintf(['%s_GR%.' num2str(kDisp) 'f'], savePOA, GRvector(j));  
0303     %better reset the model to ensure feasibility
0304     if j > 1
0305         load([savePOA '_MasterModel.mat'],'LPstart','LPmodel');
0306         LP.Model = LPmodel;
0307         LP.Start = LPstart;
0308         LP = setCplexParam(LP, solverParam);
0309     end
0310     clear LPmodel LPstart
0311     SteadyComPOAgrCplex(modelCom,optionsJ,solverParam,LP);
0312 end
0313 
0314 %collect output from save file
0315 POAtable = cell(Ncheck, Ncheck);
0316 Stat = repmat(struct('cor',0,'r2',0), [Ncheck, Ncheck, numel(GRvector)]);
0317 fluxRange = zeros(Ncheck, 2, numel(GRvector));
0318 
0319 for j = 1:numel(GRvector)
0320     data = load(sprintf(['%s_GR%.' num2str(kDisp) 'f.mat'],savePOA,GRvector(j)), 'POAtable', 'fluxRange', 'Stat');
0321     for p = 1:Ncheck
0322         for q = 1:Ncheck
0323             if isempty(data.POAtable{p,q})
0324                 POAtable{p,q} = [];
0325             elseif size(data.POAtable{p,q}, 1) == 1
0326                 %single point, no range
0327                 POAtable{p,q}(:, :, j) = repmat(data.POAtable{p,q}, Nstep, 1);
0328             else
0329                 POAtable{p,q}(:, :, j) = data.POAtable{p,q};
0330             end
0331             Stat(p,q,j).cor = data.Stat(p,q).cor;
0332             Stat(p,q,j).r2 = data.Stat(p,q).r2;
0333             fluxRange(:,:, j) = data.fluxRange;
0334         end
0335     end
0336 end
0337 
0338 end
```

---

Generated on Sat 06-May-2017 09:55:30 by **m2html** © 2005
